# Multicomponent double Mannich alkylamination involving C(sp^2^)–H and benzylic C(sp^3^)–H bonds

**DOI:** 10.1038/s41467-022-28088-z

**Published:** 2022-01-21

**Authors:** Zhencheng Lai, Rongkai Wu, Jiaming Li, Xing Chen, Linwei Zeng, Xi Wang, Jingjing Guo, Zujin Zhao, Hironao Sajiki, Sunliang Cui

**Affiliations:** 1 Institute of Drug Discovery and Design, College of Pharmaceutical Sciences, Zhejiang University, 866 Yuhangtang Road, Hangzhou, 310058 China; 2 Department of Chemistry, Zhejiang University, 38 Zheda Road, Hangzhou, 310027 China; 3 Institute of Molecular Plus, Tianjin University, Tianjin, 300072 China; 4 School of Science, Beijing Jiaotong University, Beijing, 100044 China; 5 State Key Laboratory of Luminescent Materials and Devices, South China University of Technology, Guangzhou, 510640 China; 6 Laboratory of Organic Chemistry, Gifu-Pharmaceutical University, Gifu, 501-1196 Japan

**Keywords:** Diversity-oriented synthesis, Synthetic chemistry methodology

## Abstract

Alkylamines are ubiquitous in pharmaceuticals, materials and agrochemicals. The Mannich reaction is a well-known three-component reaction for preparing alkylamines and has been widely used in academic research and industry. However, the nucleophilic components in this process rely on C(sp^2^)−H and activated C(sp^3^)−H bonds while the unactivated C(sp^3^)−H bonds involved Mannich alkylamination is a long-standing challenge. Here, we report an unprecedented multicomponent double Mannich alkylamination for both C(sp^2^)−H and unactivated benzylic C(sp^3^)−H bonds. In this process, various 3-alkylbenzofurans, formaldehyde and alkylamine hydrochlorides assemble efficiently to furnish benzofuran-fused piperidines. Mechanistic studies and density functional theory (DFT) calculations revealed a distinctive pathway that a multiple Mannich reaction and retro-Mannich reaction of benzofuran and dehydrogenation of benzylic C(sp^3^)−H bonds were key steps to constitute the alkylamination. This protocol furnishes a Mannich alkylamine synthesis from unusual C–H inputs to access benzofuran-fused piperidines with exceptional structural diversity, molecular complexity and drug-likeness. Therefore, this work opens a distinctive vision for the alkylamination of unactivated C(sp^3^)−H bonds, and provides a powerful tool in diversity-oriented synthesis (DOS) and drug discovery.

## Abstract (web summary)

The Mannich reaction is a three-component reaction for preparing alkylamines, but the nucleophilic components rely on C(sp^2^)−H and activated C(sp^3^)−H bonds. Here, the authors report an unprecedented multicomponent double Mannich alkylamination for both C(sp^2^)−H and unactivated benzylic C(sp^3^)−H bonds.

## Introduction

**Fig. 1:**
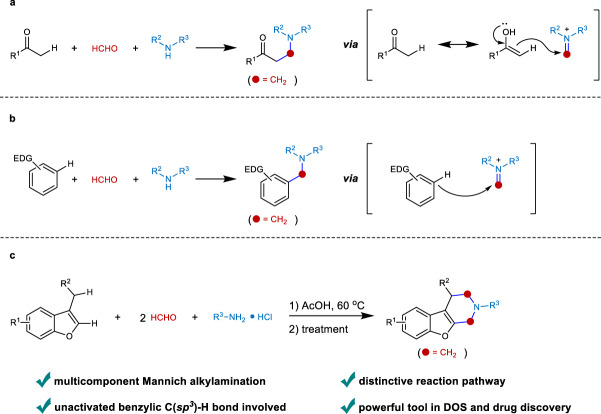
Overview of Mannich reaction. **a** Classical Mannich reaction involving activated C(sp^3^)−H bond. **b** Mannich reaction involving C(sp^2^)−H bond of electron-rich arenes. **c** This work of double Mannich alkylamination for both C(sp^2^)−H and unactivated benzylic C(sp^3^)−H bond.

Alkylamines are fundamental compounds in pharmaceuticals, materials, and agrochemicals^1,2^. For their synthesis, the Mannich reaction represents one of the most efficient approaches to access these molecules^3^. Discovered in 1912, the Mannich reaction is a prototypical three-component reaction that rapidly assembles a resonance-stabilized carbon nucleophile, an aldehyde (or ketone) and an amine to afford alkylamines. The mechanistic manifold involves iminium ion formation for sequential attack by a nucleophile. Owing to the readily accessible feedstocks, simple manipulation, and privileged product formation, the Mannich reaction is well suited for diversity-oriented synthesis (DOS) applicable in drug discovery, and its utility is demonstrated in alkaloid synthesis and the development of the antiulcer drug Ranitidine^4–11^. Recently, the asymmetric version of Mannich reaction has attracted much interests^12–22^. Despite these advances, the nucleophilic components of the Mannich reaction rely on Brønsted-acidic carbonyl C(sp^3^)−H bonds (Fig. 1a) and C(sp^2^)−H centers of electron-rich arenes (Fig. 1b)^23,24^, while the development of a Mannich reaction using unactivated C(sp^3^)–H bonds has remained a formidable challenge.

**Fig. 2:**
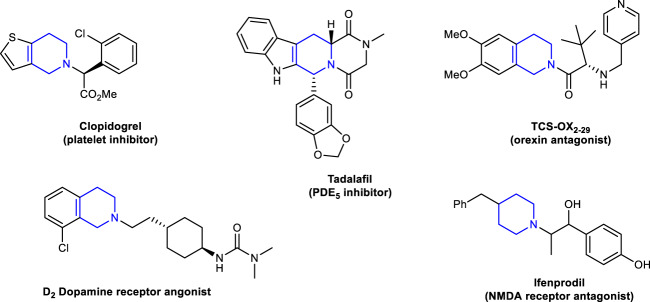
Pharmaceutical relevant examples of piperidines. Piperidine is widely existed in natural products and serves as the most commonly used nitrogen heterocycle among U.S. FDA-approved pharmaceuticals.

As noted, the wide prevalence of alkylamines in small molecule biological probes, pre-clinical candidates, and approved drugs is owing to their favorable physical and pharmacological properties^25,26^. In particular, cyclic alkylamines can enhance structural rigidity, thus improving target selectivity and druggability. For example, the cyclic six-membered piperidines represent the most commonly used nitrogen heterocycles among U.S. FDA-approved pharmaceuticals (Fig. 2). As a highly relevant synthetic strategy, the double Mannich reaction based on enolizable C−H bonds provides straightforward access to cyclic alkylamines. This was exemplified by Robinson for the facile synthesis of tropinone^27^. To engage unactivated C(sp^3^)−H bonds in this process would significantly expand the synthetic toolbox for the assembly of cyclic alkylamines and drug candidates. Based on serendipitous findings, herein, we report on the discovery of a multicomponent double Mannich reaction for both C(sp^2^)−H and unactivated benzylic C(sp^3^)−H bonds (Fig. 1c). In this process, various 3-alkylbenzofurans, formaldehyde, and primary alkylamine hydrochlorides assemble efficiently to furnish diverse benzofuran-fused piperidines. In addition, many bioactive molecules could be applicable in this protocol to prepare their analogs.

## Results

### Reaction design and optimization for double Mannich reaction

As mentioned above, the challenge for unactivated C(sp^3^)−H bonds to involve in Mannich reactions is attributed to their difficult deprotonation and therefore non-nucleophilic properties. In continuation of our interest in multi-component reactions and drug discovery^28–31^, we hypothesized that the benzylic C(sp^3^)−H bond might possess sufficient reactivity through the electronic effect of (hetero)arenes, thus enabling Mannich type alkylamination. After considerable exploration and optimization of conditions, we realized this hypothesis using electron-rich benzofurans as heteroaryl substrates. As shown in Table 1, the model reaction was explored with benzofuran **1a**, formaldehyde **2**, and glycine methyl ester hydrochloride **3a**. In the solvent of acetic acid (AcOH), these three components were mixed in a one-pot and kept at 60 °C for 3 h (entries 1–6). The amounts of **2** and **3a** were varied while a major product was formed and identified as benzofuran-fused piperidine **4a** in 74% yield (entry 6). This indicated an efficient double Mannich alkylamination involving both the benzylic C(sp^3^)−H bond and the aryl C(sp^2^)−H bond, furnishing the cyclic amine. Increasing the reaction temperature would decrease the yield (entries 7–8) and only 25% yield could be obtained when conducted at room temperature (entry 9). The survey of solvents showed that acetonitrile (CH_3_CN) worked equally well (entry 10), while the utilization of isopropanol, hexafluoroisopropanol (HFIP), and HFIP/AcOH would give inferior results (entries 11–13). Variation of **3a** to glycine methyl ester would lead to a decreased yield (entry 14). Conducting this reaction on 5 mmol scales could afford **4a** with slightly improved yield (entry 15), thus underscoring the scalability of this process.

**Table 1:** Reaction optimization^a^.


Entry	2 (equiv.)	3a (equiv.)	Solvent	T	Yield (%)^b^
1	2.5	1	AcOH (0.5 mL)	60 °C	45
2	2.5	2	AcOH (0.5 mL)	60 °C	40
3	4	1	AcOH (0.5 mL)	60 °C	63
4	4	2	AcOH (0.5 mL)	60 °C	72
5	4	2	AcOH (1 mL)	60 °C	74
6	4	2	AcOH (2 mL)	60 °C	62
7	4	2	AcOH (0.5 mL)	80 °C	60
8	4	2	AcOH (0.5 mL)	100 °C	Trace
9	4	2	AcOH (0.5 mL)	r. t.	25
10	4	2	CH_3_CN (1 mL)	60 °C	72
11	4	2	*i*-PrOH	60 °C	20
12	4	2	HFIP (0.5 mL)	50 °C	33
13	4	2	HFIP/AcOH (1 mL, 1:1)	60 °C	51
14^c^	4	2	AcOH (1 mL)	60 °C	65
15^d^	4	2	AcOH (20 mL)	60 °C	78

^a^ Reaction conditions: **1a** (0.2 mmol), **2**, **3a**, solvent, 3 h.

^b^ Isolated yields.

^c^ Methyl glycinate was used as an amine source. HFIP = (CF_3_)_2_CHOH.

^d^ 5 mmol scale.

### Substrate scope in the synthesis of benzofuran-fused piperidines

**Fig. 3:**
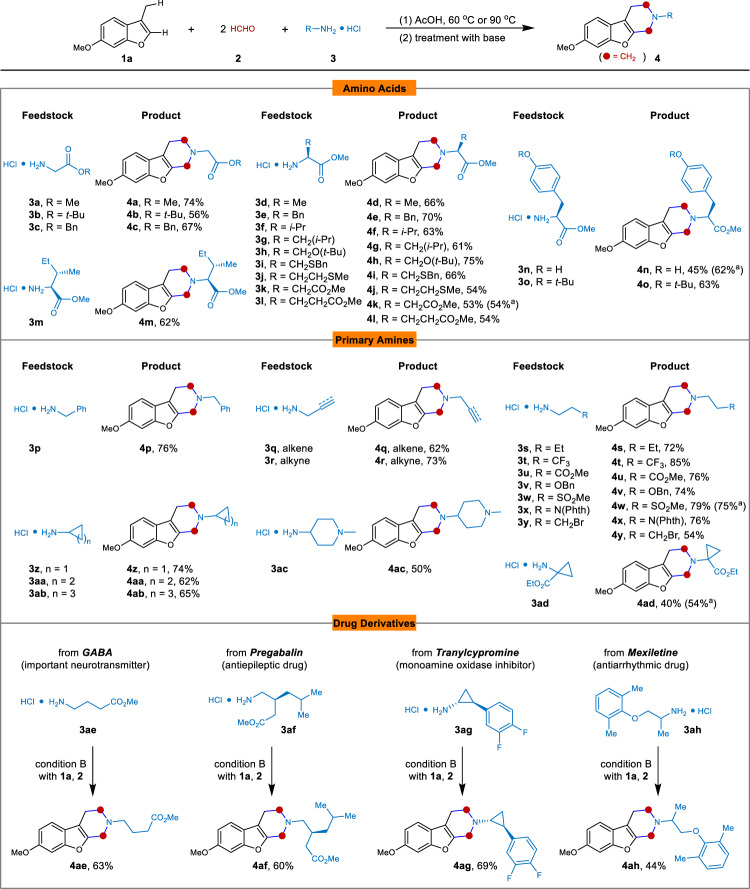
Substrate scope of amines. Condition A (for **3a**–**3o**): **1a** (0.2 mmol), **2** (0.8 mmol), **3** (0.4 mmol) in AcOH (2 mL) at 60 °C, 3 h; Condition B (for **3p**-**3ah**): **1a** (0.2 mmol), **2** (1.6 mmol), **3** (0.8 mmol) in AcOH (2 mL) at 90 °C, 30 mins. Yields refer to isolated products. N(Phth) = *N*-phthalimide. ^a^CH_3_CN (2 mL) as solvent, 8 h.

Under the optimized conditions, we examined the substrate scope of this three-component double Mannich alkylamination. Various amino acid derivatives, including glycine, l-alanine, l-phenylalanine, l-valine, l-leucine, l-serine, l-cysteine, l-methionine, l-aspartic acid, l-glutamic acid, l-tyrosine, and l-isoleucine, all reacted smoothly with benzofuran **1a** and formaldehyde **2** to furnish the corresponding fused piperidines in moderate to good yields (**4a**-**4o**) (Fig. 3). The structure of **4b** and **4n** was confirmed by X-ray analysis. Notably, the chirality and functionalities of the amino acids were retained in this process thus bestowing versatile physical and pharmacological properties on the products (see Supplementary Information). Besides, numerous primary amines hydrochlorides could engage in this process at 90 °C, and benzyl, alkene, alkyne, trifluoromethyl, ester, alcohol, sulfone, amino and bromoalkyl functionalities were well tolerated. This offers ample opportunity for further functionalization towards drug discovery and small molecule biological probe development. The structure of **4r** was confirmed by X-ray analysis. Moreover, three-, four-, five-, and six-membered ring-substituted amines were used to furnish the products in moderate to good yields (**4z**-**4ac**). Furthermore, a primary amine substituted with a quaternary carbon center was used, and also in this case the cyclization took place, giving **4ad** in moderate yield. Additionally, the late-stage modification of drug derivatives could be achieved using this method. For instance, the important neurotransmitter GABA (γ-aminobutyric acid) methyl ester hydrochloride **3ae**, the antiepileptic drug Pregabalin methyl ester hydrochloride **3af**, the monoamine oxidase inhibitor Tranylcypromine hydrochloride **3ag**, and the antiarrhythmic drug Mexiletine hydrochloride **3ah**, could all be efficiently modified under this protocol to produce the corresponding piperidine-hybrids in moderate yields (**4ae**-**4ah**). In addition, the utilization of CH_3_CN as solvent also works well with extended reaction time.

**Fig. 4:**
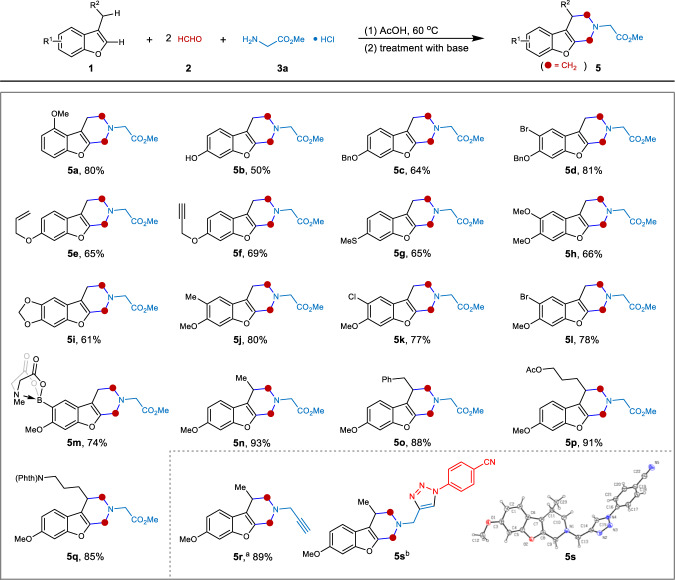
Substrate scope of heteroarenes. Reactions were carried out under condition A. Yields refer to isolated products. N(Phth) = *N*-phthalimide. ^a^**3a** was replaced with propargylamine hydrochloride under condition B. ^b^**5s** was prepared from **5r** via click reaction.

With respect to heteroarenes, a variety of electron-rich 3-alkylbenzofurans underwent the three-component double Mannich alkylaminaion efficiently, occurring at the 2-position of the benzofuran and the benzylic moiety (Fig. 4). Diverse functionalized substituents were tolerated in this process, including alkyloxy, allyloxy, propargyloxy, hydroxy, alkylthio, alkyl, chloro, bromo, and boronate (**5a**-**5m**). It should be noted that these functionalities not only bestow the products with remarkable structural diversity and favorable physical properties, but also provides ample potential for further derivatization. For example, the boronate compound **5m** might be transformed to a variety of molecules through Suzuki coupling reaction and Chan-Lam coupling reaction. When benzofurans with a substituted benzyl group like 3-ethyl, 3-(2-phenyl)ethyl, 3-(4-hydroxy)butyl, and 3-(4-amino)butyl were used, interestingly, the double Mannich reaction proceeded well to give the poly-substituted and functionalized piperidines in excellent yields (**5n**-**5r**). Besides, the hydroxy and amino group in the substituted chain would offer numerous late-stage functionalization opportunities. Moreover, compound **5r** was further subjected to a Huisgen click reaction to yield a triazole derivative **5s**, whose structure was confirmed by X-ray analysis. Considering that the piperidines are prevalent motifs in natural products as well as pharmaceuticals and are important synthetic targets for drug discovery and development^32^, this process provides an efficient and direct approach to establish libraries with high drug-likeness, broad chemical space, and modifiable physicochemical properties.

### Mechanistic investigation

**Fig. 5:**
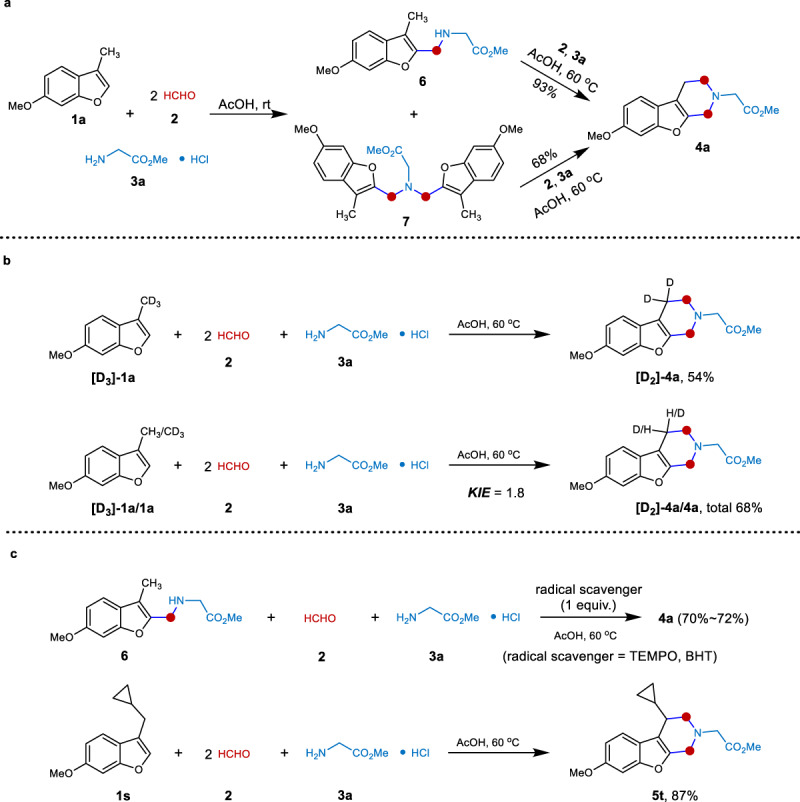
Mechanistic studies. **a** Control reaction. **b** Kinetic isotope effect experiments. **c** Radical-probe experiments.

Next, mechanistic studies were conducted to gain insights into the mechanism. (i) Control reactions (Fig. 5a): when this three-component reaction was carried out at room temperature, the common Mannich reaction occurred to deliver a mixture of **6** and **7** (**6**, 28% isolated yield; **7**, 70% isolated yield). **6** was a Mannich alkylamination product at the 2-position of benzofuran, and **7** was a dialkylated Mannich amine. Interestingly, **6** and **7** could interconvert under this condition (see Supplementary Information). Furthermore, they could react with **2a** and **3** to deliver product **4a** in yields of 93% and 68%, respectively, indicating that **6** and **7** were probable intermediates of this process. (ii) Kinetic isotopic effect experiments (Fig. 5b): benzylic deuterium-labeled **[D**_**3**_**]-1a** was prepared and combined with **2a** and **3** under standard reaction conditions, leading to the corresponding product **[D**_**2**_**]-4a** in 54% yield. Additionally, a kinetic isotope effect (KIE) study was carried out and a primary KIE value of 1.8 was observed, suggesting that the C(sp^3^)−H bond cleavage might be involved in the rate-limiting step. (iii) Radical-probe experiments (Fig. 5c): a radical scavenger was added to the one-pot reaction including **6**, **2**, and **3a** to probe the involvement of a radical intermediate. Ether the addition of TEMPO (2,2,6,6-tetramethylpiperidinooxy, 1 equiv.) or BHT (butylated hydroxytoluene, 1 equiv.) did not suppress the Mannich reaction. In addition, the cyclopropane-containing substrate **1** **s** was prepared and subjected to the reaction, and a piperidine product **5t** was isolated in 87% yield with cyclopropane retaining. These suggested exclusion of radical intermediates in this reaction^33–44^.

**Fig. 6:**
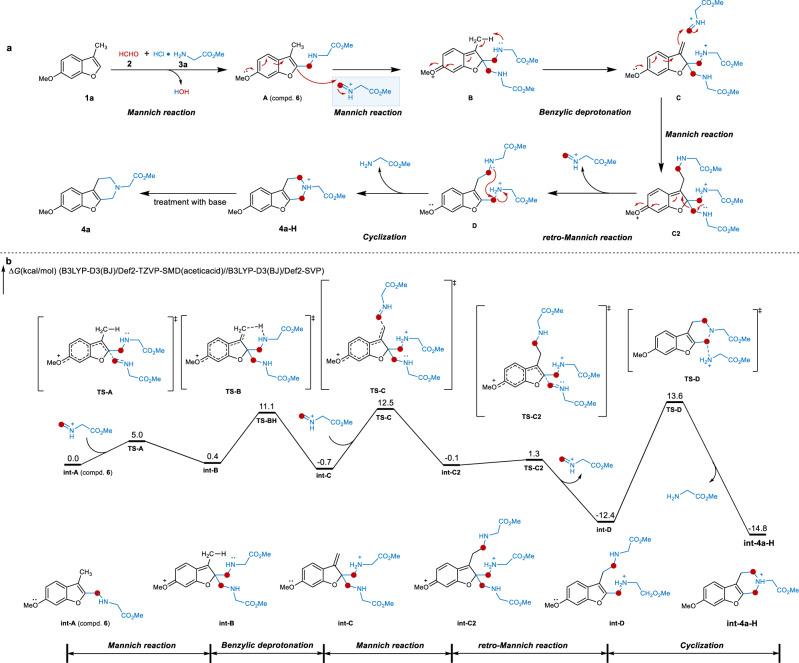
Plausible mechanism. **a** Proposed mechanism. **b** DFT calculation.

On the basis of these observations, a plausible mechanism was proposed with starting materials **1a**, formaldehyde **2**, and methyl glycinate hydrochloride **3a** as model substrates (Fig. 6a). Initially, the electron-rich 3-methylbenzofuran **1a**, formaldehyde **2**, and methyl glycinate hydrochloride **3a** undergo the first Mannich alkylamination to deliver intermediate **A** (compd. **6**). **A** undergoes the secondary Mannich reaction to furnish the cationic intermediate **B**. Subsequently, a benzylic C−H deprotonation of **B** occurs to generate the dearomatized alkenyl intermediate **C**. **C** further engages in another Mannich reaction to give the cationic intermediate **C2**. From **C2**, the retro-Mannich reaction leads to the intermediate **D**, and **D** undergoes the intramolecular nucleophilic substitution/cyclization to furnish the benzofuran-fused piperidine derivative **4a-H**. **4a-H** is eventually quenched by base to provide the observed product **4a**. Based on this mechanistic proposal, the electron-rich property of the benzofuran ring makes the multiple Mannich reaction and retro-Mannich reaction energetically feasible and eventually enables cyclization/alkylamination involving the benzylic C−H moiety. Consistent with this, when benzofurans with 3-(CH_2_R) substitution are used as inputs, the alkene intermediates **C** with poly-substituted alkene moiety are likely stabilized, thus explaining the high yields despite steric congestion (**5n**-**5r**).

### Density functional theory calculations

To further explore the plausibility of our proposal, we performed a series of density functional theory (DFT) calculations. We focus on the reaction triggered by the protonation at 2-position of the benzofuran due to its prominent nucleophilicity^45^. As shown in Fig. 6b, From the intermediate **int-A** (which can be isolated as compd. **6**), the Mannich reaction via **TS-A** is quite facile with a 5.0 kcal/mol barrier, which generates the dearomatized intermediate **int-B** in equilibrium. Subsequent intramolecular benzylic deprotonation proceeds via **TS-B** to produce the alkene intermediate **int-C**. This step requires a barrier of 10.7 kcal/mol (**int-B** to **TS-B**). From **int-C**, the Mannich reaction via **TS-C** has a 13.2 kcal/mol energy barrier and produces the intermediate **int-C2**. The retro-Mannich reaction of **int-C2** is quite efficient with only 1.4 kcal/mol barrier and exergonic, generating the benzofuran intermediate **int-D**. The final intramolecular nucleophilic substitution via **TS-D** allows the cyclization to give **int-4a-H**, which requires a barrier of 26.0 kcal/mol. Based on the additional DFT calculations, the formation of **int-D** is efficient and favorable. **int-D** is the on-cycle resting state, and the rate-determining step is the cyclization process via **TS-D**. These calculations validated the revised reaction mechanism, which discarded the original proposal involving the protonation of iminium intermediate. These DFT calculations revealed that the proposed reaction mechanism is rational.

## Discussion

In summary, we have presented an unprecedented multicomponent double Mannich alkylamination for both C(sp^2^)−H and unactivated benzylic C(sp^3^)−H bonds. A series of 3-alkylbenzofurans, formaldehyde, and primary alkylamine hydrochlorides could rapidly assemble to produce the piperidines. Mechanistic studies and DFT calculations revealed a distinctive pathway that a multiple Mannich reactions and retro-Mannich reaction of benzofuran and dehydrogenation of benzylic C(sp^3^)−H bonds were key steps to constitute the alkylamination, which opens a distinctive vision for alkylamination and functionalization of unactivated C(sp^3^)−H bonds. In addition, this protocol also provides a powerful tool in diversity-oriented synthesis and drug discovery.

## Supplementary information

Supplementary Information

## Data Availability

Materials and methods, experimental procedures, useful information, mechanistic studies, optimization studies, ^1^H NMR spectra, ^13^C NMR spectra, and mass spectrometry data are available in the Supplementary Information. Additional data are available from the corresponding author upon request. Crystallographic data for compounds **4b**, **4n**, **4r**, **5s**, are available from the Cambridge Crystallographic Data Center under reference number CCDC 2020613, 2020614, 2020615, and 2027076. These data can be obtained free of charge via https://www.ccdc.cam.ac.uk/structures/.

## References

[1] Quellette, R. J., Rawn, J. D. in *Organic Chemistry: Structure, Mechanism and Synthesis*, Chapter 23, pp. 803–842 (Elsevier, 2014).

[2] Trowbridge A, Reich D, Gaunt MJ (2018). Multicomponent synthesis of tertiary alkylamines by photocatalytic olefin-hydroaminoalkylation. Nature.

[3] Arend M, Westermann B, Risch N (1998). Modern variants of the mannich reaction. Angew. Chem. Int. Ed..

[4] Overmann, L. E., Ricca, D. J. in *Comprehensive Organic Synthesis* (Pergamon, Oxford, 1991), Vol. 2, p. 1007–1046.

[5] Shi Y, Wang Q, Gao S (2018). Recent advances in the intramolecular Mannich reaction in natural products total synthesis. Org. Chem. Front..

[6] Martin SF, Barr KJ, Smith DW, Bur SK (1999). Applications of vinylogous mannich reactions. concise eanantiospecific total syntheses of (+)-croomine. J. Am. Chem. Soc..

[7] Earley WG (2005). Aza-cope rearrangement-mannich cyclizations for the formation of complex tricyclic amines: stereocontrolled total synthesis of (±)-gelsemine. J. Am. Chem. Soc..

[8] Ge HM, Zhang L-D, Tan RX, Yao Z-J (2012). Protecting group-free total synthesis of (-)-lannotinidine B. J. Am. Chem. Soc..

[9] Numajiri Y, Pritchett BP, Chiyoda K, Stoltz BM (2015). Enantioselective synthesis of α‑quaternary mannich adducts by palladium-catalyzed allylic alkylation: total synthesis of (+)-sibirinine. J. Am. Chem. Soc..

[10] Lisnyak VG, Snyder SA (2020). A Concise, enantiospecific total synthesis of chilocorine C fueled by a reductive cyclization/mannich reaction cascade. J. Am. Chem. Soc..

[11] Brogden RN, Carmine AA, Heel RC, Speight TM, Avery GS (1982). Ranitidine: a review of its pharmacology and therapeutic use in peptic ulcer disease and other allied diseases. Drugs.

[12] Verkade JMM, van Hemert LJC, Quaedflieg PJLM, Rutjes FPJT (2008). Organocatalysed asymmetric Mannich reactions. Chem. Soc. Rev..

[13] Arrayás RG, Carretero JC (2009). Catalytic asymmetric direct Mannich reaction: a powerful tool for the synthesis of α, β-diamino acids. Chem. Soc. Rev..

[14] List B (2000). The direct catalytic asymmetric three-component mannich reaction. J. Am. Chem. Soc..

[15] Notz W, Sakthivel K, Bui T, Zhong G, Barbas CF (2001). Amine-catalyzed direct asymmetric Mannich-type reactions. Tetrahedron Lett..

[16] Saruhashi K, Kobayashi S (2006). Remarkably stable chiral zirconium complexes for asymmetric mannich-type reactions. J. Am. Chem. Soc..

[17] Guo Q-X (2007). Chiral brønsted acid-catalyzed direct asymmetric mannich reaction. J. Am. Chem. Soc..

[18] Zhao J (2013). Asymmetric synthesis of β-amino nitriles through a Sc^III^-catalyzed three-component mannich reaction of silyl ketene imines. Angew. Chem. Int. Ed..

[19] Lin S, Kawato Y, Kumagai N, Shibasaki M (2015). Catalytic asymmetric mannich-type reaction of *N*-alkylidene-α-aminoacetonitrile with ketimines. Angew. Chem. Int. Ed..

[20] Kano T, Kobayashi R, Maruoka K (2015). Versatile in situ generated *N*-Boc-imines: application to phase-transfer-catalyzed asymmetric mannich-type reactions. Angew. Chem. Int. Ed..

[21] Trost BM, Saget T, Hung C-I (2016). Direct catalytic asymmetric mannich reactions for the construction of quaternary carbon stereocenters. J. Am. Chem. Soc..

[22] Chen J (2018). Carbonyl catalysis enables a biomimetic asymmetric Mannich reaction. Science.

[23] Noble A, Anderson JC (2013). Nitro-mannich reaction. Chem. Rev..

[24] Grumbach H-J, Arend M, Risch N (1996). Aminoalkylation of electron-rich aromatic compounds using performed iminium salts derived from aldehydes other than formaldehyde. Synthesis.

[25] Vitaku E, Smith DT, Njardarson JT (2014). Analysis of the structural diversity, substitution patterns, and frequency of nitrogen heterocycles among U.S. FDA approved pharmaceuticals. J. Med. Chem..

[26] Blakemore DC (2018). Organic synthesis provides opportunities to transform drug discovry. Nat. Chem..

[27] Robinson R (1917). A synthesis of tropinone. J. Chem. Soc..

[28] Huang B, Zeng L, Shen Y, Cui S (2017). One-pot multicomponent synthesis of β-amino amides. Angew. Chem. Int. Ed..

[29] Zeng L, Huang B, Shen Y, Cui S (2018). Multicomponent synthesis of tetrahydroisoquinolines. Org. Lett..

[30] Chen C (2019). Discovery of 3,6-diaryl-1*H*-pyrazolo[3,4-*b*]pyridines as potent anaplastic lymphoma kinase (ALK) inhibitors. Bioorg. Med. Chem. Lett..

[31] Wang C, Lai Z, Xie H, Cui S (2021). Triazenyl alkynes as versatile building blocks in multicomponent reactions: diastereoselective synthesis of β-amino amides. Angew. Chem. Int. Ed..

[32] Duttwyler S (2013). Proton donor acidity controls selectivity in nonaromatic nitrogen heterocycle synthesis. Science.

[33] Zhang W (2016). Enantioselective cyanation of benzylic C−H bonds via copper-catalyzed radical relay. Science.

[34] Xu W, Mariano PS (1991). Substituted effects on amine cation radical acidity. regiocontrol of β-(aminoethyl)cyclohexenone photocyclization). J. Am. Chem. Soc..

[35] Kumar R, Flodén NJ, Whitehurst WG, Gaunt MJ (2020). A general carbonyl alkylative amination for tertiary amine synthesis. Nature.

[36] Suga S, Suzuki S, Yoshida J-I (2002). Reduction of a “cation pool”: a new approach to radical mediated C−C bond formation. J. Am. Chem. Soc..

[37] Horibe T, Ohmura S, Ishihara K (2019). Structure and reactivity of aromatic radical cations generated by FeCl_3_. J. Am. Chem. Soc..

[38] Seo H, Katcher MH, Jamison TF (2017). Photoredox activation of carbon dioxide for amino acid synthesis in continuous flow. Nat. Chem..

[39] Woźniak L, Maganano G, Melchiorre P (2018). Enantioselective photochemical organocascade catalysis. Angew. Chem. Int. Ed..

[40] Flodén NJ (2019). Streamlined synthesis of C(*sp*^*3*^)-rich N-heterospirocycles enabled by visible-light-mediated photocatalysis. J. Am. Chem. Soc..

[41] Andrieux CP, Savéant JM (1970). Electrodimerization: II. Reduction mechanism of immonium cations. J. Electroanal. Chem..

[42] Pirnot MT, Rankic DA, Martin DBC, MacMillan DWC (2013). Photoredox activation for the direct β-arylation of ketones and aldehydes. Science.

[43] Liang Z (2009). Oxidative aromatic C−O bond formation: synthesis of 3-functionalized benzo[*b*]furans by FeCl_3_-mediated ring closure of α-aryl ketones. Org. Lett..

[44] Broere DLJ (2014). Intramolecular reodx-active ligand-to-substrate single-electron transfer: radical reactivity with a palladium(II) complex. J. Am. Chem. Soc..

[45] Jones, G., Stanforth, S. P. in *Organic Reactions* Vol. 49, Chapter 1, pp. 1-330 (John Wiley & Sons, 1997).

