# Supplementary material for: Multicomponent double Mannich alkylamination involving C(sp2)–H and benzylic C(sp3)–H bonds
Source: Nat Commun. 2022 Jan 21;13:435. doi: 10.1038/s41467-022-28088-z (PMC8782832; doi:10.1038/s41467-022-28088-z)
Supplement: Supplementary file 1 — Supplementary Information [file 41467_2022_28088_MOESM1_ESM.pdf]

Supplementary Information for

Multicomponent Double Mannich Alkylamination Involving C(sp^2)–H and Benzylic C(sp^3)–H Bonds

Zhencheng Lai[1,7], Rongkai Wu[2,7], Jiaming Li[1,7], Xing Chen[3], Linwei Zeng[1], Xi Wang[4], Jingjing Guo[5], Zujin Zhao[5], Hironao Sajiki[6], and Sunliang Cui[1,*]

[1] Institute of Drug Discovery and Design, College of Pharmaceutical Sciences, Zhejiang University, 866 Yuhangtang Road, Hangzhou 310058, China

[2] Department of Chemistry, Zhejiang University, 38 Zheda Road, Hangzhou 310027, China

[3] Institute of Molecular Plus, Tianjin University, 92 Weijin Road, Tianjin 300072, China

[4] School of Science, Beijing Jiaotong University, Beijing 100044, China

[5] State Key Laboratory of Luminescent Materials and Devices, South China University of Technology, Guangzhou 510640, China

[6] Laboratory of Organic Chemistry, Gifu-Pharmaceutical University, Gifu 501-1196, Japan

[7] These authors contributed equally

* Corresponding author, E-mail: slcui@zju.edu.cn

Table of Contents

1. Supplementary Methods

General Information……………………………………………………………...S2

Synthesis of Starting Materials……………………………………………………...S3

Multicomponent Double Mannich Alkylamination …………………….…………S15

Reaction Optimization………………………………………….………...S15

Reaction Scope ……………………….……………………………….....S16

Gram-Scale Reaction ……………………………………………..……...S44

Mechanistic Investigation …………………………………………...…...S45

X-Ray Crystallography Information……………………………….………S68

2. Supplementary Figures

NMR Spectra…………………………………………………………....…S70

HPLC Charts..………………………………………………………………S139

3. Supplementary Reference…………………………………..…...…S149

1. Supplementary Methods

General Information

All reactions were carried out under argon atmosphere in oven-dried glassware. The reaction temperatures are reported corresponding to the oil bath temperatures. Reactions were monitored by thin layer chromatography (TLC) using silicycle pre-coated silica gel plates. Column chromatography was performed over silica gel (200–300 mesh).

Melting points were measured with X-4 micro melting point apparatus. Optical rotation were performed on JASCO P-1010. HRMS were performed on Agilent Technologies 6546-LC/Q-TOF LC/MS apparatus (ESI-TOF). ^{1}H NMR spectra and ^{13}C NMR spectra were recorded on a Bruker AV-600 spectrometer, Bruker AV-500 spectrometer or a WNMR-I-400 spectrometer in CD_3Cl or DMSO-d_6 (contain internal TMS). Chemical shifts of ^{1}H NMR spectra were reported in ppm with the internal TMS signal at 0 ppm as a standard, and chemical shifts of ^{13}C NMR spectra were reported in ppm with the chloroform signal at 77.16 ppm as a standard. The data is being reported as (s = singlet, d = doublet, t = triplet, q = quartet, dd = double doublet, dt = double of triplet, m = multiplet or unresolved, br = broad singlet, coupling constant(s) in Hz, integration). DFT calculations were conducted using Gaussian 16 package.

Synthesis of Starting Materials

All starting material of benzofurans were shown in **Supplementary Figure 1**.

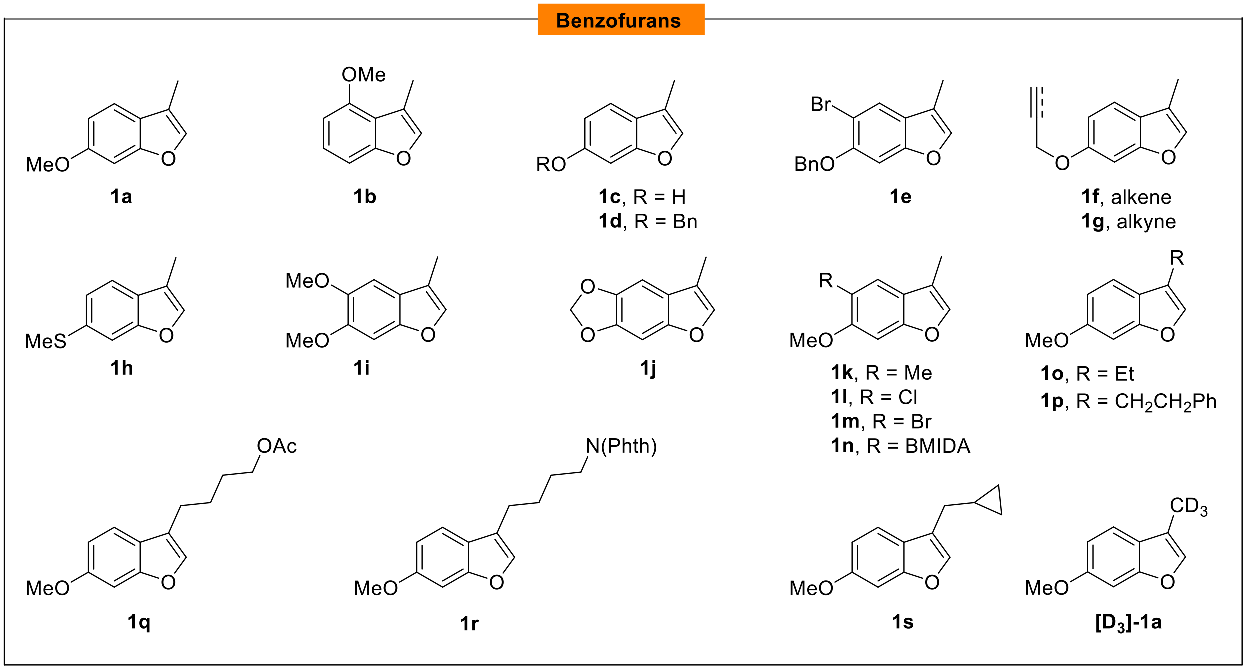

Supplementary Fig. 1. Scope of benzofurans

All starting material of amino acid derivatives were shown in **Supplementary Figure 2**.

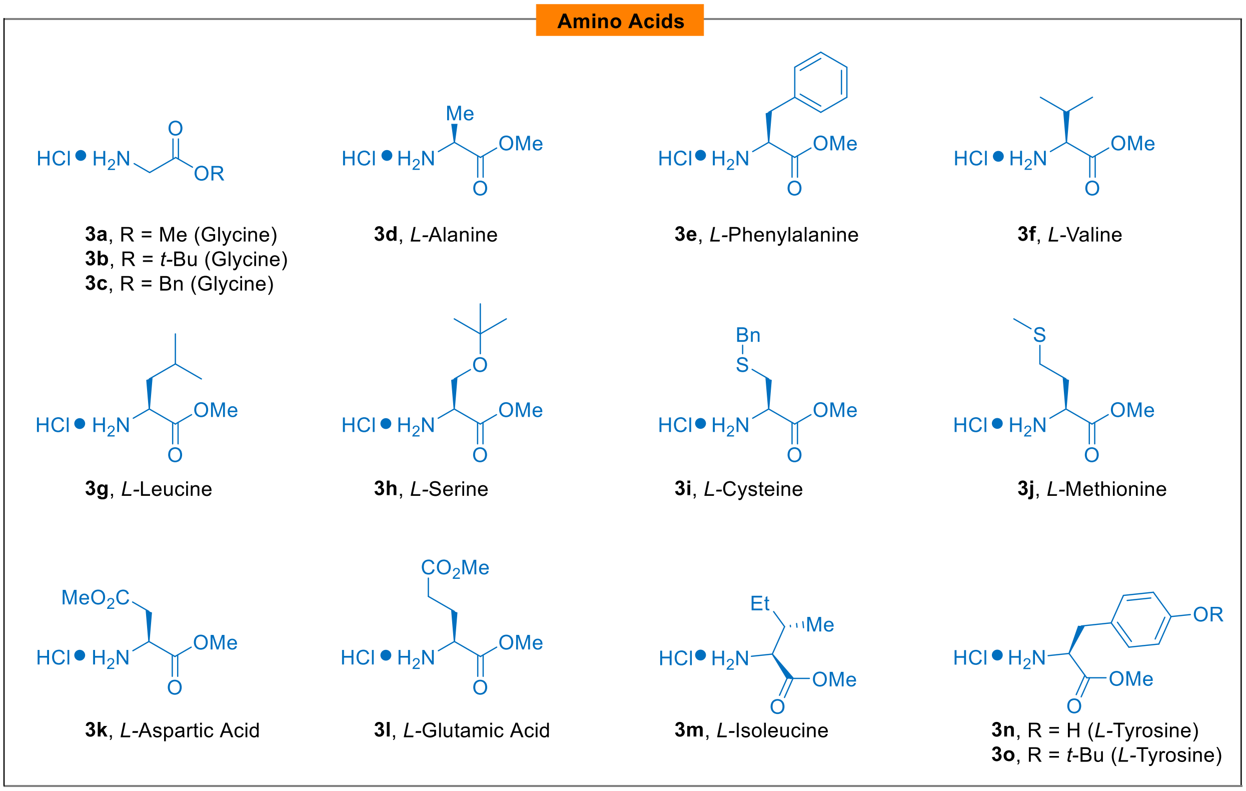

Supplementary Fig. 2. Scope of amino acid derivatives

All starting material of primary amine hydrochlorides were shown in **Supplementary Figure 3**.

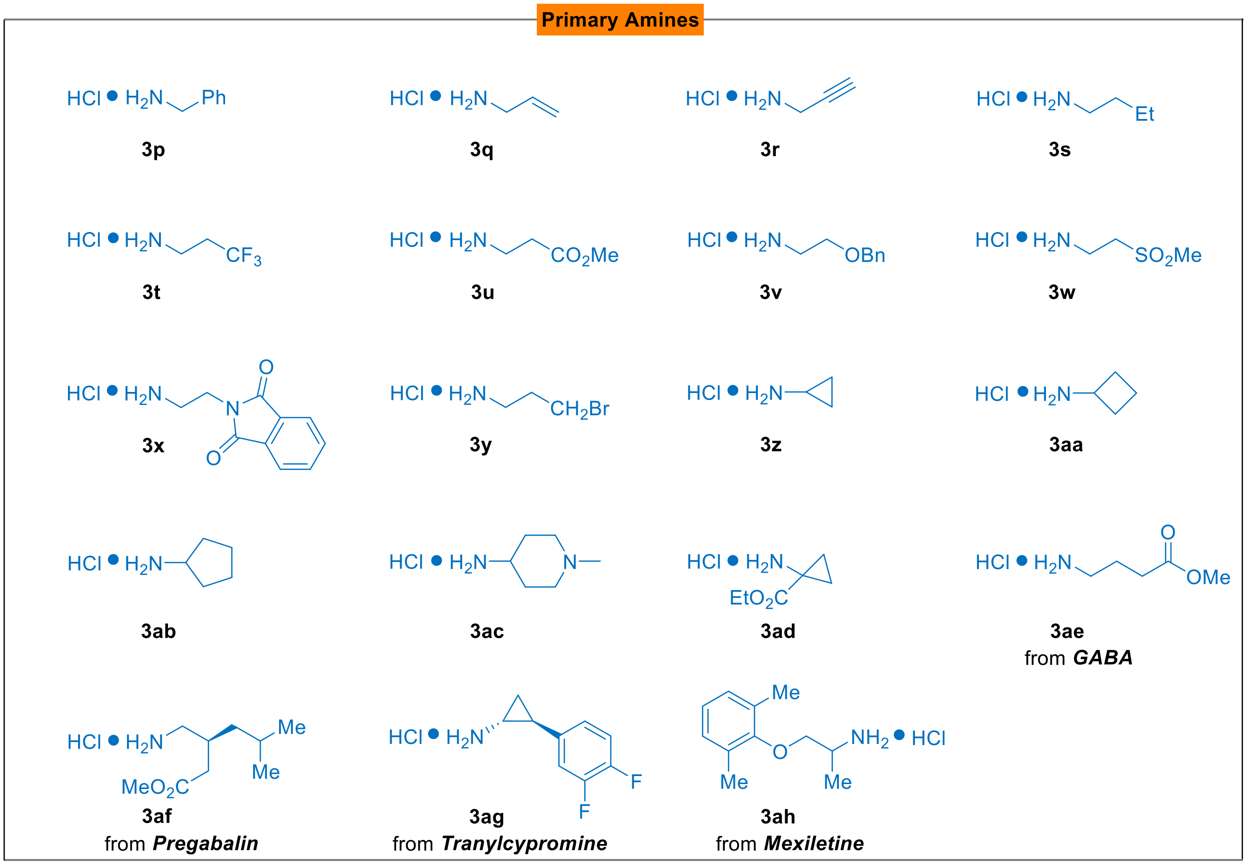

Supplementary Fig. 3. Scope of primary amine hydrochlorides

Reported starting materials

The heteroarenes including **1a** (1), **1b** (2), **1c** (3), **1d** (4), **1i** (5), **1j** (6), **1m** (7), **1o** (8), **1p** (9), were synthesized according to the reported methods. All amino acid methyl ester hydrochlorides and primary amine hydrochlorides were commercially available.

Synthesis of 1e, 1h, 1k and 1l (Method A)

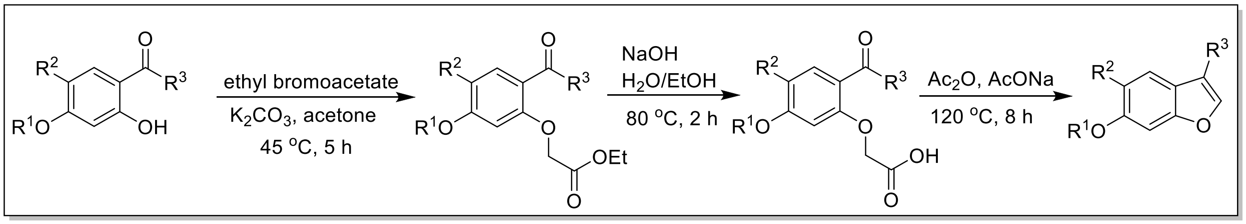

A round-bottom flask equipped with a magnetic stirrer bar was charged with phenol (10 mmol, 1 equiv.), K_2CO_3 (20 mmol, 2 equiv.) and ethyl bromoacetate (15 mmol, 1.5 equiv.). Afterwards, acetone was added and the solution was kept at 45 °C for 5 hours. After cooling to room

temperature, the reaction was filtered through a short pad of Celite. The resulting filtrate was concentrated. The crude product dissolved in EtOH, and saturated aqueous NaOH was added. After stirring at 80 °C for 2 hours, the mixture was cooled and acidified with 1N HCl to pH = 3, the resulting precipitate was collected by filtration. Another round-bottom flask equipped with a magnetic stirrer bar was charged with the acid, AcONa (60 mmol, 6 equiv.) and acetic anhydride (200 mmol, 20 equiv.). The mixture was stirred at 120 °C for 8 hours. After cooling to room temperature, the mixture was quenched by saturated aqueous $NaHCO_3$, and the aqueous layer was extracted with ethyl acetate for three times. The combined organic layer was dried over Na_2SO_4 and concentrated. The crude product was purified by column chromatography on silica gel eluted with petroleum ether/ethyl acetate (v/v, 100:1) to afford the desired benzofuran compound.

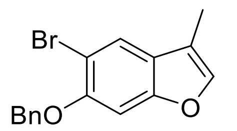

1e

6-(benzyloxy)-5-bromo-3-methylbenzofuran

1e: White solid, m. p. 62.0 – 64.6 °C (1295 mg, 41% yield for three steps in 10 mmol scale).

TLC: Rf = 0.6 (Petroleum ether : EtOAc = 20 : 1).

^{1}H NMR (400 MHz, $CDCl_3$) δ 7.67 (s, 1H), 7.52 – 7.47 (m, 2H), 7.42 – 7.35 (m, 2H), 7.34 – 7.27 (m, 2H), 7.04 (s, 1H), 5.15 (s, 2H), 2.16 (d, *J* = 1.6 Hz, 3H).

^{13}C NMR (125 MHz, $CDCl_3$) δ 155.0, 152.5, 141.4, 136.5, 128.7, 128.0, 127.1, 123.9, 123.2, 115.2, 107.5, 97.8, 71.4, 7.9.

HRMS (ESI-TOF) m/z: $[M+H]^+$ Calcd for $C_{16}H_{14}BrO_2$ 317.0177; Found: 317.0179.

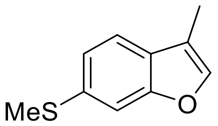

1h

3-methyl-6-(methylthio)benzofuran

1h: Yellow oil, (801 mg, 45% yield for three steps in 10 mmol scale).

TLC: Rf = 0.6 (Petroleum ether : EtOAc = 100 : 1).

^{1}H NMR (400 MHz, $CDCl_3$) δ 7.45 – 7.40 (m, 2H), 7.36 (d, *J* = 1.6 Hz, 1H), 7.24 – 7.19 (m, 1H), 2.54 (s, 3H), 2.23 (d, *J* = 1.2 Hz, 3H).

^{13}C NMR (100 MHz, CDCl$_3$) δ 155.9, 141.3, 134.4, 127.0, 122.5, 119.6, 115.7, 110.1, 17.1, 7.9.

HRMS (ESI-TOF) m/z: $[M+H]^+$ Calcd for $C_{10}H_{11}OS$ 179.0531; Found: 179.0533.

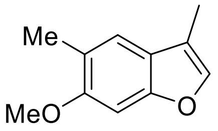

1k

6-methoxy-3,5-dimethylbenzofuran

1k: White solid, m. p. 58.5 – 60.8 °C (810 mg, 46% yield for three steps in 10 mmol scale).

TLC: Rf = 0.6 (Petroleum ether : EtOAc = 20 : 1).

^{1}H NMR (500 MHz, CDCl$_3$) δ 7.33 (s, 1H), 7.27 (s, 1H), 6.98 (s, 1H), 3.89 (s, 3H), 2.35 (s, 3H), 2.24 (s, 3H).

^{13}C NMR (125 MHz, CDCl$_3$) δ 156.1, 154.8, 140.1, 121.9, 121.5, 119.9, 115.3, 93.8, 55.6, 16.7, 7.9.

HRMS (ESI-TOF) m/z: $[M+H]^+$ Calcd for $C_{11}H_{13}O_2$ 177.0916; Found: 177.0914.

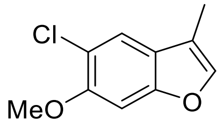

1l

5-chloro-6-methoxy-3-methylbenzofuran

1l: White solid, m. p. 59.5 – 60.8 °C (765 mg, 39% yield for three steps in 10 mmol scale).

TLC: Rf = 0.6 (Petroleum ether : EtOAc = 20 : 1).

^{1}H NMR (500 MHz, CDCl$_3$) δ 7.47 (s, 1H), 7.31 (m, 1H), 7.01 (s, 1H), 3.91 (s, 3H), 2.17 (d, *J* = 1.5 Hz, 3H).

^{13}C NMR (125 MHz, CDCl$_3$) δ 153.4, 151.7, 140.2, 121.6, 118.9, 116.9, 114.2, 94.8, 55.4, 6.8.

HRMS (ESI-TOF) m/z: $[M+H]^+$ Calcd for $C_{10}H_{10}ClO_2$ 197.0369; Found: 197.0368.

Synthesis of 1f and 1g (Method B)

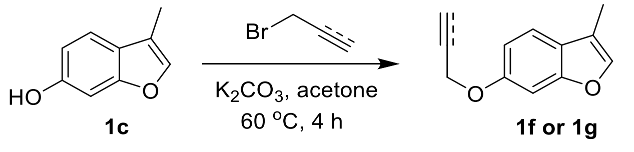

A round-bottom flask equipped with a magnetic stirrer bar was charged with **1c** (1 equiv.), K_2CO_3 (2 equiv.) and allyl bromide or 3-bromopropyne (1.5 equiv.). Afterwards, acetone was

added and the mixture was stirred at 60 °C for 4 hours. After cooling to room temperature, the reaction was filtered through a short pad of Celite. The resulting filtrate was concentrated under reduced pressure to afford the crude product. The crude product was purified by column chromatography on silica gel eluted with petroleum ether/ethyl acetate (v/v, 100:1 to 20:1) to afford the desired benzofuran compound.

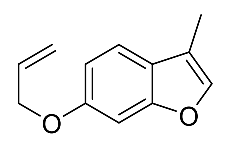

1f

6-(allyloxy)-3-methylbenzofuran

1g: Colorless oil, (837 mg, 89% yield in 5 mmol scale).

TLC: Rf = 0.5 (Petroleum ether : EtOAc = 100 : 1).

^{1}H NMR (500 MHz, $CDCl_3$) δ 7.36 (d, J = 8.5 Hz, 1H), 7.31 (d, J = 1.5 Hz, 1H), 6.99 (d, J = 2.0 Hz, 1H), 6.90 (dd, J_1 = 8.5 Hz, J_2 = 2.2 Hz, 1H), 6.14 – 6.02 (m, 1H), 5.48 – 5.39 (m, 1H), 5.33 – 5.26 (m, 1H), 4.59 – 4.52 (m, 2H), 2.20 (d, J = 1.5 Hz, 3H).

^{13}C NMR (125 MHz, $CDCl_3$) δ 156.9, 156.1, 140.6, 133.3, 122.7, 119.5, 117.8, 115.5, 111.9, 97.1, 69.4, 7.9.

HRMS (ESI-TOF) m/z: $[M+H]^+$ Calcd for $C_{12}H_{13}O_2$ 189.0916; Found: 189.0919.

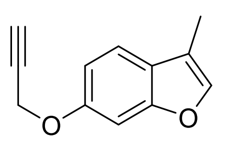

1g

3-methyl-6-(prop-2-yn-1-yloxy)benzofuran

1g: White solid, m. p. 72.2 – 74.8 °C (855 mg, 92% yield in 5 mmol scale).

TLC: Rf = 0.5 (Petroleum ether : EtOAc = 100 : 1).

^{1}H NMR (500 MHz, $CDCl_3$) δ 7.39 (d, J = 8.5 Hz, 1H), 7.33 (d, J = 1.0 Hz, 1H), 7.09 (d, J = 2.5 Hz, 1H), 6.94 (dd, J_1 = 8.5 Hz, J_2 = 2.0 Hz, 1H), 4.72 (d, J = 2.5 Hz, 2H), 2.53 (s, 1H), 2.21 (d, J = 1.5 Hz, 3H).

^{13}C NMR (125 MHz, $CDCl_3$) δ 155.9, 155.8, 140.9, 123.4, 119.6, 115.5, 111.9, 97.6, 78.6, 75.6, 56.5, 7.9.

HRMS (ESI-TOF) m/z: $[M+H]^+$ Calcd for $C_{12}H_{11}O_2$ 187.0759; Found: 187.0762.

Synthesis of the 1n (Method C)

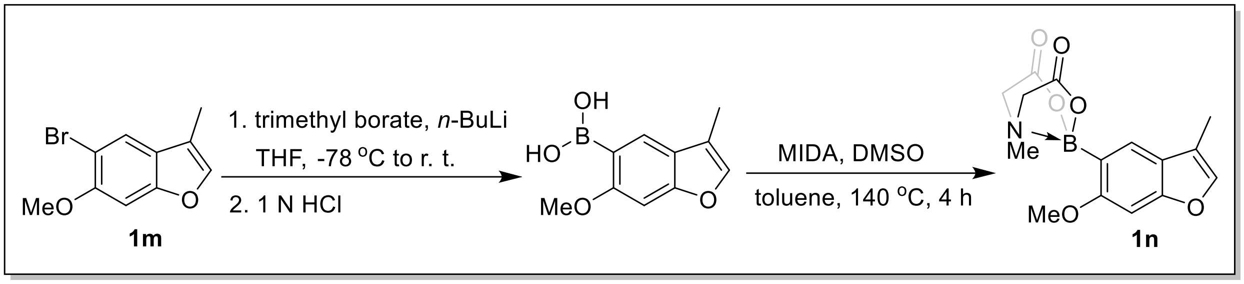

An oven-dried round-bottom flask equipped with a magnetic stirrer bar was charged with **1m** (360 mg, 1.5 mmol, 1 equiv.), then evacuated and purged with argon three times. THF (3 mL) was added and the mixture was stirred at -78 °C for 30 mins. Then *n*-BuLi (0.72 mL, 2.5 N in hexane, 1.2 equiv.) was added dropwise over 20 mins. After stirring for 30 mins, trimethyl borate (312 mg, 3 mmol, 2 equiv.) was added, and the mixture was kept at room temperature overnight. After completion, the reaction was quenched by 1 N HCl solution and the aqueous layer was extracted with ethyl acetate. The combined organic layer was dried over Na_2SO_4 and concentrated. The crude product was used in next step without purification.

Another round-bottom flask equipped with a magnetic stirrer bar was charged with the crude product, MIDA (441 mg. 3 mmol, 2 equiv.), toluene (20 mL) and DMSO (2 ~ 3 drops). After reflux for 4 hours, toluene was removed by vacuum evaporation. The mixture was added with water and extracted with ethyl acetate (three times). the combined organic layer was dried over Na_2SO_4 and concentrated. The crude was purified by flash column chromatography on silica gel with petroleum ether/acetone (v/v, 1:1) to give off-white solid (210mg, 44% for two steps).

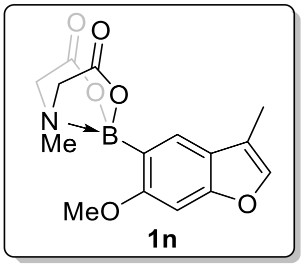

2-(6-methoxy-3-methylbenzofuran-5-yl)-6-methyl-1,3,6,2-dioxazaborocane-4,8-dione

1n: Off-white solid, m. p. 272.3 – 275.6 °C (210 mg, 44% yield for two steps).

TLC: Rf = 0.2 (Petroleum ether/acetone = 1 : 1).

^{1}H NMR (400 MHz, DMSO) δ 7.62 (d, *J* = 6.4 Hz, 2H), 7.15 (s, 1H), 4.38 (d, *J* = 16.8 Hz, 2H), 4.05 (d, *J* = 16.8 Hz, 2H), 3.76 (s, 3H), 2.59 (s, 3H), 2.18 (s, 3H).

^{13}C NMR (100 MHz, DMSO) δ 169.9, 161.1, 157.3, 141.2, 125.0, 121.9, 115.7, 94.7, 63.9, 55.9, 47.9, 8.2.

HRMS (ESI-TOF) m/z: $[M+H]^+$ Calcd for $C_{15}H_{17}BNO_6$ 318.1149; Found: 318.1151.

Synthesis of the 1q (Method D)

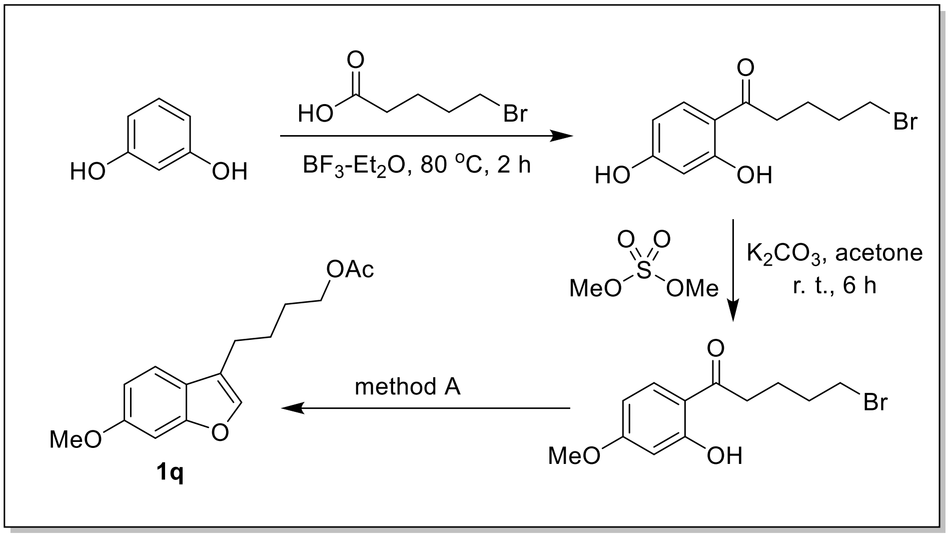

An oven-dried round-bottom flask equipped with a magnetic stirrer bar was charged with resorcinol (2200 mg, 20 mmol, 1 equiv.) and 5-bromovaleric acid (3620 mg, 20 mmol, 1equiv.), then evacuated and purged with argon three times. Boron trifluoride diethyl etherate (20 mL) was added, and the mixture was stirred at 80 ^{o}C for 2 hours. After completion, the reaction was quenched by saturated aqueous $NaHCO_3$. The aqueous layer was extracted with ethyl acetate (three times), and the combined organic layer was dried over Na_2SO_4 and concentrated. The residue was purified by flash column chromatography on silica gel with petroleum ether/ethyl acetate (v/v, 10:1 to 5:1) to give pale yellow solid (4.13 g, 76% yield). Another round-bottom flask equipped with a magnetic stirrer bar was charged with the intermediate (2720 mg, 10 mmol, 1 equiv.), K_2CO_3 (1382 mg. 10 mmol, 1 equiv.) and acetone (20 mL). After stirring at room temperature for 30 minutes, dimethyl sulfate (1260 mg, 10 mmol, 1 equiv.) was added dropwise to the mixture, which was stirred at room temperature for another 6 hours. Afterwards, the reaction was filtered through a short pad of Celite. The resulting filtrate was concentrated under reduced pressure to afford the crude product. Following the general method A, **1q** was synthesized as white solid (940 mg, 36% for four steps).

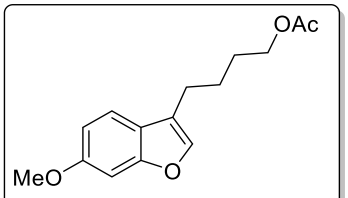

1q

4-(6-methoxybenzofuran-3-yl)butyl acetate

1q: White solid, m. p. 35.5 – 37.6 °C (940 mg, 36% for four steps in 10 mmol scale).

TLC: Rf = 0.5 (Petroleum ether : EtOAc = 20 : 1).

^{1}H NMR (500 MHz, CDCl$_3$) δ 7.37 (d, J = 8.5 Hz, 1H), 7.31 (t, J = 1.0 Hz, 1H), 6.98 (d, J = 2.5 Hz, 1H), 6.86 (dd, J_1 = 8.5 Hz, dd, J_2 = 2.5 Hz, 1H), 4.08 (t, J = 6.5 Hz, 2H), 3.81 (s, 3H), 2.70 – 2.58 (m, 2H), 2.03 (s, 3H), 1.81 – 1.62 (m, 4H).

^{13}C NMR (125 MHz, CDCl$_3$) δ 171.1, 158.0, 156.4, 140.2, 121.5, 119.9, 119.6, 111.3, 96.1, 64.2, 55.6, 28.4, 25.4, 23.2, 20.9.

HRMS (ESI-TOF) m/z: [M+H]$^+$ Calcd for $C_{15}H_{19}O_4$ 263.1283; Found: 263.1286.

Synthesis of 1r (Method E)

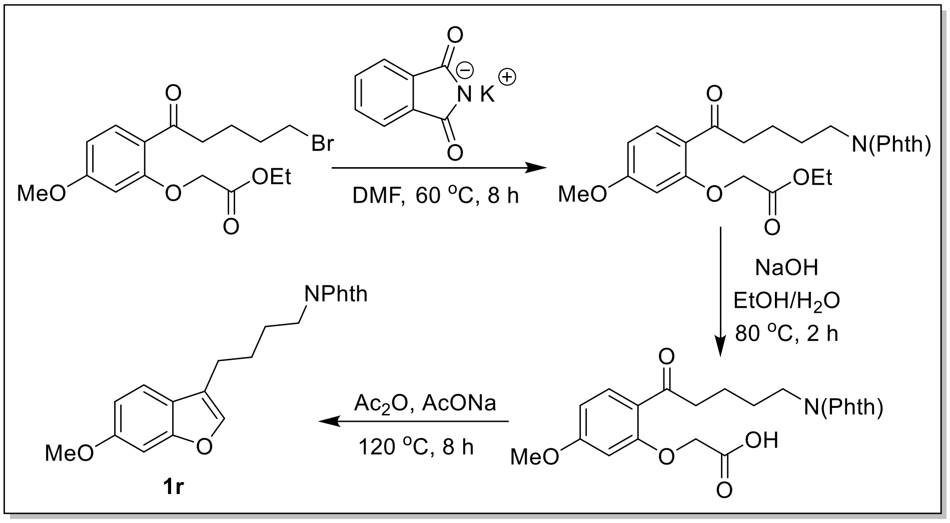

An oven-dried round-bottom flask equipped with a magnetic stirrer bar was charged with ethyl 2-(2-(5-bromopentanoyl)-5-methoxyphenoxy)acetate (3720 mg, 10 mmol, 1 equiv.), potassium phthalimide (1850 mg, 10 mmol, 1 equiv.) and DMF (20 mL). The mixture was stirred at 60 °C for 8 hours. After completion, 40 mL water was added. The aqueous layer was extracted with ethyl acetate (three times), and the combined organic layer was washed with water and dried over Na_2SO_4. The organic layer was concentrated to afford the crude product. Following method A, **1r** was synthesized as white solid (1430 mg, 41% for three steps).

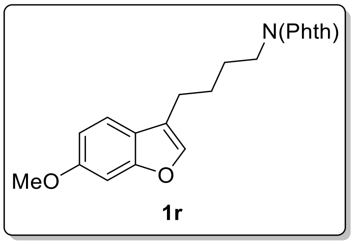

2-(4-(6-methoxybenzofuran-3-yl)butyl)isoindoline-1,3-dione

1r: White solid, m. p. 96.7 – 99.9 ^{o}C (1430 mg, 41% yield for three steps in 10 mmol scale).

TLC: Rf = 0.4 (Petroleum ether : EtOAc = 2 : 1).

^{1}H NMR (500 MHz, CDCl$_3$) δ 7.87 – 7.78 (m, 2H), 7.72 – 7.65 (m, 2H), 7.37 (d, *J* = 8.5 Hz, 1H), 7.31 (s, 1H), 6.96 (s, 1H), 6.83 (dd, J_1 = 8.5 Hz, J_2 = 1.0 Hz, 1H), 3.82 (s, 3H), 3.72 (t, *J* = 6.5 Hz, 2H), 2.67 (t, J = 7.0 Hz, 2H), 1.82 – 1.69 (m, 4H).

^{13}C NMR (125 MHz, CDCl$_3$) δ 168.4, 157.9, 156.3, 140.3, 133.9, 132.1, 123.2, 121.5, 119.8, 119.7, 111.3, 96.0, 55.7, 37.7, 28.3, 26.3, 23.1.

HRMS (ESI-TOF) m/z: $[M+H]^+$ Calcd for $C_{21}H_{20}O_4$ 336.1362; Found: 336.1266.

Synthesis of 1u (Method F)

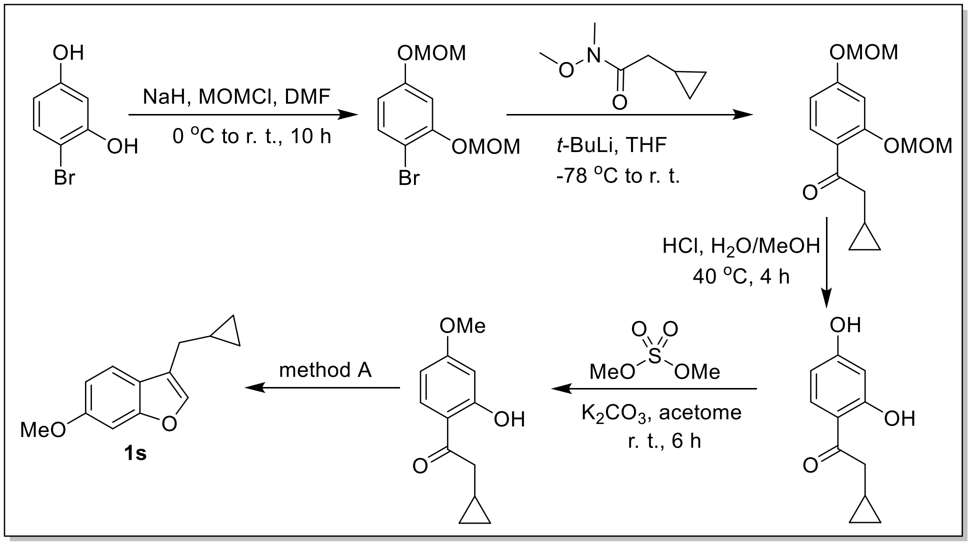

To a suspension of sodium hydride (60% in mineral oil, 1.00 g, 25 mmol) in anhydrous DMF (25 mL) under nitrogen atmosphere at 0 °C was add a DMF (25 mL) solution of 4-bromoresorcinol (1880 mg, 10 mmol, 1 equiv.). Then, methoxymethyl chloride (2025 mg, 25 mmol, 2.5 equiv.) was added dropwise to this suspension. The reaction was warmed to ambient temperature and kept for one hour. Then water was added to quench the reaction and extracted with diethyl ether. The organic layer was washed with 1N sodium hydroxide and brine, dried over Na_2SO_4 and

concentrated. The residue was purified by flash column chromatography on silica gel with petroleum ether/ethyl acetate (v/v, 20:1) to give 1-bromo-2,4-bis(methoxymethoxy)benzene as colorless oil (1821 mg, 66%).

To a THF (30 mL, anhydrous) solution of 1-bromo-2,4-bis(methoxymethoxy)benzene (1380 mg, 5 mmol, 1 equiv.) at -78 °C was added with 1.3M *tert*-butyllithium in pentane (7.7 mL, 10 mmol, 2 equiv.) over 10 minutes. The reaction was kept for 15 minutes, then 2-cyclopropyl-*N*-methoxy-*N*-methylacetamide (715 mg, 5 mmol, 1 equiv.) was added to the reaction. The reaction was kept at room temperature for 1 hour, treated with 1N aqueous hydrochloric acid (20 mL) and kept for another one hour. Then the organic layer was washed with 1N aqueous hydrochloric acid (20 mL) and brine, dried over Na_2SO_4. The solution was filtered and evaporated. The residue was dissolved in MeOH (10 L) and treated with 1N aqueous hydrochloric acid (10 mL) and kept at 40 °C for 4 hours. The mixture was poured into water (20 mL), and the aqueous layer was extracted with ethyl acetate. The organic layers was washed with brine, dried over Na_2SO_4, filtered and evaporated in vacuum. The residue was purified by flash column chromatography on silica gel with petroleum ether/ethyl acetate (v/v, 5:1 to 1:1) to give 2-cyclopropyl-1-(2,4-dihydroxyphenyl)ethan-1-one as white solid (499 mg, 52%).

A round-bottom flask equipped with a magnetic stirrer bar was charged with the 2-cyclopropyl-1-(2,4-dihydroxyphenyl)ethan-1-one (384 mg, 2 mmol, 1 equiv.), K_2CO_3 (276 mg. 2 mmol, 1 equiv.) and acetone (5 mL). After stirring at room temperature for 30 minutes, dimethyl sulfate (252 mg, 2 mmol, 1 equiv.) was added dropwise to the mixture and kept at room temperature for another 6 hours. Afterwards, the reaction was filtered through a short pad of Celite. The resulting filtrate was concentrated to afford the crude product. Following the general method A, **1u** was synthesized as white solid (218 mg, 54% for four steps).

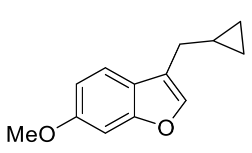

1s

3-(cyclopropylmethyl)-6-methoxybenzofuran

1s: Colorless oil, (218 mg, 54% yield for four steps in 2 mmol scale).

TLC: Rf = 0.4 (Petroleum ether).

^{1}H NMR (500 MHz, CDCl$_3$) δ 7.51 – 7.39 (m, 2H), 7.02 (d, J = 2.0 Hz, 1H), 6.89 (dd, J_1 = 8.5 Hz, J_2 = 2.5 Hz, 1H), 3.86 (s, 3H), 2.56 (dd, J_1 = 7.0 Hz, J_2 = 1.5 Hz, 2H), 1.13 – 1.02 (m, 1H), 0.63 – 0.53 (m, 2H), 0.30 – 0.20 (m, 2H).

^{13}C NMR (125 MHz, CDCl$_3$) δ 157.9, 156.4, 140.3, 121.9, 120.3, 119.8, 111.3, 96.0, 55.7, 28.6, 10.3, 4.9.

HRMS (ESI-TOF) m/z: $[M+H]^+$ Calcd for $C_{13}H_{15}O_2$ 203.1072; Found: 203.1076.

Synthesis of [D$_3$]-1a

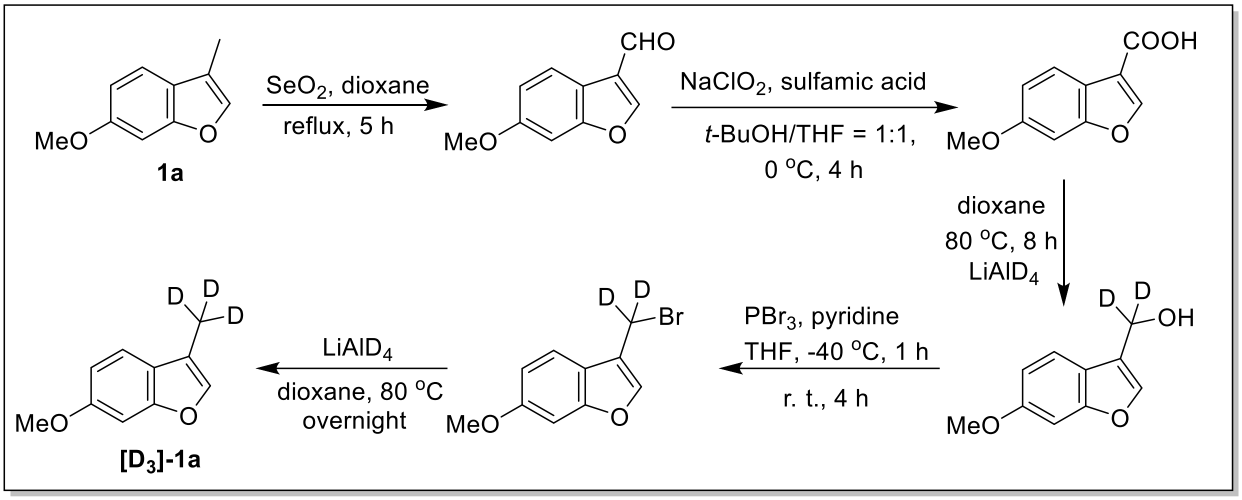

A round-bottom flask equipped with a magnetic stirrer bar was charged with **1a** (1.31 g, 8 mmol, 1 equiv.) and SeO_2 (1.33 g, 12 mmol, 1.5 equiv.). 1,4-Dioxane (20 mL) was added and the mixture was reflux for 5 hours. After completion, the reaction was concentrated. The crude product was purified by flash column chromatography on silica gel with petroleum ether to give 6-methoxybenzofuran-3-carbaldehyde as pale yellow solid (840 mg, 60%).

To a solution (*t*-BuOH and THF, 20 mL, 1:1) of 6-methoxybenzofuran-3-carbaldehyde (528 mg, 3 mmol, 1equiv.) and sulfamic acid (873 mg, 9 mmol, 3 equiv.) at -5 ^oC was added aqueous sodium chlorite solution (80% in 850 mg, 7.5 mmol, 2.5 equiv., in 5 mL water). The resulting mixture was kept at -5 ^oC for 4 hours. The solution was diluted with water and extracted with ethyl acetate. The combined organic layer was dried over Na_2SO_4 and concentrated. The residue was purified by flash column chromatography on silica gel with petroleum ether/ethyl acetate (v/v, 4:1 to 2:1) to give 6-methoxybenzofuran-3-carboxylic acid as yellow solid (415 mg, 72%).

A Schlenk tube equipped with a magnetic stirrer bar was charged with the acid (394 mg, 2 mmol, 1 equiv.) and $LiAlD_4$ (deuterated rate ≥ 98%, 168 mg, 4 mmol, 2 equiv.), evacuated and purged with argon. 1,4-Dioxane (3 mL) was added and the reaction was stirred at 80 ^oC for 8 hours.

Then the reaction was quenched by water. The aqueous layer was extracted with ethyl acetate and the combined organic layer was dried over Na_2SO_4 and concentrated. The residue was purified by flash column chromatography on silica gel with petroleum ether/ethyl acetate (v/v, 4:1)to give (6-methoxybenzofuran-3-yl) methan-*d₂*-ol as off-white solid (257 mg, 71%).

Afterwards, a Schlenk tube equipped with a magnetic stirrer bar was charged with (6-methoxybenzofuran-3-yl) methan-d_2-ol (240 mg, 1.33 mmol, 1 equiv.), evacuated and purged with argon. Anhydrous THF (4 mL) and pyridine (10 μL, 0.13 mmol, 0.1 equiv.) were added and stirred at -40 ^{o}C for 15 minutes. Then a THF solution of phosphorus tribromide (66 μL, 0.66 mmol, 0.5 equiv. in 1.5 mL THF) was added slowly. The reaction was stirred at room temperature for 3 hours and then neutralized by saturated aqueous $NaHCO_3$ solution. The aqueous layer was extracted with ethyl acetate and the combined organic layer was dried over Na_2SO_4 and concentrated. The crude product was used in next step without purification.

A Schlenk tube equipped with a magnetic stirrer bar was charged with the crude and $LiAlD_4$ (deuterated rate ≥ 98%, 126 mg, 3 mmol, 2.25 equiv.), evacuated and purged with argon for three times. 1,4-Dioxane (3 mL) was added and the solution was kept at 80 ^{o}C overnight and then quenched with water. The aqueous layer was extracted with ethyl acetate and the combined organic layer was dried over Na_2SO_4 and concentrated. The residue was purified by flash column chromatography on silica gel with petroleum ether to give 6-methoxy-3-(methyl-d_3)benzofuran as white solid (150 mg, 68% for two steps).

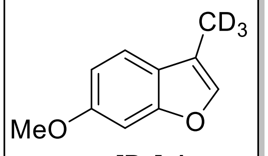

[D₃]-1a

6-methoxy-3-(methyl-*d₃*)benzofuran

[D₃]-1a: White solid, m. p. 44.9 – 48.2 ^{o}C (150 mg, 68% for two steps in 3 mmol scale).

TLC: Rf = 0.7 (Petroleum ether : acetone = 100 : 1).

^{1}H NMR (500 MHz, $CDCl_3$) δ 7.36 (d, *J* = 8.5 Hz, 1H), 7.30 (s, 1H), 7.04 – 6.93 (m, 1H), 6.93 – 6.83 (m, 1H), 3.82 (s, 3H).

^{13}C NMR (125 MHz, $CDCl_3$) δ 158.0, 156.3, 140.5, 122.5, 119.5, 115.4, 111.3, 95.9, 55.7, 7.3 (m).

HRMS (ESI-TOF) m/z: $[M+H]^+$ Calcd for $C_{10}H_8D_3O_2$ 166.0947; Found: 166.0951.

Multicomponent Double Mannich Alkylamination

Reaction Optimization

The optimizations were conducted with 0.2 mmol scale and yield refers to isolated product by column chromatography on silica gel eluted with petroleum ether/ethyl acetate (v/v, 5:1).

Supplementary Table 1. Reaction Optimization.

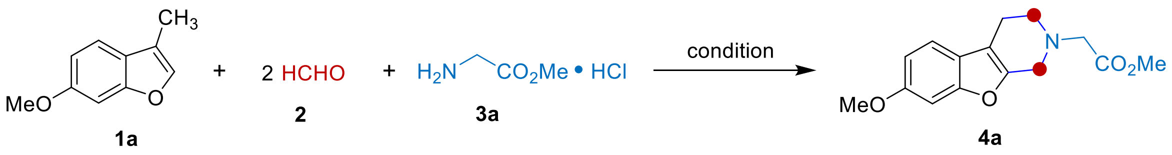

Entry	2 (equiv.)	3a (equiv.)	Solvent	T	Yield[b]
1	2.5	1	AcOH (0.5 mL)	60 °C	45%
2	2.5	2	AcOH (0.5 mL)	60 °C	40%
3	4	1	AcOH (0.5 mL)	60 °C	63%
4	4	2	AcOH (0.5 mL)	60 °C	72%
5	4	2	AcOH (1 mL)	60 °C	74%
6	4	2	AcOH (2 mL)	60 °C	62%
7	4	2	AcOH (0.5 mL)	80 °C	60%
8	4	2	AcOH (0.5 mL)	100 °C	trace
9	4	2	AcOH (0.5 mL)	r. t.	25%
10	4	2	CH_3CN (1 mL)	60 °C	72%
11	4	2	*i*-PrOH	60 °C	20%
12	4	2	HFIP (0.5 mL)	50 °C	33%
13	4	2	HFIP/AcOH (1 mL, 1:1)	60 °C	51%
14[c]	4	2	AcOH (1 mL)	60 °C	65%
15[d]	4	2	AcOH (20 mL)	60 °C	78%

[a] Reaction conditions: **1a** (0.2 mmol), **2**, **3a**, solvent, 3 hours.

[b] Isolated yields.

[c] Methyl glycinate was used as amine source. HFIP = $(CF_3)_2CHOH$.

[d] 5 mmol scale.

Reaction Scope

Procedure for the synthesis of 4

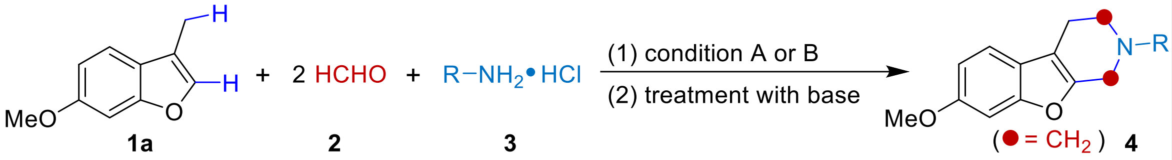

Condition A (for 3a-3o)

An oven-dried Schlenk tube equipped with a magnetic stirrer bar was charged with **1** (0.2 mmol, 1 equiv.), **2** (0.8 mmol, 4 equiv.) and **3** (0.4 mmol, 2 equiv.), evacuated and purged with argon for three times. Then 1 mL AcOH was added by a syringe, and the reaction was stirred at 60 ^{o}C for 3 hours to be completed. After completion, the reaction was quenched by saturated aqueous $NaHCO_3$. The aqueous layer was extracted with ethyl acetate (three times), and the combined organic layer was dried over Na_2SO_4 and concentrated. Purification by flash column chromatography afforded the pure product.

Condition B (for 3p-3ah)

An oven-dried Schlenk tube equipped with a magnetic stirrer bar was charged with **1** (0.2 mmol, 1 equiv.), **2** (1.6 mmol, 8 equiv.) and **3** (0.8 mmol, 4 equiv.), evacuated and purged with argon for three times. Then 1 mL AcOH was added by a syringe, and the reaction was stirred at 90 ^{o}C for 30 minutes to be completed. After completion, the reaction was quenched by saturated aqueous $NaHCO_3$. The aqueous layer was extracted with ethyl acetate (three times), and the combined organic layer was dried over Na_2SO_4 and concentrated. Purification by flash column chromatography afforded the pure product.

Characterization Data

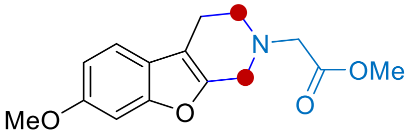

4a

methyl 2-(7-methoxy-3,4-dihydrobenzofuro[2,3-*c*]pyridin-2(1*H*)-yl)acetate

4a: Pale yellow solid, m. p. 69.2 – 70.4 ^{o}C (41 mg, 74% yield; 1079 mg, 78% yield in 5 mmol scale).

TLC: Rf = 0.3 (Petroleum ether : EtOAc = 4 : 1).

^{1}H NMR (500 MHz, $CDCl_3$) δ 7.28 (d, *J* = 8.5 Hz, 1H), 6.97 (d, *J* = 2.5 Hz, 1H), 6.83 (dd, J_1 = 8.5 Hz, J_2 =2.5 Hz, 1H), 3.84 (m, 5H), 3.75 (s, 3H), 3.51 (s, 2H), 2.98 (t, *J* = 6.0 Hz, 2H), 2.73 (t, *J* = 6.0 Hz, 2H).

^{13}C NMR (100 MHz, $CDCl_3$) δ 170.9, 157.4, 155.6, 149.5, 121.5, 118.6, 110.9, 110.8, 96.4, 57.8, 55.7, 51.8, 50.0, 49.6, 20.6.

HRMS (ESI-TOF) m/z: $[M+H]^+$ Calcd for $C_{15}H_{18}NO_4$ 276.1236; Found: 276.1239.

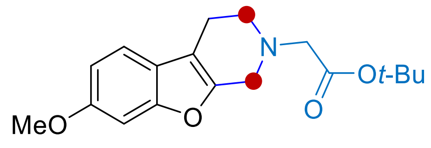

4b

***tert*-butyl 2-(7-methoxy-3,4-dihydrobenzofuro[2,3-*c*]pyridin-2(1*H*)-yl)acetate**

4b: Pale yellow solid, m. p. 88.7 – 90.9 °C (35 mg, 56% yield).

TLC: Rf = 0.3 (Petroleum ether : EtOAc = 4 : 1).

^{1}H NMR (600 MHz, $CDCl_3$) δ 7.28 (d, *J* = 8.4 Hz, 1H), 6.97 (d, *J* = 2.4 Hz, 1H), 6.83 (dd, J_1 = 8.4 Hz, J_2 = 2.4 Hz, 1H), 3.86 – 3.82 (m, 5H), 3.40 (s, 2H), 2.99 (t, *J* = 5.4 Hz, 2H), 2.75 – 2.69 (m, 2H), 1.49 (s, 9H).

^{13}C NMR (150 MHz, $CDCl_3$) δ 169.8, 157.3, 155.6, 149.8, 121.6, 118.6, 110.9, 110.8, 96.4, 81.3, 58.6, 55.8, 49.9, 49.5, 28.2, 20.8.

HRMS (ESI-TOF) m/z: $[M+H]^+$ Calcd for $C_{18}H_{24}NO_4$ 318.1705; Found: 318.1704.

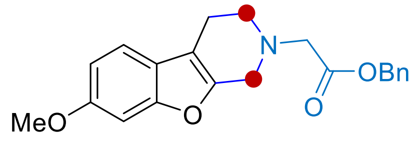

4c

benzyl 2-(7-methoxy-3,4-dihydrobenzofuro[2,3-*c*]pyridin-2(1*H*)-yl)acetate

4c: Pale yellow solid, m. p. 89.1 – 93.4 °C (47 mg, 67% yield).

TLC: Rf = 0.4 (Petroleum ether : EtOAc = 4 : 1).

^{1}H NMR (400 MHz, $CDCl_3$) δ 7.39 – 7.32 (m, 5H), 7.28 (d, *J* = 8.4 Hz, 1H), 6.98 (d, *J* = 2.0 Hz, 1H), 6.84 (dd, J_1 = 8.4 Hz, J_2 = 2.0 Hz, 1H), 5.20 (s, 2H), 3.86 (t, *J* = 2.0 Hz, 2H), 3.84 (s, 3H), 3.56 (s, 2H), 3.00 (t, *J* = 5.6 Hz, 2H), 2.76 – 2.69 (m, 2H).

^{13}C NMR (100 MHz, CDCl$_3$) δ 170.4, 157.4, 155.6, 149.6, 135.6, 128.7, 128.5, 121.5, 118.6, 110.9, 110.9, 96.4, 66.6, 57.9, 55.8, 50.0, 49.6, 20.7.

HRMS (ESI-TOF) m/z: $[M+H]^+$ Calcd for $C_{21}H_{22}NO_4$ 352.1549; Found: 352.1549.

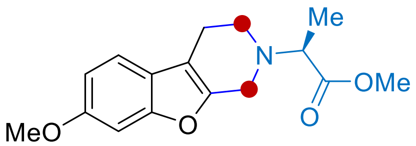

4d

methyl (*S*)-2-(7-methoxy-3,4-dihydrobenzofuro[2,3-*c*]pyridin-2(1*H*)-yl)propanoate

4d: Orange solid, m. p. 79.6 – 82.4 °C (38 mg, 66% yield), $[\alpha]_D{}^{20}$ = -10.4 (c = 3.0, CHCl$_3$).

TLC: Rf = 0.5 (Petroleum ether : EtOAc = 10 : 1).

HPLC: The *ee* value was determined by HPLC analysis on a CHIRALPAK IC-3 column (hexane/isopropanol = 95/5, flow = 1.0 mL/min, 254 nm) with t_r = 19.3 min (major), 20.9 min (minor): > 99% *ee*.

^{1}H NMR (600 MHz, CDCl$_3$) δ 7.27 (d, *J* = 8.4 Hz, 1H), 6.97 (d, *J* = 2.4 Hz, 1H), 6.82 (dd, J_1 = 8.4 Hz, J_2 = 2.4 Hz, 1H), 3.85 (s, 2H), 3.83 (s, 3H), 3.73 (s, 3H), 3.61 (q, *J* = 7.2 Hz, 1H), 3.06 – 2.99 (m, 1H), 2.92 – 2.86 (m, 1H), 2.73 – 2.63 (m, 2H), 1.43 (d, *J* = 7.2 Hz, 3H).

^{13}C NMR (150 MHz, CDCl$_3$) δ 173.5, 157.3, 155.7, 150.1, 121.6, 118.6, 111.1, 110.8, 96.4, 61.8, 55.7, 51.6, 47.2, 46.4, 21.5, 15.2.

HRMS (ESI-TOF) m/z: $[M+H]^+$ Calcd for $C_{16}H_{20}NO_4$ 290.1392; Found: 290.1396.

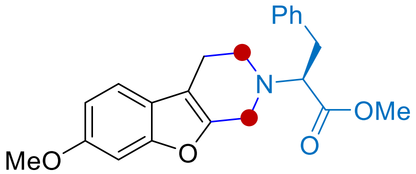

4e

methyl (*S*)-2-(7-methoxy-3,4-dihydrobenzofuro[2,3-*c*]pyridin-2(1*H*)-yl)-3-phenylpropanoate

4e: Pale yellow solid, m. p. 62.9 – 63.5 °C (51 mg, 70% yield), $[\alpha]_D{}^{20}$ = -31.0 (c = 0.9, CHCl$_3$).

TLC: Rf = 0.4 (Petroleum ether : EtOAc = 10 : 1).

HPLC: The *ee* value was determined by HPLC analysis on a CHIRALPAK IC-3 column (hexane/isopropanol = 95/5, flow = 1.0 mL/min, 254 nm) with t_r = 12.8 min (minor), 14.6 min (major): > 99% *ee*.

^{1}H NMR (600 MHz, $CDCl_3$) δ 7.33 – 7.18 (m, 6H), 6.98 (d, *J* = 2.4 Hz, 1H), 6.84 (dd, J_1 = 8.4 Hz, J_2 = 1.8 Hz, 1H), 3.97 – 3.86 (m, 2H), 3.84 (s, 3H), 3.76 – 3.70 (m, 1H), 3.63 (s, 3H), 3.24 – 3.17 (m, 1H), 3.13 – 3.05 (m, 2H), 2.94 – 2.88 (m, 1H), 2.74 – 2.62 (m, 2H).

^{13}C NMR (100 MHz, $CDCl_3$) δ 171.9, 157.3, 155.7, 150.1, 137.9, 129.2, 128.5, 126.6, 121.6, 118.6, 111.2, 110.8, 96.4, 68.9, 55.8, 51.4, 47.4, 46.9, 36.1, 21.7.

HRMS (ESI-TOF) m/z: $[M+H]^+$ Calcd for $C_{22}H_{24}NO_4$ 366.1705; Found: 366.1708.

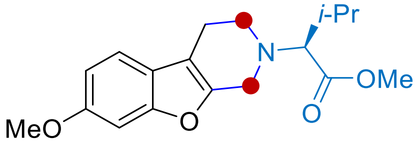

4f

methyl (*S*)-2-(7-methoxy-3,4-dihydrobenzofuro[2,3-*c*]pyridin-2(1*H*)-yl)-3-methylbutanoate

4f: Pale yellow solid, m. p. 77.6 – 79.1 °C (40 mg, 63% yield), $[\alpha]_D^{20}$ = -25.8 (c = 1.5, $CHCl_3$).

TLC: Rf = 0.5 (Petroleum ether : EtOAc = 10 : 1).

HPLC: The *ee* value was determined by HPLC analysis on a CHIRALPAK IC-3 column (hexane/isopropanol = 95/5, flow = 1.0 mL/min, 254 nm) with t_r = 5.4 min (minor), 6.7 min (major): 95% *ee*.

^{1}H NMR (400 MHz, $CDCl_3$) δ 7.27 (d, *J* = 8.4 Hz, 1H), 6.97 (d, *J* = 2.4 Hz, 1H), 6.83 (dd, J_1 = 8.4 Hz, J_2 = 2.0 Hz, 1H), 3.84 (s, 3H), 3.80 – 3.76 (m, 2H), 3.71 (s, 3H), 3.05 – 2.96 (m, 2H), 2.81 – 2.70 (m, 1H), 2.71 – 2.60 (m, 2H), 2.25 – 2.14 (m, 1H), 1.02 (d, *J* = 6.8 Hz, 3H), 0.93 (d, *J* = 6.8 Hz, 3H).

^{13}C NMR (100 MHz, $CDCl_3$) δ 172.3, 157.2, 155.6, 150.6, 121.7, 118.6, 111.2, 110.8, 96.4, 73.9, 55.8, 50.9, 46.9, 46.8, 27.3, 21.7, 19.9, 19.4.

HRMS (ESI-TOF) m/z: $[M+H]^+$ Calcd for $C_{18}H_{24}NO_4$ 318.1705; Found: 318.1708.

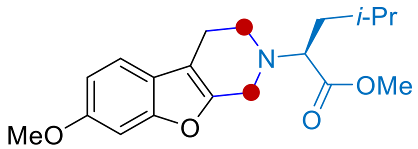

4g

methyl (*S*)-2-(7-methoxy-3,4-dihydrobenzofuro[2,3-*c*]pyridin-2(1*H*)-yl)-4-methylpentanoate

4g: Pale yellow solid, m. p. 79.6 – 81.0 °C (40 mg, 61% yield), $[\alpha]_D^{20}$ = -18.8 (c = 2.5, $CHCl_3$).

TLC: Rf = 0.3 (Petroleum ether : EtOAc = 20 : 1).

HPLC: The *ee* value was determined by HPLC analysis on a CHIRALPAK IC-3 column (hexane/isopropanol = 95/5, flow = 1.0 mL/min, 254 nm) with t_r = 6.6 min (minor), 8.3 min (major): >99% *ee*.

^{1}H NMR (400 MHz, CDCl$_3$) δ 7.30 (d, *J* = 8.4 Hz, 1H), 7.01 (d, *J* = 2.4 Hz, 1H), 6.86 (dd, J_1 = 8.4 Hz, J_2 = 2.4 Hz, 1H), 3.88 (t, J = 2.0 Hz, 2H), 3.87 (s, 3H), 3.75 (s, 3H), 3.62 – 3.55 (m, 1H), 3.12 – 3.03 (m, 1H), 2.94 – 2.86 (m, 1H), 2.77 – 2.60 (m, 2H), 1.79 – 1.69 (m, 3H), 1.01 – 0.94 (m, 6H).

^{13}C NMR (100 MHz, CDCl$_3$) δ 173.2, 157.2, 155.6, 150.5, 121.7, 118.6, 111.2, 110.8, 96.4, 64.9, 55.8, 51.3, 47.1, 46.5, 38.5, 25.0, 22.7, 22.5, 21.8.

HRMS (ESI-TOF) m/z: [M+H]$^+$ Calcd for $C_{19}H_{26}NO_4$ 332.1862; Found: 332.1871.

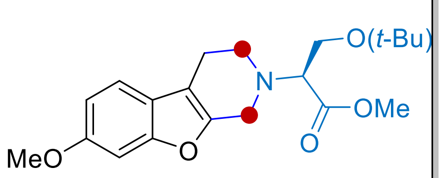

4h

methyl (*S*)-3-(*tert*-butoxy)-2-(7-methoxy-3,4-dihydrobenzofuro[2,3-*c*]pyridin-2(1*H*)-yl)propanoate

4h: Pale yellow solid, m. p. 57.7 – 60.1 °C (54 mg, 75% yield), $[\alpha]_D^{20}$ = -3.4 (c = 2.6, CHCl$_3$).

TLC: Rf = 0.3 (Petroleum ether : EtOAc = 5 : 1).

HPLC: The *ee* value was determined by HPLC analysis on a CHIRALPAK IE-3 column (hexane/isopropanol = 75/25, flow = 0.6 mL/min, 254 nm) with t_r = 6.9 min (major), 7.4 min (minor): >99% *ee*.

^{1}H NMR (400 MHz, CDCl$_3$) δ 7.27 (d, *J* = 8.4 Hz, 1H), 6.97 (d, *J* = 2.0 Hz, 1H), 6.82 (dd, J_1 = 8.4 Hz, J_2 = 2.0 Hz, 1H), 4.02 – 3.85 (m, 2H), 3.87 – 3.79 (m, 4H), 3.77 – 3.69 (m, 4H), 3.70 – 3.63 (m, 1H), 3.10 – 2.92 (m, 2H), 2.73 – 2.63 (m, 2H), 1.19 (s, 9H).

^{13}C NMR (100 MHz, CDCl$_3$) δ 171.7, 157.4, 155.7, 150.3, 121.8, 118.7, 111.1, 110.8, 96.5, 73.6, 67.4, 61.2, 55.9, 51.6, 48.1, 47.5, 27.5, 21.6.

HRMS (ESI-TOF) m/z: [M+H]$^+$ Calcd for $C_{20}H_{28}NO_5$ 362.1967; Found: 362.1975.

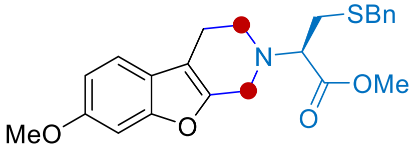

4i

methyl (*R*)-3-(benzylthio)-2-(7-methoxy-3,4-dihydrobenzofuro[2,3-*c*]pyridin-2(1*H*)-yl) propanoate

4i: Pale yellow solid, m. p. 120.4 – 121.8 °C (54 mg, 66% yield), $[\alpha]_D^{20}$ = -10.3 (c = 3.2, $CHCl_3$).

TLC: Rf = 0.3 (Petroleum ether : EtOAc = 10 : 1).

^{1}H NMR (400 MHz, $CDCl_3$) δ 7.34 – 7.22 (m, 6H), 6.96 (d, *J* = 2.0 Hz, 1H), 6.82 (dd, J_1 = 8.4 Hz, J_2 = 2.0 Hz, 1H), 3.87 – 3.78 (m, 4H), 3.79 – 3.70 (m, 6H), 3.60 – 3.52 (m, 1H), 3.01 – 2.88 (m, 2H), 2.84 – 2.75 (m, 2H), 2.69 – 2.60 (m, 2H).

^{13}C NMR (100 MHz, $CDCl_3$) δ 171.2, 157.3, 155.6, 149.9, 138.1, 128.9, 128.6, 127.2, 121.6, 118.6, 111.1, 110.8, 96.4, 66.9, 55.8, 51.6, 47.3, 46.7, 36.9, 30.8, 21.5.

HRMS (ESI-TOF) m/z: $[M+H]^+$ Calcd for $C_{23}H_{26}NO_4S$ 412.1583; Found: 412.1580.

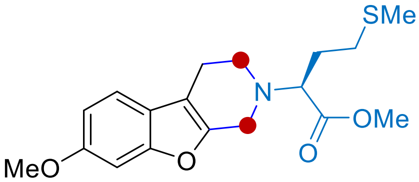

4j

methyl (*S*)-2-(7-methoxy-3,4-dihydrobenzofuro[2,3-*c*]pyridin-2(1*H*)-yl)-4-(methylthio) butanoate

4j: Pale yellow solid, m. p. 58.2 – 59.6 °C (38 mg, 54% yield), $[\alpha]_D^{20}$ = -36.0 (c = 2.4, $CHCl_3$).

TLC: Rf = 0.4 (Petroleum ether : EtOAc = 10 : 1).

HPLC: The *ee* value was determined by HPLC analysis on a CHIRALPAK IC-3 column (hexane/isopropanol = 95/5, flow = 1.0 mL/min, 254 nm) with t_r = 14.6 min (minor), 16.6 min (major): 95% *ee*.

^{1}H NMR (600 MHz, $CDCl_3$) δ 7.27 (d, *J* = 8.4 Hz, 1H), 6.97 (d, *J* = 2.4 Hz, 1H), 6.83 (dd, J_1 = 8.4 Hz, J_2 = 2.4 Hz, 1H), 3.93 – 3.78 (m, 5H), 3.72 (s, 3H), 3.70 – 3.65 (m, 1H), 3.09 – 3.03 (m, 1H), 2.89 – 2.82 (m, 1H), 2.72 – 2.56 (m, 4H), 2.15 – 2.03 (m, 5H).

^{13}C NMR (150 MHz, CDCl$_3$) δ 172.5, 157.3, 155.6, 150.3, 121.6, 118.5, 111.1, 110.8, 96.4, 65.1, 55.8, 51.4, 46.9, 46.6, 30.8, 28.8, 21.7, 15.5.

HRMS (ESI-TOF) m/z: $[M+H]^+$ Calcd for $C_{18}H_{24}NO_4S$ 350.1426; Found: 350.1426.

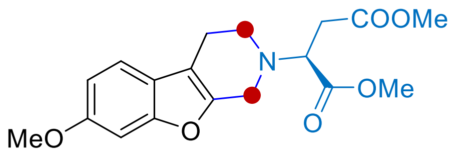

4k

dimethyl (*S*)-2-(7-methoxy-3,4-dihydrobenzofuro[2,3-*c*]pyridin-2(1*H*)-yl)succinate

4k: Pale yellow solid, m. p. 88.3 – 92.6 °C (37 mg, 53% yield), $[\alpha]_D^{20}$ = -59.3 (c = 2.8, CHCl$_3$).

TLC: Rf = 0.4 (Petroleum ether : EtOAc = 5 : 1).

HPLC: The *ee* value was determined by HPLC analysis on a CHIRALPAK IE-3 column (hexane/isopropanol = 70/30, flow = 0.6 mL/min, 254 nm) with t_r = 16.9 min (major), 19.9 min (minor): >99% *ee*.

^{1}H NMR (600 MHz, CDCl$_3$) δ 7.26 (d, J = 8.4 Hz, 1H), 6.97 (d, J = 2.4 Hz, 1H), 6.82 (dd, J_1 = 8.4 Hz, J_2 = 2.4 Hz, 1H), 4.07 – 4.01 (m, 1H), 3.99 – 3.93 (m, 1H), 3.83 (s, 3H), 3.78 – 3.70 (m, 4H), 3.68 (s, 3H), 3.07 – 2.94 (m, 2H), 2.86 – 2.79 (m, 1H), 2.79 – 2.71 (m, 1H), 2.70 – 2.60 (m, 2H).

^{13}C NMR (150 MHz, CDCl$_3$) δ 171.6, 171.3, 157.3, 155.6, 149.9, 121.5, 118.6, 111.1, 110.8, 96.4, 63.1, 55.7, 51.9, 51.7, 47.2, 47.0, 34.7, 21.7.

HRMS (ESI-TOF) m/z: $[M+H]^+$ Calcd for $C_{18}H_{22}NO_6$ 348.1447; Found: 348.1448.

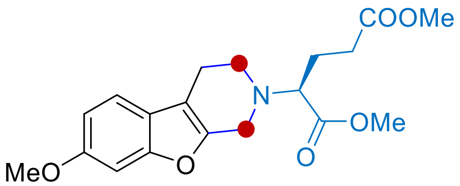

4l

dimethyl (*S*)-2-(7-methoxy-3,4-dihydrobenzofuro[2,3-*c*]pyridin-2(1*H*)-yl)pentanedioate

4l: Pale yellow solid, m. p. 54.9 – 56.0 °C (39 mg, 54% yield), $[\alpha]_D^{20}$ = 43.7 (c = 2.5, CHCl$_3$).

TLC: Rf = 0.3 (Petroleum ether : EtOAc = 10 : 1).

HPLC: The *ee* value was determined by HPLC analysis on a CHIRALPAK IE-3 column (hexane/isopropanol = 70/30, flow = 0.6 mL/min, 254 nm) with t_r = 19.9 min (major), 22.5 min (minor): >99% *ee*.

^{1}H NMR (600 MHz, CDCl$_3$) δ 7.26 (d, *J* = 8.4 Hz, 1H), 6.97 (d, *J* = 2.4 Hz, 1H), 6.83 (dd, J_1 = 8.4 Hz, J_2 = 2.4 Hz, 1H), 3.94 – 3.87 (m, 1H), 3.83 (s, 3H), 3.78 – 3.72 (m, 1H), 3.72 (s, 3H), 3.60 (s, 3H), 3.54 – 3.48 (m, 1H), 3.11 – 3.04 (m, 1H), 2.85 – 2.77 (m, 1H), 2.69 – 2.58 (m, 2H), 2.50 – 2.39 (m, 2H), 2.21 – 2.07 (m, 2H).

^{13}C NMR (150 MHz, CDCl$_3$) δ 173.6, 172.2, 157.3, 155.6, 150.3, 121.6, 118.5, 111.1, 110.8, 96.4, 65.8, 55.8, 51.6, 51.4, 46.9, 46.6, 30.7, 24.4, 21.6.

HRMS (ESI-TOF) m/z: $[M+H]^+$ Calcd for $C_{19}H_{24}NO_6$ 362.1604; Found: 362.1613.

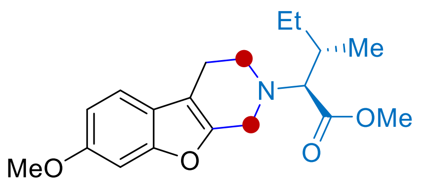

4m

methyl (2*S*,3*S*)-2-(7-methoxy-3,4-dihydrobenzofuro[2,3-*c*]pyridin-2(1*H*)-yl)-3-methylpentanoate

4m: Pale yellow solid, m. p. 91.9 – 93.0 °C (41 mg, 62% yield), $[\alpha]_D^{20}$ = -24.0 (c = 2.1, CHCl$_3$).

TLC: Rf = 0.3 (Petroleum ether : EtOAc = 20 : 1).

HPLC: The *ee* value was determined by HPLC analysis on a CHIRALPAK IC-3 column (hexane/isopropanol = 95/5, flow = 1.0 mL/min, 254 nm) with t_r = 4.8 min (minor), 5.8 min (major): >99% *ee*.

^{1}H NMR (600 MHz, CDCl$_3$) δ 7.26 (d, *J* = 7.8 Hz, 1H), 6.97 (d, *J* = 1.8 Hz, 1H), 6.83 (dd, J_1 = 8.4 Hz, J_2 = 1.8 Hz, 1H), 3.83 (s, 3H), 3.82 – 3.73 (m, 2H), 3.71 (s, 3H), 3.13 (d, *J* = 10.2 Hz, 1H), 3.03 – 2.95 (m, 1H), 2.81 – 2.73 (m, 1H), 2.71 – 2.57 (m, 2H), 2.07 – 1.95 (m, 1H), 1.80 – 1.69 (m, 1H), 1.24 – 1.12 (m, 1H), 0.93 – 0.85 (m, 6H).

^{13}C NMR (150 MHz, CDCl$_3$) δ 172.3, 157.2, 155.6, 150.6, 121.7, 118.5, 111.1, 110.7, 96.4, 72.1, 55.8, 50.8, 47.2, 46.6, 32.9, 25.0, 21.7, 15.9, 10.5.

HRMS (ESI-TOF) m/z: $[M+H]^+$ Calcd for $C_{19}H_{26}NO_4$ 332.1862; Found: 332.1863.

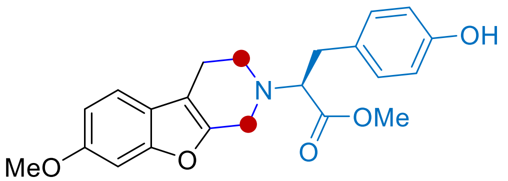

4n

methyl (*S*)-3-(4-hydroxyphenyl)-2-(7-methoxy-3,4-dihydrobenzofuro[2,3-*c*]pyridin-2(1*H*)-yl)propanoate

4n: Pale yellow solid, m. p. 121.2 – 123.9 °C (34 mg, 45% yield), $[\alpha]_D^{20}$ = -24.7 (c = 4.1, $CHCl_3$).

TLC: Rf = 0.4 (Petroleum ether : EtOAc = 2 : 1).

HPLC: The *ee* value was determined by HPLC analysis on a CHIRALPAK AD-H column (hexane/isopropanol = 80/20, flow = 1.0 mL/min, 254 nm) with t_r = 12.2 min (minor), 14.3 min (major): >99% *ee*.

^{1}H NMR (600 MHz, $CDCl_3$) δ 7.27 (d, *J* = 8.4 Hz, 1H), 7.05 (d, *J* = 7.8 Hz, 2H), 7.00 – 6.94 (d, *J* = 1.8 Hz 1H), 6.87 – 6.81 (dd, J_1 = 8.4 Hz, J_2 = 1.8 1H), 6.70 (d, *J* = 7.8 Hz, 2H), 5.67 (s, 1H), 3.96 – 3.86 (m, 2H), 3.84 (s, 3H), 3.71 – 3.65 (m, 1H), 3.62 (s, 3H), 3.16 – 3.04 (m, 2H), 3.03 – 2.96 (m, 1H), 2.95 – 2.87 (m, 1H), 2.75 – 2.61 (m, 2H).

^{13}C NMR (150 MHz, $CDCl_3$) δ 172.2, 157.3, 155.7, 154.5, 149.9, 130.3, 129.5, 121.6, 118.6, 115.4, 111.2, 110.9, 96.4, 69.2, 55.8, 51.4, 47.5, 46.8, 35.2, 21.5.

HRMS (ESI-TOF) m/z: $[M+H]^+$ Calcd for $C_{22}H_{24}NO_5$ 382.1654; Found: 382.1657.

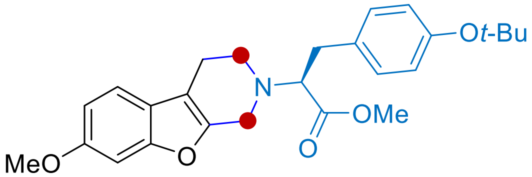

4o

methyl (*S*)-3-(4-(*tert*-butoxy)phenyl)-2-(7-methoxy-3,4-dihydrobenzofuro[2,3-*c*]pyridin-2(1*H*)-yl)propanoate

4o: Pale yellow solid, m. p. 102.1 – 104.9 °C (55 mg, 63% yield), $[\alpha]_D^{20}$ = -16.2 (c = 2.0, $CHCl_3$).

TLC: Rf = 0.4 (Petroleum ether : EtOAc = 5 : 1).

^{1}H NMR (400 MHz, CDCl$_3$) δ 7.31 (d, J = 8.8 Hz, 1H), 7.15 (d, J = 8.0 Hz, 2H), 7.01 (d, J = 2.0 Hz, 1H), 6.93 (d, J = 8.4 Hz, 2H), 6.87 (dd, J_1 = 8.4 Hz, J_2 = 2.0 Hz, 1H), 4.01 – 3.90 (m, 2H), 3.87 (s, 3H), 3.77 – 3.69 (m, 1H), 3.63 (s, 3H), 3.25 – 3.15 (m, 1H), 3.17 – 3.02 (m, 2H), 3.00 – 2.90 (m, 1H), 2.79 – 2.62 (m, 2H), 1.36 (s, 9H).

^{13}C NMR (100 MHz, CDCl$_3$) δ 172.1, 157.3, 155.7, 153.9, 150.1, 132.6, 129.6, 124.2, 121.6, 118.6, 111.2, 110.8, 96.4, 78.4, 69.1, 55.8, 51.3, 47.5, 46.9, 35.5, 28.9, 21.6.

HRMS (ESI-TOF) m/z: $[M+H]^+$ Calcd for $C_{26}H_{32}NO_5$ 438.2280; Found: 438.2285.

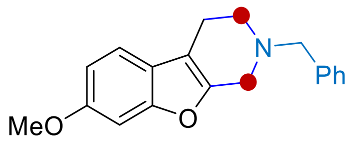

4p

2-benzyl-7-methoxy-1,2,3,4-tetrahydrobenzofuro[2,3-*c*]pyridine

4p: Pale yellow solid, m. p. 98.5 – 99.7 °C (45 mg, 76% yield).

TLC: Rf = 0.2 (Petroleum ether : EtOAc = 8 : 1).

^{1}H NMR (600 MHz, CDCl$_3$) δ 7.41 – 7.38 (m, 2H), 7.35 (t, J = 7.6 Hz, 2H), 7.29 – 7.27 (m, 2H), 6.96 (d, J = 2.4 Hz, 1H), 6.83 (dd, J_1 = 8.4 Hz, J_2 = 2.4 Hz, 1H), 3.83 (s, 3H), 3.78 (s, 2H), 3.64 (t, J = 2.4 Hz, 2H), 2.86 (t, J = 6.0 Hz, 2H), 2.71 – 2.69 (m, 2H).

^{13}C NMR (100 MHz, CDCl$_3$) δ 157.3, 155.7, 150.3, 138.1, 129.2, 128.5, 127.4, 121.7, 118.6, 111.1, 110.8, 96.4, 61.9, 55.8, 50.2, 50.1, 20.9.

HRMS (ESI-TOF) m/z: $[M+H]^+$ Calcd for $C_{19}H_{20}NO_2$ 294.1494; Found: 294.1499.

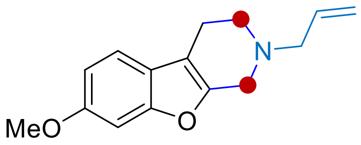

4q

2-allyl-7-methoxy-1,2,3,4-tetrahydrobenzofuro[2,3-*c*]pyridine

4q: Pale yellow gum, (30 mg, 62% yield).

TLC: Rf = 0.3 (Petroleum ether : EtOAc = 10 : 1).

^{1}H NMR (400 MHz, CDCl$_3$) δ 7.28 (d, J = 8.8 Hz, 1H), 6.97 (d, J = 2.0 Hz, 1H), 6.83 (dd, J_1 = 8.4 Hz, J_2 = 2.0 Hz, 1H), 6.03 – 5.89 (m, 1H), 5.31 – 5.18 (m, 2H), 3.84 (s, 3H), 3.65 (t, J = 2.0 Hz, 2H), 3.30 – 3.23 (m, 2H), 2.85 (t, J = 5.6 Hz, 2H), 2.75 – 2.67 (m, 2H).

^{13}C NMR (100 MHz, $CDCl_3$) δ 157.3, 155.7, 150.2, 135.1, 121.6, 118.6, 118.4, 111.1, 110.8, 96.4, 60.6, 55.8, 50.0, 20.8.

HRMS (ESI-TOF) m/z: $[M+H]^+$ Calcd for $C_{15}H_{18}NO_2$ 244.1338; Found: 244.1341.

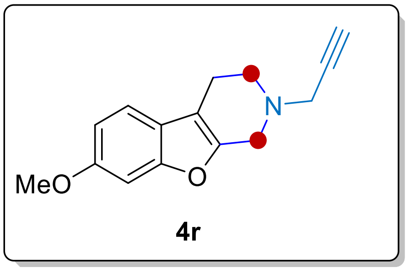

7-methoxy-2-(prop-2-yn-1-yl)-1,2,3,4-tetrahydrobenzofuro[2,3-*c*]pyridine

4r: White solid, m. p. 90.4 – 93.1 °C (35 mg, 73% yield).

TLC: Rf = 0.3 (Petroleum ether : EtOAc = 10 : 1).

^{1}H NMR (400 MHz, $CDCl_3$) δ 7.32 (d, J = 8.4 Hz, 1H), 7.02 (d, J = 2.4 Hz, 1H), 6.88 (dd, J_1 = 8.4 Hz, J_2 = 2.4 Hz, 1H), 3.88 (s, 3H), 3.82 (t, J = 2.0 Hz, 2H), 3.62 (d, J = 2.4 Hz, 2H), 2.96 (t, J = 5.6 Hz, 2H), 2.84 – 2.73 (m, 2H), 2.35 (t, J = 2.4 Hz, 1H).

^{13}C NMR (100 MHz, $CDCl_3$) δ 157.3, 155.7, 149.9, 121.5, 118.6, 110.8, 110.8, 96.4, 78.5, 73.7, 55.8, 49.4, 48.7, 46.3, 21.0.

HRMS (ESI-TOF) m/z: $[M+H]^+$ Calcd for $C_{15}H_{16}NO_2$ 242.1181; Found: 242.1184.

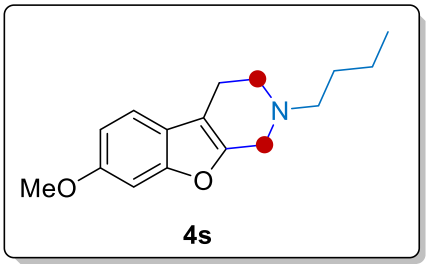

2-butyl-7-methoxy-1,2,3,4-tetrahydrobenzofuro[2,3-*c*]pyridine

4s: Orange gum (37 mg, 72% yield).

TLC: Rf = 0.4 (Petroleum ether : EtOAc = 10 : 1).

^{1}H NMR (600 MHz, $CDCl_3$) δ 7.27 (d, J = 8.4 Hz, 1H), 6.97 (d, J = 2.0 Hz, 1H), 6.83 (dd, J_1 = 8.4 Hz, J_2 = 2.4 Hz, 1H), 3.83 (s, 3H), 3.63 (t, J = 2.4 Hz, 2H), 2.82 (t, J = 6.0 Hz, 2H), 2.75 – 2.65 (m, 2H), 2.65 – 2.52 (m, 2H), 1.68 – 1.44 (m, 2H), 1.49 – 1.33 (m, 2H), 0.95 (t, J = 7.2 Hz, 3H).

^{13}C NMR (125 MHz, $CDCl_3$) δ 157.2, 155.6, 150.4, 121.7, 118.6, 111.1, 110.7, 96.4, 57.5, 55.8, 50.6, 50.3, 29.6, 20.9, 20.7, 14.1.

HRMS (ESI-TOF) m/z: $[M+H]^+$ Calcd for $C_{16}H_{22}NO_2$ 260.1651; Found: 260.1656.

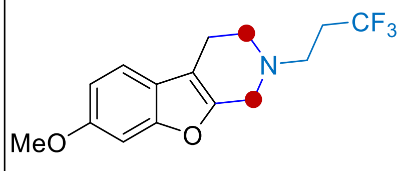

4t

7-methoxy-2-(3,3,3-trifluoropropyl)-1,2,3,4-tetrahydrobenzofuro[2,3-*c*]pyridine

4t: Orange gum (51 mg, 85% yield).

TLC: Rf = 0.3 (Petroleum ether : EtOAc = 10 : 1).

^{1}H NMR (500 MHz, $CDCl_3$) δ 7.29 (d, J = 8.5 Hz, 1H), 6.98 (d, J = 2.5 Hz, 1H), 6.84 (dd, J_1 = 8.5 Hz, J_2 = 2.5 Hz, 1H), 3.84 (s, 3H), 3.68 (t, J = 2.0 Hz, 2H), 2.91 – 2.83 (m, 4H), 2.76 – 2.66 (m, 2H), 2.48 – 2.34 (m, 2H).

^{13}C NMR (125 MHz, $CDCl_3$) δ 157.4, 155.7, 149.5, 126.5 (q, J_{C-F} = 312.5 Hz), 121.4, 118.7, 111.1, 110.9, 96.4, 55.8, 50.4, 50.1, 49.9 (q, J_{C-F} = 3.8 Hz), 32.5 (q, J_{C-F} = 27.5 Hz), 20.8.

HRMS (ESI-TOF) m/z: $[M+H]^+$ Calcd for $C_{15}H_{17}F_3NO_2$ 300.1211; Found: 300.1211.

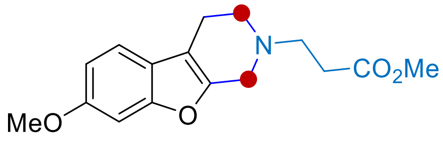

4u

methyl 3-(7-methoxy-3,4-dihydrobenzofuro[2,3-*c*]pyridin-2(1*H*)-yl)propanoate

4u: Orange gum (44 mg, 76% yield).

TLC: Rf = 0.3 (Petroleum ether : EtOAc = 5 : 1).

^{1}H NMR (400 MHz, $CDCl_3$) δ 7.28 (d, J = 8.4 Hz, 1H), 6.97 (d, J = 2.4 Hz, 1H), 6.83 (dd, J_1 = 8.4 Hz, J_2 = 2.4 Hz, 1H), 3.84 (s, 3H), 3.70 (s, 3H), 3.68 (t, J = 2.0 Hz, 2H), 2.96 (t, J = 7.2 Hz, 2H), 2.86 (t, J = 5.6 Hz, 2H), 2.74 – 2.64 (m, 2H), 2.62 (t, J = 7.2 Hz, 2H).

^{13}C NMR (100 MHz, $CDCl_3$) δ 172.8, 157.3, 155.6, 149.9, 121.5, 118.6, 111.1, 110.8, 96.4, 55.8, 52.5, 51.8, 50.3, 49.9, 32.7, 20.8.

HRMS (ESI-TOF) m/z: $[M+H]^+$ Calcd for $C_{16}H_{20}NO_4$ 290.1392; Found: 290.1393.

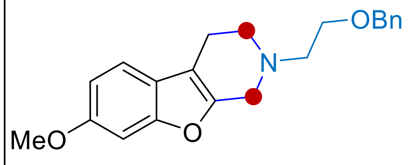

4v

2-(2-(benzyloxy)ethyl)-7-methoxy-1,2,3,4-tetrahydrobenzofuro[2,3-*c*]pyridine

4v: Pale yellow solid, m. p. 65.3 – 66.6 °C (50 mg, 74% yield).

TLC: Rf = 0.3 (Petroleum ether : EtOAc = 4 : 1).

^{1}H NMR (500 MHz, $CDCl_3$) δ 7.45 – 7.16 (m, 6H), 6.97 (d, J = 2.0 Hz, 1H), 6.83 (dd, J_1 = 8.5 Hz, J_2 = 2.0 Hz, 1H), 4.57 (s, 2H), 3.83 (s, 3H), 3.73 (t, J = 2.0 Hz, 2H), 3.68 (t, J = 6.0 Hz, 2H), 2.93 – 2.85 (m, 4H), 2.74 – 2.66 (m, 2H).

^{13}C NMR (125 MHz, $CDCl_3$) δ 157.3, 155.6, 150.2, 138.3, 128.4, 127.8, 127.7, 121.6, 118.6, 111.0, 110.7, 96.4, 73.3, 68.3, 56.7, 55.8, 50.8, 50.5, 20.8.

HRMS (ESI-TOF) m/z: $[M+H]^+$ Calcd for $C_{21}H_{24}NO_3$ 338.1756; Found: 338.1760.

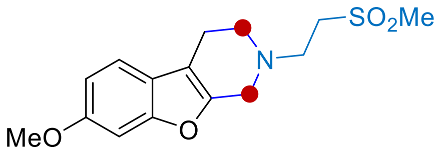

4w

7-methoxy-2-(2-(methylsulfonyl)ethyl)-1,2,3,4-tetrahydrobenzofuro[2,3-*c*]pyridine

4w: White solid, m. p. 168.9 – 170.1 °C (49 mg, 79% yield).

TLC: Rf = 0.3 (Petroleum ether : EtOAc = 8 : 1).

^{1}H NMR (500 MHz, $CDCl_3$) δ 7.29 (d, J = 8.5 Hz, 1H), 6.98 (d, J = 2.5 Hz, 1H), 6.85 (dd, J_1 = 8.5, J_2 = 2.0 Hz, 1H), 3.84 (s, 3H), 3.71 (t, J = 2.0 Hz, 2H), 3.29 – 3.21 (m, 2H), 3.19 – 3.12 (m, 2H), 3.03 (s, 3H), 2.91 (t, J = 5.5 Hz, 2H), 2.77 – 2.66 (m, 2H).

^{13}C NMR (100 MHz, $CDCl_3$) δ 157.5, 155.7, 149.2, 121.2, 118.7, 111.1, 111.0, 96.4, 55.8, 52.8, 50.8, 50.3, 49.9, 42.6, 20.7.

HRMS (ESI-TOF) m/z: $[M+H]^+$ Calcd for $C_{15}H_{20}NO_4S$ 310.1113; Found: 310.1112.

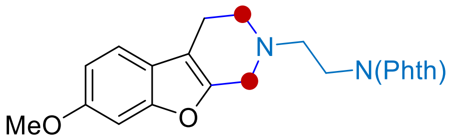

4x

2-(2-(7-methoxy-3,4-dihydrobenzofuro[2,3-*c*]pyridin-2(1*H*)-yl)ethyl)isoindoline-1,3-dione

4x: Pale yellow solid, m. p. 134.5 – 136.0 °C (57 mg, 76% yield).

TLC: Rf = 0.3 (Petroleum ether : EtOAc = 1 : 1).

^{1}H NMR (500 MHz, $CDCl_3$) δ 7.87 – 7.79 (m, 2H), 7.72 – 7.66 (m, 2H), 7.26 (d, J = 8.0 Hz, 1H), 6.96 (d, J = 1.5 Hz, 1H), 6.82 (dd, J_1 = 8.0 Hz, J_2 = 1.5 Hz, 1H), 3.92 (t, J = 6.5 Hz, 2H), 3.83 (s, 3H), 3.73 (s, 2H), 2.92 (t, J = 6.0 Hz, 4H), 2.65 (s, 2H).

^{13}C NMR (125 MHz, $CDCl_3$) δ 168.4, 157.3, 155.6, 150.0, 133.9, 132.2, 123.3, 121.6, 118.6, 111.2, 110.8, 96.4, 55.8, 54.5, 50.3, 49.9, 35.7, 20.7.

HRMS (ESI-TOF) m/z: $[M+H]^+$ Calcd for $C_{22}H_{21}N_2O_4$ 377.1501; Found: 377.1509.

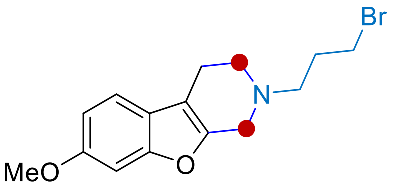

4y

2-(3-bromopropyl)-7-methoxy-1,2,3,4-tetrahydrobenzofuro[2,3-*c*]pyridine

4y: Orange solid, m. p. 76.6 – 78.9 °C (35 mg, 54% yield).

TLC: Rf = 0.3 (Petroleum ether : EtOAc = 10 : 1).

^{1}H NMR (500 MHz, $CDCl_3$) δ 7.28 (d, J = 8.5 Hz, 1H), 6.97 (d, J = 2.0 Hz, 1H), 6.84 (dd, J_1 = 8.5 Hz, J_2 = 2.0 Hz, 1H), 3.84 (s, 3H), 3.66 (t, J = 2.0 Hz, 2H), 3.52 (t, J = 6.5 Hz, 2H), 2.85 (t, J = 6.0 Hz, 2H), 2.77 (t, J = 7.0 Hz, 2H), 2.74 – 2.67 (m, 2H), 2.14 (p, J = 7.0 Hz, 2H).

^{13}C NMR (125 MHz, $CDCl_3$) δ 157.4, 155.6, 149.9, 121.5, 118.6, 111.1, 110.8, 96.4, 77.3, 77.1, 76.8, 55.8, 55.4, 50.5, 50.2, 31.6, 30.5, 29.7, 20.8.

HRMS (ESI-TOF) m/z: $[M+H]^+$ Calcd for $C_{15}H_{19}BrNO_2$ 324.0599; Found: 324.0590.

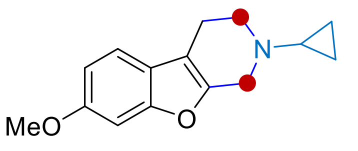

4z

2-cyclopropyl-7-methoxy-1,2,3,4-tetrahydrobenzofuro[2,3-*c*]pyridine

4z: Orange gum (36 mg, 74% yield).

TLC: Rf = 0.3 (Petroleum ether : EtOAc = 10 : 1).

^{1}H NMR (600 MHz, $CDCl_3$) δ 7.27 (d, J = 8.4 Hz, 1H), 6.98 (d, J = 1.8 Hz, 1H), 6.83 (dd, J_1 = 8.4 Hz, J_2 = 2.0 Hz, 1H), 3.83 (s, 3H), 3.81 (t, J = 1.8 Hz, 2H), 3.01 (t, J = 6.0 Hz, 2H), 2.76 – 2.66 (m, 2H), 2.01 – 1.95 (m, 1H), 0.56 (d, J = 5.4 Hz, 4H).

^{13}C NMR (150 MHz, $CDCl_3$) δ 157.3, 155.6, 150.2, 121.7, 118.6, 111.1, 110.7, 96.4, 55.8, 50.4, 37.4, 20.7, 6.5.

HRMS (ESI-TOF) m/z: $[M+H]^+$ Calcd for $C_{15}H_{18}NO_2$ 244.1338; Found: 244.1240.

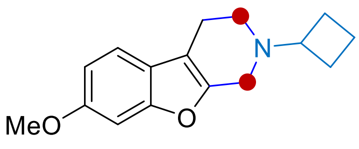

4aa

2-cyclobutyl-7-methoxy-1,2,3,4-tetrahydrobenzofuro[2,3-*c*]pyridine

4aa: Pale yellow solid, m. p. 68.3 – 69.2 °C (32 mg, 62% yield).

TLC: Rf = 0.3 (Petroleum ether : EtOAc = 10 : 1).

^{1}H NMR (400 MHz, $CDCl_3$) δ 7.31 (d, J = 8.4 Hz, 1H), 7.01 (d, J = 2.0 Hz, 1H), 6.86 (dd, J_1 = 8.4 Hz, J_2 = 2.0 Hz, 1H), 3.87 (s, 3H), 3.57 (s, 2H), 3.14 – 2.99 (m, 1H), 2.73 (s, 4H), 2.22 – 2.13 (m, 2H), 2.05 – 1.97 (m, 2H), 1.90 – 1.67 (m, 2H).

^{13}C NMR (100 MHz, $CDCl_3$) δ 157.3, 155.6, 150.2, 121.6, 118.6, 111.2, 110.7, 96.4, 59.3, 55.8, 46.8, 46.3, 27.7, 20.8, 14.4.

HRMS (ESI-TOF) m/z: $[M+H]^+$ Calcd for $C_{16}H_{20}NO_2$ 258.1494; Found: 258.1495 .

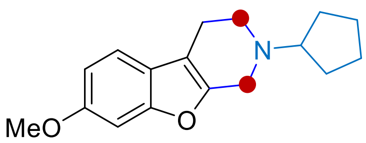

4ab

2-cyclopentyl-7-methoxy-1,2,3,4-tetrahydrobenzofuro[2,3-*c*]pyridine

4ab: Orange solid, m. p. 67.2 – 68.0 °C (35 mg, 65% yield).

TLC: Rf = 0.4 (Petroleum ether : EtOAc = 10 : 1).

^{1}H NMR (600 MHz, $CDCl_3$) δ 7.27 (d, J = 8.4 Hz, 1H), 6.97 (d, J = 2.4 Hz, 1H), 6.83 (dd, J_1 = 8.4 Hz, J_2 = 2.4 Hz, 1H), 3.84 (s, 3H), 3.71 (t, J = 2.4 Hz, 2H), 2.91 – 2.80 (m, 3H), 2.77 – 2.66 (m, 2H), 2.00 – 1.94 (m, 2H), 1.79 – 1.69 (m, 2H), 1.64 – 1.52 (m, 4H).

^{13}C NMR (150 MHz, $CDCl_3$) δ 157.3, 155.6, 150.4, 121.6, 118.6, 111.2, 110.7, 96.4, 66.4, 55.8, 49.5, 49.2, 31.0, 24.1, 21.0.

HRMS (ESI-TOF) m/z: $[M+H]^+$ Calcd for $C_{17}H_{22}NO_2$ 272.1651; Found: 272.1655.

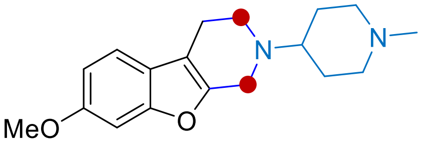

4ac

7-methoxy-2-(1-methylpiperidin-4-yl)-1,2,3,4-tetrahydrobenzofuro[2,3-*c*]pyridine

4ac: Pale yellow solid, m. p. 229.6 – 232.7 °C (30 mg, 50% yield).

TLC: Rf = 0.2 (Dichloromethane : MeOH = 20 : 1).

^{1}H NMR (500 MHz, $CDCl_3$) δ 7.28 (d, J = 7.5 Hz, 1H), 6.97 (d, J = 2.0 Hz, 1H), 6.83 (dd, J_1 = 8.5, J_2 = 2.5 Hz, 1H), 3.84 (s, 3H), 3.76 (t, J = 2.0 Hz, 2H), 3.07 – 2.95 (m, 2H), 2.91 (t, J = 5.6 Hz, 2H), 2.73 – 2.60 (m, 2H), 2.59 – 2.50 (m, 1H), 2.32 (s, 3H), 2.11 – 1.97 (m, 2H), 1.96 – 1.86 (m, 2H), 1.83 – 1.69 (m, 2H).

^{13}C NMR (125 MHz, $CDCl_3$) δ 157.2, 155.7, 150.6, 121.6, 118.6, 111.3, 110.7, 96.3, 60.6, 55.8, 55.2, 46.7, 46.3, 45.9, 28.2, 21.5.

HRMS (ESI-TOF) m/z: $[M+H]^+$ Calcd for $C_{18}H_{25}N_2O_2$ 301.1916; Found: 301.1920.

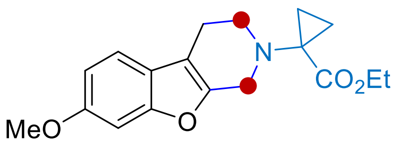

4ad

ethyl 1-(7-methoxy-3,4-dihydrobenzofuro[2,3-*c*]pyridin-2(1*H*)-yl)cyclopropane-1-carboxylate

4ad: Orange solid, m. p. 67.1 – 67.7 °C (25 mg, 40% yield).

TLC: Rf = 0.3 (Petroleum ether : EtOAc = 4 : 1).

^{1}H NMR (400 MHz, $CDCl_3$) δ 7.26 (d, *J* = 8.4 Hz, 1H), 6.97 (d, *J* = 2.0 Hz, 1H), 6.82 (dd, J_1 = 8.5 Hz, J_2 = 2.0 Hz, 1H), 4.21 – 4.14 (m, 2H), 4.14 (s, 2H), 3.84 (s, 3H), 3.30 (s, 2H), 2.73 – 2.49 (m, 2H), 1.39 (q, *J* = 4,0 Hz, 2H), 1.27 (t, *J* = 7.2 Hz, 3H), 1.09 (q, *J* = 4.0 Hz, 2H).

^{13}C NMR (100 MHz, $CDCl_3$) δ 174.2, 157.2, 155.6, 150.7, 121.9, 118.5, 110.9, 110.7, 96.4, 60.5, 55.8, 47.7, 46.3, 45.6, 22.2, 19.4, 14.4.

HRMS (ESI-TOF) m/z: $[M+H]^+$ Calcd for $C_{18}H_{22}NO_4$ 316.1549; Found: 316.1550.

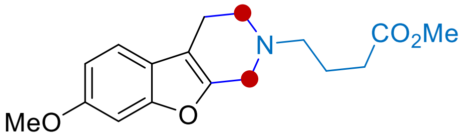

4ae

methyl 4-(7-methoxy-3,4-dihydrobenzofuro[2,3-*c*]pyridin-2(1*H*)-yl)butanoate

4ae: Pale yellow solid, m. p. 56.1 – 57.3 °C (38 mg, 63% yield).

TLC: Rf = 0.3 (Petroleum ether : EtOAc = 10 : 1).

^{1}H NMR (500 MHz, $CDCl_3$) δ 7.28 (d, *J* = 8.0 Hz, 1H), 6.97 (d, *J* = 2.0 Hz, 1H), 6.83 (dd, J_1 = 8.0 Hz, J_2 = 2.0 Hz, 1H), 3.84 (s, 3H), 3.65 (s, 3H), 3.64 (t, *J* = 2.0 Hz, 2H), 2.82 (t, *J* = 5.5 Hz, 2H), 2.72 – 2.66 (m, 2H), 2.63 (t, *J* = 7.5 Hz, 2H), 2.41 (t, *J* = 7.5 Hz, 2H), 1.95-1.89 (m, 2H).

^{13}C NMR (125 MHz, $CDCl_3$) δ 174.0, 157.3, 155.6, 150.2, 121.6, 118.6, 111.1, 110.8, 96.4, 56.6, 55.8, 51.6, 50.3, 50.1, 31.9, 22.7, 20.8.

HRMS (ESI-TOF) m/z: $[M+H]^+$ Calcd for $C_{17}H_{22}NO_4$ 304.1549; Found: 304.1553.

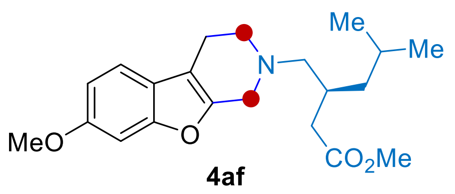

methyl (*S*)-3-((7-methoxy-3,4-dihydrobenzofuro[2,3-*c*]pyridin-2(1*H*)-yl)methyl)-5-methylhexanoate

4af: Colorless oil (43 mg, 60% yield), $[\alpha]_D^{20}$ = -63.3 (c = 3.4, $CHCl_3$).

TLC: Rf = 0.5 (Petroleum ether : EtOAc = 10 : 1).

^{1}H NMR (400 MHz, $CDCl_3$) δ 7.27 (d, J = 8.4 Hz, 1H), 6.97 (d, J = 2.4 Hz, 1H), 6.83 (dd, J_1 = 8.4 Hz, J_1 = 2.4 Hz, 1H), 3.84 (s, 3H), 3.71 – 3.48 (m, 5H), 2.93 – 2.81 (m, 1H), 2.75 – 2.66 (m, 1H), 2.68 – 2.57 (m, 2H), 2.59 – 2.47 (m, 1H), 2.37 – 2.23 (m, 4H), 1.73-1.60 (m, 1H), 1.27 – 1.09 (m, 2H), 0.95 – 0.87 (m, 6H).

^{13}C NMR (100 MHz, $CDCl_3$) δ 174.1, 157.3, 155.6, 150.7, 121.7, 118.6, 111.1, 110.7, 96.4, 62.7, 55.8, 51.3, 50.7, 50.6, 42.7, 38.9, 31.6, 25.4, 22.9, 22.7, 20.9.

HRMS (ESI-TOF) m/z: $[M+H]^+$ Calcd for $C_{21}H_{30}NO_4$ 360.2175; Found: 360.2179.

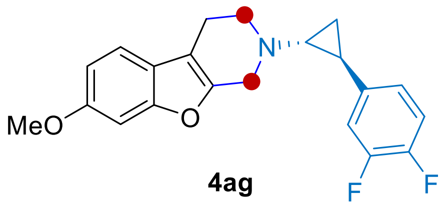

2-((1*R*,2*S*)-2-(3,4-difluorophenyl)cyclopropyl)-7-methoxy-1,2,3,4-tetrahydrobenzofuro[2,3-*c*]pyridine

4ag: Orange gum (49 mg, 69% yield) $[\alpha]_D^{20}$ = -16.2 (c = 2.0, $CHCl_3$), $[\alpha]_D^{20}$ = -71.6 (c = 2.3, $CHCl_3$).

TLC: Rf = 0.4 (Petroleum ether : EtOAc = 5 : 1).

^{1}H NMR (400 MHz, $CDCl_3$) δ 7.28 (d, J = 8.4 Hz, 1H), 7.09 – 7.00 (m, 1H), 6.98 (d, J = 2.0 Hz, 1H), 6.90 – 6.78 (m, 3H), 3.88 – 3.76 (m, 5H), 3.04 (t, J = 5.6 Hz, 2H), 2.75 – 2.67 (m, 2H), 2.23 – 2.12 (m, 1H), 2.12 – 2.04 (m, 1H), 1.31 – 1.22 (m, 1H), 1.10 – 0.93 (m, 1H).

^{13}C NMR (100 MHz, $CDCl_3$) δ 157.4, 155.7, 150.2 (dd, $J_{C\text{-}F1}$ = 246.0 Hz, $J_{C\text{-}F2}$ = 12.0 Hz), 149.8, 148.7 (dd, $J_{C\text{-}F1}$ = 239.0 Hz, $J_{C\text{-}F2}$ = 13.0 Hz), 138.9 (dd, $J_{C\text{-}F1}$ = 6.0 Hz, $J_{C\text{-}F2}$ = 4.0 Hz), 122.1 (dd,

$J_{C\text{-}F1}$ = 6.0 Hz, $J_{C\text{-}F2}$ = 4.0 Hz), 121.5, 118.6, 117.0 (d, $J_{C\text{-}F}$ = 17.0 Hz), 114.9 (d, $J_{C\text{-}F}$ = 17.0 Hz), 111.1, 110.9, 96.4, 55.8, 50.0, 47.6, 24.6, 20.7, 17.2.

HRMS (ESI-TOF) m/z: $[M+H]^+$ Calcd for $C_{21}H_{20}F_2NO_2$ 356.1462; Found: 356.1462.

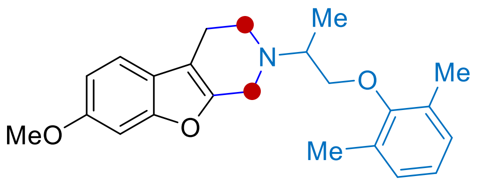

4ah

2-(1-(2,6-dimethylphenoxy)propan-2-yl)-7-methoxy-1,2,3,4-tetrahydrobenzofuro[2,3-*c*]pyridine

4ah: Pale yellow gum (32 mg, 44% yield).

TLC: Rf = 0.3 (Petroleum ether : EtOAc = 10 : 1).

^{1}H NMR (500 MHz, $CDCl_3$) δ 7.29 (d, *J* = 8.5 Hz, 1H), 7.05 – 6.97 (m, 3H), 6.97 – 6.90 (m, 1H), 6.84 (dd, J_1 = 8.5 Hz, J_2 = 2.5 Hz, 1H), 4.02 – 3.95 (m, 1H), 3.91 (t, *J* = 2.0 Hz, 2H), 3.84 (s, 3H), 3.83 – 3.75 (m, 1H), 3.39 – 3.31 (m, 1H), 3.09 – 2.97 (m, 2H), 2.77 – 2.68 (m, 2H), 2.32 (s, 6H), 1.36 (d, *J* = 7.0 Hz, 3H).

^{13}C NMR (150 MHz, $CDCl_3$) δ 157.2, 155.8, 155.7, 150.8, 130.8, 128.9, 123.9, 121.8, 118.6, 111.3, 110.7, 96.4, 73.9, 58.9, 55.8, 46.8, 46.7, 21.8, 16.5, 13.1.

HRMS (ESI-TOF) m/z: $[M+H]^+$ Calcd for $C_{23}H_{28}NO_3$ 366.2069; Found: 366.2074.

Procedure for the synthesis of 5

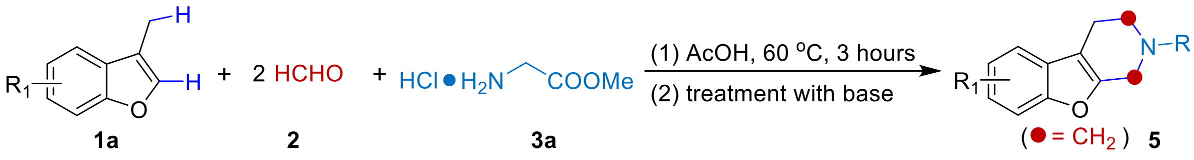

An oven-dried Schlenk tube equipped with a magnetic stirrer bar was charged with **1** (0.2 mmol, 1 equiv.), **2** (0.8 mmol, 4 equiv.) and **3a** (0.4 mmol, 2 equiv.), evacuated and purged with argon for three times. Then 1 mL AcOH was added by a syringe, and the reaction was stirred at 60 °C for 3 hours to be completed. After completion, the reaction was quenched by saturated aqueous $NaHCO_3$. The aqueous layer was extracted with ethyl acetate (three times), and the

combined organic layer was dried over Na_2SO_4 and concentrated. Purification by flash column chromatography afforded the pure product.

Characterization Data

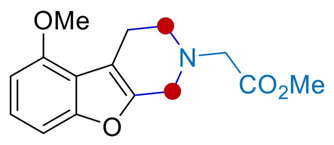

5a

methyl 2-(5-methoxy-3,4-dihydrobenzofuro[2,3-*c*]pyridin-2(1*H*)-yl)acetate

5a: Pale yellow solid, m. p. 92.0 – 93.5 °C (44 mg, 80% yield).

TLC: Rf = 0.3 (Petroleum ether : EtOAc = 4 : 1).

^{1}H NMR (600 MHz, $CDCl_3$) δ 7.11 (t, J = 8.4 Hz, 1H), 7.03 (dd, J_1 = 8.4 Hz, J_2 = 1.2 Hz, 1H), 6.61 (d, J = 7.8 Hz, 1H), 3.88 (s, 3H), 3.85 (d, J = 1.8 Hz, 2H), 3.75 (s, 3H), 3.51 (s, 2H), 3.02 – 2.90 (m, 4H).

^{13}C NMR (125 MHz, $CDCl_3$) δ 170.9, 155.9, 154.2, 148.7, 124.0, 117.7, 110.7, 104.5, 103.3, 57.8, 55.5, 51.9, 50.3, 49.6, 22.1.

HRMS (ESI-TOF) m/z: $[M+H]^+$ Calcd for $C_{15}H_{18}NO_4$ 276.1236; Found: 276.1241.

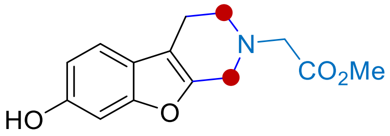

5b

methyl 2-(7-hydroxy-3,4-dihydrobenzofuro[2,3-*c*]pyridin-2(1*H*)-yl)acetate

5b: Orange solid, m. p. 155.4 – 157.6 °C (26 mg, 50% yield).

TLC: Rf = 0.2 (Petroleum ether : acetone = 2 : 1).

^{1}H NMR (600 MHz, $CDCl_3$) δ 7.16 (d, J = 8.4 Hz, 1H), 6.86 (d, J = 1.8 Hz, 1H), 6.71 (dd, J_1 = 8.4 Hz, J_2 = 2.4 Hz, 1H), 3.82 (s, 2H), 3.77 (s, 3H), 3.52 (s, 2H), 2.98 (t, J = 6.0 Hz, 2H), 2.79 – 2.62 (m, 2H).

^{13}C NMR (150 MHz, $CDCl_3$) δ 170.9, 155.4, 153.1, 149.2, 121.5, 118.6, 111.4, 110.9, 98.7, 57.9, 51.9, 50.2, 49.7, 20.6.

HRMS (ESI-TOF) m/z: $[M+H]^+$ Calcd for $C_{14}H_{16}NO_4$ 262.1079; Found: 262.1081.

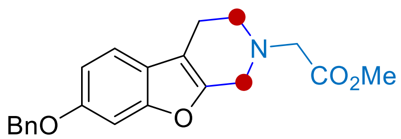

5c

methyl 2-(7-(benzyloxy)-3,4-dihydrobenzofuro[2,3-*c*]pyridin-2(1*H*)-yl)acetate

5c: Pale yellow solid, m. p. 78.3 – 79.9 °C (45 mg, 64% yield).

TLC: Rf = 0.3 (Petroleum ether : EtOAc = 4 : 1).

^{1}H NMR (600 MHz, CDCl$_3$) δ 7.48 – 7.43 (m, 2H), 7.41 – 7.36 (m, 2H), 7.35 – 7.28 (m, 2H), 7.05 (d, J = 1.8 Hz, 1H), 6.92 (dd, J_1 = 8.0 Hz, J_2 = 1.8 Hz, 1H), 5.09 (s, 2H), 3.84 (t, J = 1.8 Hz, 2H), 3.76 (s, 3H), 3.51 (s, 2H), 2.99 (t, J = 6.0 Hz, 2H), 2.74 – 2.72 (m, 2H).

^{13}C NMR (150 MHz, CDCl$_3$) δ 170.9, 156.5, 155.5, 149.7, 137.1, 128.6, 127.9, 127.5, 121.7, 118.6, 111.7, 110.9, 97.7, 70.7, 57.8, 51.8, 50.0, 49.7, 20.6.

HRMS (ESI-TOF) m/z: [M+H]$^+$ Calcd for $C_{21}H_{22}NO_4$ 352.1549; Found: 352.1552.

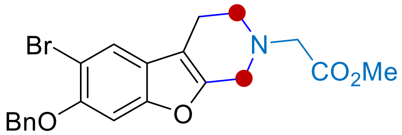

5d

methyl 2-(7-(benzyloxy)-6-bromo-3,4-dihydrobenzofuro[2,3-*c*]pyridin-2(1*H*)-yl)acetate

5d: Orang gum, (69 mg, 81% yield).

TLC: Rf = 0.3 (Petroleum ether : EtOAc = 4 : 1).

^{1}H NMR (500 MHz, CDCl$_3$) δ 7.59 (s, 1H), 7.52 – 7.46 (m, 2H), 7.42 – 7.35 (m, 2H), 7.34 – 7.29 (m, 1H), 7.03 (s, 1H), 5.15 (s, 2H), 3.82 (t, J = 2.0 Hz, 2H), 3.75 (s, 3H), 3.50 (s, 2H), 2.97 (t, J = 5.5 Hz, 2H), 2.77 – 2.65 (m, 2H).

^{13}C NMR (125 MHz, CDCl$_3$) δ 170.8, 154.4, 151.9, 150.7, 136.5, 128.6, 127.9, 127.1, 122.9, 122.3, 110.6, 107.5, 98.1, 71.4, 57.8, 51.9, 49.9, 49.5, 20.6.

HRMS (ESI-TOF) m/z: [M+H]$^+$ Calcd for $C_{21}H_{21}BrNO_4$ 430.0654; Found: 430.0653.

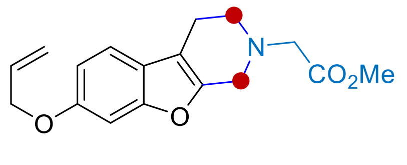

5e

methyl 2-(7-(allyloxy)-3,4-dihydrobenzofuro[2,3-*c*]pyridin-2(1*H*)-yl)acetate

5e: Pale yellow gum (39 mg, 65% yield).

TLC: Rf = 0.3 (Petroleum ether : EtOAc = 4 : 1).

^{1}H NMR (600 MHz, CDCl$_3$) δ 7.28 (d, *J* = 8.4 Hz, 1H), 6.98 (d, *J* = 2.4 Hz, 1H), 6.86 (dd, J_1 = 8.4 Hz, J_2 = 2.4 Hz, 1H), 6.13 – 6.03 (m, 1H), 5.47 – 5.39 (m, 1H), 5.32 – 5.25 (m, 1H), 4.59 – 4.53 (m, 2H), 3.84 (t, *J* = 2.4 Hz, 2H), 3.75 (s, 3H), 3.51 (s, 2H), 2.99 (t, *J* = 5.4 Hz, 2H), 2.76 – 2.71 (m, 2H).

^{13}C NMR (125 MHz, CDCl$_3$) δ 170.9, 156.3, 155.5, 149.6, 133.3, 121.7, 118.6, 117.8, 111.6, 110.9, 97.5, 69.5, 57.8, 51.9, 50.0, 49.7, 20.6.

HRMS (ESI-TOF) m/z: $[M+H]^+$ Calcd for $C_{17}H_{20}NO_4$ 302.1392; Found: 302.1396.

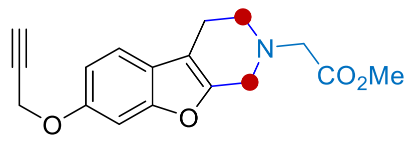

5f

methyl 2-(7-(prop-2-yn-1-yloxy)-3,4-dihydrobenzofuro[2,3-*c*]pyridin-2(1*H*)-yl)acetate

5f: Pale yellow solid, m. p. 74.4 – 75.2 °C (41 mg, 69% yield).

TLC: Rf = 0.3 (Petroleum ether : EtOAc = 5 : 1).

^{1}H NMR (500 MHz, CDCl$_3$) δ 7.31 (d, *J* = 8.5 Hz, 1H), 7.08 (d, *J* = 2.5 Hz, 1H), 6.91 (dd, J_1 = 8.5 Hz, J_2 = 2.5 Hz, 1H), 4.72 (d, *J* = 2.5 Hz, 2H), 3.85 (t, *J* = 2.0 Hz, 2H), 3.76 (s, 3H), 3.52 (s, 2H), 2.99 (t, *J* = 5.5 Hz, 2H), 2.78 – 2.70 (m, 2H), 2.53 (t, *J* = 2.5 Hz, 1H).

^{13}C NMR (125 MHz, CDCl$_3$) δ 170.9, 155.3, 155.2, 149.9, 122.4, 118.7, 111.6, 110.9, 98.0, 78.6, 75.6, 57.8, 56.6, 51.9, 50.0, 49.6, 20.6.

HRMS (ESI-TOF) m/z: $[M+H]^+$ Calcd for $C_{17}H_{18}NO_4$ 300.1236; Found: 300.1235.

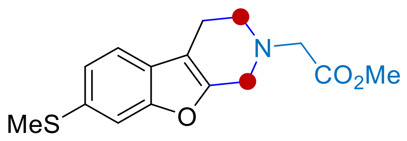

5g

methyl 2-(7-(methylthio)-3,4-dihydrobenzofuro[2,3-*c*]pyridin-2(1*H*)-yl)acetate

5g: Orange gum (38 mg, 65% yield).

TLC: Rf = 0.3 (Petroleum ether : EtOAc = 5 : 1).

^{1}H NMR (400 MHz, $CDCl_3$) δ 7.36 (d, *J* = 1.2 Hz, 1H), 7.33 (d, *J* = 8.4 Hz, 1H), 7.17 (dd, J_1 = 8.4 Hz, J_2 = 1.2 Hz, 1H), 3.86 (t, *J* = 1.6 Hz, 2H), 3.76 (s, 3H), 3.53 (s, 2H), 3.00 (t, *J* = 5.6 Hz, 2H), 2.80 – 2.71 (m, 2H), 2.52 (s, 3H).

^{13}C NMR (100 MHz, $CDCl_3$) δ 170.9, 155.2, 150.5, 133.3, 126.0, 122.8, 118.7, 111.1, 110.3, 57.9, 51.9, 49.9, 49.6, 20.6, 17.3.

HRMS (ESI-TOF) m/z: $[M+H]^+$ Calcd for $C_{15}H_{18}NO_3S$ 292.1007; Found: 292.1012.

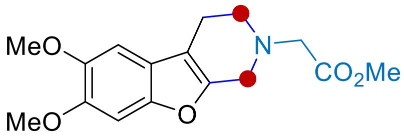

5h

methyl 2-(6,7-dimethoxy-3,4-dihydrobenzofuro[2,3-*c*]pyridin-2(1*H*)-yl)acetate

5h: Orange gum, m. p. 93.8 – 94.6 °C (40 mg, 66% yield).

TLC: Rf = 0.3 (Petroleum ether : EtOAc = 3 : 1).

^{1}H NMR (600 MHz, $CDCl_3$) δ 7.00 (s, 1H), 6.87 (s, 1H), 3.92 (s, 3H), 3.90 (s, 3H), 3.84 (t, *J* = 1.8 Hz, 2H), 3.76 (s, 3H), 3.51 (s, 2H), 3.00 (t, *J* = 6.0 Hz, 2H), 2.74 – 2.72 (m, 2H).

^{13}C NMR (100 MHz, $CDCl_3$) δ 170.9, 149.5, 149.1, 147.1, 146.2, 119.9, 111.1, 100.6, 95.7, 57.9, 56.5, 56.3, 51.8, 50.1, 49.8, 20.7.

HRMS (ESI-TOF) m/z: $[M+H]^+$ Calcd for $C_{16}H_{20}NO_5$ 306.1341; Found: 306.1342.

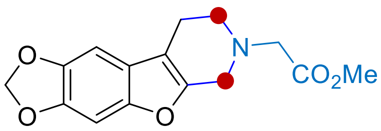

5i

methyl 2-(8,9-dihydro-[1,3]dioxolo[4',5':5,6]benzofuro[2,3-*c*]pyridin-7(6*H*)-yl)acetate

5i: Orange solid, m. p. 158.4 – 160.2 °C (35 mg, 61% yield).

TLC: Rf = 0.3 (Petroleum ether : EtOAc = 4 : 1).

^{1}H NMR (400 MHz, CDCl$_3$) δ 6.96 (s, 1H), 6.83 (s, 1H), 5.99 (s, 2H), 3.85 (t, *J* = 2.0 Hz, 2H), 3.79 (s, 3H), 3.54 (s, 2H), 3.01 (t, *J* = 6.0 Hz, 2H), 2.73 (t, *J* = 6.0 Hz, 2H).

^{13}C NMR (100 MHz, CDCl$_3$) δ 170.9, 149.9, 149.6, 145.2, 144.2, 121.3, 111.4, 101.2, 97.6, 93.7, 57.9, 51.9, 50.1, 49.7, 20.7.

HRMS (ESI-TOF) m/z: $[M+H]^+$ Calcd for $C_{15}H_{16}NO_5$ 290.1028; Found: 290.1032.

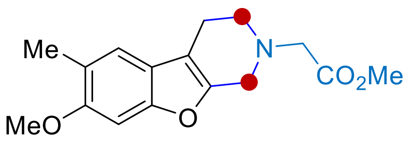

5j

methyl 2-(7-methoxy-6-methyl-3,4-dihydrobenzofuro[2,3-*c*]pyridin-2(1*H*)-yl)acetate

5j: Orange solid, m. p. 67.6 – 69.2 °C (46 mg, 80% yield).

TLC: Rf = 0.3 (Petroleum ether : EtOAc = 4 : 1).

^{1}H NMR (400 MHz, CDCl$_3$) δ 7.19 (s, 1H), 6.96 (s, 1H), 3.89 (s, 3H), 3.87 (t, *J* = 2.0 Hz, 2H), 3.80 (s, 3H), 3.55 (s, 2H), 3.02 (t, *J* = 5.6 Hz, 2H), 2.82 – 2.72 (m, 2H), 2.31 (s, 3H).

^{13}C NMR (100 MHz, CDCl$_3$) δ 171.0, 155.5, 154.1, 148.9, 121.9, 120.5, 119.3, 110.7, 94.0, 57.9, 55.7, 51.9, 50.2, 49.8, 20.7, 16.7.

HRMS (ESI-TOF) m/z: $[M+H]^+$ Calcd for $C_{16}H_{20}NO_4$ 290.1392; Found: 290.1396.

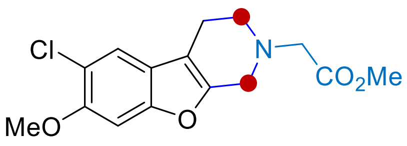

5k

methyl 2-(6-chloro-7-methoxy-3,4-dihydrobenzofuro[2,3-*c*]pyridin-2(1*H*)-yl)acetate

5k: Pale yellow solid, m. p. 85.9 – 87.8 °C (48 mg, 77% yield).

TLC: Rf = 0.3 (Petroleum ether : EtOAc = 4 : 1).

^{1}H NMR (500 MHz, CDCl$_3$) δ 7.40 (s, 1H), 7.03 (s, 1H), 3.92 (s, 3H), 3.84 (t, *J* = 2.0 Hz, 2H), 3.76 (s, 3H), 3.52 (s, 2H), 2.99 (t, *J* = 6.0 Hz, 2H), 2.78 – 2.61 (m, 2H).

^{13}C NMR (125 MHz, CDCl$_3$) δ 170.8, 153.9, 152.2, 150.6, 121.6, 119.3, 117.9, 110.7, 96.1, 57.8, 56.6, 51.9, 49.9, 49.6, 20.6.

HRMS (ESI-TOF) m/z: $[M+H]^+$ Calcd for $C_{15}H_{17}ClNO_4$ 310.0846; Found: 310.0843.

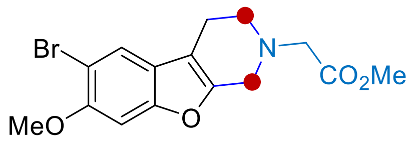

5l

methyl 2-(6-bromo-7-methoxy-3,4-dihydrobenzofuro[2,3-*c*]pyridin-2(1*H*)-yl)acetate

5l: Pale yellow solid, m. p. 84.7 – 86.8 °C (55 mg, 78% yield).

TLC: Rf = 0.3 (Petroleum ether : EtOAc = 4 : 1).

^{1}H NMR (600 MHz, $CDCl_3$) δ 7.56 (s, 1H), 6.99 (s, 1H), 3.90 (s, 3H), 3.83 (s, 2H), 3.75 (s, 3H), 3.50 (s, 2H), 2.97 (t, J = 6.0 Hz, 2H), 2.74 – 2.66 (m, 2H).

^{13}C NMR (100 MHz, $CDCl_3$) δ 170.8, 154.6, 152.9, 150.5, 122.4, 122.3, 110.5, 106.6, 96.0, 57.8, 56.6, 51.9, 49.9, 49.5, 20.6.

HRMS (ESI-TOF) m/z: $[M+H]^+$ Calcd for $C_{15}H_{17}BrNO_4$ 354.0341; Found: 354.0341.

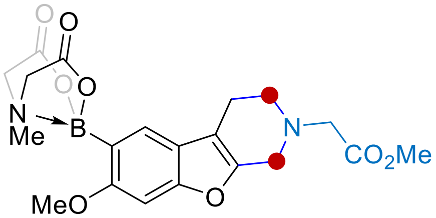

5m

methyl 2-(7-methoxy-6-(6-methyl-4,8-dioxo-1,3,6,2-dioxazaborocan-2-yl)-3,4-dihydrobenzofuro[2,3-*c*]pyridin-2(1*H*)-yl)acetate

5m: Yellow gum (64 mg, 74% yield).

TLC: Rf = 0.3 (Petroleum ether : acetone = 1 : 1).

^{1}H NMR (600 MHz, DMSO) δ 7.54 (s, 1H), 7.16 (s, 1H), 4.36 (d, J = 16.8 Hz, 2H), 4.03 (d, J = 16.8 Hz, 2H), 3.76 (s, 2H), 3.75 (s, 3H), 3.65 (s, 3H), 3.54 (s, 2H), 2.91 (t, J = 5.4 Hz, 2H), 2.64 (d, J = 5.4 Hz, 2H), 2.58 (s, 3H).

^{13}C NMR (125 MHz, DMSO) δ 171.2, 169.9, 160.4, 156.5, 150.0, 124.1, 120.9, 111.1, 94.9, 63.8, 57.4, 55.9, 51.7, 49.5, 48.9, 47.9, 20.8.

HRMS (ESI-TOF) m/z: $[M+H]^+$ Calcd for $C_{20}H_{24}BN_2O_8$ 431.1626; Found: 431.1630.

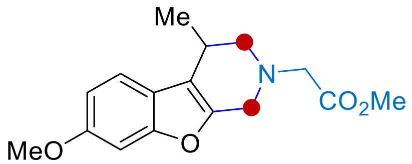

5n

methyl 2-(7-methoxy-4-methyl-3,4-dihydrobenzofuro[2,3-*c*]pyridin-2(1*H*)-yl)acetate

5n: Pale yellow gum (54 mg, 93% yield).

TLC: Rf = 0.3 (Petroleum ether : EtOAc = 4 : 1).

^{1}H NMR (600 MHz, $CDCl_3$) δ 7.36 (d, J = 8.4 Hz, 1H), 6.96 (d, J = 1.8 Hz, 1H), 6.82 (dd, J_1 = 8.4 Hz, J_2 = 2.4 Hz, 1H), 3.85 – 3.76 (m, 5H), 3.74 (s, 3H), 3.49 (s, 2H), 3.17 – 3.06 (m, 1H), 3.08 – 2.96 (m, 1H), 2.58 – 2.46 (m, 1H), 1.32 (d, J = 6.6 Hz, 3H).

^{13}C NMR (150 MHz, $CDCl_3$) δ 170.9, 157.2, 155.8, 149.2, 121.0, 119.5, 115.6, 110.8, 96.5, 58.3, 57.9, 55.7, 51.7, 49.7, 27.7, 17.8.

HRMS (ESI-TOF) m/z: $[M+H]^+$ Calcd for $C_{16}H_{20}NO_4$ 290.1392; Found: 290.1398.

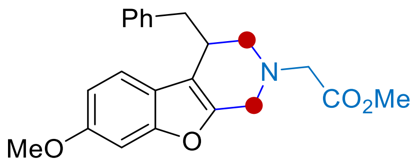

5o

methyl 2-(4-benzyl-7-methoxy-3,4-dihydrobenzofuro[2,3-*c*]pyridin-2(1*H*)-yl)acetate

5o: Yellow gum (64 mg, 88% yield).

TLC: Rf = 0.3 (Petroleum ether : EtOAc = 4 : 1).

^{1}H NMR (500 MHz, $CDCl_3$) δ 7.37 – 7.19 (m, 5H), 7.09 – 6.94 (m, 2H), 6.84 – 6.68 (m, 1H), 3.92 – 3.85 (m, 1H), 3.82 (s, 3H), 3.80 – 3.73 (m, 1H), 3.73 (s, 3H), 3.56 – 3.41 (m, 2H), 3.32 – 3.24 (m, 1H), 3.24 – 3.15 (m, 1H), 2.89 – 2.63 (m, 3H).

^{13}C NMR (125 MHz, $CDCl_3$) δ 170.9, 157.2, 155.7, 149.9, 140.5, 129.4, 128.5, 126.2, 120.9, 119.3, 114.5, 110.8, 96.3, 58.3, 55.7, 54.7, 51.7, 49.8, 39.5, 35.3.

HRMS (ESI-TOF) m/z: $[M+H]^+$ Calcd for $C_{22}H_{24}NO_4$ 366.1705; Found: 366.1710.

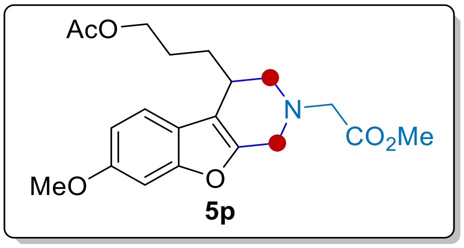

methyl 2-(4-(3-acetoxypropyl)-7-methoxy-3,4-dihydrobenzofuro[2,3-*c*]pyridin-2(1*H*)-yl)acetate

5p: Pale yellow gum (68 mg, 91% yield).

TLC: Rf = 0.4 (Petroleum ether : EtOAc = 4 : 1).

^{1}H NMR (500 MHz, $CDCl_3$) δ 7.34 (d, *J* = 8.5 Hz, 1H), 6.98 (d, *J* = 2.5 Hz, 1H), 6.84 (dd, J_1 = 8.5 Hz, J_2 = 2.5 Hz, 1H), 4.17 – 4.04 (m, 2H), 3.89 – 3.81 (m, 4H), 3.80 – 3.73 (m, 4H), 3.57 – 3.45 (m, 2H), 3.07 – 2.93 (m, 2H), 2.80 – 2.71 (m, 1H), 2.05 (s, 3H), 2.02 – 1.92 (m, 1H), 1.80 – 1.71 (m, 3H).

^{13}C NMR (125 MHz, $CDCl_3$) δ 171.2, 170.9, 157.2, 155.7, 149.8, 120.9, 119.3, 114.3, 110.9, 96.5, 64.6, 58.2, 55.8, 55.2, 51.8, 49.8, 32.6, 29.2, 26.3, 21.0.

HRMS (ESI-TOF) m/z: $[M+H]^+$ Calcd for $C_{20}H_{26}NO_6$ 376.1760; Found: 376.1765.

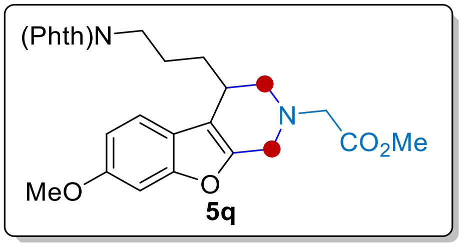

methyl 2-(4-(3-(1,3-dioxoisoindolin-2-yl)propyl)-7-methoxy-3,4-dihydrobenzofuro[2,3-*c*]pyridin-2(1*H*)-yl)acetate

5q: Pale yellow gum (78 mg, 85% yield).

TLC: Rf = 0.2 (Petroleum ether : EtOAc = 1 : 1).

^{1}H NMR (600 MHz, $CDCl_3$) δ 7.81 (dd, J_1 = 5.4 Hz, J_2 = 3.0 Hz, 2H), 7.68 (dd, J_1 = 5.4 Hz, J_2 = 3.0 Hz, 2H), 7.29 (d, *J* = 8.4 Hz, 1H), 6.93 (d, *J* = 2.4 Hz, 1H), 6.76 (dd, J_1 = 8.4 Hz, J_2 = 2.4 Hz, 1H), 3.80 (s, 3H), 3.78 – 3.68 (m, 7H), 3.52 – 3.42 (m, 2H), 3.03 – 2.97 (m, 1H), 2.98 – 2.91 (m, 1H), 2.75 – 2.64 (m, 1H), 2.03 – 1.92 (m, 1H), 1.85 – 1.68 (m, 3H).

^{13}C NMR (150 MHz, $CDCl_3$) δ 170.8, 168.4, 157.2, 155.7, 149.8, 133.9, 132.1, 123.2, 120.9, 119.3, 114.3, 110.9, 96.5, 58.2, 55.7, 55.1, 51.7, 49.7, 38.1, 32.6, 29.9, 26.3.

HRMS (ESI-TOF) m/z: $[M+H]^+$ Calcd for $C_{26}H_{27}N_2O_6$ 463.1869; Found: 463.1869.

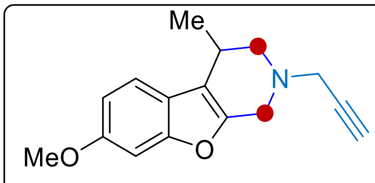

5r

7-methoxy-4-methyl-2-(prop-2-yn-1-yl)-1,2,3,4-tetrahydrobenzofuro[2,3-*c*]pyridine

5r: Pale yellow gum (45 mg, 89% yield).

TLC: Rf = 0.3 (Petroleum ether : EtOAc = 5 : 1).

^{1}H NMR (500 MHz, $CDCl_3$) δ 7.37 (d, J = 8.5 Hz, 1H), 6.98 (d, J = 2.5 Hz, 1H), 6.83 (dd, J_1 = 8.5 Hz, J_2 = 2.5 Hz, 1H), 3.83 (s, 3H), 3.81 – 3.68 (m, 2H), 3.57 (t, J = 2.5 Hz, 2H), 3.19 – 3.07 (m, 1H), 3.04 – 2.93 (m, 1H), 2.54 – 2.42 (m, 1H), 2.31 (t, J = 2.5 Hz, 1H), 1.34 (d, J = 7.0 Hz, 3H).

^{13}C NMR (125 MHz, $CDCl_3$) δ 157.2, 155.8, 149.6, 121.0, 119.4, 115.5, 110.8, 96.5, 78.5, 73.7, 57.6, 55.8, 48.7, 46.3, 27.9, 18.1.

HRMS (ESI-TOF) m/z: $[M+H]^+$ Calcd for $C_{16}H_{18}NO_2$ 256.1338; Found: 256.1340.

HRMS (ESI-TOF) m/z: $[M+H]^+$ Calcd for $C_{33}H_{38}NO_5$ 572.2437; Found: 572.2433.

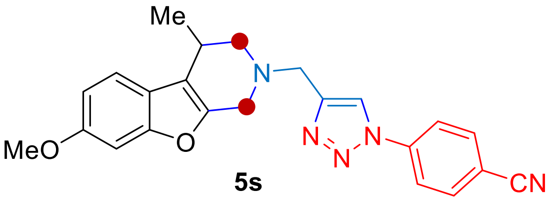

5s

4-(4-((7-methoxy-4-methyl-3,4-dihydrobenzofuro[2,3-*c*]pyridin-2(1*H*)-yl)methyl)-1*H*-1,2,3-triazol-1-yl)benzonitrile

5s: Pale yellow solid, m. p. 203.5 – 209.4 °C (48 mg, 60% yield for two steps).

TLC: Rf = 0.1 (Petroleum ether : EtOAc = 1 : 1).

^{1}H NMR (500 MHz, $CDCl_3$) δ 8.08 (s, 1H), 7.91 (d, J = 8.5 Hz, 2H), 7.82 (d, J = 8.5 Hz, 2H), 7.35 (d, J = 8.5 Hz, 1H), 6.95 (d, J = 2.0 Hz, 1H), 6.82 (dd, J_1 = 8.5 Hz, J_2 = 2.5 Hz, 1H), 4.02 (s, 2H), 3.82 (s, 3H), 3.80 – 3.63 (m, 2H), 3.15 – 3.07 (m, 1H), 3.08 – 3.01 (m, 1H), 2.51 – 2.43 (m, 1H), 1.31 (d, J = 6.5 Hz, 3H).

^{13}C NMR (125 MHz, $CDCl_3$) δ 157.2, 155.8, 149.4, 146.7, 139.9, 133.9, 120.9, 120.5, 120.5, 119.5, 117.8, 115.8, 112.3, 110.8, 96.5, 58.5, 55.8, 52.4, 50.3, 27.8, 18.0.

HRMS (ESI-TOF) m/z: $[M+H]^+$ Calcd for $C_{23}H_{22}N_5O_2$ 400.1771; Found: 400.1773.

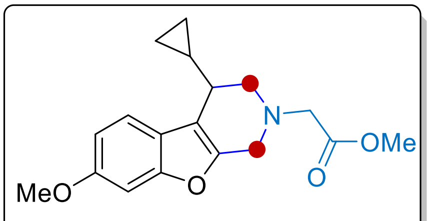

5t

4-cyclopropyl-7-methoxy-2-(prop-2-yn-1-yl)-1,2,3,4-tetrahydrobenzofuro[2,3-*c*]pyridine

5t: Pale yellow solid, m. p.114.5– 116.2 °C (55 mg, 87% yield).

TLC: Rf = 0.3 (Petroleum ether : EtOAc = 5 : 1).

^{1}H NMR (600 MHz, $CDCl_3$) δ 7.56 (d, *J* = 8.0 Hz, 1H), 6.98 (d, *J* = 2.4 Hz, 1H), 6.84 (dd, J_1 = 8.0 Hz, J_2 = 2.4 Hz, 1H), 3.89 – 3.78 (m, 5H), 3.75 (s, 3H), 3.57 – 3.43 (m, 2H), 3.13 – 3.01 (m, 1H), 2.83 – 2.70 (m, 1H), 2.23 – 2.09 (m, 1H), 0.89 – 0.74 (m, 2H), 0.62 – 0.50 (m, 2H), 0.32 – 0.22 (m, 1H).

^{13}C NMR (125 MHz, $CDCl_3$) δ 170.9, 157.2, 155.8, 149.6, 121.3, 120.6, 115.0, 110.8, 96.3, 58.1, 56.6, 55.7, 51.8, 49.7, 39.4, 13.9, 6.1, 3.2.

HRMS (ESI-TOF) m/z: $[M+H]^+$ Calcd for $C_{18}H_{22}NO_4$ 316.1549; Found: 316.1551.

Gram-Scale Reaction

An oven-dried three-necked flask equipped with a magnetic stirrer bar was charged with **1a** (5 mmol, 1 equiv.), **2** (20 mmol, 4 equiv.) and **3a** (10 mmol, 2 equiv.), then evacuated and purged with argon three times. 20 mL AcOH was added to the flask by a syringe, and the solution was stirred at 60 °C for 3 hours to be completed. Then the solvent was evaporated, while ethyl acetate (30 mL) and saturated aqueous $NaHCO_3$ (30 mL) were added. The aqueous layer was extracted with ethyl acetate (three times), and the combined organic layer was washed with brine, dried over Na_2SO_4 and concentrated. The crude product was purified by column chromatography on silica gel eluted with petroleum ether/ethyl acetate (v/v, 10:1 to 5:1) to afford the compound **4a** (1079 mg, 78% yield).

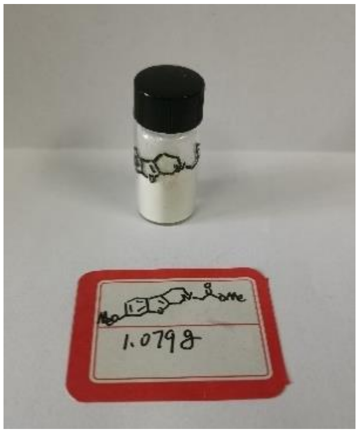

Mechanistic Investigation

Synthesis of 6 and 7

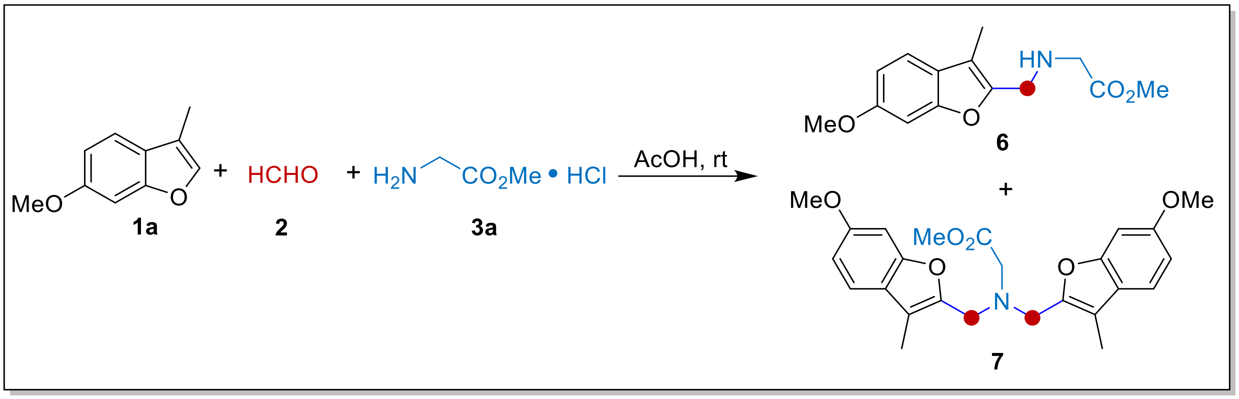

An oven-dried three-necked flask equipped with a magnetic stirrer bar was charged with **1a** (5 mmol, 1 equiv.), **2** (5 mmol, 1 equiv.) and **3a** (5 mmol, 1 equiv.), then evacuated and purged with argon three times. 20 mL anhydrous acetic acid was added by a syringe, and the mixture was stirred at rt. Then the solvent was evaporated, while ethyl acetate (30 mL) and saturated aqueous $NaHCO_3$ (30 mL) were added. The aqueous layer was extracted with ethyl acetate (three times), and the combined organic layer was washed with brine, dried over Na_2SO_4 and concentrated. The crude product was purified by column chromatography on silica gel eluted with petroleum ether/ethyl acetate (v/v, 10:1 to 5:1) to afford the compound **6** (370 mg, 28% yield) and **7** (765 mg, 70% yield).

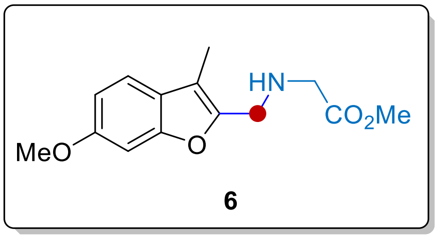

methyl ((6-methoxy-3-methylbenzofuran-2-yl)methyl)glycinate

6: Pale yellow solid, m. p. 75.2 – 78.4 °C (370 mg, 28% yield in 5 mmol scale).

TLC: Rf = 0.3 (Petroleum ether : EtOAc = 2 : 1).

^{1}H NMR (500 MHz, $CDCl_3$) δ 7.30 (d, J = 8.5 Hz, 1H), 6.92 (d, J = 2.0 Hz, 1H), 6.84 (dd, J_1 = 8.5 Hz, J_2 = 2.0 Hz, 1H), 4.04 (s, 2H), 3.83 (s, 3H), 3.72 (s, 1H), 3.60 (s, 3H), 3.52 (s, 2H), 2.16 (s, 3H).

^{13}C NMR (100 MHz, $CDCl_3$) δ 172.7, 158.0, 155.2, 149.4, 123.2, 119.4, 112.8, 111.0, 95.9, 55.8, 51.9, 49.4, 43.5, 7.9.

HRMS (ESI-TOF) m/z: $[M+H]^+$ Calcd for $C_{14}H_{18}NO_4$ 264.1236; Found: 254.1240.

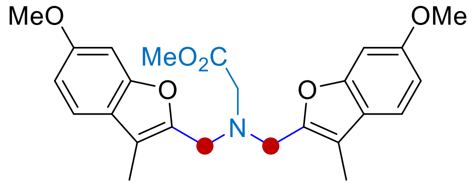

7

methyl bis((6-methoxy-3-methylbenzofuran-2-yl)methyl)glycinate

7: White solid, m. p. 83.5 – 86.4 °C (765 mg, 70% yield in 5 mmol scale).

TLC: Rf = 0.6 (Petroleum ether : EtOAc = 4 : 1).

^{1}H NMR (400 MHz, $CDCl_3$) δ 7.33 (dd, J_1 = 8.4 Hz, J_2 = 1.2 Hz, 2H), 7.01 (d, J = 2.4 Hz, 2H), 6.88 (dd, J_1 = 8.4 Hz, J_2 = 2.4 Hz, 2H), 4.08 (s, 4H), 3.86 (d, J = 2.8 Hz, 6H), 3.66 (s, 3H), 3.51 (s, 2H), 2.20 (s, 6H).

^{13}C NMR (100 MHz, $CDCl_3$) δ 171.6, 158.0, 155.3, 148.6, 123.2, 119.3, 114.0, 111.0, 96.0, 55.7, 53.5, 51.5, 48.4, 8.1.

HRMS (ESI-TOF) m/z: $[M+H]^+$ Calcd for $C_{25}H_{28}NO_6$ 438.1917; Found: 438.1915.

Control reaction of compound 6

An oven-dried Schlenk tube equipped with a magnetic stirrer bar was charged with **6** (0.2 mmol, 1 equiv.), **2** and **3a**, then evacuated and purged with argon three times. 1 mL anhydrous AcOH was added by a syringe, and the solution was stirred at 60 °C for 3 hours to be completed. Then the reaction was quenched by the saturated aqueous $NaHCO_3$. The aqueous layer was extracted with ethyl acetate (three times), and the combined organic layer was dried over Na_2SO_4 and concentrated. Purification by flash column chromatography afforded the pure product. The results are shown in **Supplementary Table 2**.

Supplementary Table 2. Control reaction of compound 6.

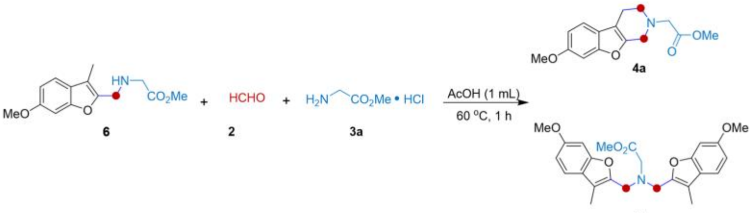

	**3a** (equiv.)	**2** (equiv.)	Yield **4a**	Yield **7**
a	1	2	93%	trace
b	0	2	54%	< 10%
c	1	0	31%	trace

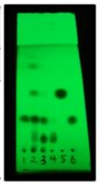

1: Reaction a
2: Reaction b
3: Reaction c
4: Compound **6**
5: Compound **7**
6: Compound **4a**

Control reaction of compound 7

An oven-dried Schlenk tube equipped with a magnetic stirrer bar was charged with **7** (0.2 mmol, 1 equiv.), **2** and **3a**, then evacuated and purged with argon three times. 1 mL anhydrous AcOH was added by a syringe, and the mixture was stirred at 60 °C. After completion, the reaction was quenched by the saturated aqueous $NaHCO_3$. The aqueous layer was extracted with ethyl acetate (three times), and the combined organic layer was dried over Na_2SO_4 and concentrated. Purification by flash column chromatography afforded the pure product. The results are shown in **Supplementary Table 3. Control reaction of 7.**

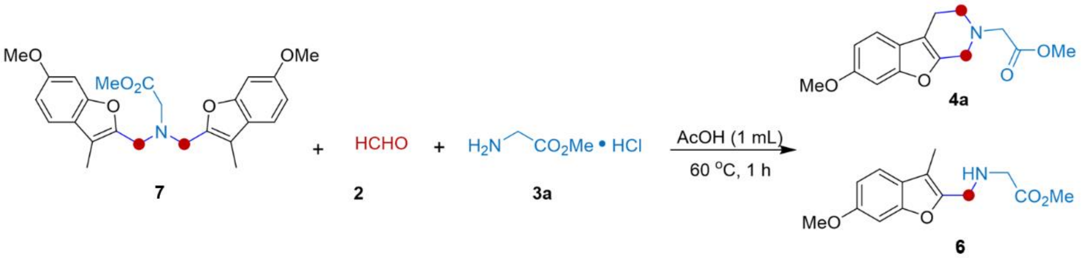

	**3a** (equiv.)	**2** (equiv.)	Yield	
			**4a**	**6**
a	1	2	69%	trace
b	0	2	< 10%	< 10%
c	1	0	trace	72%

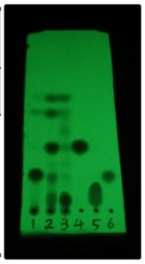

1: Reaction a
2: Reaction b
3: Reaction c
4: Compound **7**
5: Compound **6**
6: Compound **4a**

Synthesis of [D₂]-4a

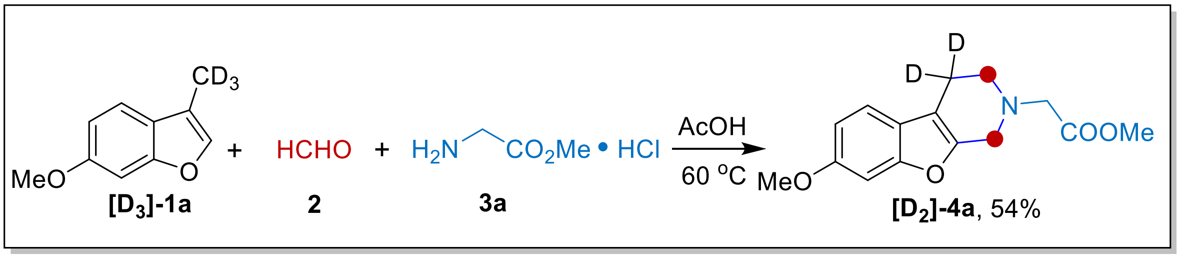

An oven-dried Schlenk tube equipped with a magnetic stirrer bar was charged with **[D₃]-1a** (33 mg, 0.2 mmol, 1 equiv.), **2** (24 mg, 0.8 mmol, 4 equiv.) and **3a** (50 mg, 0.4 mmol, 2 equiv.), then evacuated and purged with argon for three times. Then 1 mL anhydrous AcOH was added by a syringe, and the solution was stirred at 60 °C. After completion, the reaction was quenched by the saturated aqueous $NaHCO_3$. The aqueous layer was extracted with ethyl acetate (three times), and the combined organic layer was dried over Na_2SO_4 and concentrated. The crude product was

purified by column chromatography on silica gel eluted with petroleum ether/ethyl acetate (v/v, 5:1) to afford the compound **[D_2]-4a** (30 mg, 54% yield).

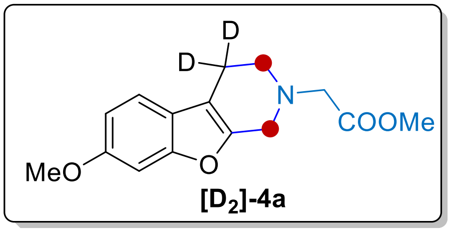

methyl 2-(7-methoxy-3,4-dihydrobenzofuro[2,3-*c*]pyridin-2(1*H*)-yl-4,4-d2)acetate

[D_2]-4a: Pale yellow solid, m. p. 72.5 – 74.4 °C (30 mg, 54% yield).

TLC: Rf = 0.3 (Petroleum ether : acetone = 4 : 1).

^{1}H NMR (500 MHz, $CDCl_3$) δ 7.28 (d, J = 8.5 Hz, 1H), 6.97 (d, J = 2.5 Hz, 1H), 6.83 (dd, J_1 = 8.5 Hz, J_2 = 2.5 Hz, 1H), 3.84 (s, 2H), 3.83 (s, 3H), 3.76 (s, 3H), 3.51 (s, 2H), 2.98 (s, 2H).

^{13}C NMR (125 MHz, $CDCl_3$) δ 170.9, 157.4, 155.6, 149.5, 121.5, 118.6, 110.8, 110.7, 96.4, 57.9, 55.8, 51.8, 49.9, 49.7, 20.2 (p, $J_{C\text{-}D}$ = 20.0 Hz).

HRMS (ESI-TOF) m/z: $[M+H]^+$ Calcd for $C_{15}H_{16}D_2NO_4$ 278.1361; Found: 278.1361.

Kinetic Isotopic Effect Experiments

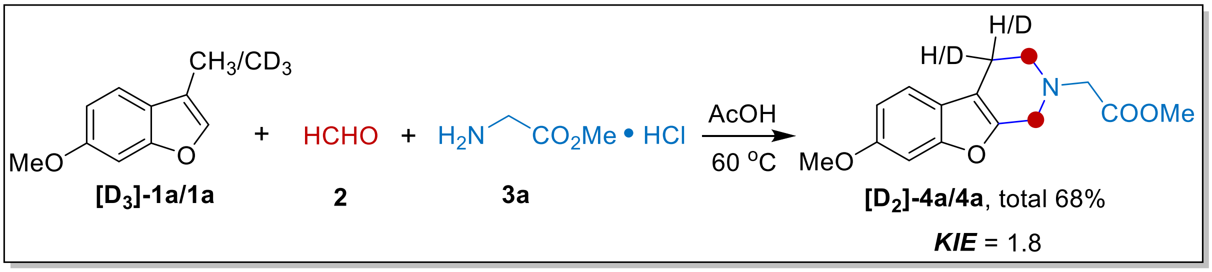

An oven-dried Schlenk tube equipped with a magnetic stirrer bar was charged with **[D_3]-1a** (16 mg, 0.1 mmol, 1 equiv.), **1a** (16 mg, 0.1 mmol, 1 equiv.) **2** (24 mg, 0.8 mmol) and **3a** (50 mg, 0.4 mmol), then evacuated and purged with argon for three times. Then 1 mL anhydrous AcOH was added by a syringe, and the solution was stirred at 60 °C for 30 minutes. The reaction was quenched by the saturated aqueous $NaHCO_3$, and the aqueous layer was extracted with ethyl acetate (three times). The combined organic layer was dried over Na_2SO_4 and concentrated. The crude product was purified by column chromatography on silica gel eluted with petroleum ether/ethyl acetate (v/v, 5:1) to afford the compounds **[D_2]-4a** and **4a** (38 mg, 68% yield, *KIE* = 1.8).

Radical-Probe Experiments

An oven-dried Schlenk tube equipped with a magnetic stirrer bar was charged with **6** (0.2 mmol, 1 equiv.), **2** (24 mg, 0.8 mmol, 4 equiv.), **3a** (50 mg, 0.4 mmol, 2 equiv.), and TEMPO (0.2 mmol, 1 equiv.), evacuated and purged with argon three times. Then 1 mL anhydrous AcOH was added by a syringe, and the solution was stirred at 60 ^{o}C for 3 hr. Upon Completion, **4a** could be formed in comparable yield (38.5 mg, 70%).

When BHT (butylated hydroxytoluene, 0.2 mmol, 1 equiv.) was used as radical scavenger, the Mannich reaction still works to give **4a** (39.6 mg, 78%).

DFT computational details

All DFT calculations were performed with the Gaussian 09 software package[10]. Geometry optimizations of all the minima and transition states were carried out at the B3LYP functional[11] with Grimme's D3 (Becke-Johnson damping) dispersion correction[12] and Def2SVP basis set[13] in gas phase. Vibrational frequencies were computed at the same level to evaluate its zero-point energy (***ZPE***) and thermal corrections at 298 K, and to check whether each optimized structure is a transition state or not. For critical transition states, IRC (intrinsic reaction coordinates) analysis was also performed at the same level of theory as geometry optimization to verify the proposed process. The single-point energies and solvent effects in acetic acid were computed at the same level of theory as geometry optimization with Def2TZVP basis set[13]. Solvation energies were evaluated by a self-consistent reaction field (SCRF) using the SMD model[14]. The 3D diagrams of molecules were generated using CYLView software[15].

Energies, Enthalpies, and Free Energy of the Calculated Structures

Zero-point correction (*ZPE*), thermal correction to enthalpy (Δ*H*), thermal correction to Gibbs free energy (Δ*G*), energies (*E*), enthalpies (*H*), Gibbs free energies (*G*) (in Hartree) and imaginary frequencies of the transition states calculated at the B3LYP-D3(BJ)/Def2-TZVP-SMD(Acetic acid)//B3LYP-D3(BJ)/ Def2-SVP level of theory

Structures	*ZPE*	*tcH*	*tcG*	*E*	*H*	*G*	Imaginary Frequency
A	0.293774	0.313611	0.244146	-899.782408	-899.468797	-898.524620	
TS-A	0.421153	0.449370	0.360746	-1262.219636	-1261.770266	-1260.386380	-152.8*i*

B	0.422895	0.451212	0.361389	-1262.227567	-1261.776355	-1260.393537	
TS-B	0.419285	0.446598	0.359062	-1262.208139	-1261.761541	-1260.375067	-1417.2*i*
C	0.424628	0.452397	0.363650	-1262.231482	-1261.779085	-1260.381827	
TS-C	0.552399	0.588899	0.478729	-1624.654112	-1624.065213	-1622.155534	-315.1*i*
C2	0.555709	0.591750	0.484997	-1624.680555	-1624.088805	-1622.192575	
TS-C2	0.554347	0.590112	0.484136	-1624.677309	-1624.087197	-1622.186314	-233.8*i*
D	0.426980	0.454471	0.368201	-1262.254818	-1261.800347	-1260.409168	
TS-D	0.424127	0.451758	0.362726	-1262.207796	-1261.756038	-1260.371207	-390.5*i*
4a-H	0.317134	0.336123	0.269817	-938.349011	-938.012888	-936.966737	
Iminium	0.125002	0.133942	0.092083	-362.423624	-362.289682	-362.331541	
Amine	0.107377	0.115499	0.076246	-323.890489	-323.774990	-323.429978	

The optimized geometries of all intermediates and transition states are shown as following:

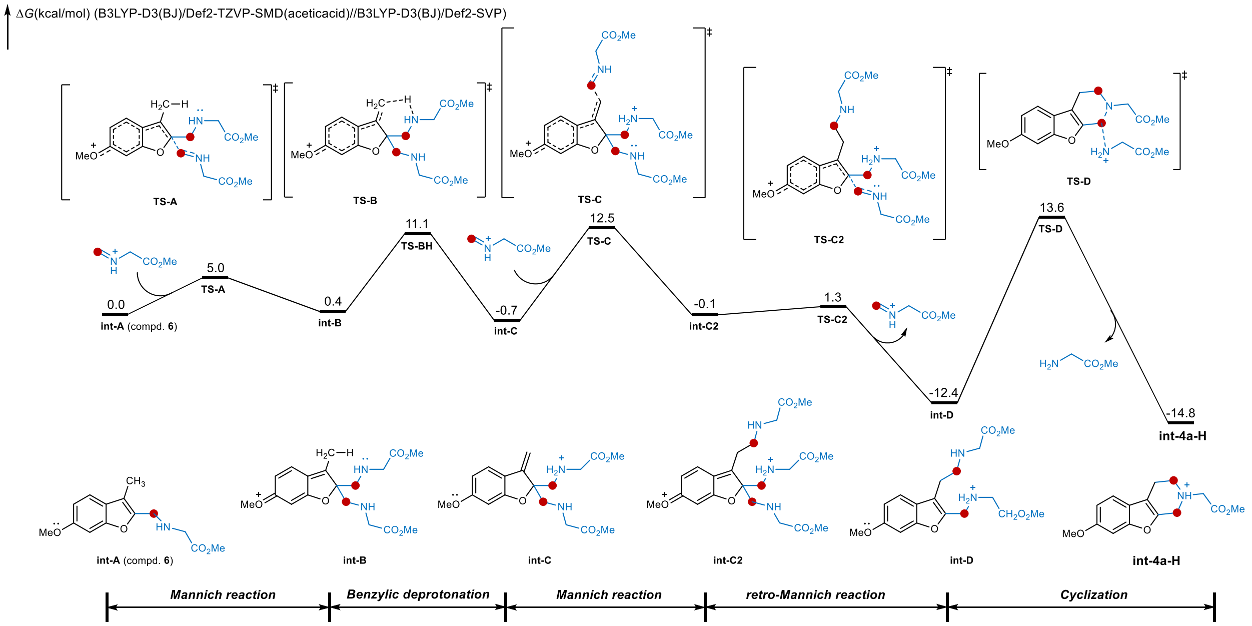

The IRC results are shown as following:

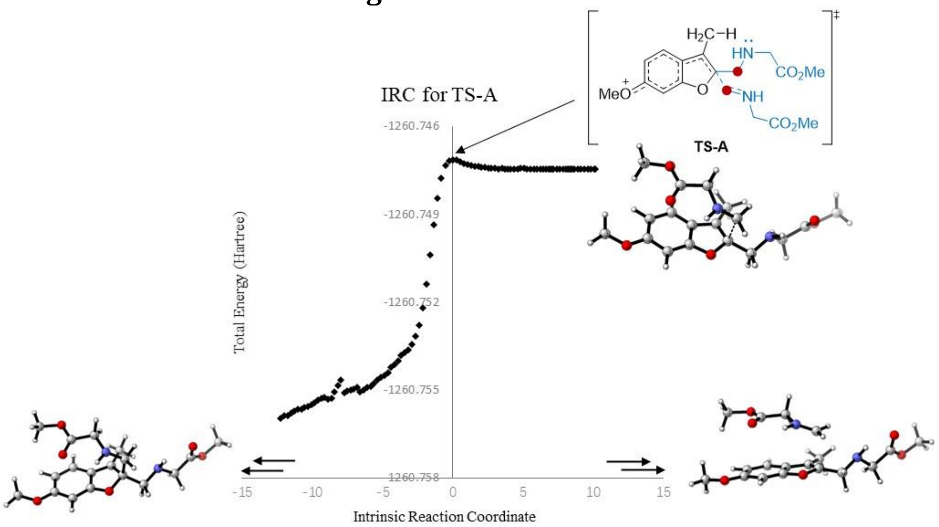

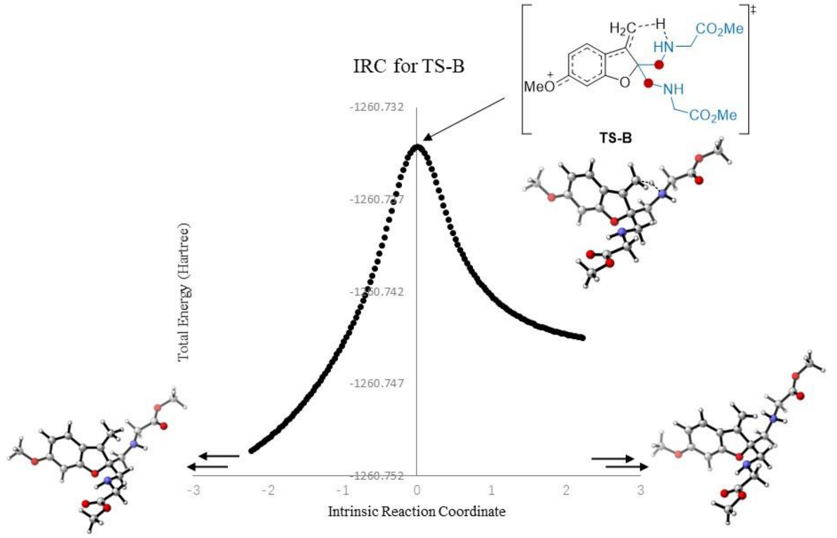

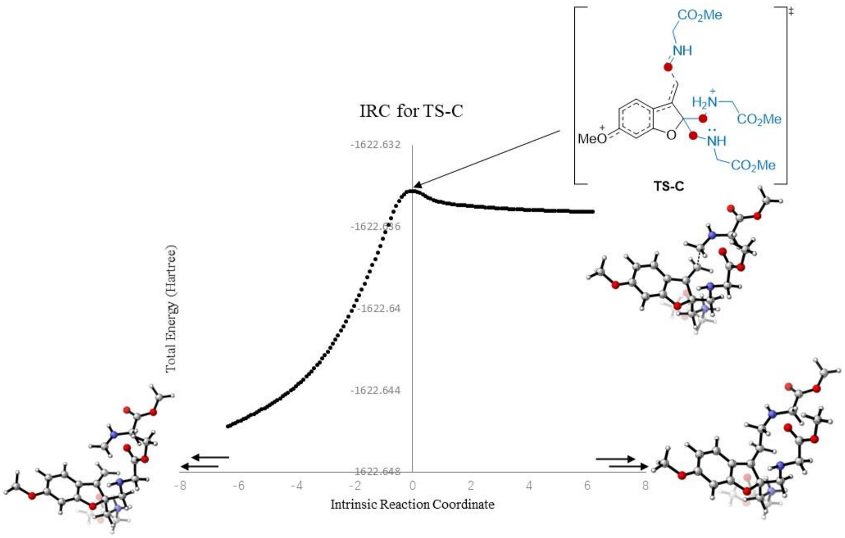

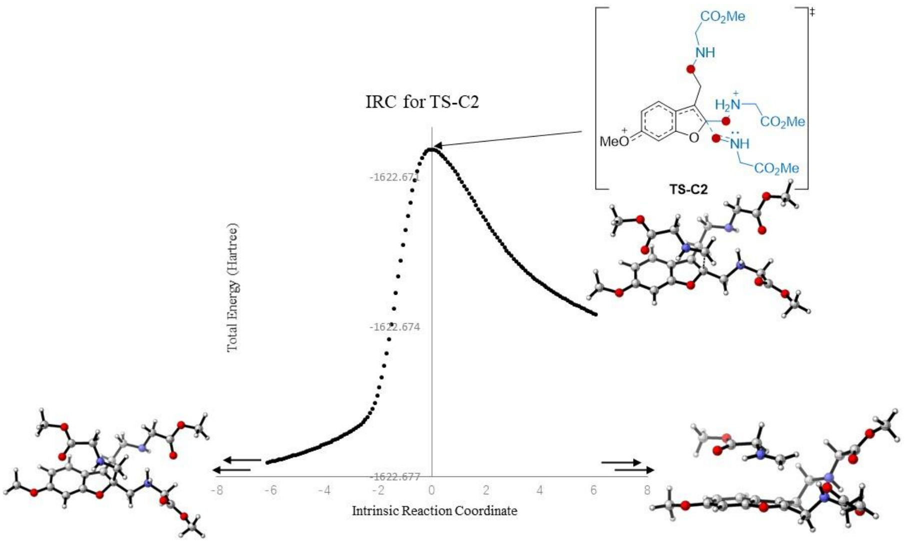

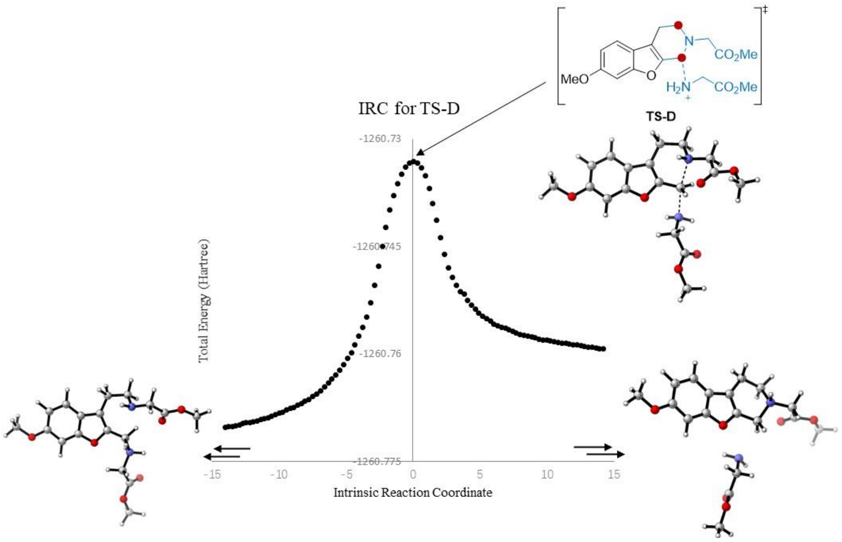

Cartesian Coordinates for the Optimized Structures

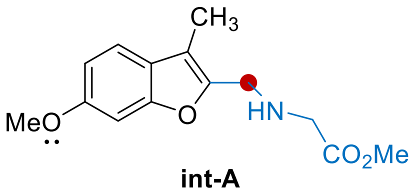

int-A

C	2.08532400	1.12249200	-0.13819300
C	1.69826900	-0.13457500	0.37355100
C	2.55342100	-1.22067000	0.47727900
C	3.87464800	-1.03084000	0.04171900
C	4.29534800	0.21557900	-0.47575800
C	3.40647600	1.28970600	-0.56697200
C	0.90004800	1.95917800	-0.07808200
C	-0.07829000	1.17258600	0.45681300

H	2.23697600	-2.18510900	0.87357900
H	5.32275800	0.35040600	-0.81112800
H	3.74838900	2.24540000	-0.97107500
O	0.38790600	-0.10055800	0.73730500
C	0.81012100	3.38129800	-0.52783200
H	-0.20292700	3.78851900	-0.40022000
H	1.50637300	4.02384600	0.03735500
H	1.07825900	3.47569900	-1.59356100
C	-1.51251700	1.39043800	0.78508400
H	-1.64960600	1.27989900	1.88816500
H	-1.77101100	2.43589700	0.54949700
C	-3.75711600	0.54615600	0.40694900
H	-4.25380600	1.44960500	0.01357600
H	-3.92759500	0.56786300	1.51202200
O	4.68971900	-2.11304800	0.15589000
C	6.03043500	-2.01141100	-0.25831500
H	6.58373200	-1.24690100	0.31711100
H	6.48955500	-2.99258400	-0.07745600
H	6.11328400	-1.77121600	-1.33398500
N	-2.36909700	0.49232500	0.03067900
H	-2.03499100	-0.46892600	0.09967800
C	-4.48779200	-0.67928600	-0.10000800
O	-3.95824000	-1.73275700	-0.35486900
O	-5.80938500	-0.46560800	-0.18494600
C	-6.60223800	-1.58570500	-0.58040200
H	-7.64170700	-1.23641900	-0.59107900
H	-6.30620100	-1.93845300	-1.57962500
H	-6.48461400	-2.41769800	0.13036100

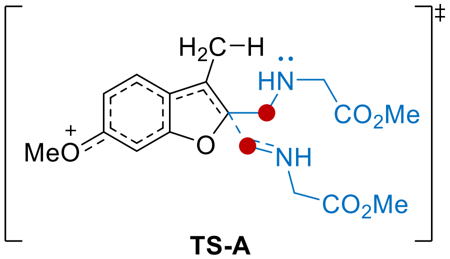

TS-A

C	0.98493100	-0.74817500	0.74926500
C	1.31189400	-1.41149500	-0.46698300
C	2.59743000	-1.79141500	-0.81417600
C	3.61224700	-1.49270200	0.10730200
C	3.31593200	-0.83513400	1.34407200
C	2.02559100	-0.46301900	1.66469400
C	-0.41665400	-0.53469900	0.72893100
C	-0.85919200	-0.98104300	-0.53823100
H	2.83481600	-2.29359600	-1.75098200
H	4.11892400	-0.63072600	2.05022700

H	1.81831800	0.03827000	2.61191500
O	0.19691800	-1.60650600	-1.20248500
C	-1.25368200	0.07093000	1.80370200
H	-2.06459500	0.67968000	1.38267000
H	-1.73209600	-0.72624800	2.39915000
H	-0.65115300	0.67606200	2.49557000
C	-0.71744200	0.85457500	-1.56213500
H	-1.02749000	0.35115300	-2.47980000
H	-1.49175900	1.38678400	-1.00538500
C	1.02354300	2.25530000	-0.55381100
H	0.55422700	2.07495300	0.42322600
H	0.80969100	3.30374400	-0.83204000
O	4.84455100	-1.85972600	-0.24551500
C	5.95236100	-1.62373900	0.61100400
H	5.83379500	-2.14605700	1.57436000
H	6.82996300	-2.02266500	0.08905800
H	6.09757100	-0.54527800	0.78849000
N	0.52161900	1.33234800	-1.54897800
H	1.23408500	0.96594900	-2.18217900
C	2.53018900	2.08844000	-0.44655900
O	3.17922100	1.45741100	-1.24502300
O	3.00867700	2.73863700	0.60334600
C	4.43493300	2.72908700	0.77584900
H	4.63620800	3.34736000	1.65718700
H	4.78699000	1.70026300	0.93485100
H	4.92990400	3.14491500	-0.11271400
C	-2.23406200	-1.46279900	-0.94703800
H	-2.20038900	-1.69271800	-2.02710100
H	-2.41886700	-2.42980800	-0.44585200
N	-3.26441300	-0.50081400	-0.61132000
C	-4.36194700	-0.95387400	0.21719600
H	-3.65348700	-0.02738500	-1.42495800
H	-4.08263300	-1.01728600	1.28192100
H	-4.75054300	-1.95696700	-0.06103800
C	-5.52070500	0.01705200	0.06890000
O	-5.60650700	0.81082900	-0.83803300
O	-6.42395200	-0.14713200	1.02958300
C	-7.60720600	0.66198100	0.94729600
H	-8.22928000	0.37471300	1.80215400
H	-7.34638200	1.72873500	1.00010100
H	-8.13650700	0.47169300	0.00260100

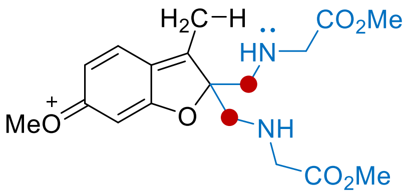

int-B

C	0.97749100	-1.45184600	0.67061400
C	1.37747200	-1.48399100	-0.71221700
C	2.47058500	-2.20178400	-1.15398700
C	3.20470900	-2.90895700	-0.18119600
C	2.82879000	-2.89429800	1.21266100
C	1.73846700	-2.18119300	1.63344800
C	-0.12285000	-0.62466600	0.75982900
C	-0.46612400	-0.16424000	-0.63062000
H	2.77920900	-2.22510400	-2.19821200
H	3.42129700	-3.45313000	1.93486000
H	1.45719800	-2.16615200	2.68774900
O	0.57691700	-0.74284500	-1.46931500
C	-0.89215500	-0.21130500	1.94181600
H	-0.68232600	0.85817100	2.12416300
H	-1.96933300	-0.26925600	1.70416100
H	-0.63646400	-0.78236000	2.84314700
C	-0.39791400	1.35748800	-0.76695100
H	-0.54417400	1.61945200	-1.83738900
H	-1.25541600	1.77188900	-0.21195000
C	0.97815000	3.28142600	-0.15052100
H	0.42307100	3.73393800	0.68742800
H	0.61716800	3.78942000	-1.07232800
O	4.24885100	-3.57769100	-0.62796300
C	5.10005100	-4.33012300	0.24105900
H	4.53941300	-5.13908900	0.73387400
H	5.87421800	-4.76066900	-0.40356400
H	5.56798700	-3.67578000	0.99224900
N	0.82875300	1.84150600	-0.18566000
H	1.64880300	1.45507100	-0.65635200
C	2.45285500	3.62119900	-0.02346100
O	3.33685000	2.84131200	-0.29049900
O	2.63583200	4.87664300	0.37163700
C	3.99235400	5.33728800	0.45120200
H	3.93789100	6.38299700	0.77354400
H	4.55844000	4.73819000	1.17887400
H	4.48203300	5.26059100	-0.53035000
C	-1.80807500	-0.75495300	-1.15291400
H	-1.81747300	-0.56872300	-2.24566500
H	-1.76396500	-1.84722000	-1.01933600
N	-2.93923200	-0.22835300	-0.44354300

C	-4.14415400	-1.03506300	-0.45670300
H	-3.18637700	0.72236000	-0.71942100
H	-4.07171000	-1.89087300	0.23424400
H	-4.38698200	-1.45728100	-1.45531400
C	-5.32058500	-0.16053500	-0.05792600
O	-5.28509900	1.04667000	-0.09060400
O	-6.37808500	-0.88748100	0.28379500
C	-7.57884100	-0.16851300	0.60603000
H	-8.33046800	-0.92886700	0.84501300
H	-7.40950500	0.49213700	1.46828400
H	-7.90265100	0.43973900	-0.25067400

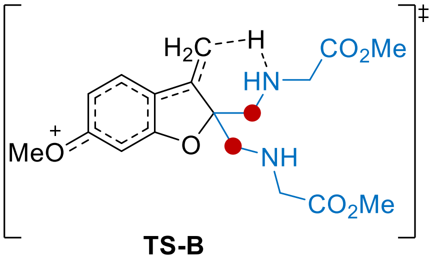

TS-B

C	-0.45596800	1.50465000	0.60148100
C	-1.01181000	1.59846000	-0.70428200
C	-1.84158400	2.63087800	-1.09232500
C	-2.14164700	3.61210700	-0.12448100
C	-1.60224000	3.53921300	1.19605400
C	-0.77208700	2.49546600	1.55606200
C	0.28960200	0.30349000	0.65725600
C	0.28349400	-0.25772000	-0.75044300
H	-2.27047400	2.69822400	-2.09127800
H	-1.85271300	4.30357000	1.92930200
H	-0.37206100	2.43614000	2.57006300
O	-0.63983400	0.56409900	-1.49023800
C	1.14505500	-0.23027400	1.62457400
H	1.19049700	-1.32487700	1.69613000
H	2.21451800	-0.24087200	0.71810500
H	1.24290100	0.28675400	2.58404100
C	-0.15777500	-1.71145000	-0.87047800
H	-0.15062900	-1.98637500	-1.94955500
H	0.60453200	-2.34667300	-0.38362900
C	-1.92383200	-3.23999800	-0.15661300
H	-1.44910800	-3.82539200	0.64820400
H	-1.76363800	-3.81531700	-1.09638200
O	-2.95071500	4.58601000	-0.52893000
C	-3.35066100	5.62569700	0.35776500
H	-2.48171200	6.20687000	0.70607700
H	-4.01472500	6.27707900	-0.22197700
H	-3.90024200	5.21964500	1.22200700

N	-1.42103600	-1.88528700	-0.21098200
H	-2.14168300	-1.28793500	-0.61913100
C	-3.42552700	-3.20282100	0.06729400
O	-4.10642400	-2.23239500	-0.16377000
O	-3.88603400	-4.37244500	0.50260900
C	-5.30573600	-4.47907700	0.67745900
H	-5.49129300	-5.50003400	1.02930800
H	-5.65718700	-3.74586900	1.41758800
H	-5.82518600	-4.29875200	-0.27491200
C	1.69401000	0.00929100	-1.37959000
H	1.74601400	-0.49616700	-2.35582700
H	1.77921500	1.09138100	-1.54629400
N	2.77581600	-0.41433200	-0.49220400
C	4.04635700	0.31229700	-0.58191100
H	2.98923600	-1.41813300	-0.54705800
H	3.98930800	1.26622700	-0.03587600
H	4.31197800	0.54256600	-1.62815300
C	5.13197300	-0.57897000	0.00722300
O	4.94271100	-1.75156400	0.23396800
O	6.25741300	0.08144900	0.19476200
C	7.38172600	-0.66795400	0.69853600
H	8.20835900	0.04663300	0.76871600
H	7.14327300	-1.08656300	1.68609300
H	7.63033600	-1.48574200	0.00805100

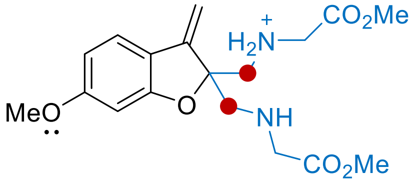

int-C

C	0.78049900	-1.56979800	0.72356800
C	1.24623400	-1.53313800	-0.60552300
C	2.20946300	-2.39290500	-1.09395900
C	2.74298900	-3.34085600	-0.19533400
C	2.29649200	-3.39794800	1.14834300
C	1.32413300	-2.51273100	1.60422300
C	-0.17515900	-0.49286600	0.88515200
C	-0.35903800	0.07753200	-0.52719900
H	2.56800200	-2.35070800	-2.12172400
H	2.71593600	-4.12994200	1.83603700
H	0.99526900	-2.55761300	2.64459500
O	0.64945600	-0.54526400	-1.33947600
C	-0.84285200	-0.06684700	1.98214800
H	-1.40304300	0.87269600	1.98578800
H	-2.48821800	-0.29257600	0.75261100

H	-0.69743500	-0.54672500	2.95215000
C	-0.19574700	1.59204100	-0.63892600
H	-0.39773800	1.88658300	-1.69580500
H	-0.97847600	2.07978800	-0.02696200
C	1.35165600	3.39259800	-0.07568800
H	0.89519800	3.85020900	0.81808100
H	0.96682900	3.96654900	-0.94964300
O	3.67619100	-4.15444900	-0.70499500
C	4.29816700	-5.13055600	0.11495700
H	3.56413200	-5.85737900	0.50151700
H	5.02007400	-5.65222300	-0.52476600
H	4.83222000	-4.66368000	0.95930700
N	1.09393700	1.97382300	-0.14928100
H	1.84368300	1.52172400	-0.67385300
C	2.85212800	3.62497500	-0.04581200
O	3.65827800	2.81007100	-0.42445600
O	3.15124900	4.84166000	0.40486900
C	4.53992500	5.19913800	0.40883000
H	4.58799000	6.22269800	0.79712000
H	5.11062900	4.51495700	1.05319800
H	4.95244000	5.15279700	-0.60955300
C	-1.69110700	-0.43580900	-1.12220900
H	-1.88173800	0.00239500	-2.11140000
H	-1.63511100	-1.52834600	-1.21236000
N	-2.83723200	-0.11794500	-0.22409400
C	-4.11013000	-0.84350700	-0.46879500
H	-3.09165800	0.88923300	-0.23974100
H	-4.13282800	-1.78515200	0.09582600
H	-4.20223500	-1.07837000	-1.53972300
C	-5.23898200	0.09994600	-0.05825800
O	-5.01669500	1.27029600	0.15971100
O	-6.39739700	-0.50862000	-0.01373400
C	-7.55902100	0.29093800	0.31350200
H	-8.40862900	-0.39834900	0.28246000
H	-7.44109500	0.72415500	1.31589400
H	-7.67767200	1.09533900	-0.42488600

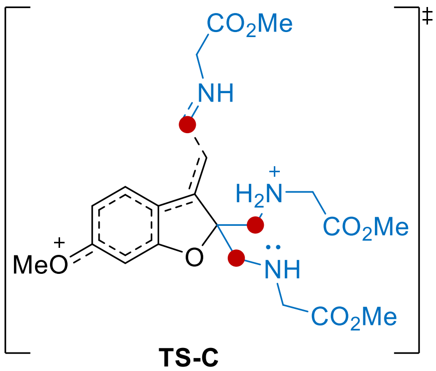

TS-C

C	1.70465900	1.01862000	0.66144200
C	2.24596300	1.86836600	-0.35157700
C	2.98295700	2.99426800	-0.07823700
C	3.19765600	3.30658200	1.28836900
C	2.67271200	2.47208300	2.33012800
C	1.93746300	1.34866300	2.02355500
C	0.95664300	0.01137400	0.02993100
C	1.21404100	0.17929000	-1.46918500
H	3.38602100	3.64374500	-0.85476700
H	2.85902400	2.72621000	3.37225900
H	1.55354000	0.71353300	2.82427300
O	1.91547400	1.43459600	-1.59265600
C	0.04091600	-0.88947300	0.62559800
H	-0.44063100	-1.61986200	-0.03440100
H	1.56419300	-2.42200500	-0.81287800
C	-0.02053500	0.23282900	-2.36891800
H	0.33165500	0.42091500	-3.40653800
H	-0.50237600	-0.76320600	-2.37575800
C	-2.20044100	1.30431200	-2.63803300
H	-2.53235500	0.29565800	-2.94145900
H	-2.10640500	1.88981300	-3.57050800
O	3.90051200	4.39431200	1.51646500
C	4.18906700	4.85145300	2.84434200
H	4.79114600	4.10758900	3.38784600
H	4.76672900	5.77372000	2.72017900
H	3.25820500	5.06741400	3.39020400
N	-0.97204600	1.19013100	-1.87020300
H	-0.53163500	2.10359100	-1.76706300
C	-3.33639600	1.87990400	-1.79713600
O	-3.40595100	1.77857700	-0.59192200
O	-4.24532500	2.45307000	-2.56611300
C	-5.42495200	2.97712700	-1.92681700
H	-6.01810600	3.43654600	-2.72437500
H	-5.98813700	2.16445200	-1.44632700
H	-5.14572300	3.72267200	-1.16975600
C	2.26462000	-0.84575900	-1.96501500

H	2.49869300	-0.66372500	-3.02255300
H	3.17868200	-0.72963000	-1.36902300
N	1.83290800	-2.27289900	-1.81672000
C	2.92386000	-3.28011800	-2.02445400
H	1.02425200	-2.47533900	-2.41405300
H	3.70455200	-2.88019700	-2.68495200
H	2.50793900	-4.18799100	-2.48501500
C	3.45422900	-3.59964200	-0.62240600
O	2.86136500	-3.18079500	0.35195700
O	4.52195300	-4.33859800	-0.65698900
C	5.10400100	-4.77382600	0.60308500
H	5.96668000	-5.38771000	0.32731600
H	5.41330300	-3.89616400	1.18562500
H	4.36482300	-5.35970600	1.16494100
H	0.35300300	-1.32211400	1.58094200
C	-1.38628600	0.14848300	1.25579100
H	-0.83194700	0.71119300	2.01659000
H	-1.64537500	0.72730800	0.36250100
N	-2.38923900	-0.61079000	1.73945100
C	-3.53135000	-1.04500800	0.96960500
H	-2.35836800	-1.01433000	2.67838700
H	-3.24739700	-1.69946100	0.12229800
H	-4.06971400	-0.18433700	0.54264500
C	-4.44293400	-1.84215300	1.89345000
O	-4.12741500	-2.12830100	3.02404100
O	-5.57283200	-2.16876200	1.29274200
C	-6.52738000	-2.92751500	2.06373100
H	-7.37942300	-3.09990200	1.39790300
H	-6.08375200	-3.88027100	2.38446800
H	-6.83576500	-2.35546100	2.94965900

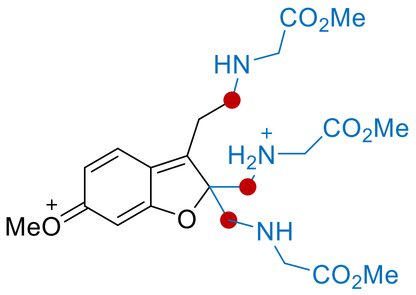

int-C2

C	-2.14413000	0.65910500	1.10688800
C	-2.22436100	1.60301200	0.01037000
C	-3.40309800	2.15227500	-0.42595700
C	-4.57828600	1.72862500	0.23124500
C	-4.53466200	0.81229200	1.35707800
C	-3.35331800	0.29345000	1.79122300

C	-0.82228300	0.33758500	1.28059900
C	-0.07333600	0.96129600	0.13365600
H	-3.46205700	2.86331100	-1.24890400
H	-5.46391200	0.53153500	1.85008700
H	-3.33066500	-0.40079600	2.63279100
O	-1.00501600	1.89526400	-0.44442100
C	-0.25178600	-0.47343700	2.40220200
H	0.42655600	0.16932600	2.99473100
H	2.31244300	0.01703400	0.93500000
C	0.17294500	-0.09091800	-1.06431600
H	0.78646700	0.46413900	-1.79465800
H	0.77804400	-0.92488800	-0.68206900
C	-1.52780700	-1.87990900	-1.52663700
H	-1.08119600	-2.37045700	-0.64631000
H	-1.27281400	-2.52493200	-2.38822800
O	-5.69667200	2.22441000	-0.22597700
C	-6.98480100	1.89118000	0.31914600
H	-7.05350600	2.21354900	1.36845000
H	-7.70955500	2.44573000	-0.28616600
H	-7.16949300	0.81107100	0.22714500
N	-1.03537600	-0.53505600	-1.63834900
H	-1.37875500	-0.00530700	-2.43009300
C	-3.04500400	-1.93641100	-1.33919100
O	-3.74039600	-0.98501800	-1.07201100
O	-3.47459200	-3.17988700	-1.49764600
C	-4.88600000	-3.41926800	-1.35828300
H	-5.02927700	-4.49012400	-1.53720200
H	-5.22052200	-3.14914100	-0.34632400
H	-5.44497200	-2.82698800	-2.09621900
C	1.18823300	1.76626000	0.47023900
H	1.28335100	2.56922400	-0.27302800
H	1.10854500	2.22543500	1.46491300
N	2.42648300	0.94953200	0.42364000
C	3.66106500	1.68724500	0.80364000
H	2.61856000	0.69446100	-0.56384900
H	4.38794600	0.97106900	1.20587500
H	3.44789000	2.46131900	1.55241300
C	4.21448500	2.28565100	-0.48974600
O	3.72860900	1.98681600	-1.56082200
O	5.22333700	3.08545600	-0.27250200
C	5.87544200	3.68135300	-1.42239300
H	6.69235600	4.28554000	-1.01592200
H	6.25934400	2.89094700	-2.08090100
H	5.15911700	4.30729800	-1.97112400
H	-1.07324100	-0.72940200	3.08514400
C	0.49625100	-1.77090700	2.04006800

H	0.40948400	-2.47114100	2.88897400
H	0.00246100	-2.26373600	1.18973200
N	1.90211700	-1.53281300	1.68489400
C	2.54145400	-2.68185700	1.03441700
H	2.41758200	-1.33046200	2.54557100
H	1.89037300	-3.04595600	0.22142800
H	2.69756800	-3.54213900	1.70800600
C	3.86549600	-2.29056300	0.39398000
O	4.18473500	-1.13786100	0.17413600
O	4.57643100	-3.34915400	0.08558000
C	5.83892900	-3.14940000	-0.58834500
H	6.51974000	-2.57784300	0.05705000
H	6.23537600	-4.15208800	-0.77706200
H	5.68011700	-2.60737300	-1.53053700

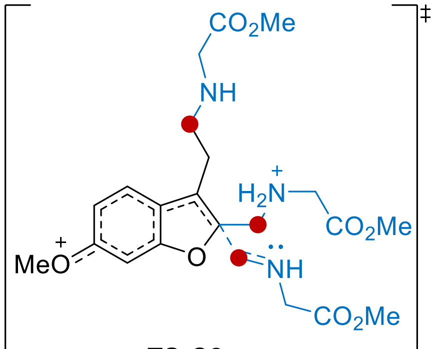

TS-C2

C	2.08958200	-0.38405500	1.19618900
C	2.15453500	-1.51511400	0.31740800
C	3.33134200	-2.08130700	-0.12571600
C	4.52307300	-1.47877700	0.31616700
C	4.49660400	-0.33982400	1.20132400
C	3.31148700	0.19842700	1.63897200
C	0.73124500	-0.15191000	1.45887300
C	0.00377800	-1.03378900	0.59354900
H	3.36534500	-2.94321800	-0.79075000
H	5.43650300	0.09478500	1.53856500
H	3.31514700	1.05543300	2.31497100
O	0.91106500	-1.94671900	0.03304100
C	0.16897100	0.80164900	2.47210100
H	-0.49946900	0.24587100	3.15526300
H	-2.41674900	0.10838300	0.93945600
C	-0.21814100	0.04485400	-1.14930600
H	-0.71820100	-0.78531500	-1.65773700
H	-0.85723900	0.86899300	-0.82364500
C	1.64216800	1.64452000	-1.41105400
H	1.42620200	2.04116100	-0.40914400
H	1.30678700	2.40039500	-2.14474000

O	5.64093900	-2.02181100	-0.12021900
C	6.92875500	-1.52620600	0.25605100
H	7.06983300	-1.58747900	1.34597500
H	7.65481100	-2.17597800	-0.24459200
H	7.06365200	-0.49026800	-0.09152900
N	0.98529100	0.37154200	-1.62436000
H	1.54008800	-0.30403800	-2.15296000
C	3.14533100	1.45879900	-1.57126500
O	3.63149300	0.44614100	-2.01329200
O	3.79779000	2.53802700	-1.18193600
C	5.22879500	2.54326100	-1.35802200
H	5.56415900	3.54195800	-1.05998300
H	5.68512500	1.77437600	-0.71865000
H	5.48246200	2.34203700	-2.40754300
C	-1.31103900	-1.70165100	0.92054500
H	-1.36377000	-2.64577500	0.36232200
H	-1.37713900	-1.93532000	1.99412500
N	-2.50245500	-0.88529100	0.56513600
C	-3.80225500	-1.53900200	0.87101000
H	-2.54703900	-0.78953600	-0.46553000
H	-4.55683600	-0.76041600	1.03759200
H	-3.72709300	-2.18044800	1.75891400
C	-4.17412500	-2.33753000	-0.37896900
O	-3.52882800	-2.21128400	-1.40034600
O	-5.22022500	-3.09409000	-0.19219100
C	-5.70958900	-3.86881800	-1.31665600
H	-6.58582900	-4.40693800	-0.94226300
H	-5.98250900	-3.19456600	-2.13922900
H	-4.93158400	-4.56829600	-1.65033600
H	0.99942400	1.15425300	3.09932100
C	-0.58135900	2.03630700	1.94305900
H	-0.53694700	2.82953100	2.70991300
H	-0.06134000	2.43855400	1.06050100
N	-1.97133500	1.74438400	1.56018800
C	-2.60241200	2.84217300	0.81859900
H	-2.50736900	1.60096700	2.42030900
H	-1.90804400	3.19673100	0.03752400
H	-2.83201700	3.72395400	1.44111600
C	-3.86311100	2.38380600	0.10017800
O	-4.11080500	1.21549800	-0.13265500
O	-4.60435000	3.40415000	-0.26035500
C	-5.81092000	3.14193100	-1.01086100
H	-6.50536700	2.54569600	-0.40333800
H	-6.23929300	4.12406800	-1.23464800
H	-5.56916100	2.60024000	-1.93542600

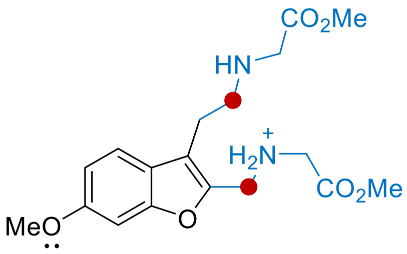

int-D

C	2.82369000	0.50954200	0.34780100
C	3.14304100	-0.47372800	-0.61101000
C	4.43397600	-0.88973400	-0.89261200
C	5.46599300	-0.27664000	-0.16162700
C	5.17714200	0.71626200	0.81134300
C	3.86770100	1.11014700	1.06765500
C	1.38157700	0.64865000	0.33483700
C	0.94644800	-0.23158800	-0.62603000
H	4.66585000	-1.64703900	-1.64061700
H	5.98793300	1.18249500	1.36865400
H	3.66686500	1.87671200	1.81868300
O	2.00054100	-0.92911700	-1.19965500
C	0.64455900	1.56446600	1.27250300
H	0.84734200	1.21004300	2.29815200
H	-0.17582000	-2.67548300	-0.68766400
H	1.10350800	2.56550500	1.20716600
C	-0.86488900	1.71942800	1.10644000
H	-1.35668400	0.72436500	1.14654000
H	-1.24444300	2.26728300	1.98506100
C	-2.58557700	2.83485600	-0.26100300
H	-2.69477000	3.56155500	-1.08484200
H	-2.98370800	3.34974200	0.63047200
O	6.71105200	-0.69526600	-0.45227400
C	7.82327900	-0.13398300	0.22016100
H	7.77638700	-0.32043600	1.30680000
H	8.71214500	-0.62871900	-0.19027800
H	7.89977300	0.95201700	0.04035000
N	-1.20653800	2.49692600	-0.07012700
H	-0.74182400	2.18969200	-0.91893100
C	-3.53362200	1.67860400	-0.57408300
O	-3.18393400	0.53495500	-0.81138300
O	-4.79993300	2.06843000	-0.58193300
C	-5.79468800	1.10017000	-0.94029800
H	-5.65453400	0.77654800	-1.98201100
H	-5.73224400	0.22137900	-0.28375000
H	-6.76176800	1.60137000	-0.82426000
C	-0.37179600	-0.63558400	-1.14134600
H	-1.16483400	0.09560200	-0.95677000

H	-0.32646200	-0.85721900	-2.21711600
N	-0.84351800	-1.92785600	-0.46913500
C	-2.25457900	-2.32836300	-0.74322500
H	-0.81492600	-1.77538400	0.56265100
H	-2.31126400	-3.39417200	-1.00369800
H	-2.66922400	-1.72702400	-1.56079400
C	-3.03194700	-2.05175400	0.53957100
O	-2.46593900	-1.68447500	1.54635300
O	-4.31388300	-2.29062900	0.40686400
C	-5.14012500	-2.09571800	1.57463400
H	-6.15720400	-2.35756400	1.26568100
H	-4.79931600	-2.74848400	2.38979200
H	-5.08471700	-1.04767300	1.89952500

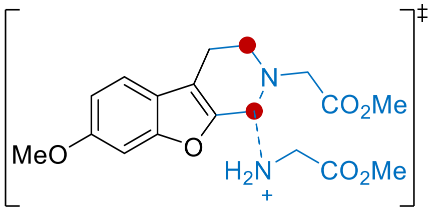

TS-D

C	2.57481600	-1.17891800	-0.38430300
C	2.37399100	0.14824600	0.04875000
C	3.38330400	0.97171000	0.51608600
C	4.67662800	0.42053400	0.55814800
C	4.91052600	-0.91351100	0.13551500
C	3.87076400	-1.71222800	-0.33277100
C	1.27474400	-1.66072700	-0.79413700
C	0.40512800	-0.62411100	-0.58772500
H	3.21346500	1.99522800	0.84858500
H	5.91727600	-1.32619500	0.17364400
H	4.07424500	-2.73514300	-0.65641300
O	1.05132200	0.48013000	-0.06907100
C	0.92558200	-3.06055200	-1.19383100
H	1.44076400	-3.34523100	-2.12599800
H	-0.26183100	1.75625700	-1.70279100
H	1.31579500	-3.74676000	-0.42122900
C	-0.57671900	-3.28538900	-1.37778000
H	-0.91022100	-2.92750600	-2.36644700
H	-0.80509100	-4.36453700	-1.33876000
C	-2.78457200	-2.85728200	-0.38051200
H	-2.98007500	-3.94456900	-0.35543300
H	-3.23979500	-2.48658800	-1.31509100
O	5.64050400	1.23727100	1.01995700
C	6.97580100	0.77523200	1.11380800
H	7.37865700	0.49700600	0.12492200
H	7.56195900	1.60969200	1.51787300

H	7.05815000	-0.08778300	1.79643700
N	-1.35936800	-2.55569100	-0.37044300
H	-0.98023400	-2.67141300	0.57288000
C	-3.48346400	-2.20759700	0.80393400
O	-2.92779100	-1.47085600	1.58663700
O	-4.75720800	-2.55828400	0.84943600
C	-5.54960600	-2.01958800	1.92439100
H	-6.55534100	-2.43113100	1.78943200
H	-5.13068000	-2.32483700	2.89328200
H	-5.56837200	-0.92205100	1.86880400
C	-1.05701700	-0.55563500	-0.74140800
H	-1.52946300	-0.74798300	-1.70187400
H	-1.68218500	-0.28899800	0.10801300
N	-1.13828400	1.50831300	-1.24219000
C	-1.37675500	2.37068800	-0.08763000
H	-1.89096500	1.66882700	-1.92129600
H	-0.43230700	2.62226900	0.41055200
H	-1.99264200	1.84196500	0.66229100
C	-2.13687100	3.62637000	-0.49653300
O	-2.68775600	3.73237400	-1.56691800
O	-2.13965800	4.53487700	0.46739400
C	-2.87306100	5.74656100	0.21592500
H	-2.76482400	6.35621100	1.11943800
H	-2.45592400	6.26898800	-0.65651200
H	-3.93141000	5.51902500	0.02506500

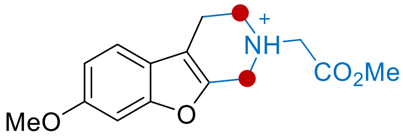

int-4a-H

C	-1.86003800	0.70319000	-0.10868300
C	-2.03055400	-0.58081600	0.46159000
C	-3.23856200	-1.25220700	0.50990600
C	-4.34731600	-0.59510000	-0.05327100
C	-4.20932900	0.69183200	-0.63368300
C	-2.97769800	1.34088800	-0.66423800
C	-0.45840100	1.01313200	0.06738500
C	0.08030800	-0.06903100	0.69330800
H	-3.35109100	-2.24062600	0.95384200
H	-5.07703600	1.18850800	-1.06463400
H	-2.89696900	2.33180200	-1.11591700
O	-0.83027000	-1.04631500	0.94780200
C	0.37041700	2.19500100	-0.32756100
H	0.49636700	2.24248400	-1.42420000
H	-0.11167600	3.14217700	-0.03632400

C	1.49955700	-0.26041000	1.08206300
H	1.69646500	-0.07371900	2.15144500
H	1.86663800	-1.26701300	0.83008700
C	3.76806900	0.65355700	0.63224500
H	3.90894900	0.38218700	1.68626000
H	4.24672100	1.62831800	0.46038000
O	-5.51034100	-1.26791700	0.00286800
C	-6.68710300	-0.69493000	-0.53700200
H	-6.58555500	-0.50697300	-1.61963400
H	-7.48803000	-1.42626700	-0.37329400
H	-6.94912400	0.24760700	-0.02647300
N	2.31716400	0.73782200	0.29565500
C	4.37978300	-0.38137100	-0.31162200
O	3.75374100	-0.79497100	-1.26221000
O	5.60178900	-0.69799200	0.03252100
C	6.31486800	-1.63145400	-0.81544000
H	7.30117300	-1.75062300	-0.35636000
H	5.77709700	-2.58849400	-0.84725100
H	6.39976200	-1.22089400	-1.83054900
C	1.73310300	2.13528200	0.36276400
H	1.66264500	2.38802900	1.43054000
H	2.46029200	2.80682300	-0.11187500
H	2.28124000	0.40411100	-0.69003600

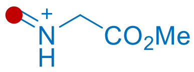

Iminium

C	-3.10709800	-0.03865900	0.00009500
H	-3.80405800	0.80688800	-0.00038500
H	-3.48685800	-1.06729600	0.00084100
C	-0.75200900	-0.78245300	0.00007100
H	-0.79751800	-1.42353300	-0.89401100
H	-0.79780200	-1.42298900	0.89452600
N	-1.85308100	0.18536300	-0.00026800
H	-1.45990600	1.15372100	-0.00100300
C	0.52592100	0.06443600	0.00004300
O	0.45497800	1.27323500	0.00022900
O	1.59872500	-0.67666600	-0.00019400
C	2.88463300	-0.00291200	0.00002800
H	3.63222200	-0.80195300	0.00167700
H	2.97435700	0.62009000	-0.89972000
H	2.97283200	0.62251400	0.89824300

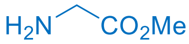

Amine

C	-1.24886100	-0.71081900	0.14326400
H	-1.14343800	-1.60767500	-0.48963100
H	-1.27278700	-1.08488200	1.19313800
N	-2.37840900	0.09786400	-0.26225200
H	-2.26727100	1.03964600	0.11479800
C	0.03002700	0.09491800	0.04491000
O	0.08426500	1.29957000	0.08975200
O	1.10909800	-0.69837400	-0.04393300
C	2.37450400	-0.03791300	-0.06256900
H	3.13209600	-0.82868400	-0.12187400
H	2.45167600	0.63150400	-0.93247300
H	2.51660900	0.56130700	0.84962900
H	-3.24893900	-0.28295000	0.10199400

X-Ray Crystallography Information

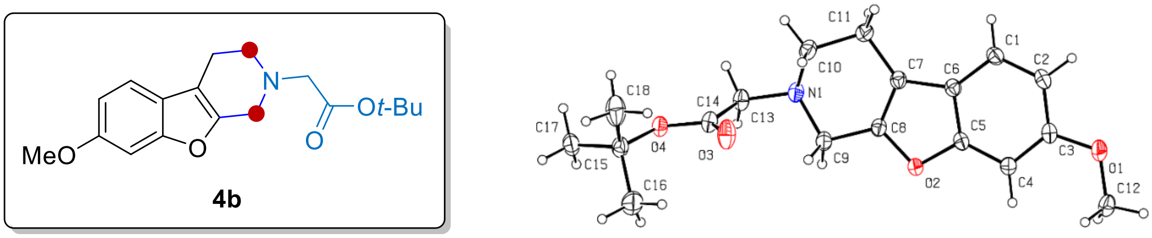

Supplementary Fig. 6. X-Ray Crystallography of 4b

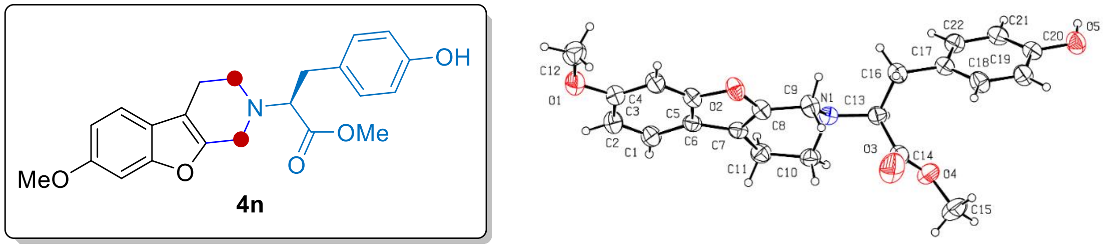

Supplementary Fig. 7. X-Ray Crystallography of 4n

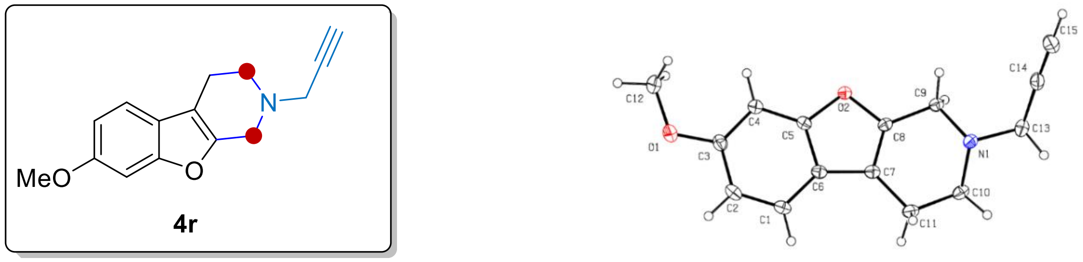

Supplementary Fig. 8. X-Ray Crystallography of 4r

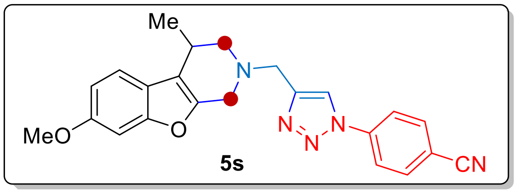

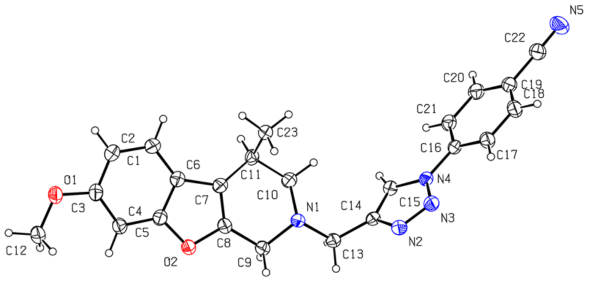

Supplementary Fig. 9. X-Ray Crystallography of 5s

Supplementary Table 4. Crystallography Data

Compound	4b	4m	4r	5s
**CCDC code**	2020613	2020614	2020615	2027076
**Empirical formula**	$C_{18}H_{23}NO_4$	$C_{22}H_{23}NO_5$	$C_{15}H_{15}NO_2$	$C_{23}H_{21}N_5O_2$
**Formula weight**	317.37	381.41	241.28	399.45
**Temperature/K**	170	299	170	170
**Crystal system**	monoclinic	orthorhombic	monoclinic	triclinic
**Space group**	$P2_1/c$	$P2_12_12_1$	$P2_1/c$	P-1
**a/Å**	16.401(7)	5.7905(4)	13.3280(4)	5.516(4)
**b/Å**	5.747(2)	14.6606(12)	14.9892(3)	13.186(9)
**c/Å**	19.628(7)	23.3541(19)	6.3197(2)	13.805(8)
**α/°**	90	90	90	101.22(3)
**β/°**	114.316(14)	90	100.5910(10)	92.90(3)
**γ/°**	90	90	90	92.60(2)
**Volume/Å^3**	1685.8(11)	1982.6(3)	1241.02(6)	982.1(11)
**Z**	4	4	4	2
**ρ_{calc}g/cm^3**	1.25	1.278	1.291	1.351
**μ/mm^{-1}**	0.088	0.091	0.086	0.09
**F(000)**	680	808	512	420
**Crystal size/mm^3**	0.45 × 0.36 × 0.33	0.36 × 0.23 × 0.05	0.49 × 0.18 × 0.06	0.48 × 0.3 × 0.2
**Radiation**	MoKα (λ = 0.71073)	MoKα (λ = 0.71073)	MoKα (λ = 0.71073)	MoKα (λ = 0.71073)
**2Θ range for data collection/°**	4.554 to 54.14	4.46 to 52.73	5.436 to 54.306	4.774 to 54.304
**Index ranges**	$-21 \le h \le 21$<br>$-6 \le k \le 7$<br>$-25 \le l \le 25$	$-7 \le h \le 7$<br>$-18 \le k \le 18$<br>$-29 \le l \le 27$	$-17 \le h \le 17$<br>$-19 \le k \le 19$<br>$-8 \le l \le 7$	$-7 \le h \le 7$<br>$-15 \le k \le 16$<br>$-17 \le l \le 17$
**Reflections collected**	23777	16045	12613	11862
**Independent reflections**	3713 [R_{int} = 0.0319, R_{sigma} = 0.0244]	4045 [R_{int} = 0.0380, R_{sigma} = 0.0303]	2727 [R_{int} = 0.0303, R_{sigma} = 0.0292]	4329 [R_{int} = 0.0341, R_{sigma} = 0.0427]
**Data/restraints/parameters**	3713/0/212	4045/0/256	2727/0 : 164	4329/5/293
**Goodness-of-fit on F^2**	1.027	1.057	1.041	1.032
**Final R indexes [I>=2σ (I)]**	R_1 = 0.0392<br>wR_2 = 0.1012	R_1 = 0.0392<br>wR_2 = 0.0940	R_1 = 0.0369<br>wR_2 = 0.0898	R_1 = 0.0463<br>wR_2 = 0.1147
**Final R indexes [all data]**	R_1 = 0.0452<br>wR_2 = 0.1068	R_1 = 0.0527<br>wR_2 = 0.1036	R_1 = 0.0418<br>wR_2 = 0.0938	R_1 = 0.0583<br>wR_2 = 0.1249

**Largest diff. peak/hole / e Å^{-3}**	0.24/-0.20	0.10/-0.15	0.23/-0.23	0.19/-0.19

2. Supplementary Figures

NMR Spectra

Supplementary Figure 10. ^{1}H-NMR spectrum of compound **1e** (400 MHz, $CDCl_3$)

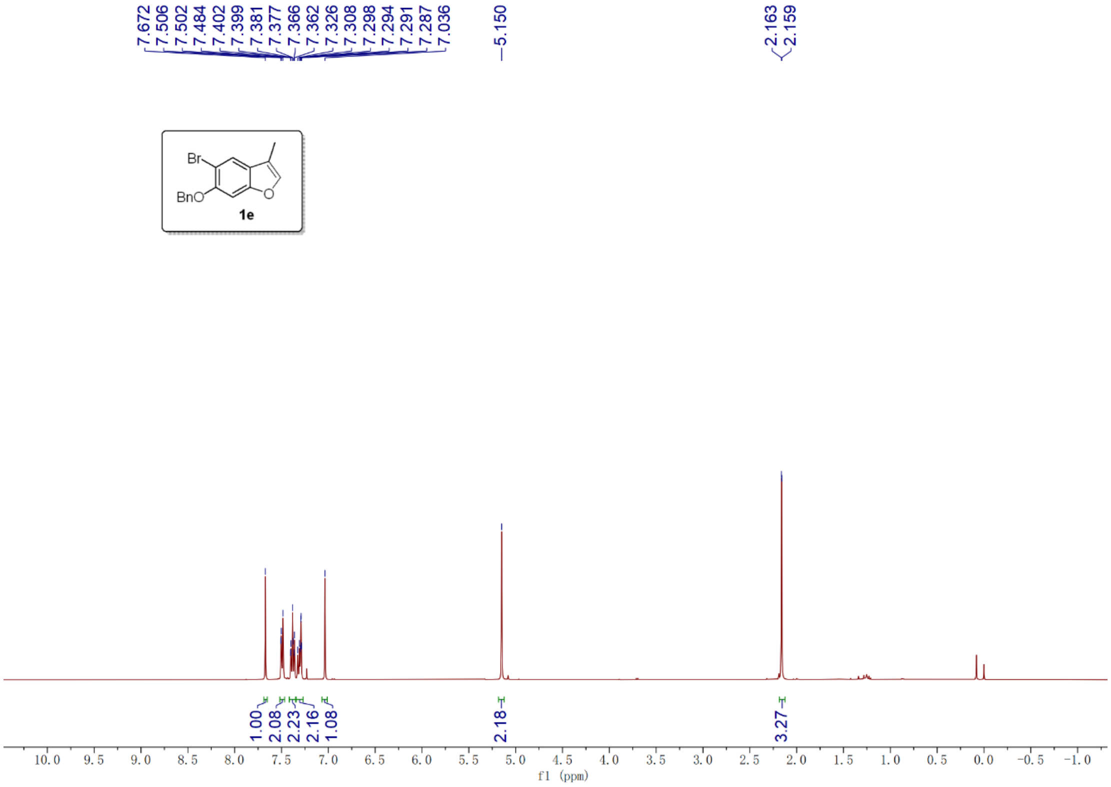

Supplementary Figure 11. ^{13}C-NMR spectrum of compound **1e** (125 MHz, $CDCl_3$)

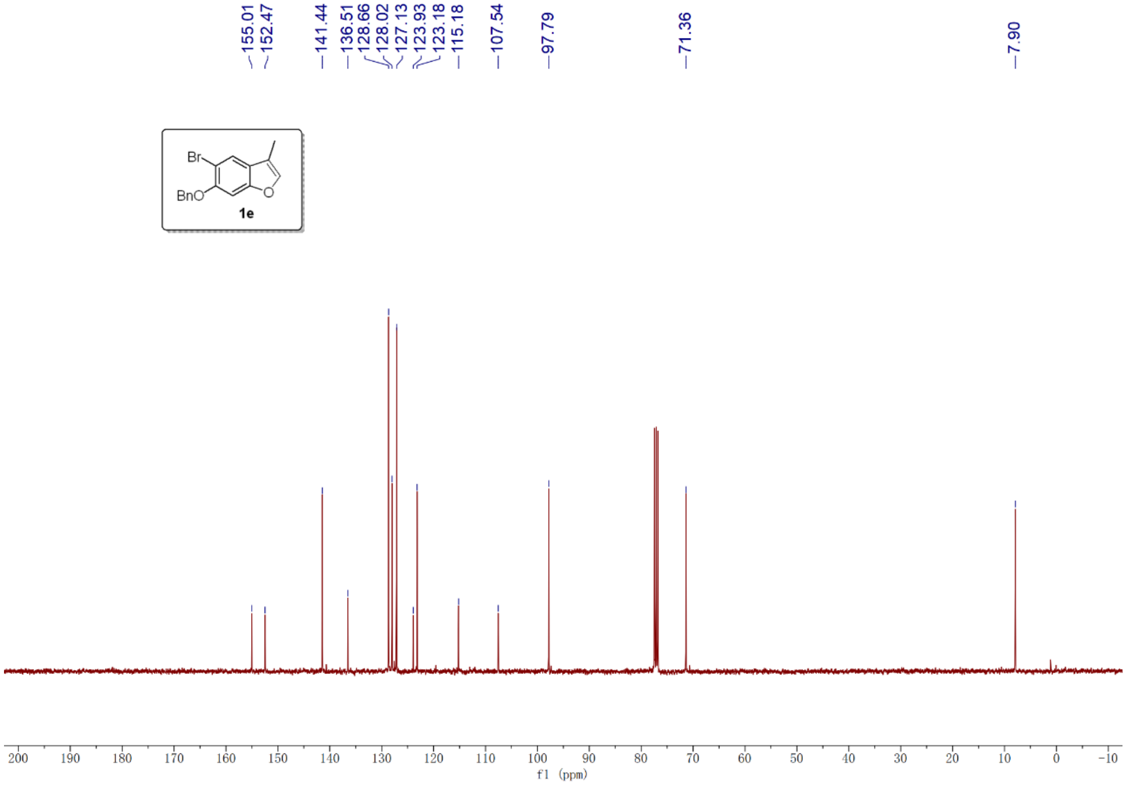

Supplementary Figure 12. ^{1}H-NMR spectrum of compound **1f** (500 MHz, $CDCl_3$)

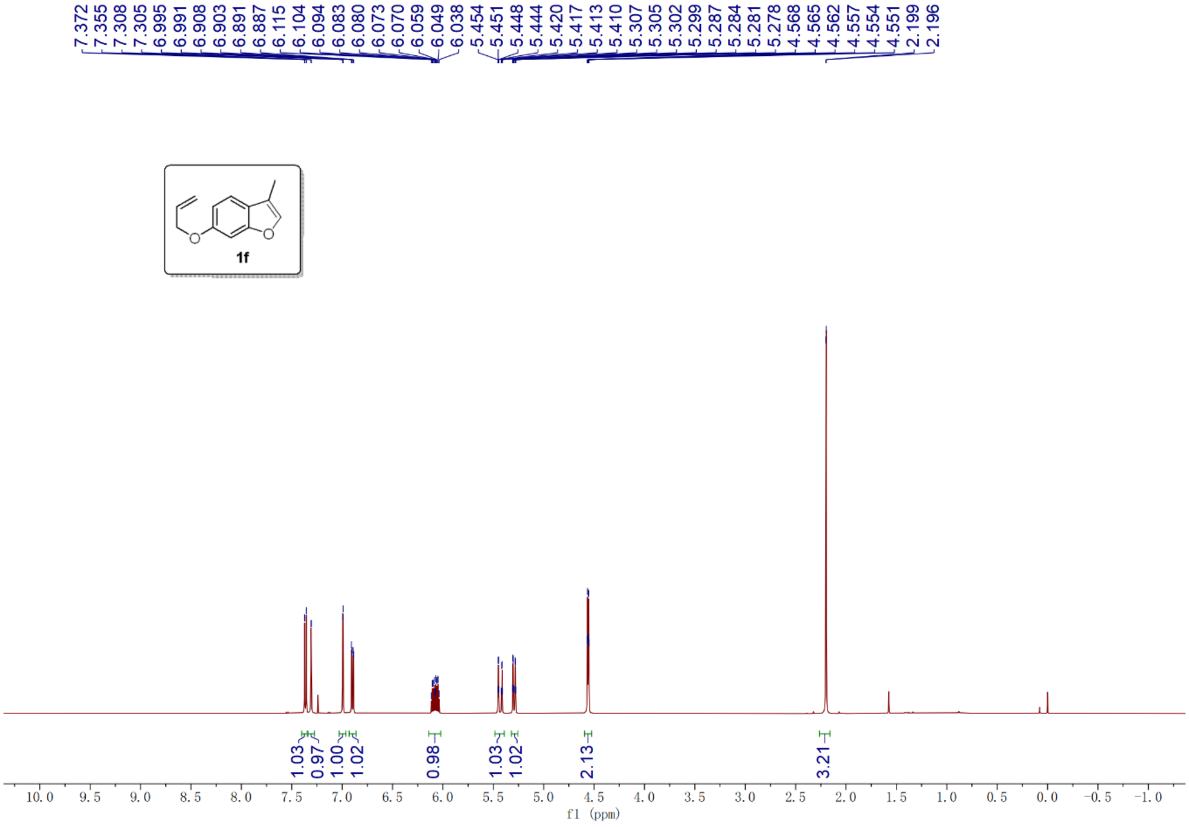

Supplementary Figure 13. ^{13}C-NMR spectrum of compound **1f** (125 MHz, $CDCl_3$)

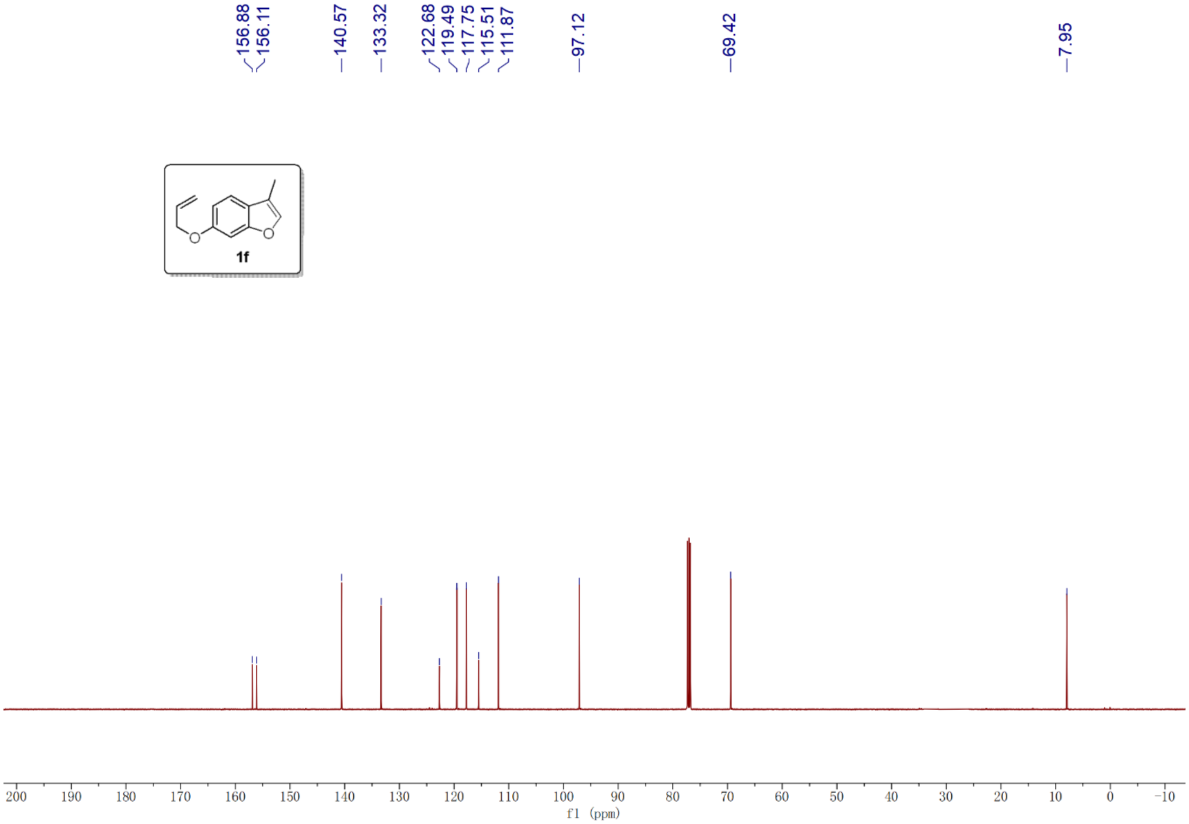

Supplementary Figure 14. ^{1}H-NMR spectrum of compound **1g** (500 MHz, $CDCl_3$)

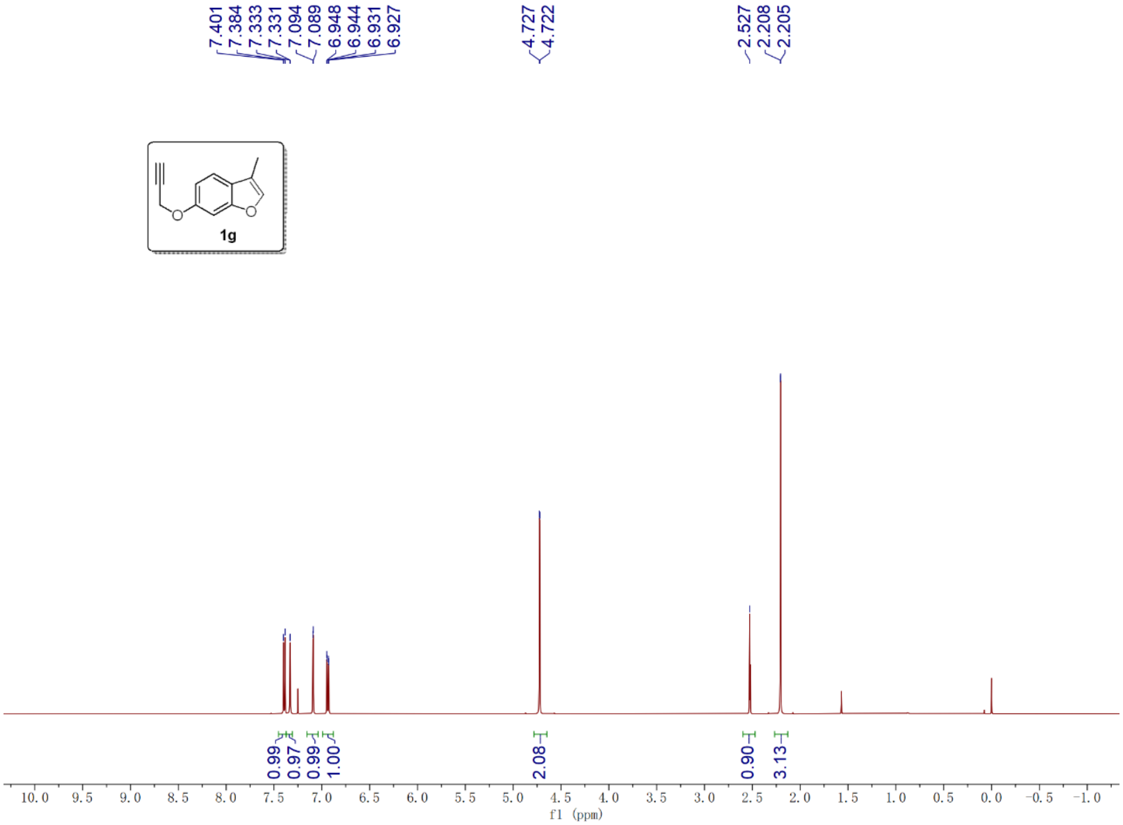

Supplementary Figure 15. ^{13}C-NMR spectrum of compound **1g** (125 MHz, $CDCl_3$)

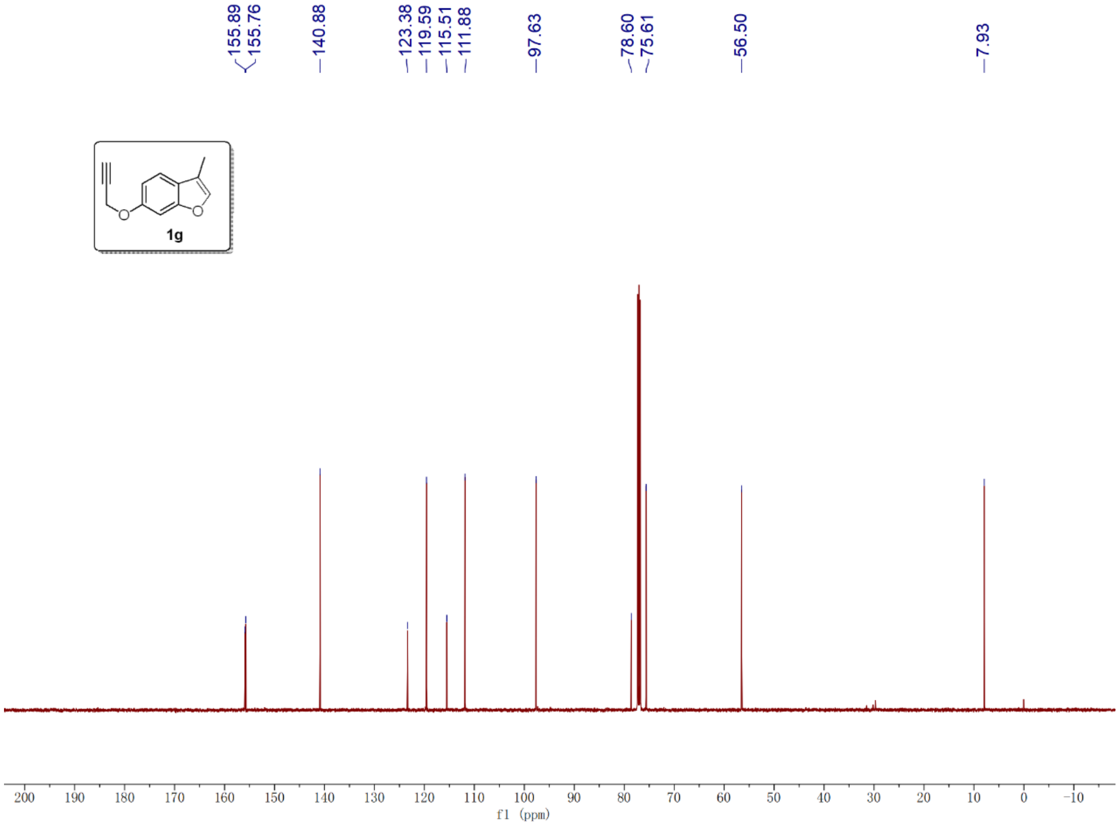

Supplementary Figure 16. ^{1}H-NMR spectrum of compound **1h** (400 MHz, $CDCl_3$)

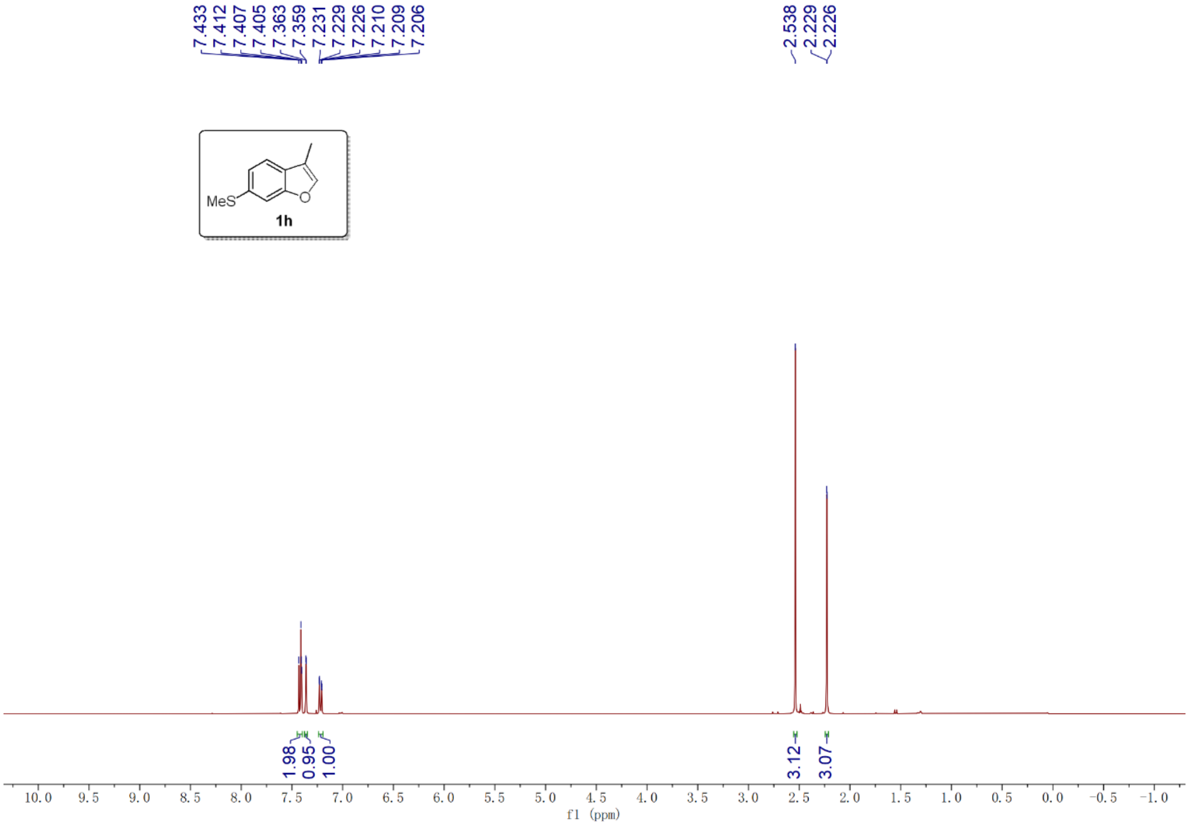

Supplementary Figure 17. ^{13}C-NMR spectrum of compound **1h** (100 MHz, $CDCl_3$)

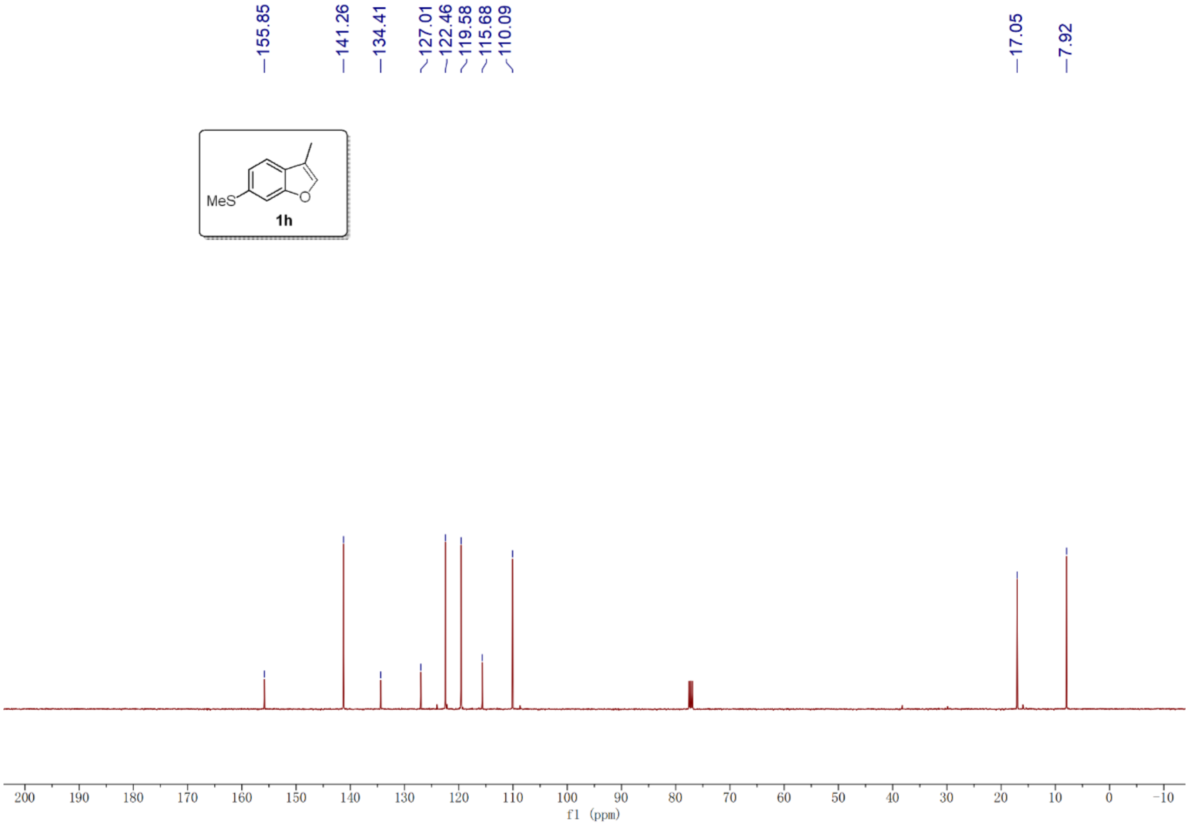

Supplementary Figure 18. ^{1}H-NMR spectrum of compound **1k** (500 MHz, $CDCl_3$)

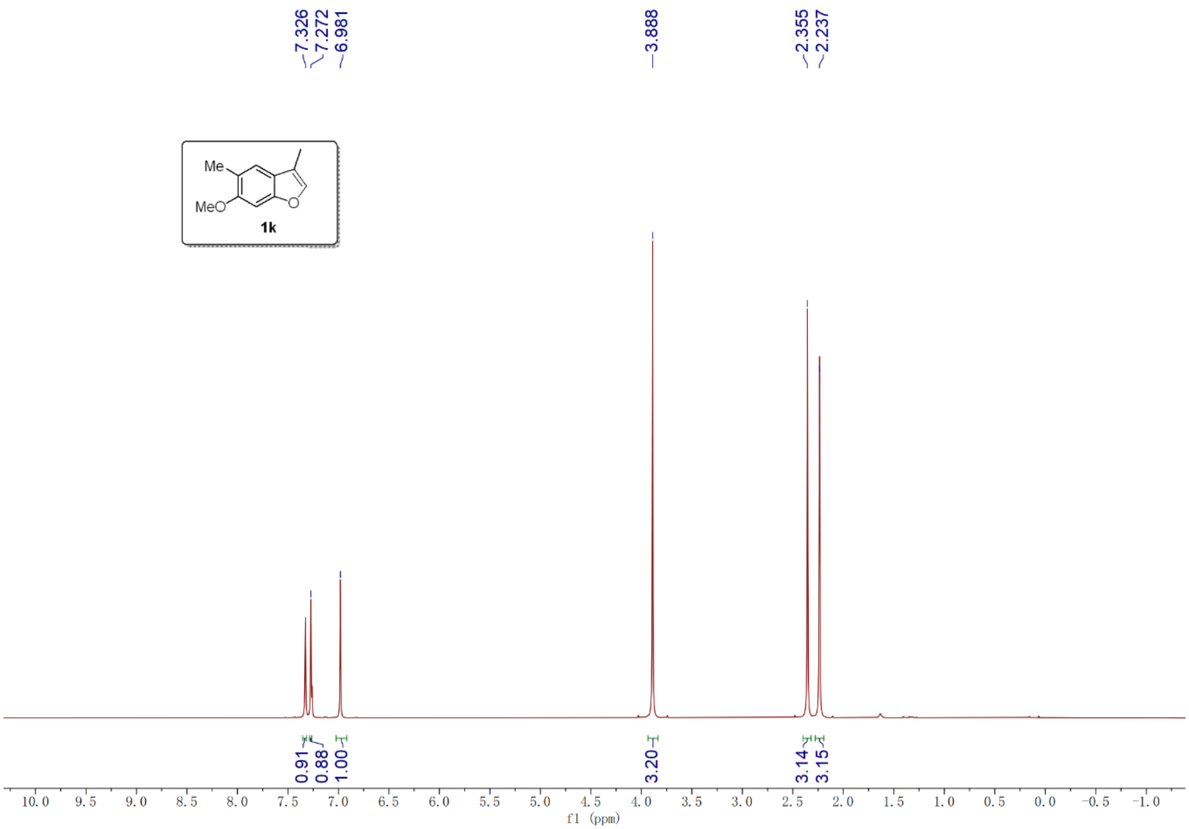

Supplementary Figure 19. ^{13}C-NMR spectrum of compound **1k** (125 MHz, $CDCl_3$)

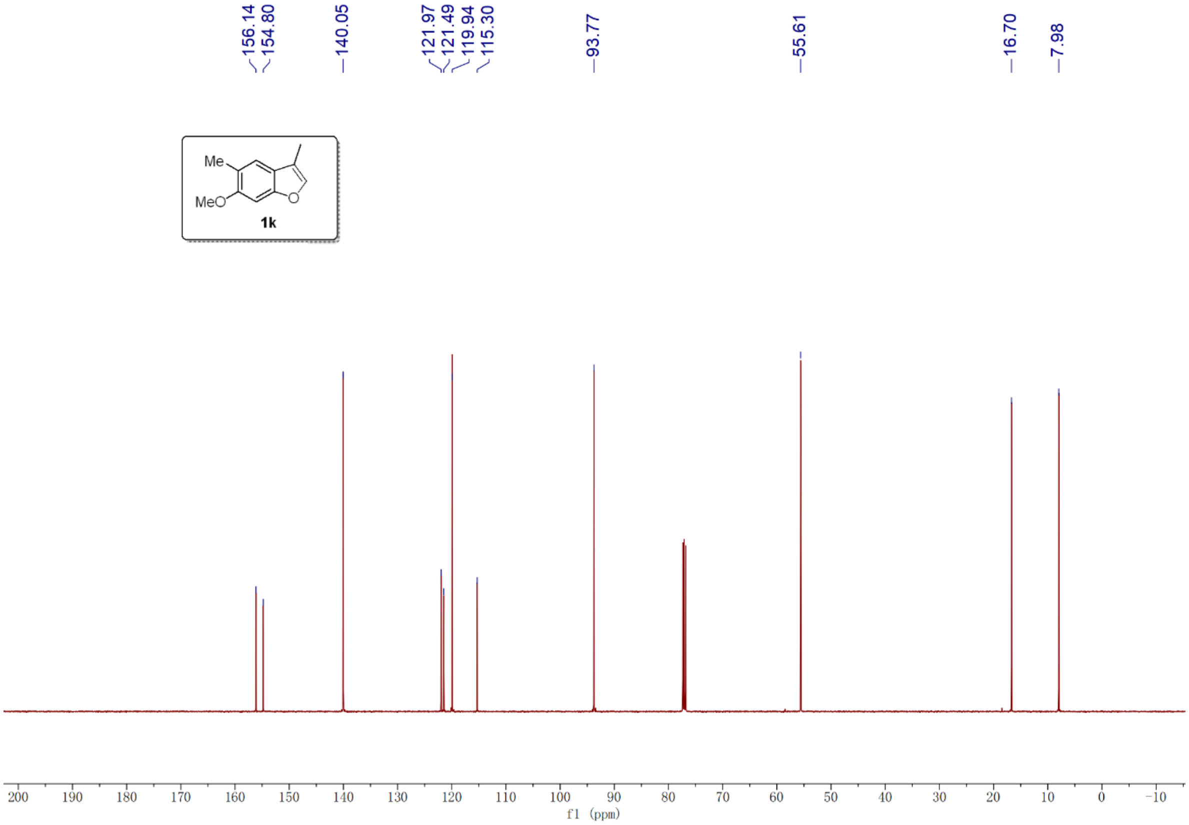

Supplementary Figure 20. ^{1}H-NMR spectrum of compound **1l** (500 MHz, $CDCl_3$)

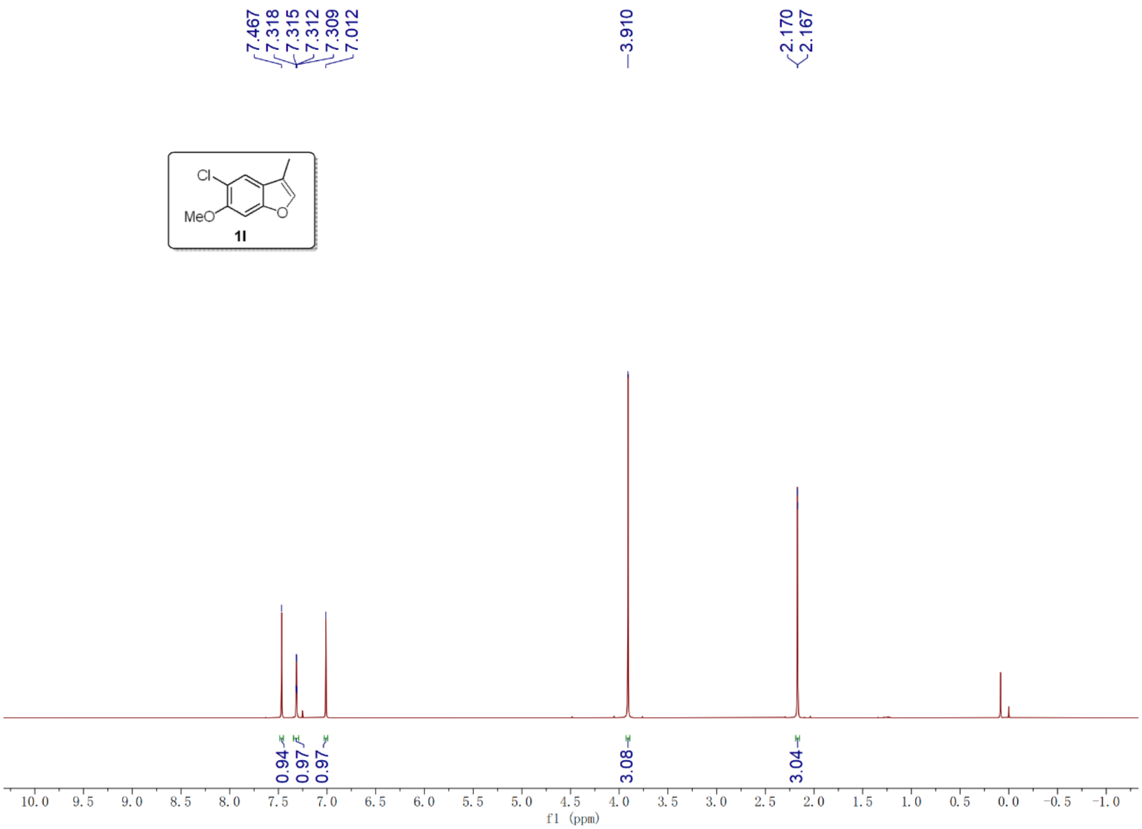

Supplementary Figure 21. ^{13}C-NMR spectrum of compound **1l** (125 MHz, $CDCl_3$)

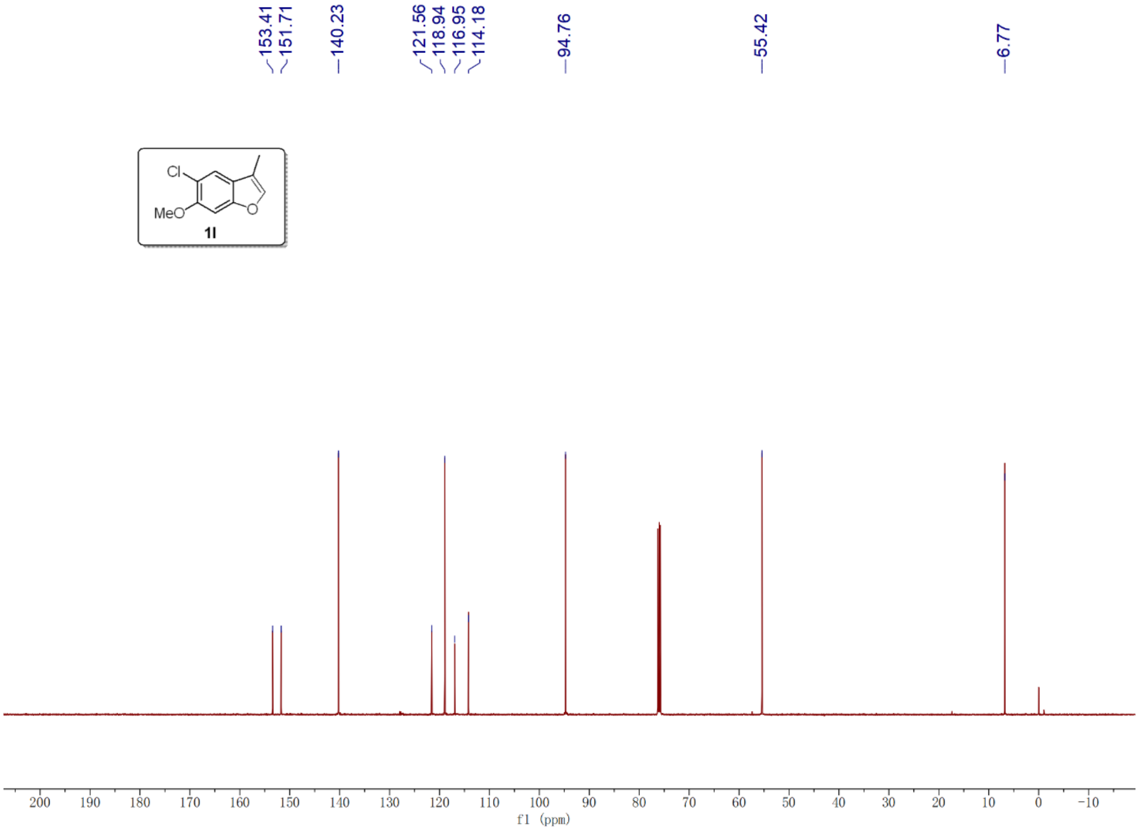

Supplementary Figure 22. ^{1}H-NMR spectrum of compound **1n** (400 MHz, DMSO)

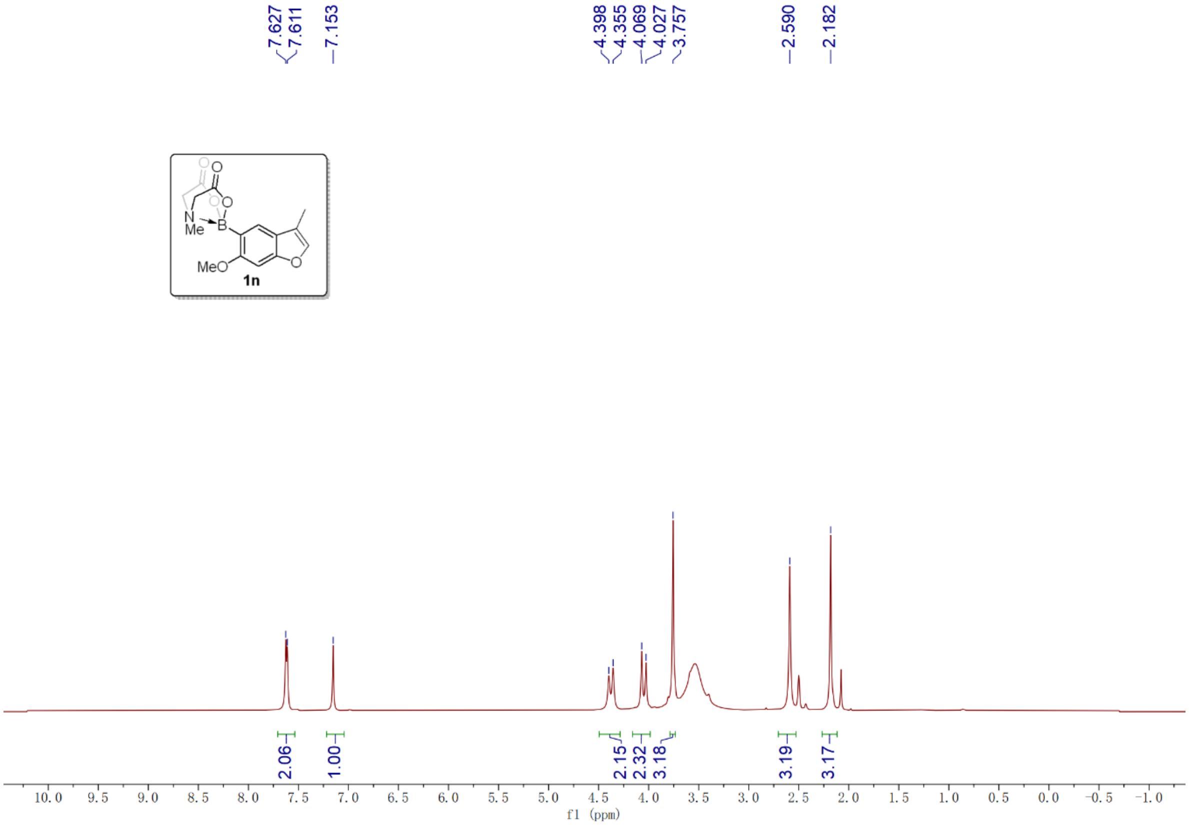

Supplementary Figure 23. ^{13}C-NMR spectrum of compound **1n** (100 MHz, DMSO)

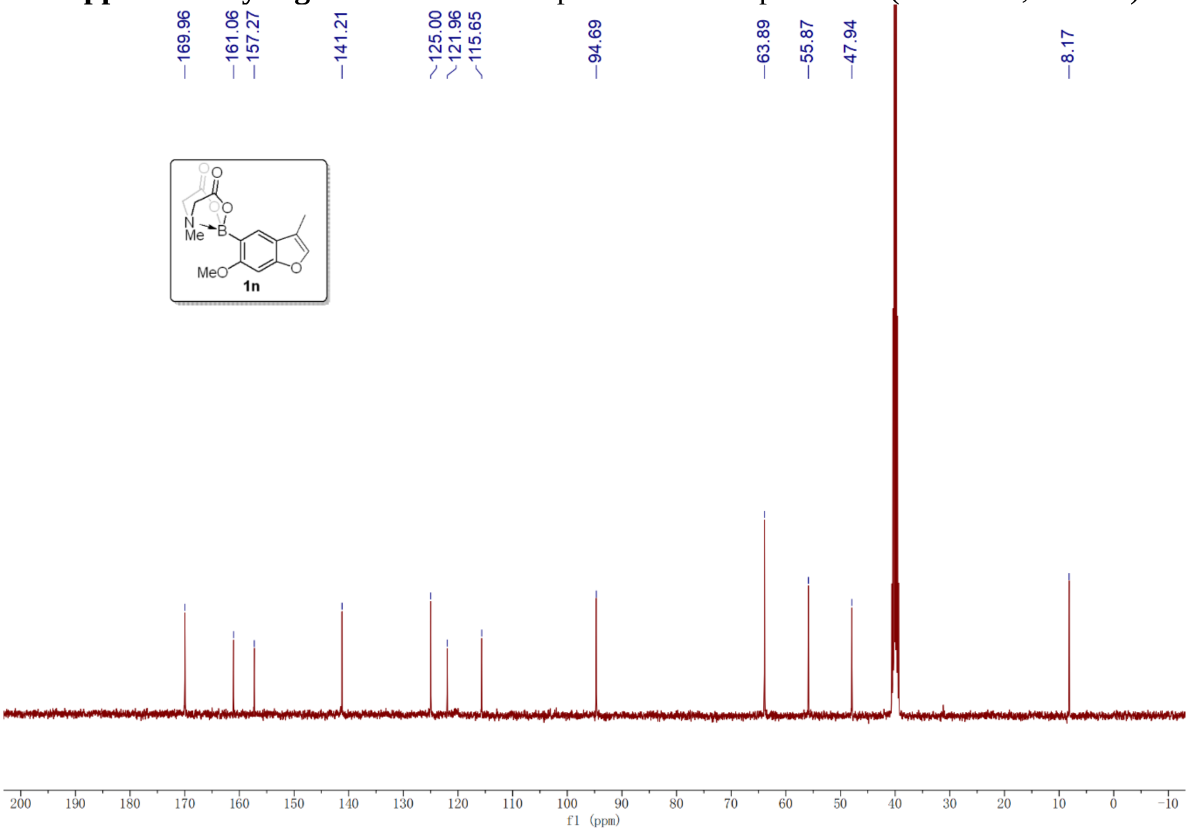

Supplementary Figure 24. ^{1}H-NMR spectrum of compound **1q** (500 MHz, $CDCl_3$)

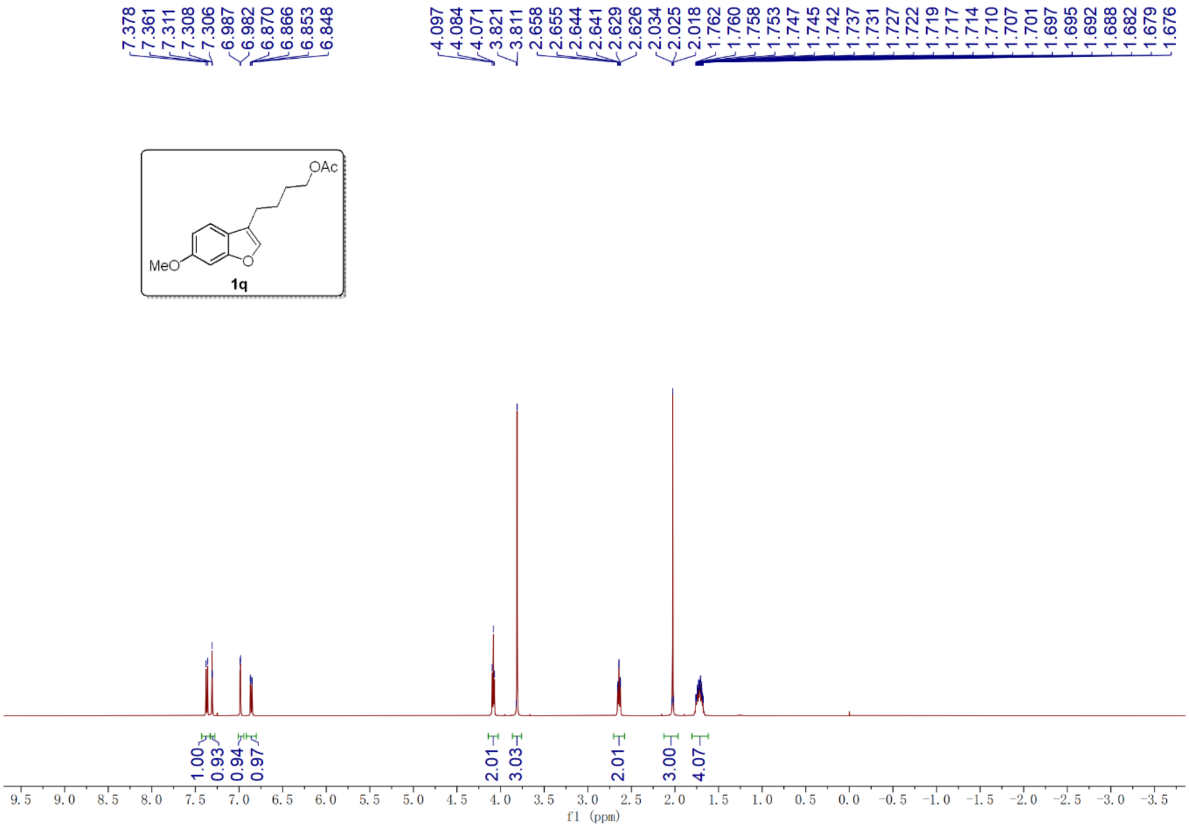

Supplementary Figure 25. ^{13}C-NMR spectrum of compound **1q** (125 MHz, $CDCl_3$)

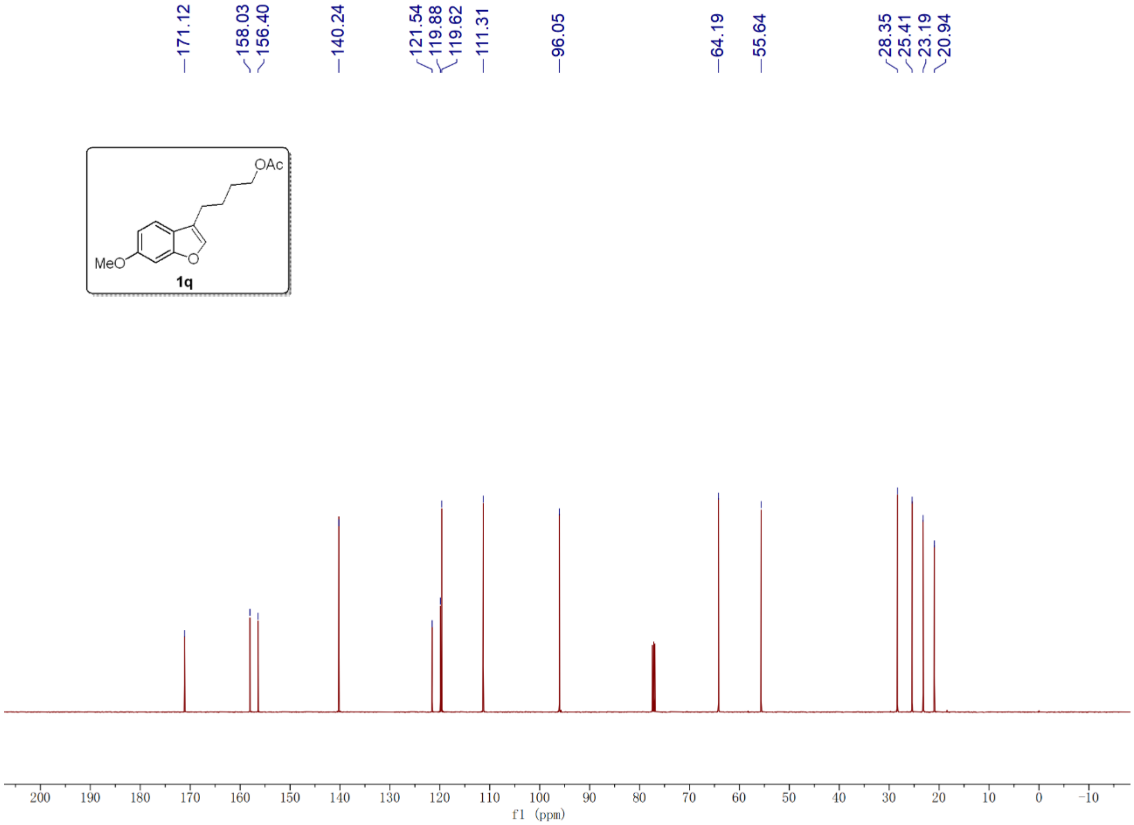

Supplementary Figure 26. ^{1}H-NMR spectrum of compound **1r** (500 MHz, $CDCl_3$)

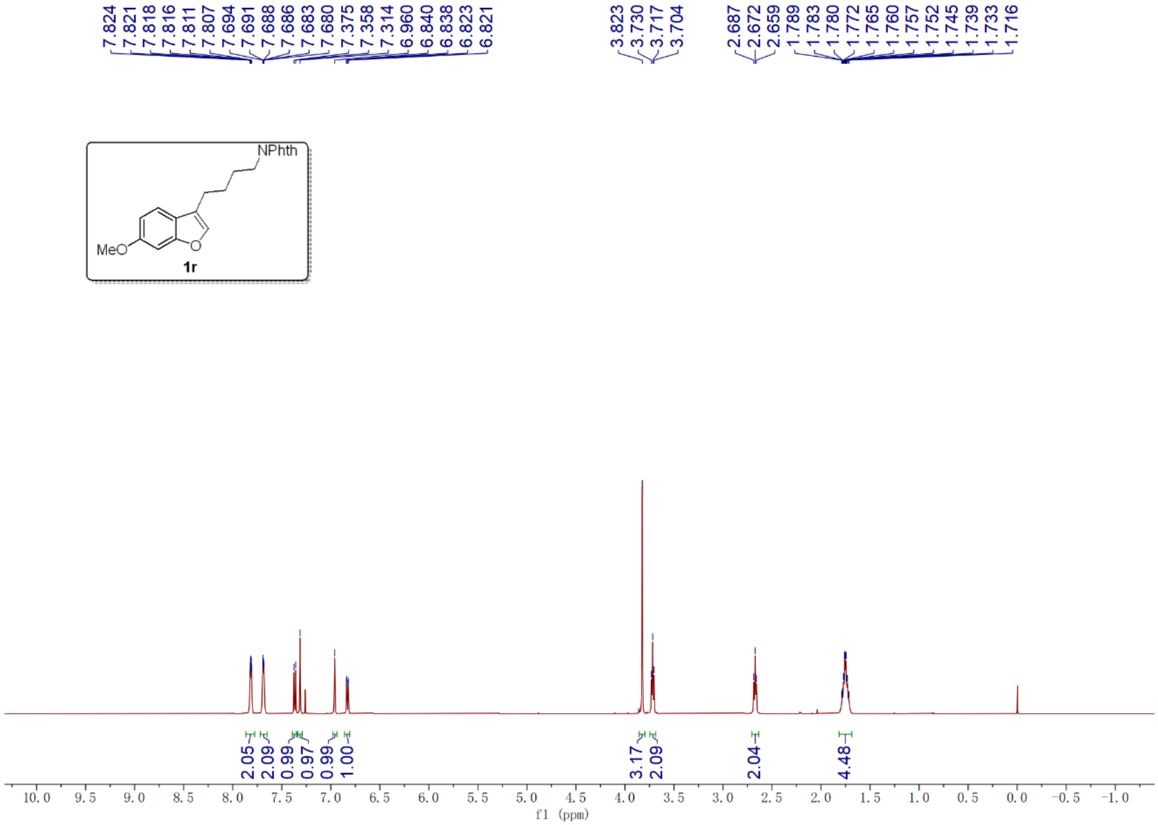

Supplementary Figure 27. ^{13}C-NMR spectrum of compound **1r** (125 MHz, $CDCl_3$)

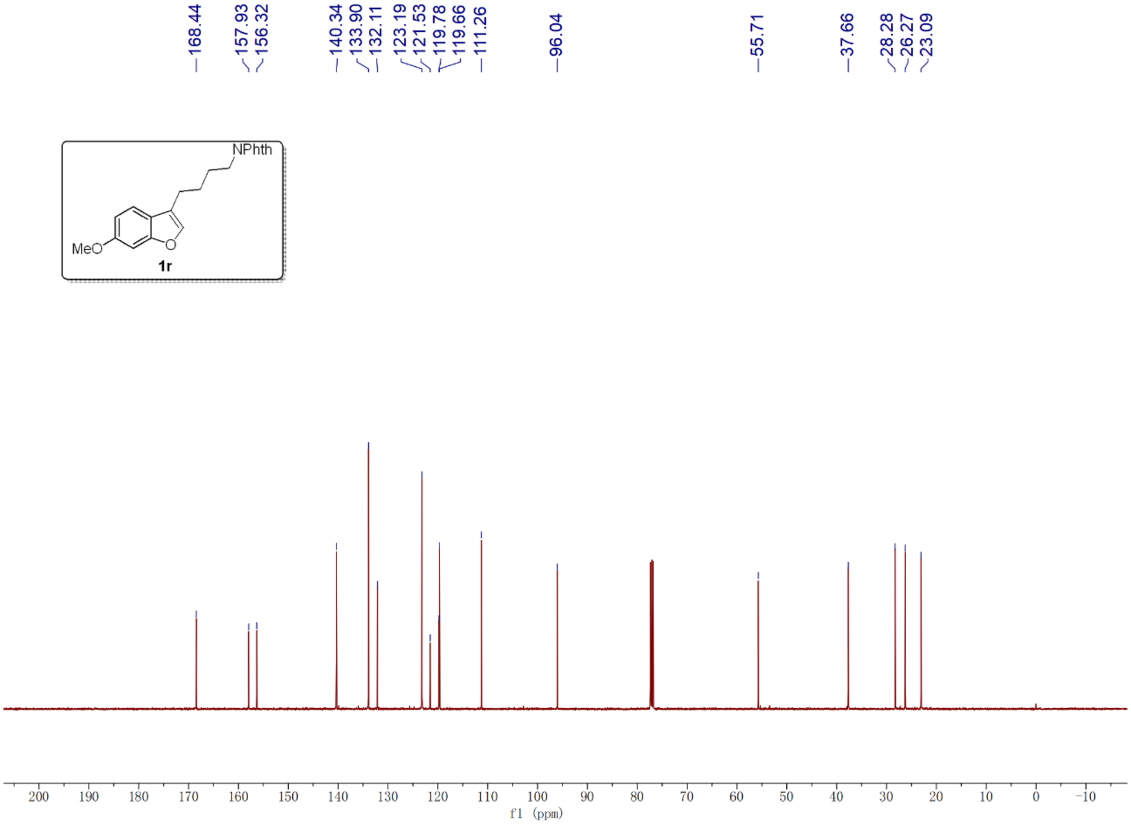

Supplementary Figure 28. ^{1}H-NMR spectrum of compound **1s** (500 MHz, $CDCl_3$)

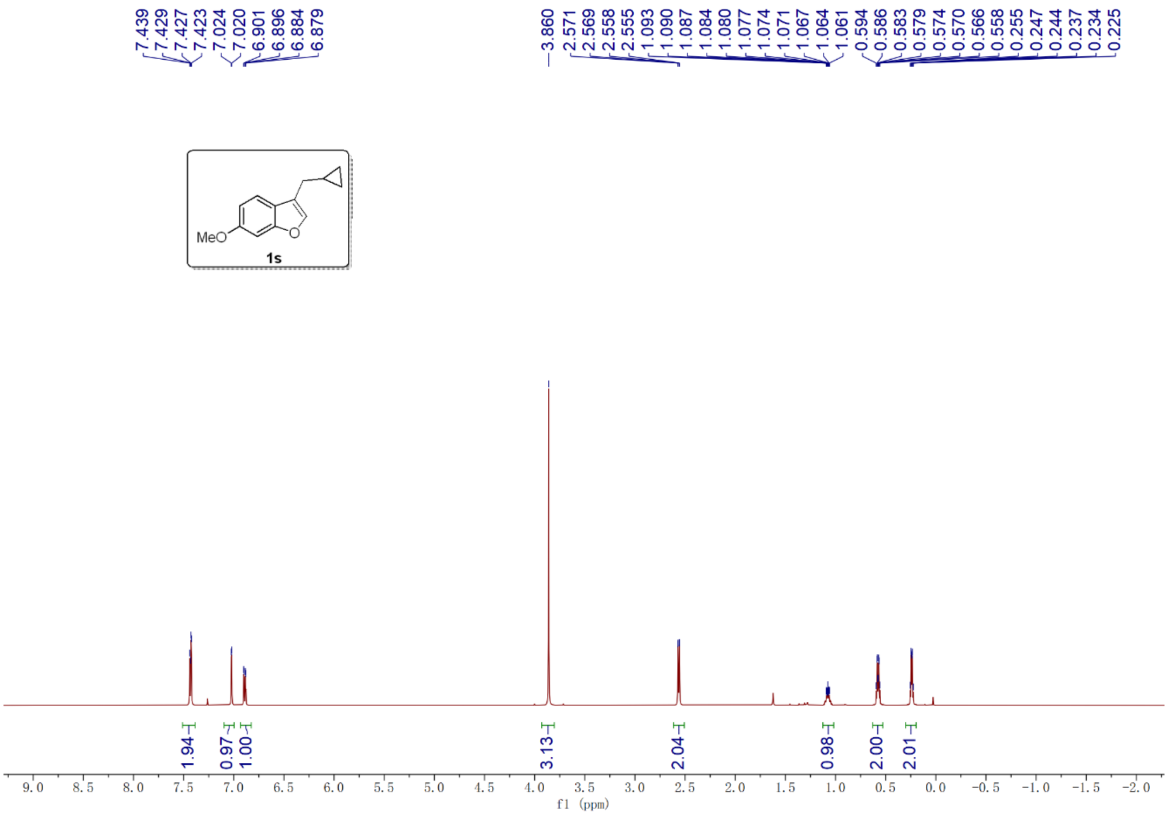

Supplementary Figure 29. ^{13}C-NMR spectrum of compound **1s** (125 MHz, $CDCl_3$)

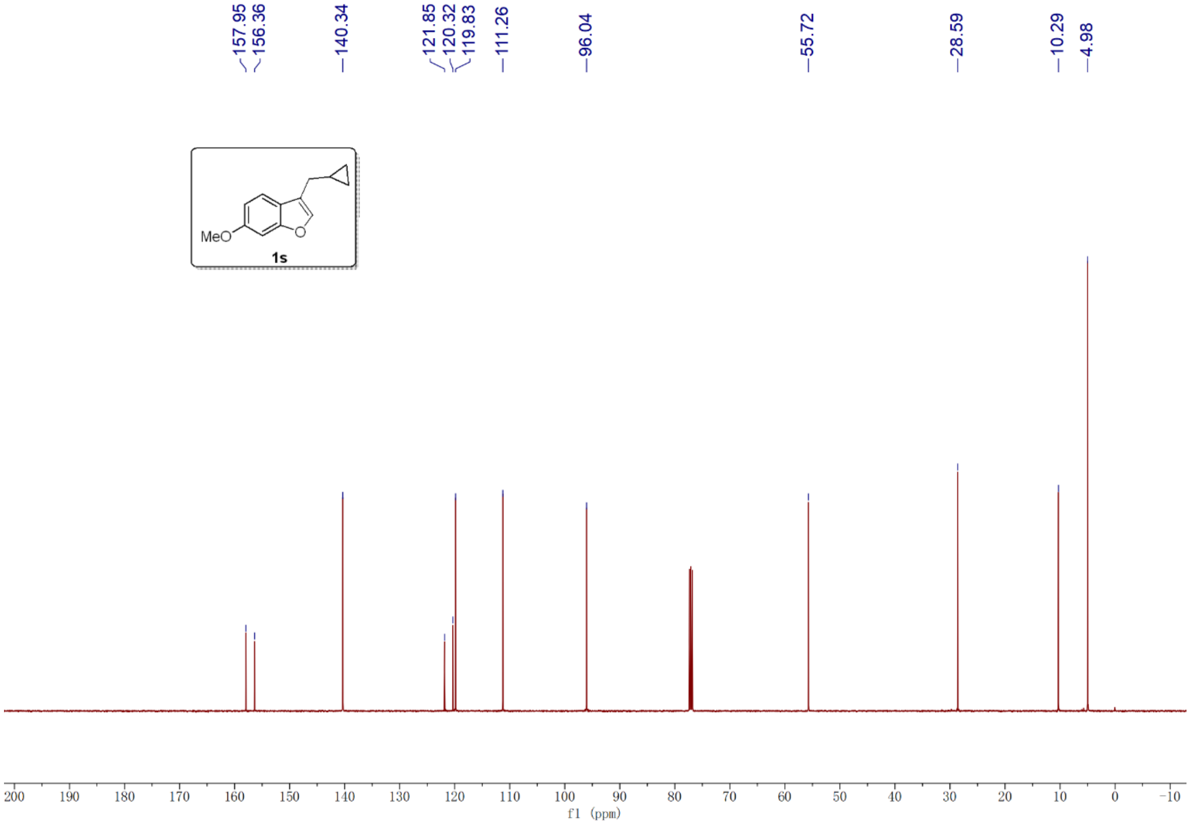

Supplementary Figure 30. ^{1}H-NMR spectrum of compound **[D$_3$]-1a** (500 MHz, $CDCl_3$)

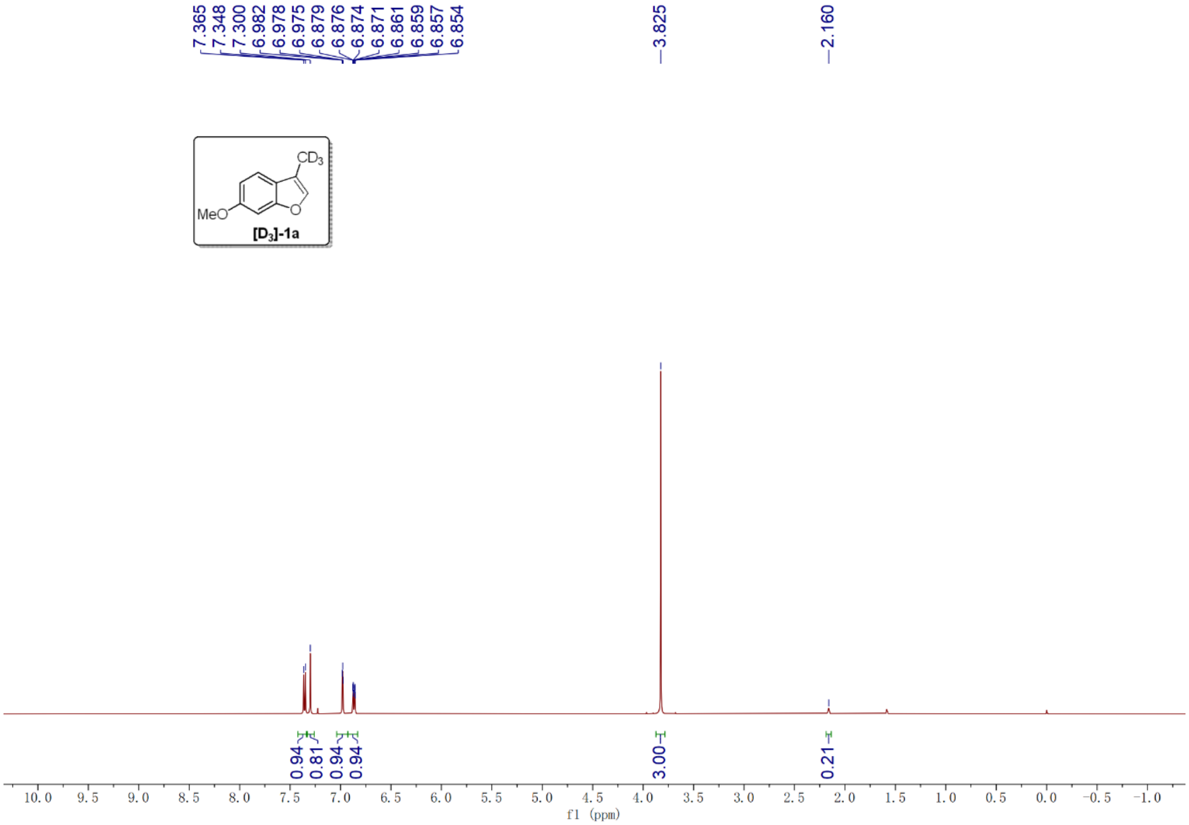

Supplementary Figure 31. ^{13}C-NMR spectrum of compound **[D$_3$]-1a** (125 MHz, $CDCl_3$)

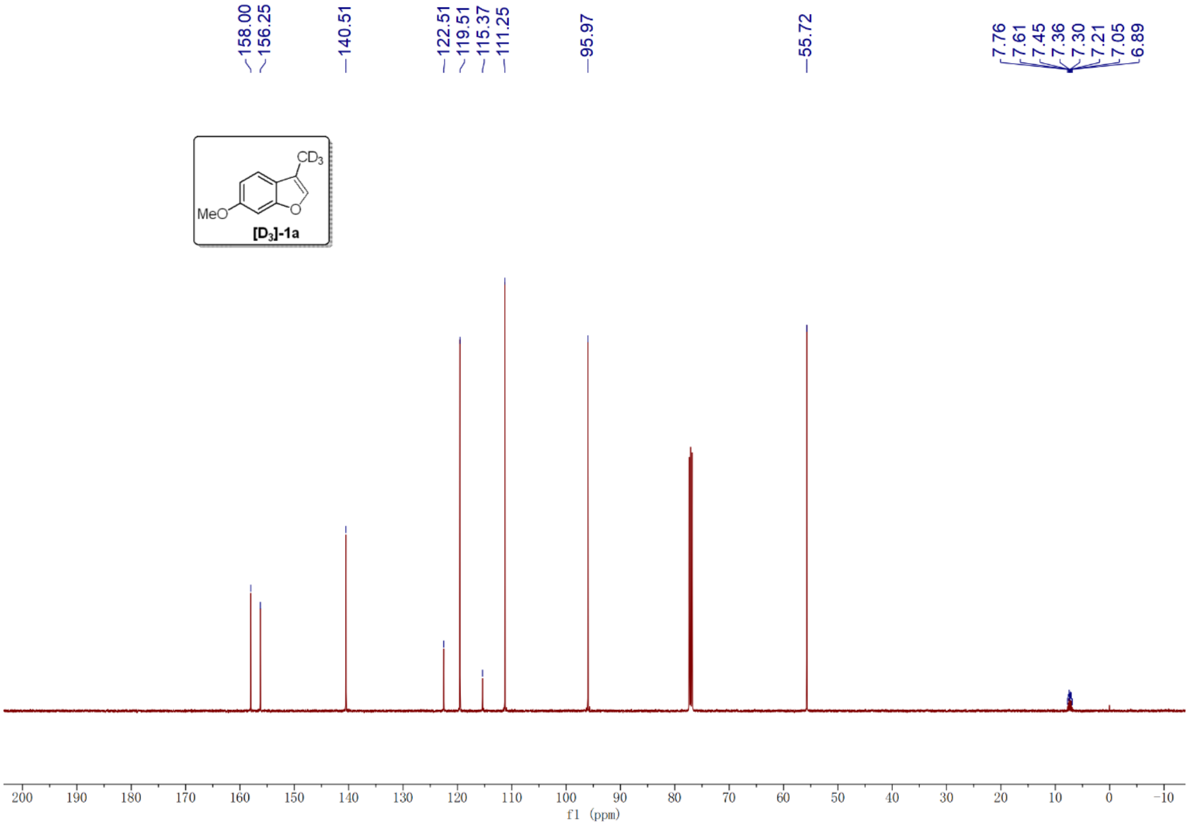

Supplementary Figure 32. ^{1}H-NMR spectrum of compound **4a** (500 MHz, $CDCl_3$)

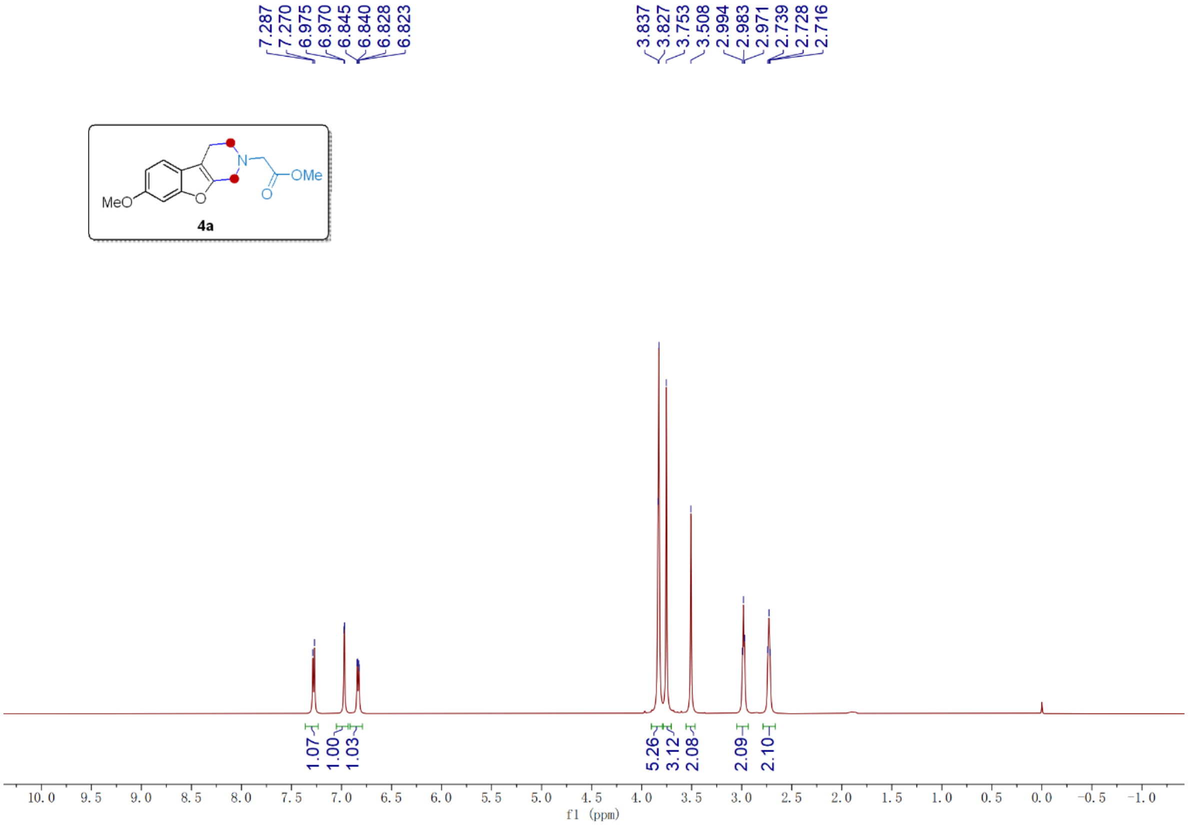

Supplementary Figure 33. ^{13}C-NMR spectrum of compound **4a** (100 MHz, $CDCl_3$)

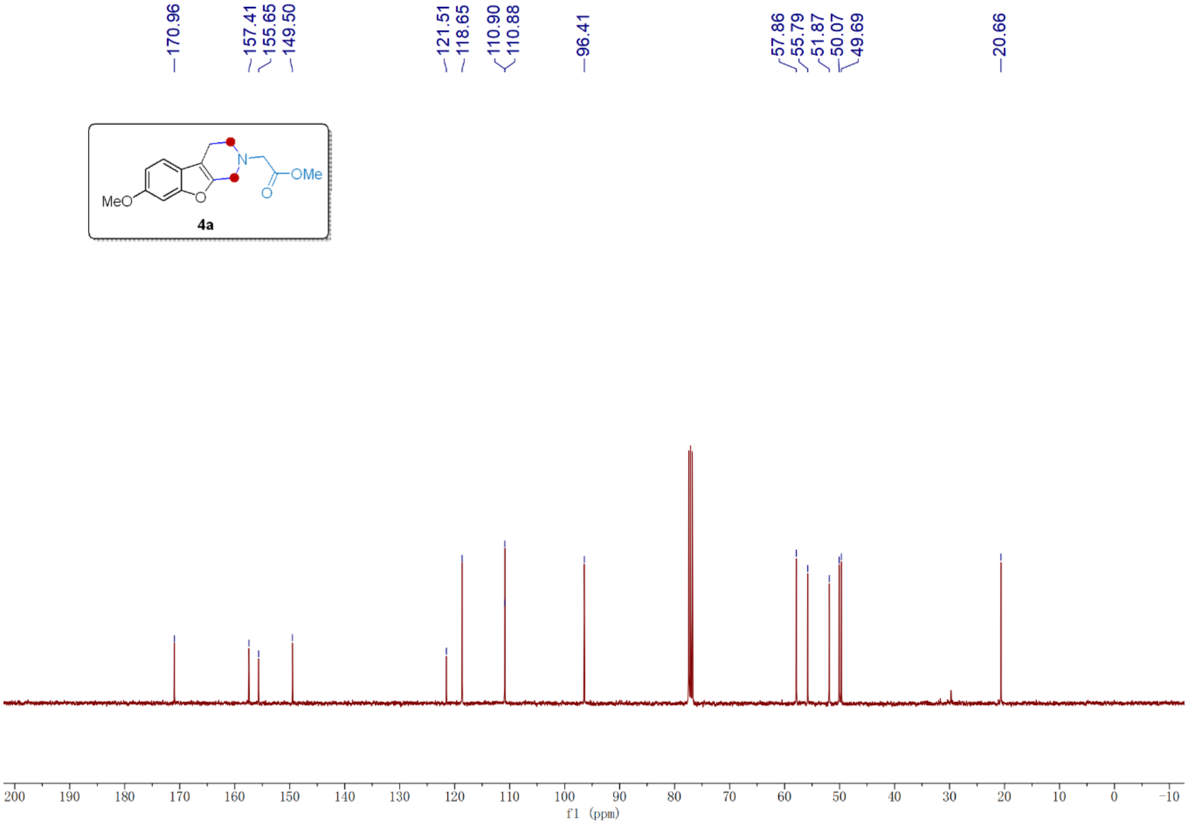

Supplementary Figure 34. ^{1}H-NMR spectrum of compound **4b** (600 MHz, $CDCl_3$)

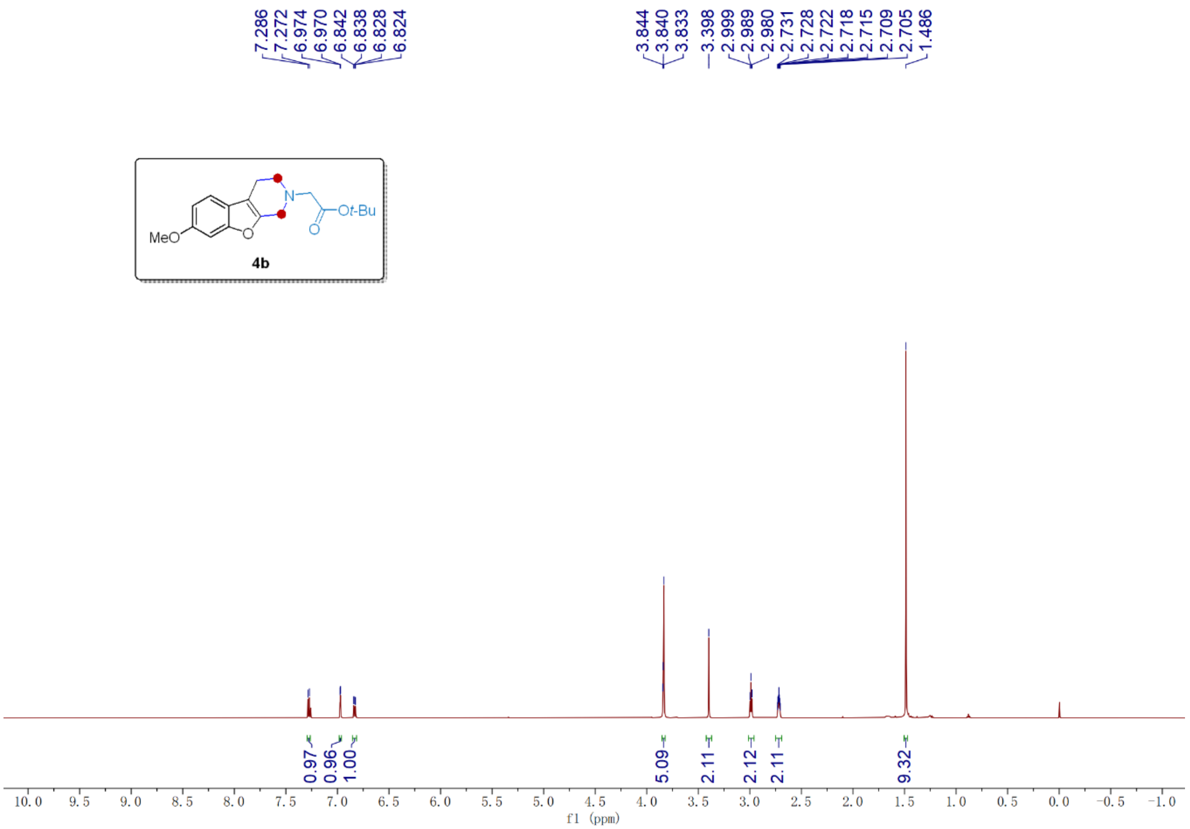

Supplementary Figure 35. ^{13}C-NMR spectrum of compound **4b** (150 MHz, $CDCl_3$)

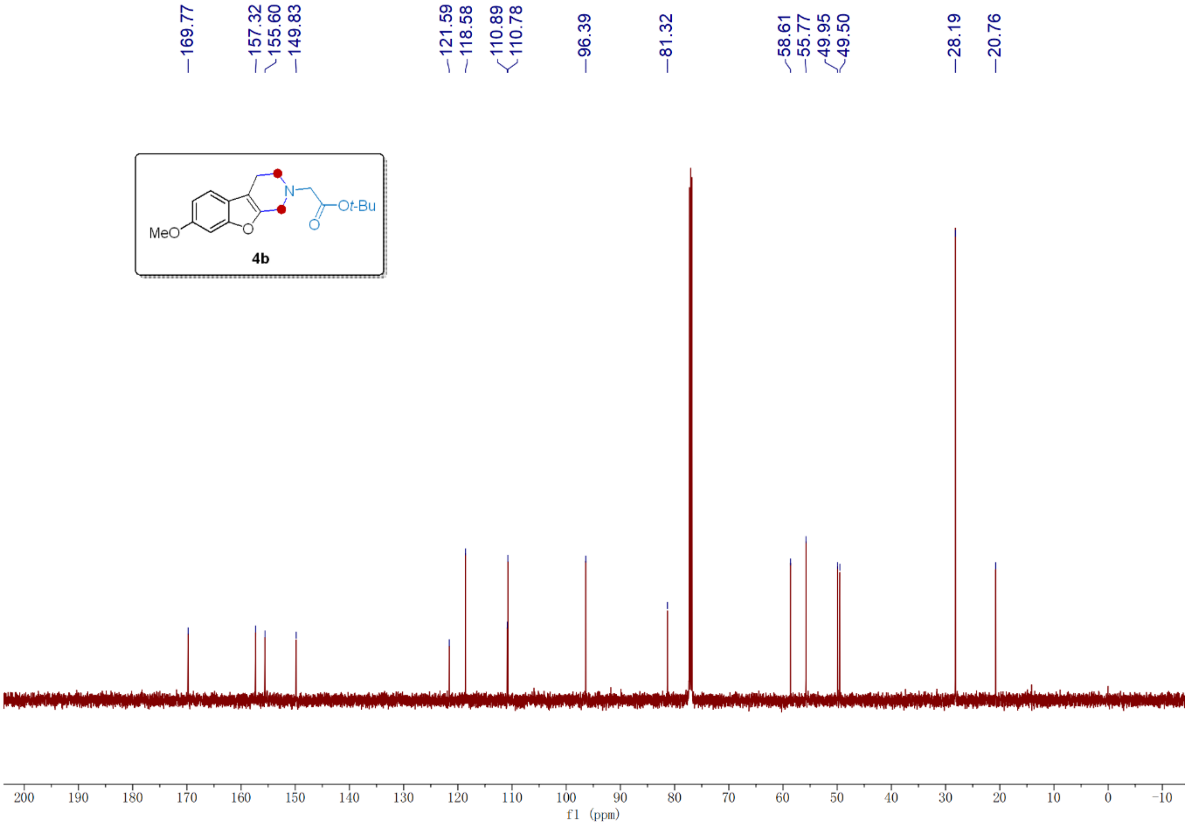

Supplementary Figure 36. ^{1}H-NMR spectrum of compound **4c** (400 MHz, $CDCl_3$)

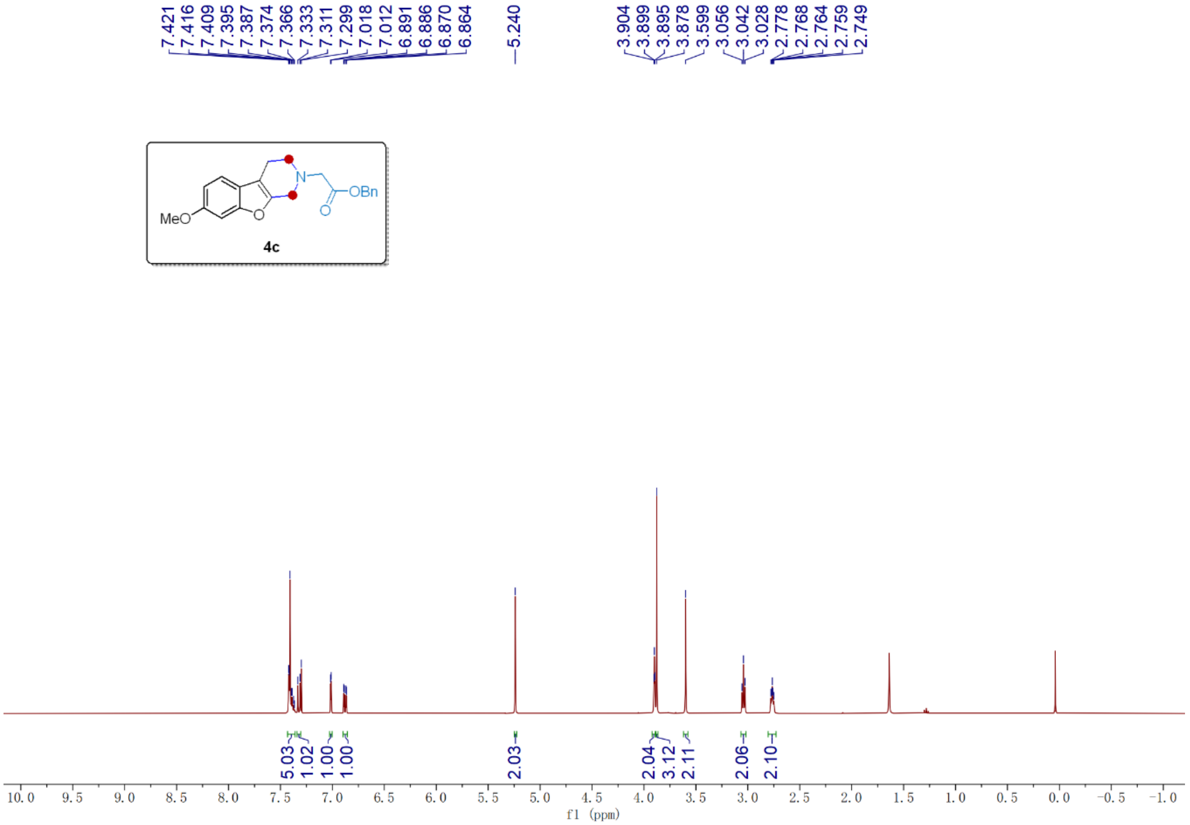

Supplementary Figure 37. ^{13}C-NMR spectrum of compound **4c** (100 MHz, $CDCl_3$)

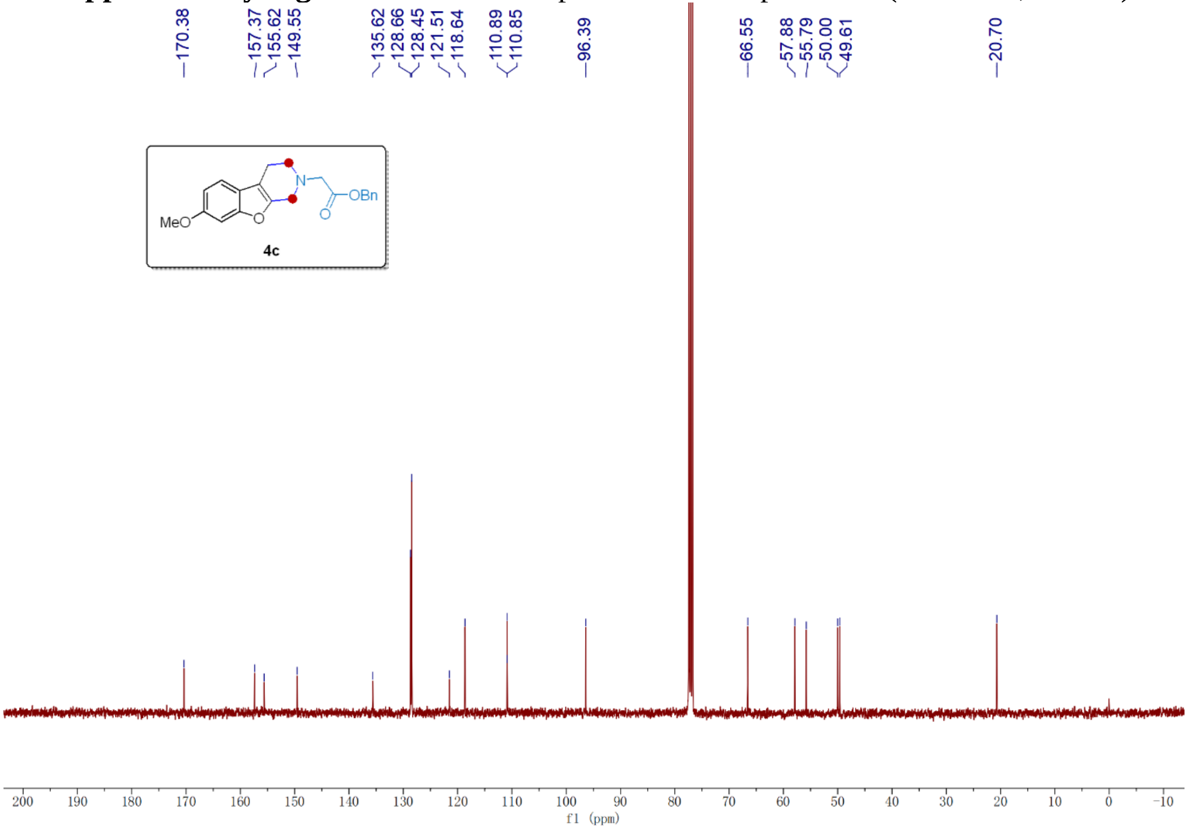

Supplementary Figure 38. ^{1}H-NMR spectrum of compound **4d** (600 MHz, $CDCl_3$)

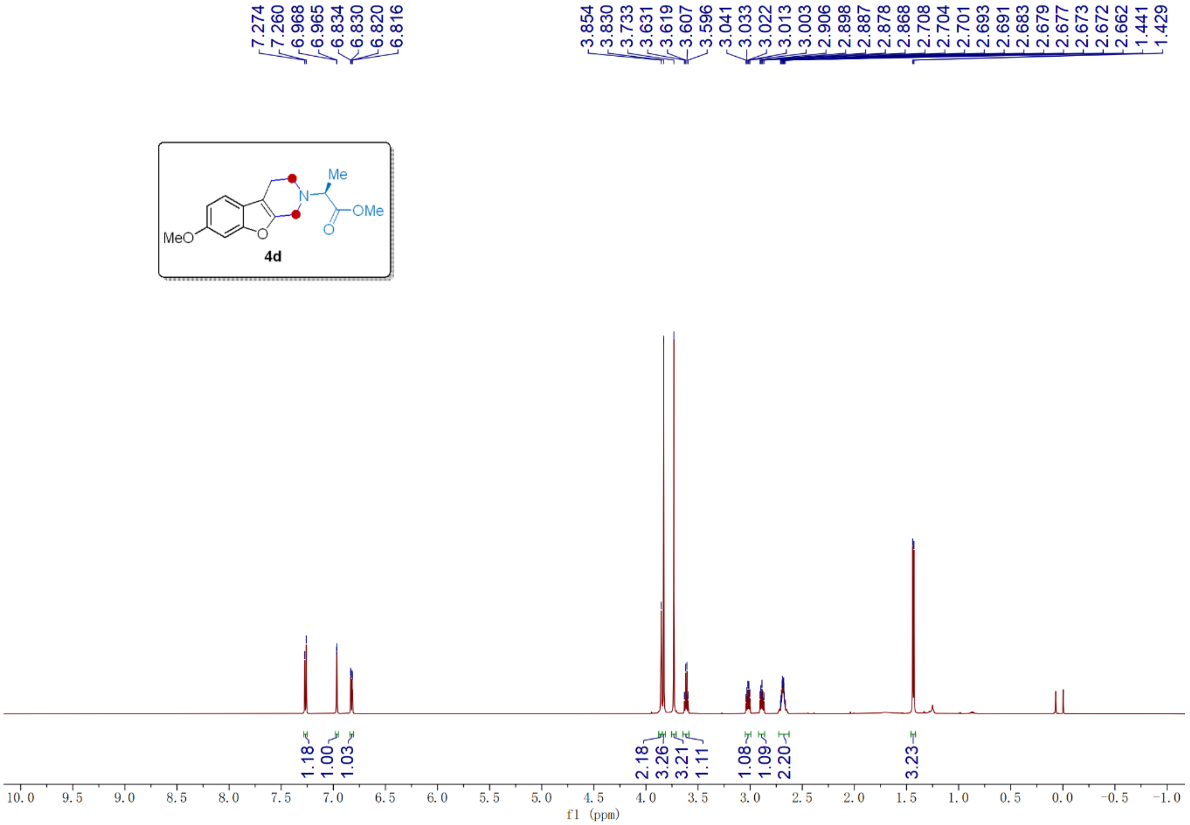

Supplementary Figure 39. ^{13}C-NMR spectrum of compound **4d** (150 MHz, $CDCl_3$)

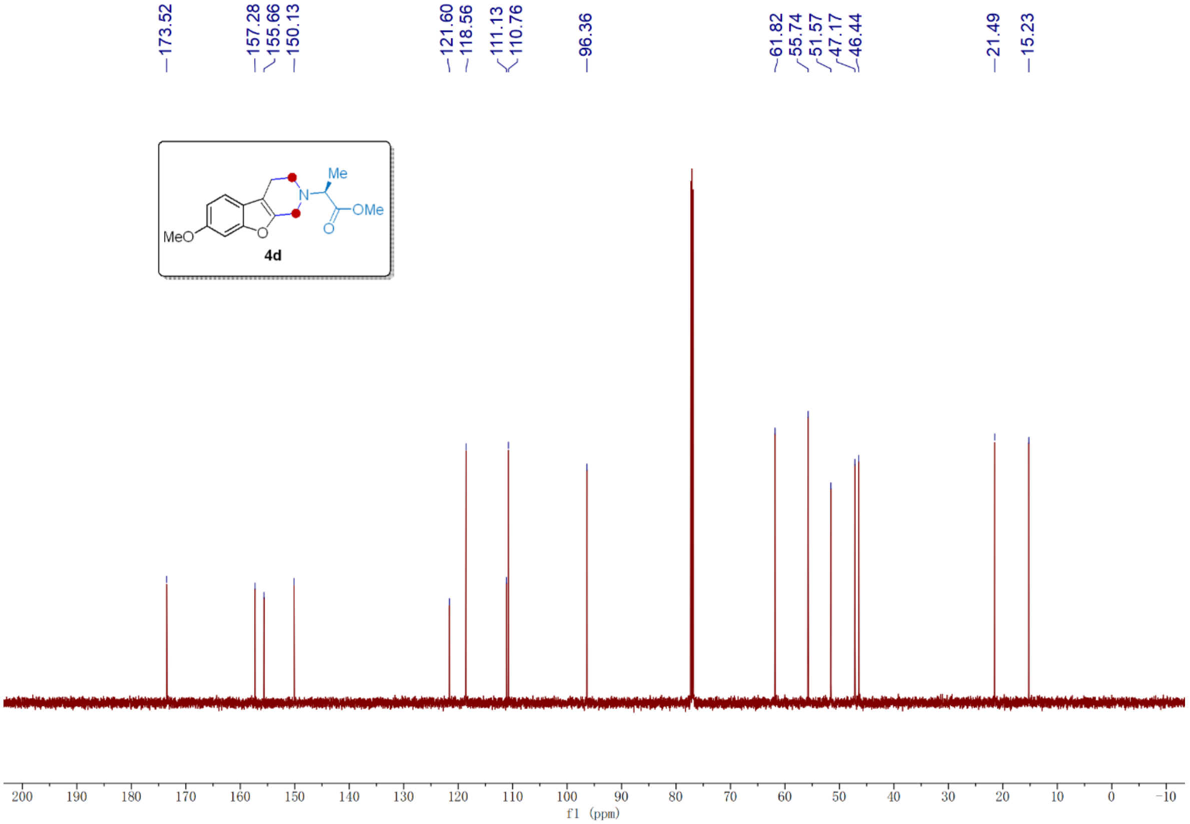

Supplementary Figure 40. ^{1}H-NMR spectrum of compound **4e** (600 MHz, $CDCl_3$)

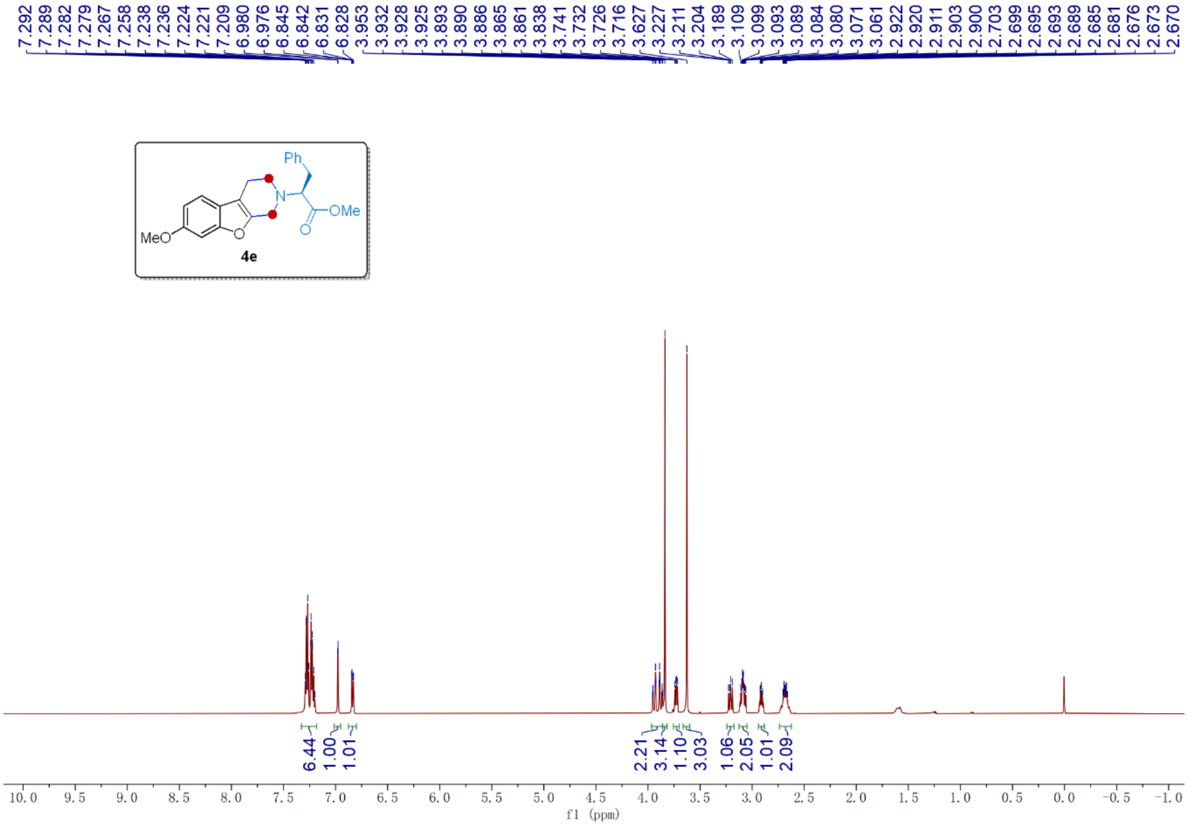

Supplementary Figure 41. ^{13}C-NMR spectrum of compound **4e** (100 MHz, $CDCl_3$)

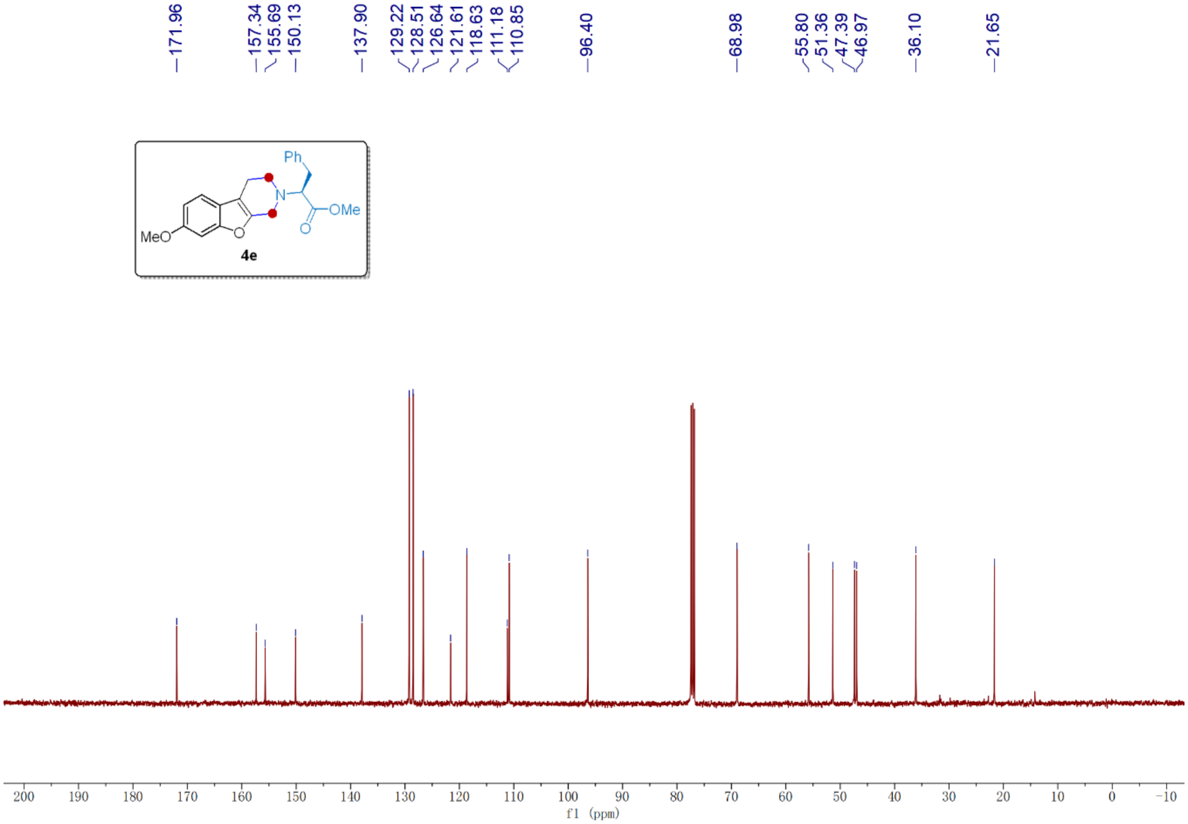

Supplementary Figure 42. ^{1}H-NMR spectrum of compound **4f** (400 MHz, $CDCl_3$)

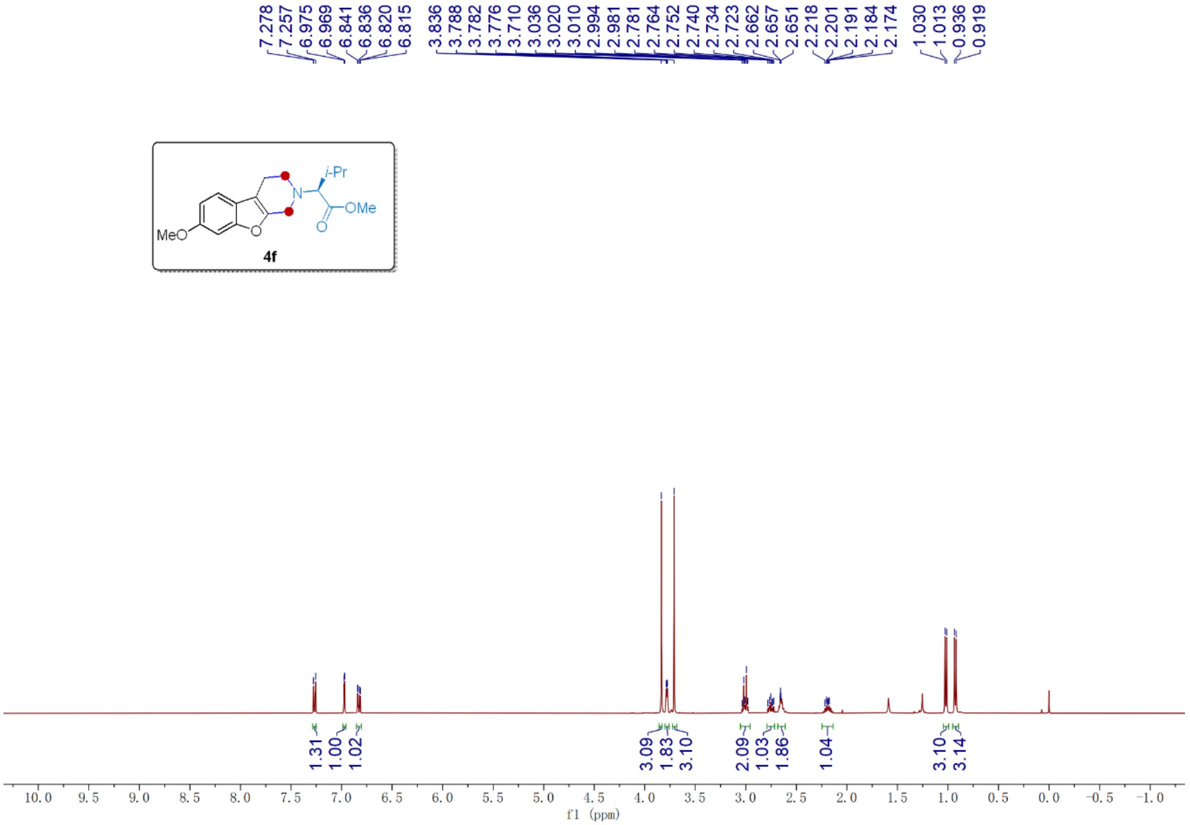

Supplementary Figure 43. ^{13}C-NMR spectrum of compound **4f** (100 MHz, $CDCl_3$)

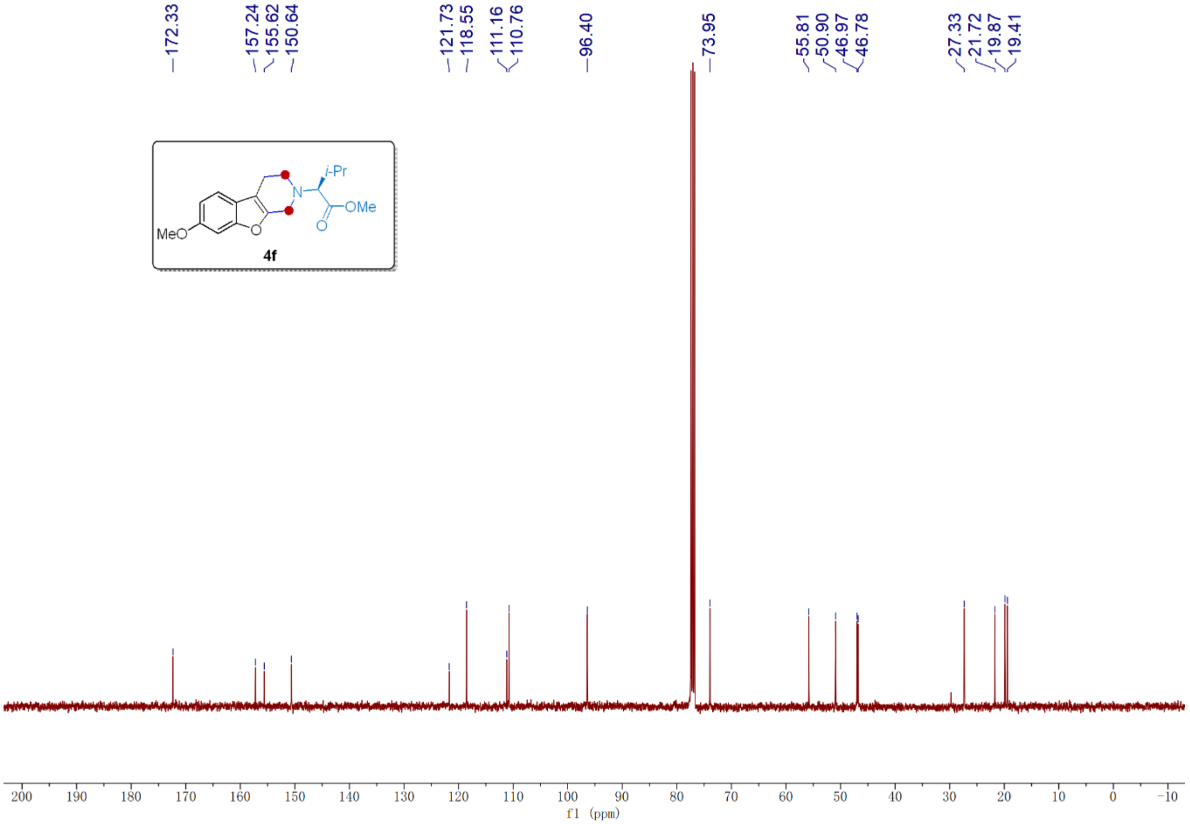

Supplementary Figure 44. ^{1}H-NMR spectrum of compound **4g** (400 MHz, $CDCl_3$)

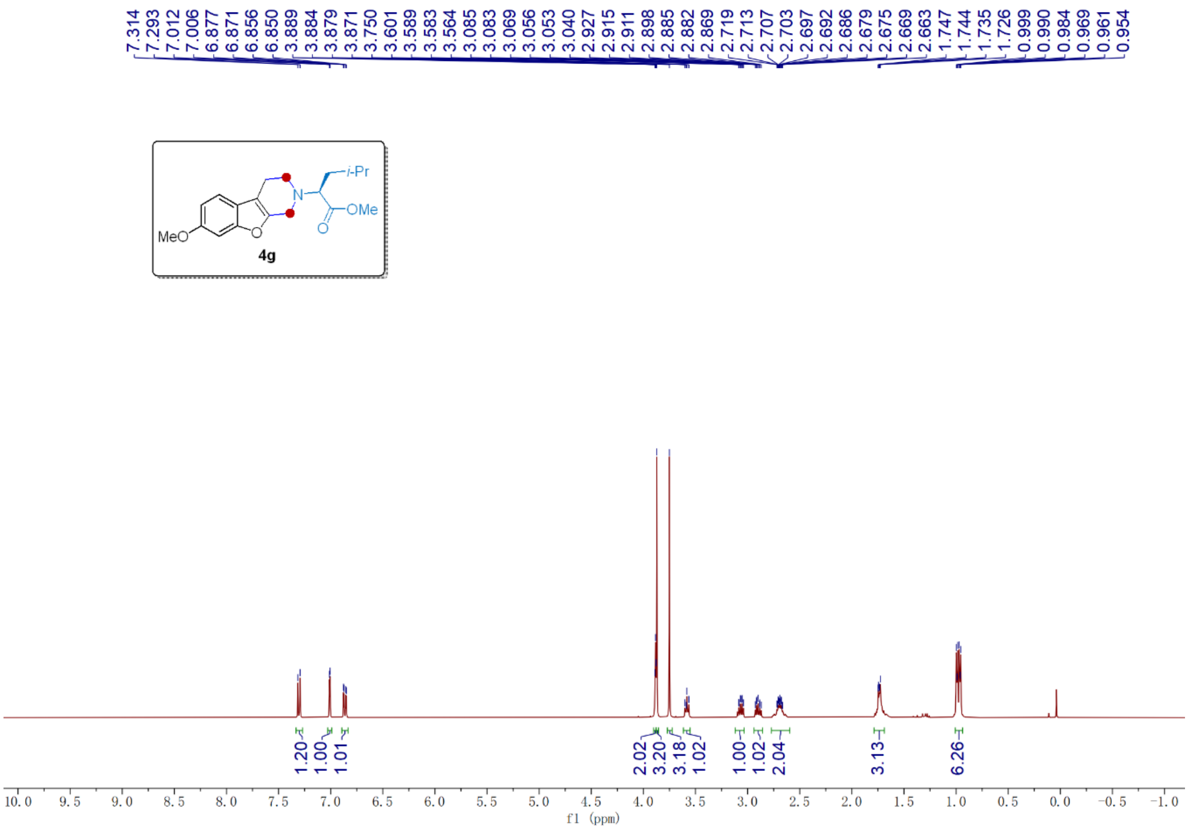

Supplementary Figure 45. ^{13}C-NMR spectrum of compound **4g** (100 MHz, $CDCl_3$)

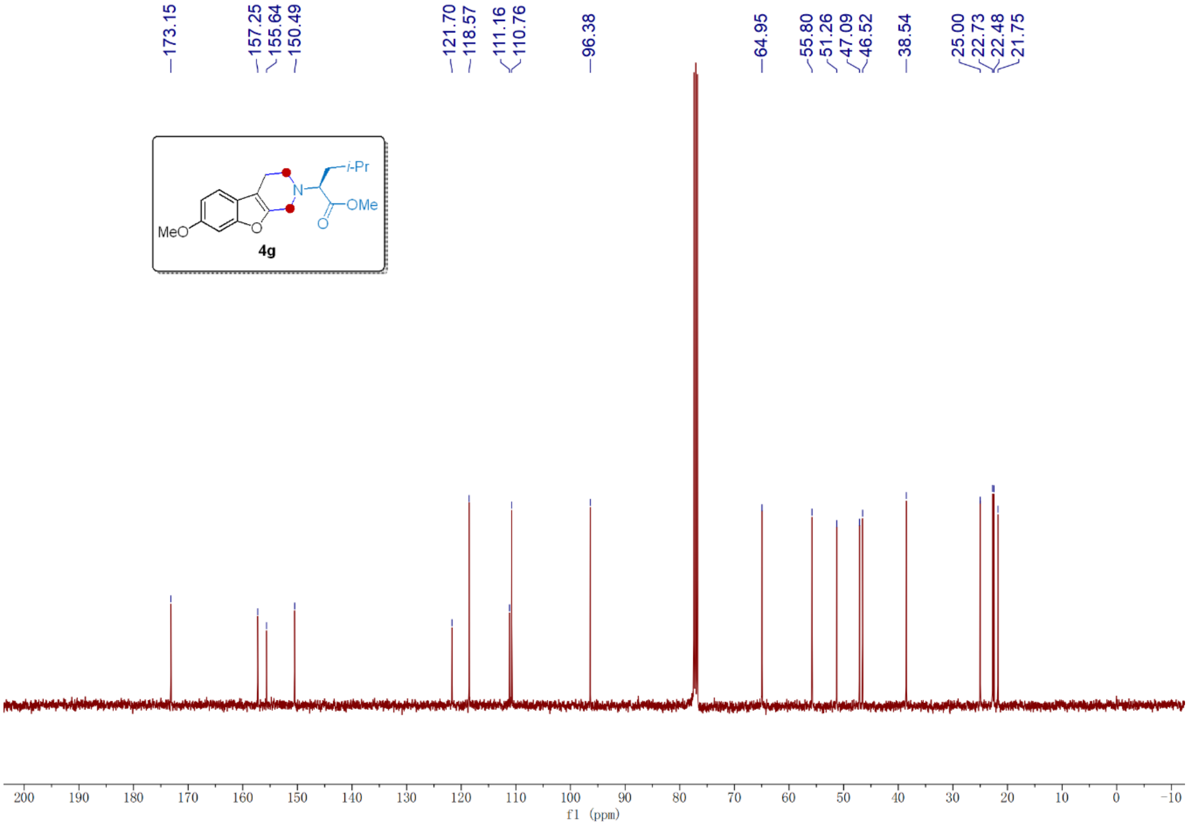

Supplementary Figure 46. ^{1}H-NMR spectrum of compound **4h** (400 MHz, $CDCl_3$)

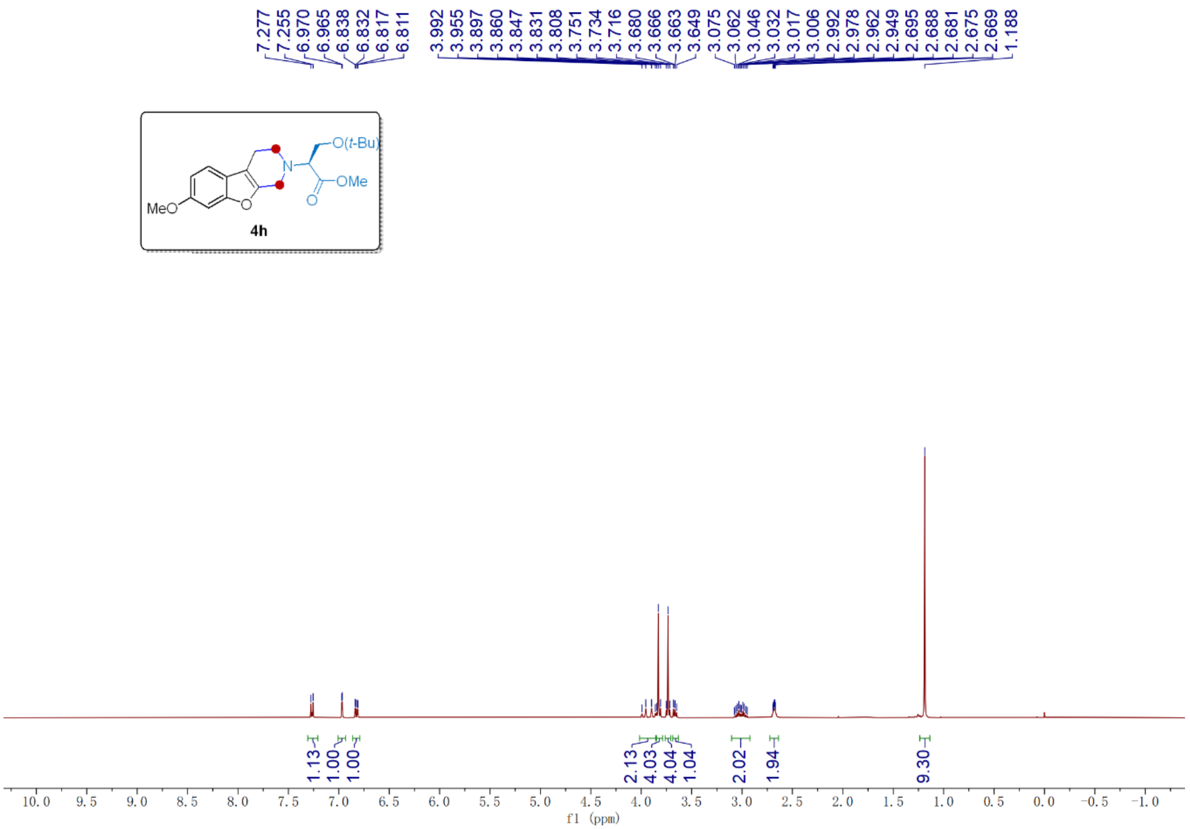

Supplementary Figure 47. ^{13}C-NMR spectrum of compound **4h** (125 MHz, $CDCl_3$)

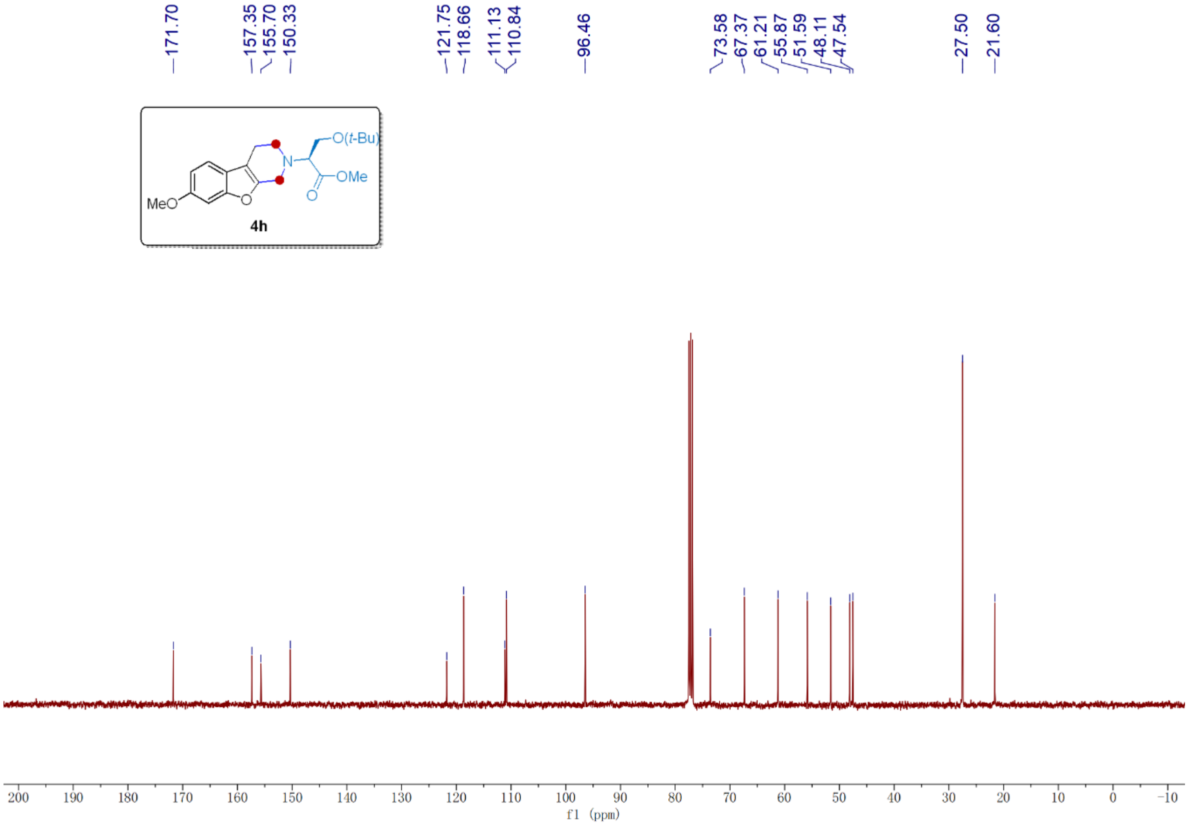

Supplementary Figure 48. ^{1}H-NMR spectrum of compound **4i** (400 MHz, $CDCl_3$)

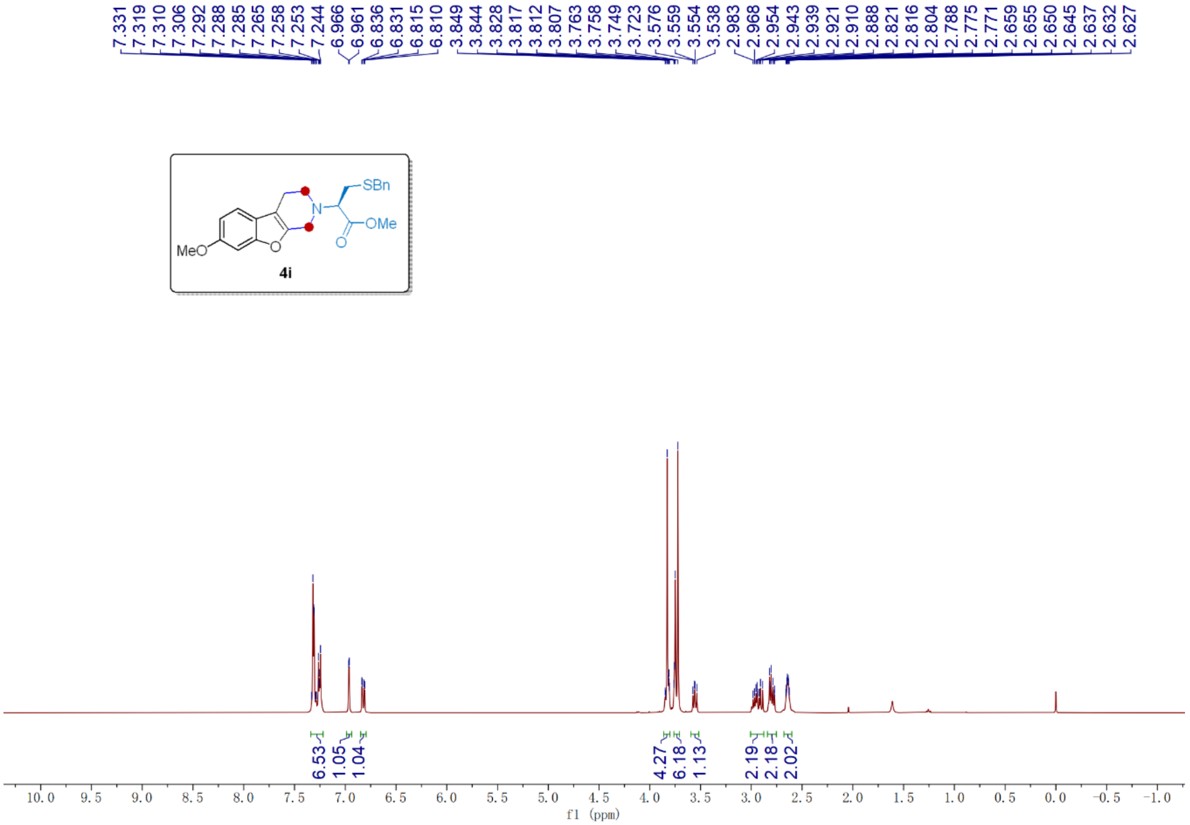

Supplementary Figure 49. ^{13}C-NMR spectrum of compound **4i** (100 MHz, $CDCl_3$)

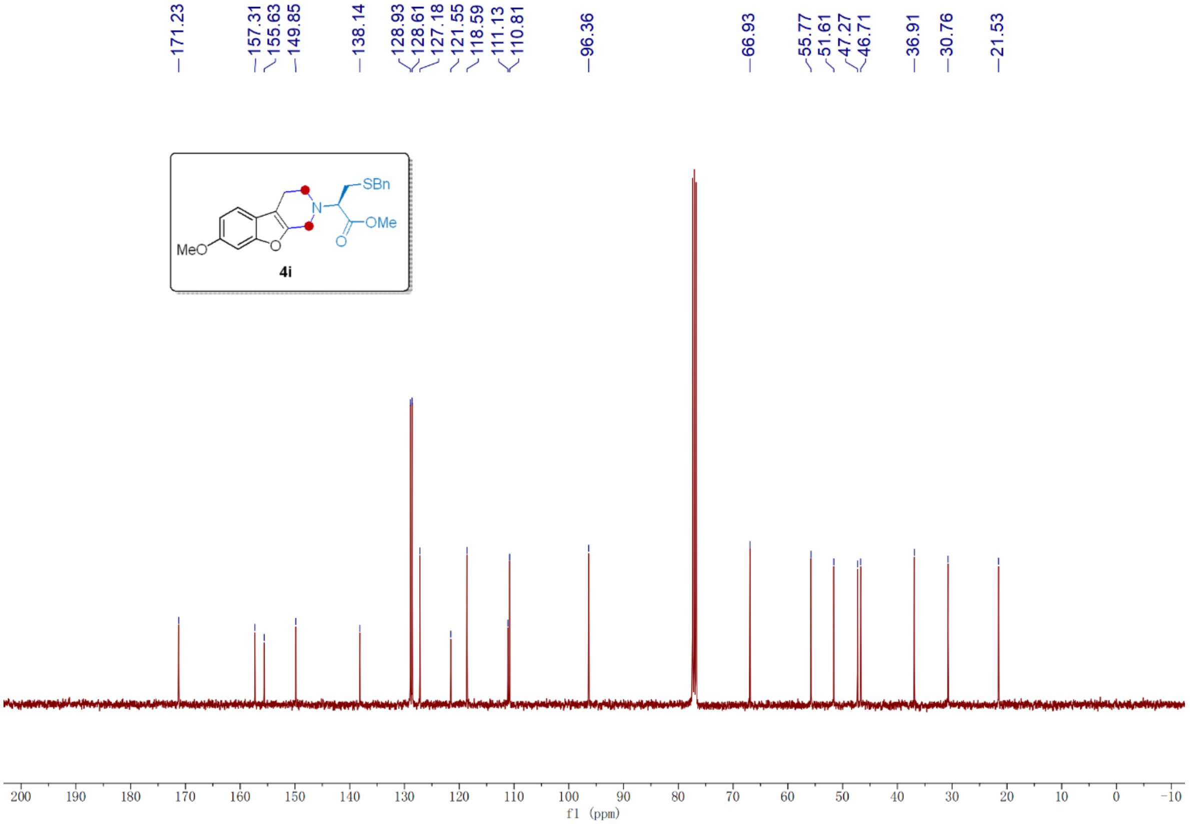

Supplementary Figure 50. ^{1}H-NMR spectrum of compound **4j** (600 MHz, $CDCl_3$)

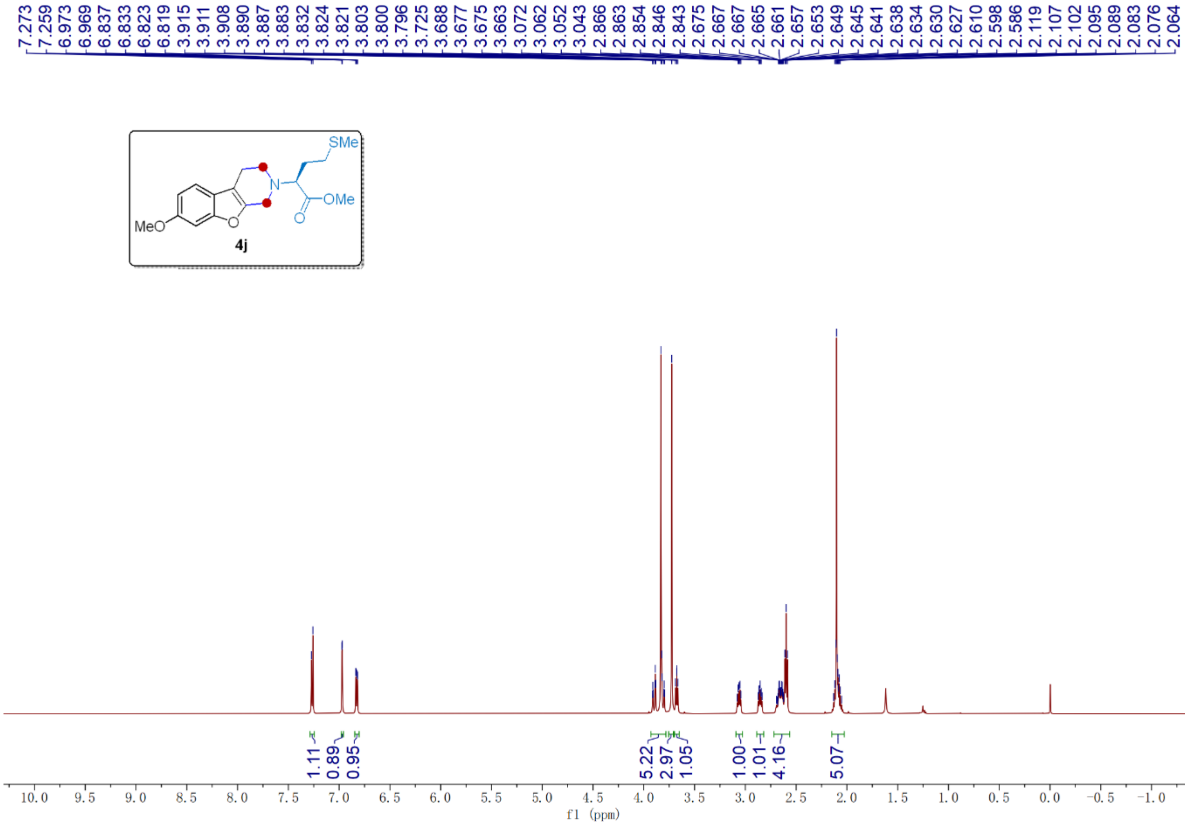

Supplementary Figure 51. ^{13}C-NMR spectrum of compound **4j** (150 MHz, $CDCl_3$)

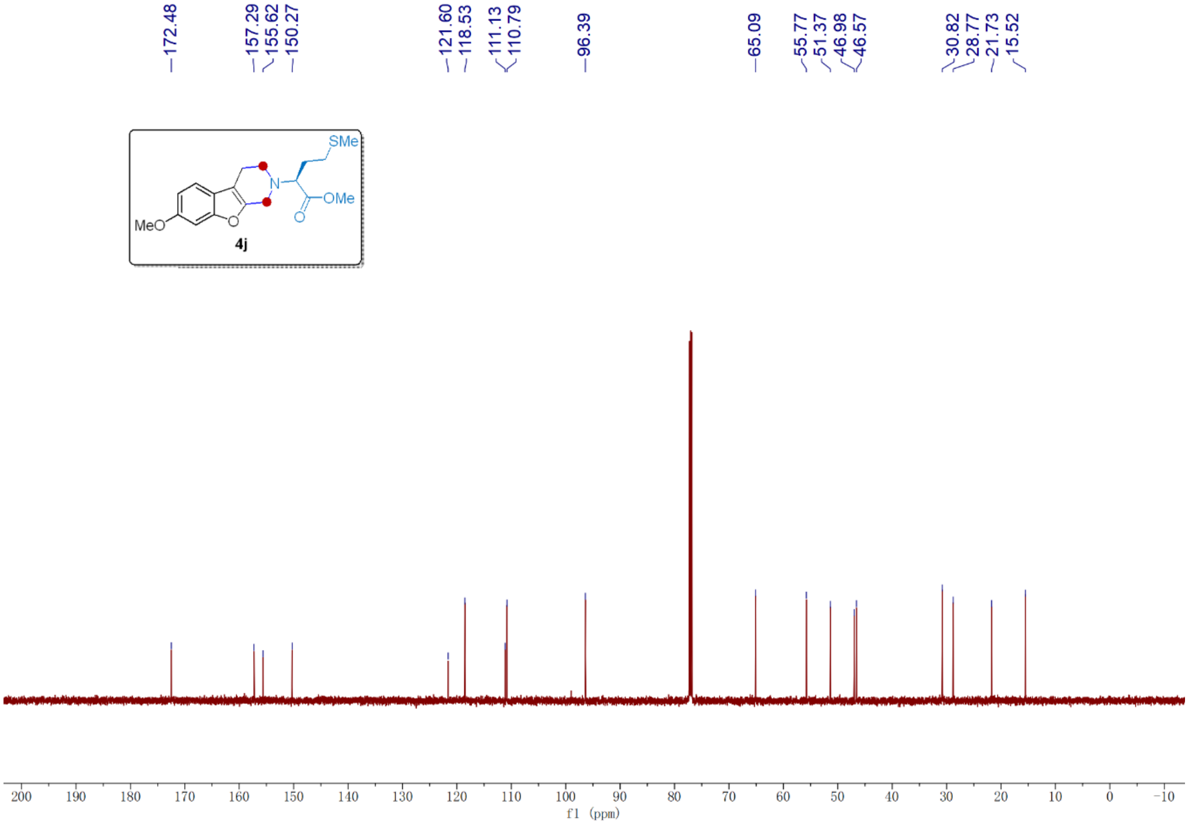

Supplementary Figure 52. ^{1}H-NMR spectrum of compound **4k** (600 MHz, $CDCl_3$)

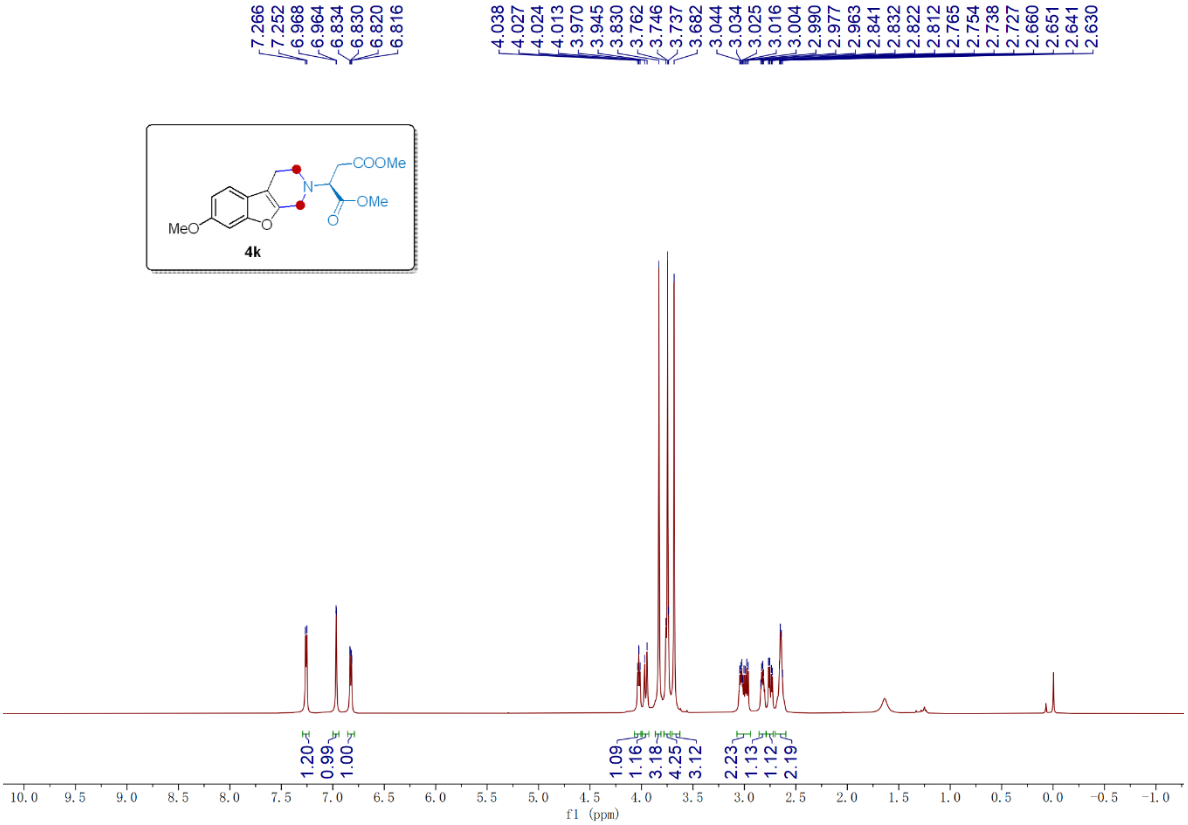

Supplementary Figure 53. ^{13}C-NMR spectrum of compound **4k** (150 MHz, $CDCl_3$)

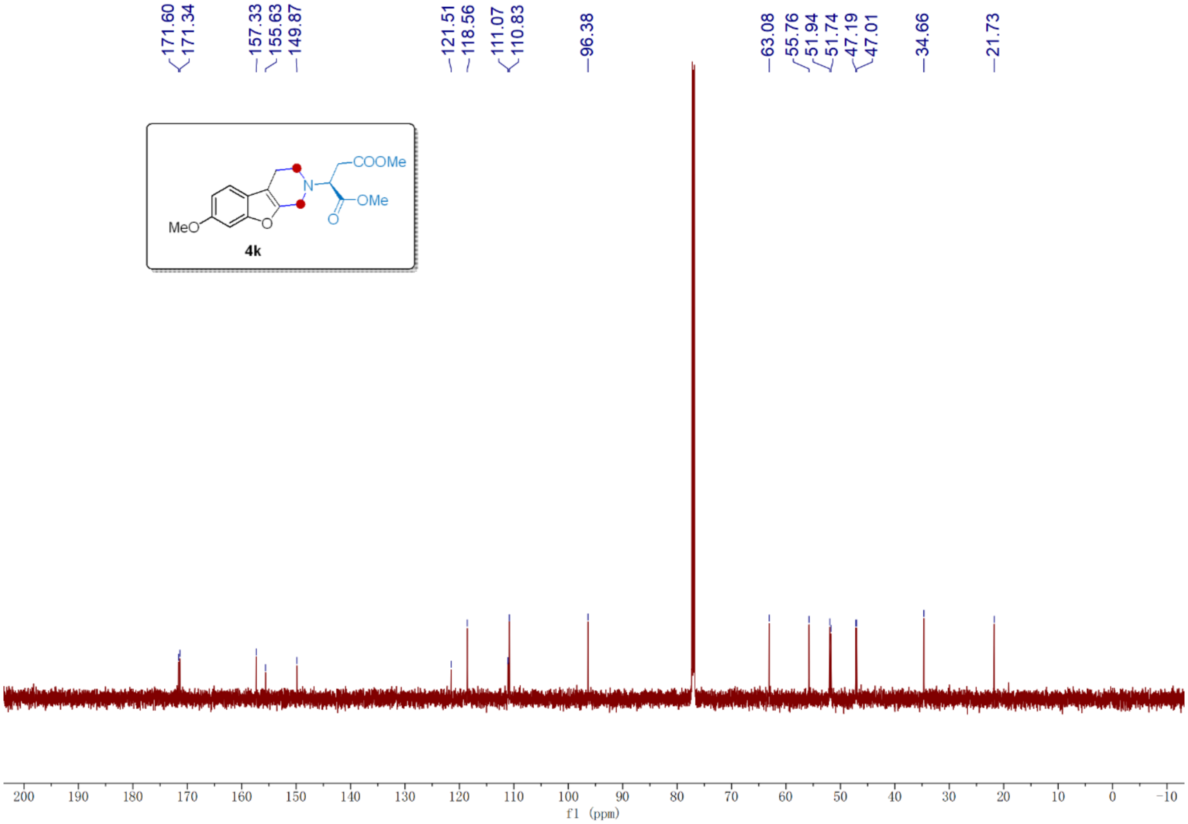

Supplementary Figure 54. ^{1}H-NMR spectrum of compound **4l** (600 MHz, $CDCl_3$)

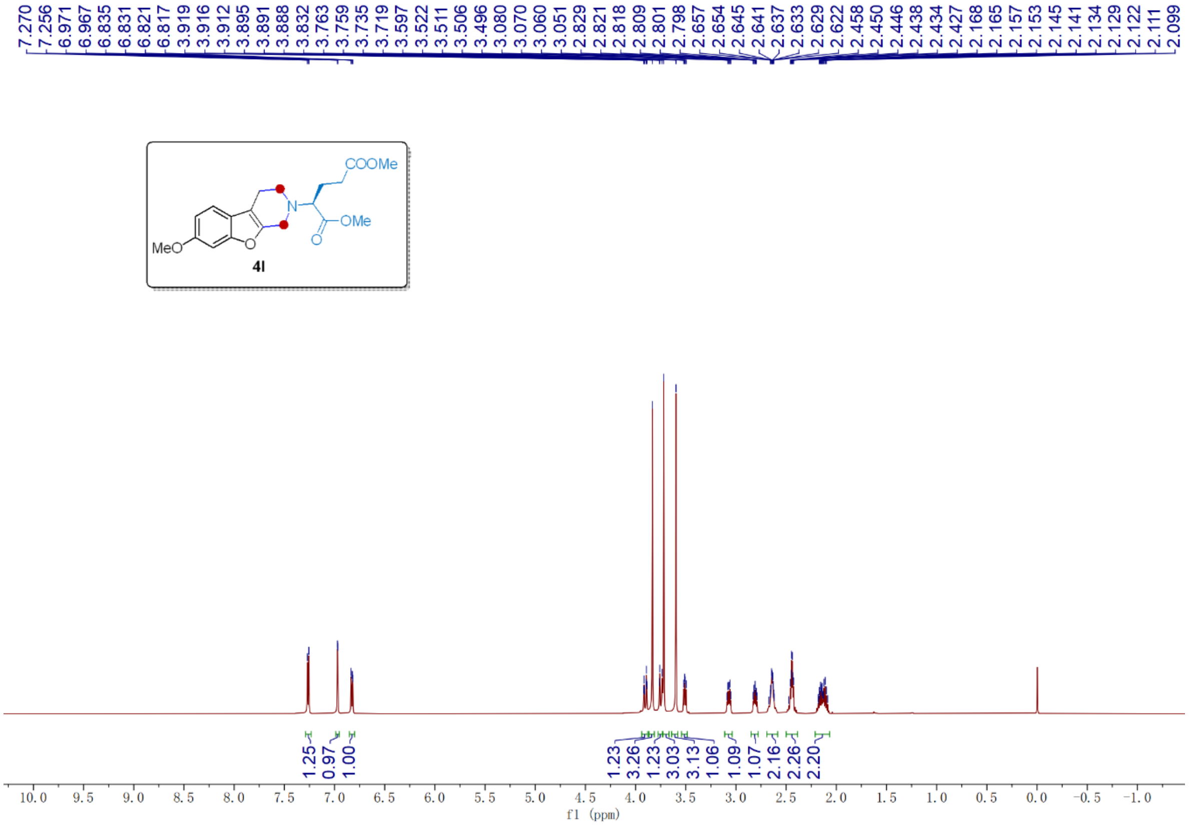

Supplementary Figure 55. ^{13}C-NMR spectrum of compound **4l** (150 MHz, $CDCl_3$)

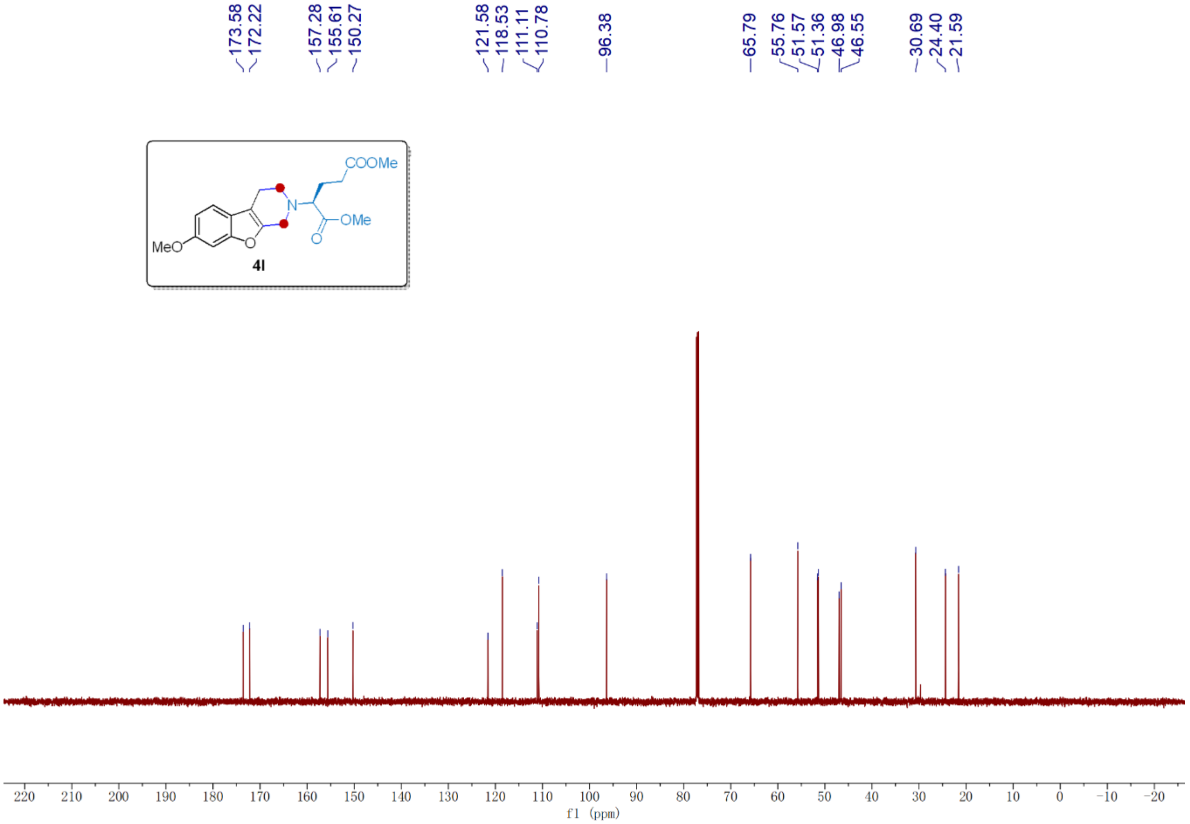

Supplementary Figure 56. ^{1}H-NMR spectrum of compound **4m** (600 MHz, $CDCl_3$)

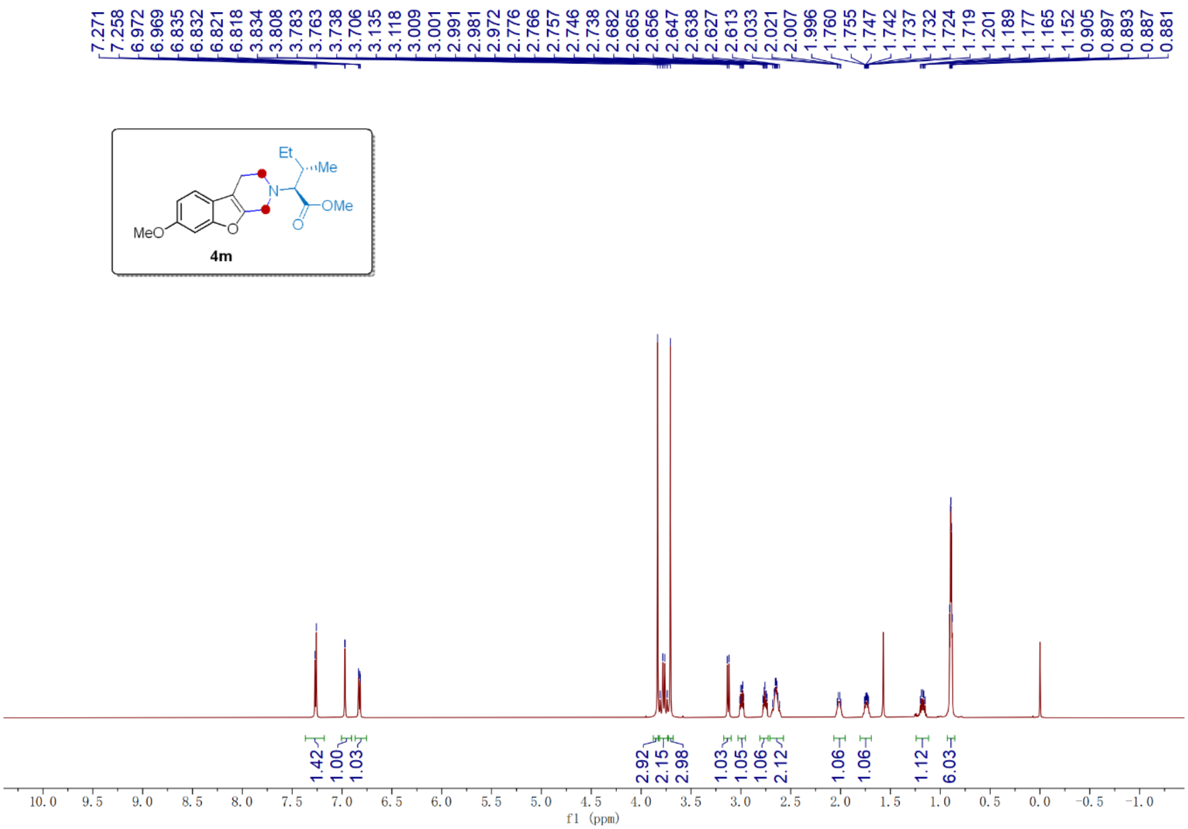

Supplementary Figure 57. ^{13}C-NMR spectrum of compound **4m** (150 MHz, $CDCl_3$)

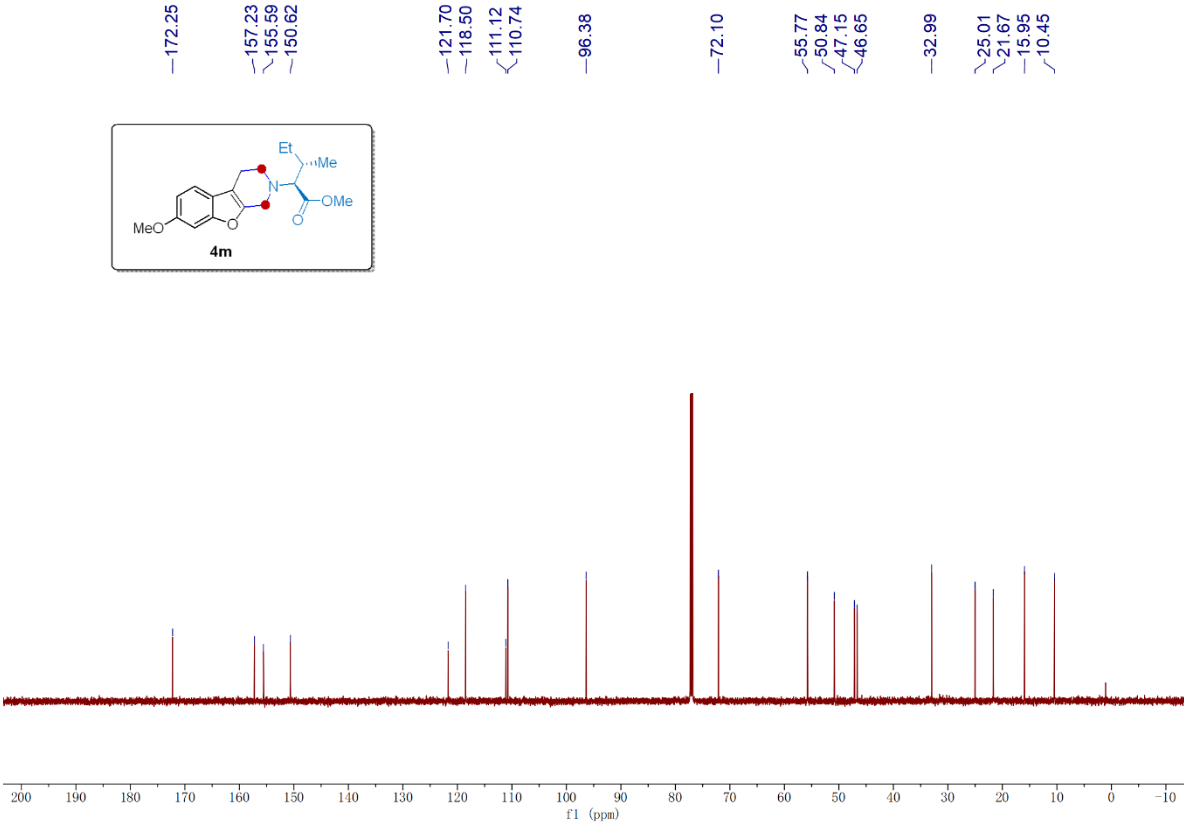

Supplementary Figure 58. ^{1}H-NMR spectrum of compound **4n** (600 MHz, $CDCl_3$)

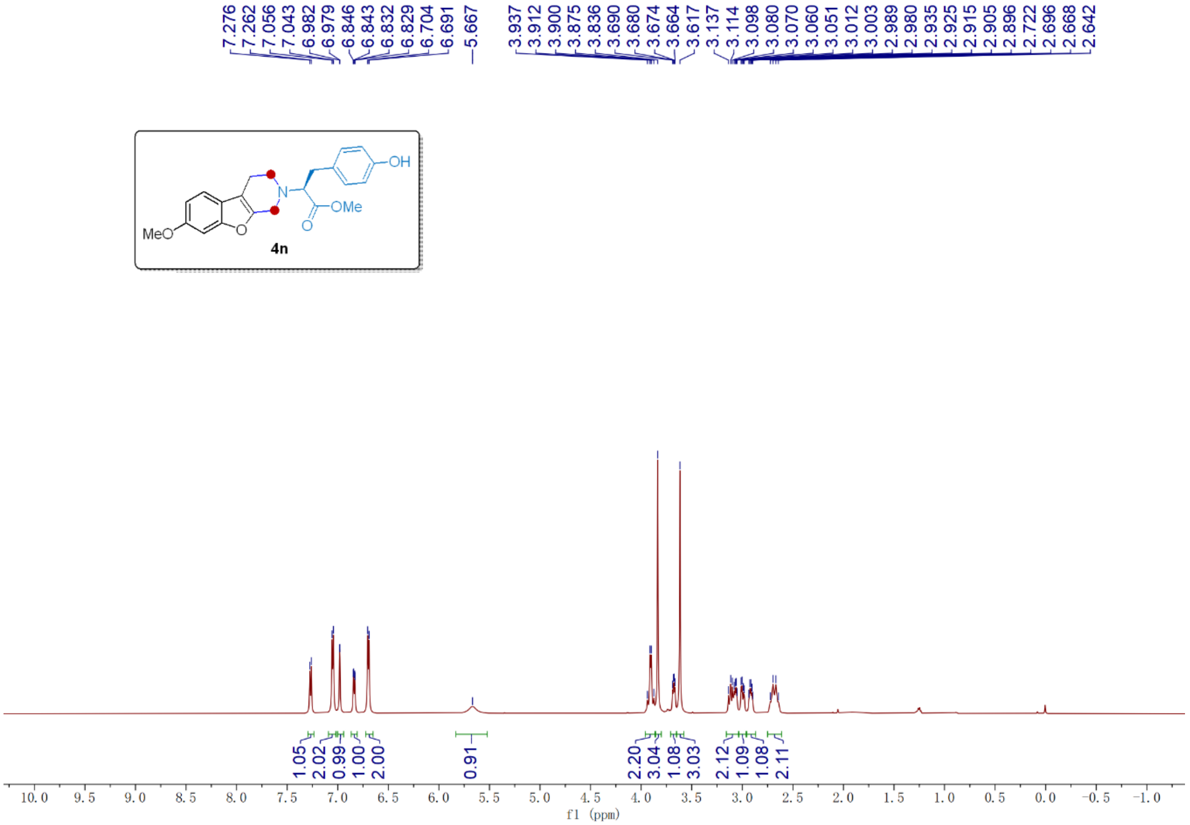

Supplementary Figure 59. ^{13}C-NMR spectrum of compound **4n** (150 MHz, $CDCl_3$)

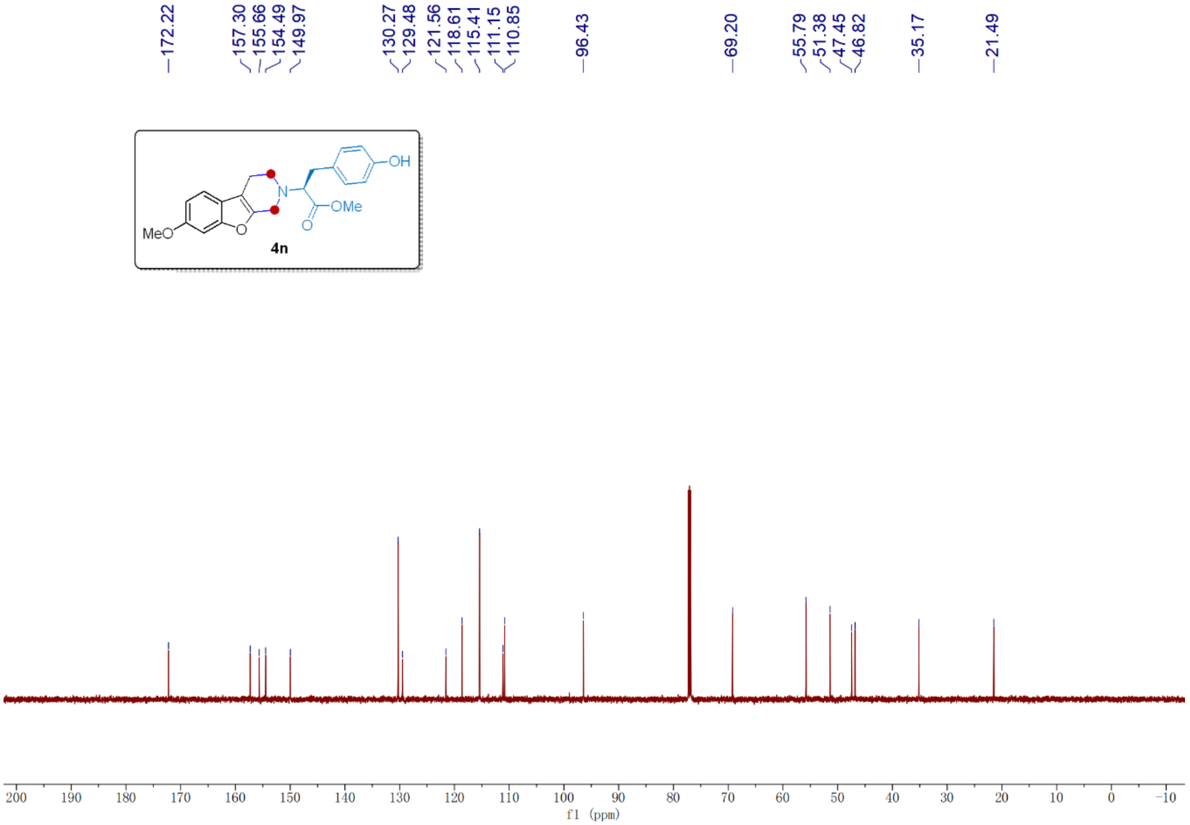

Supplementary Figure 60. ^{1}H-NMR spectrum of compound **4o** (400 MHz, $CDCl_3$)

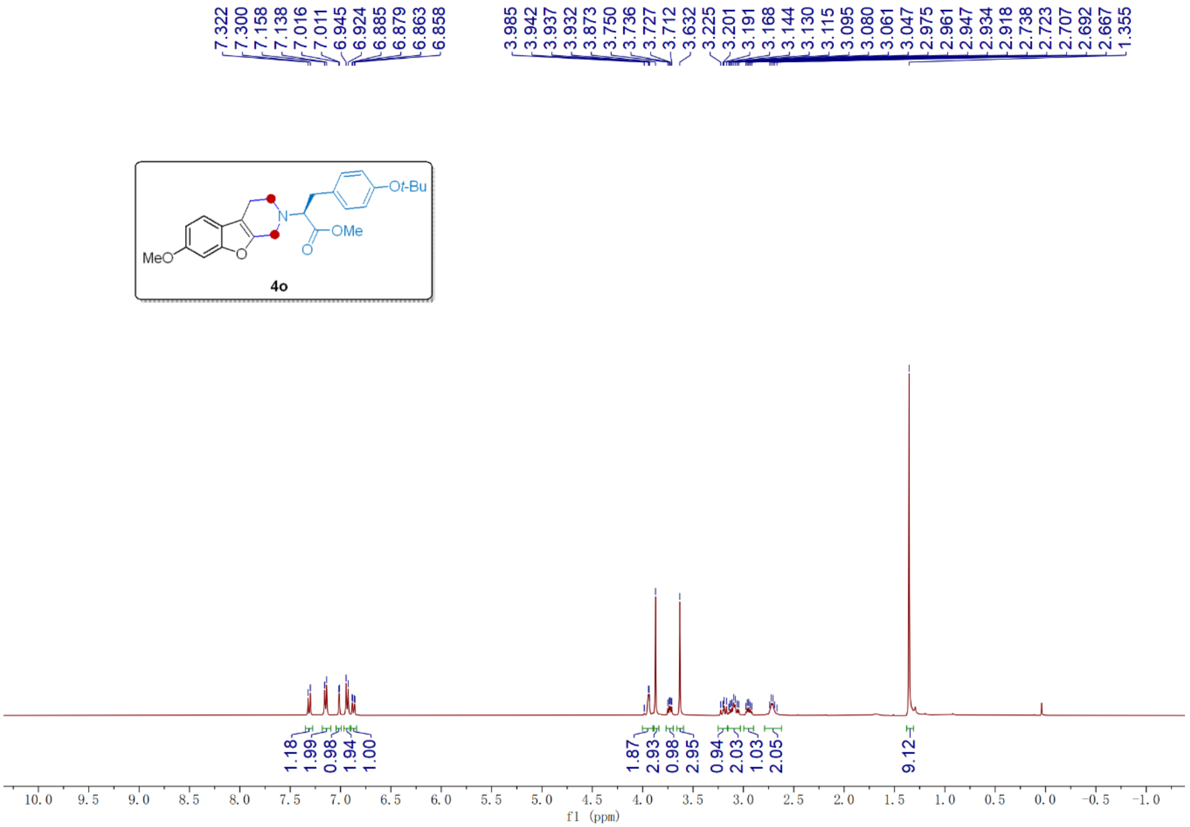

Supplementary Figure 61. ^{13}C-NMR spectrum of compound **4o** (100 MHz, $CDCl_3$)

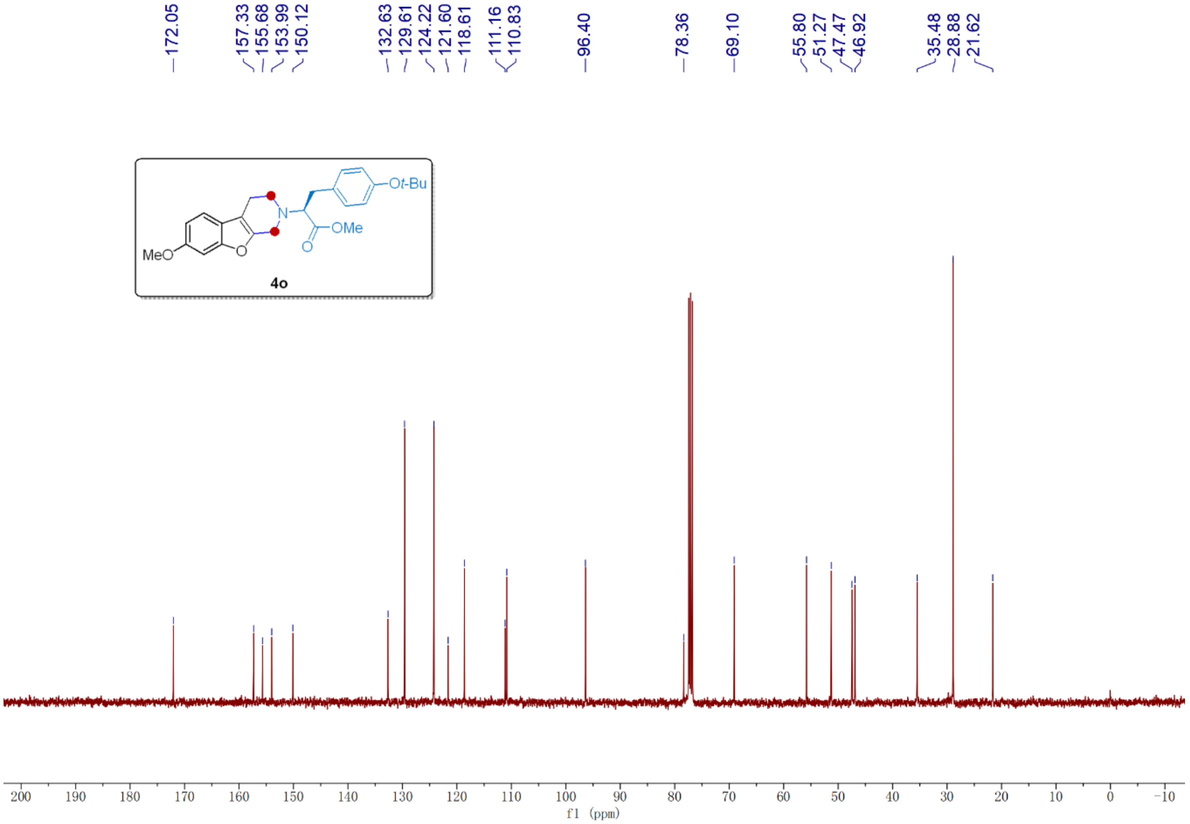

Supplementary Figure 62. ^{1}H-NMR spectrum of compound **4p** (600 MHz, $CDCl_3$)

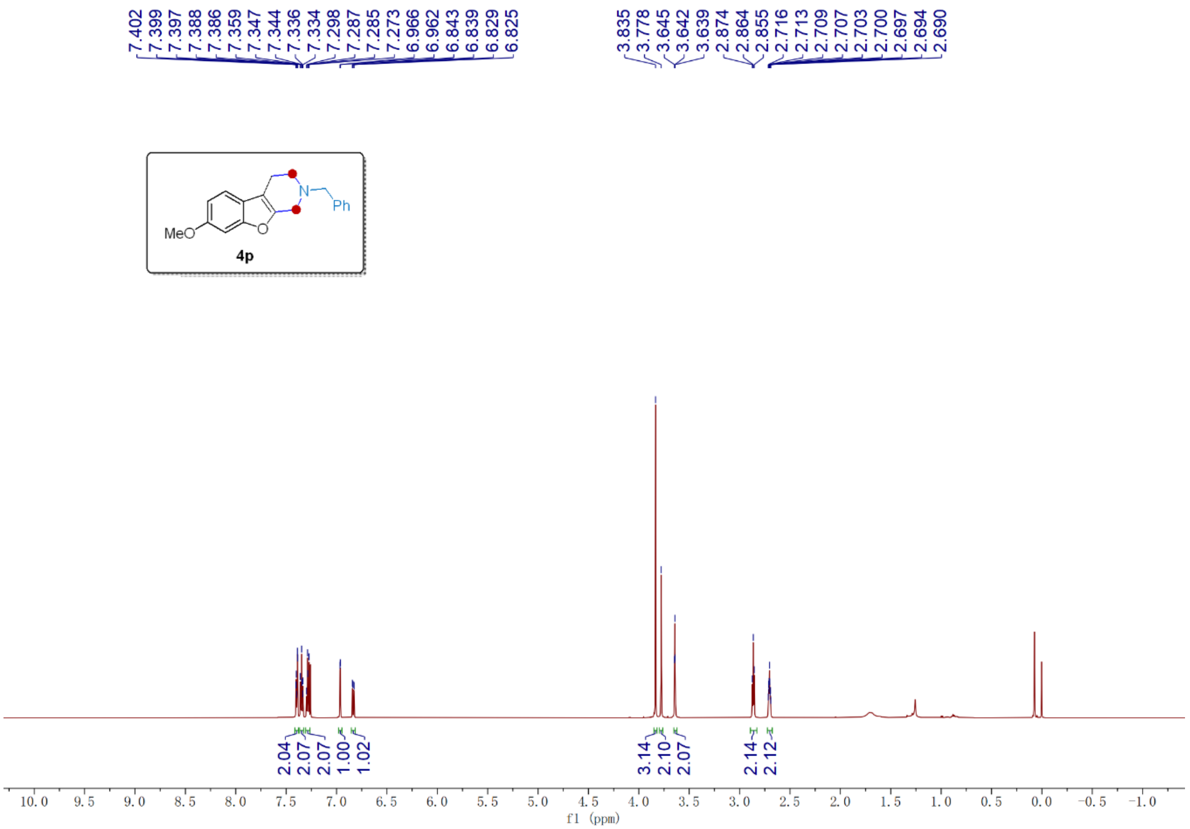

Supplementary Figure 63. ^{13}C-NMR spectrum of compound **4p** (100 MHz, $CDCl_3$)

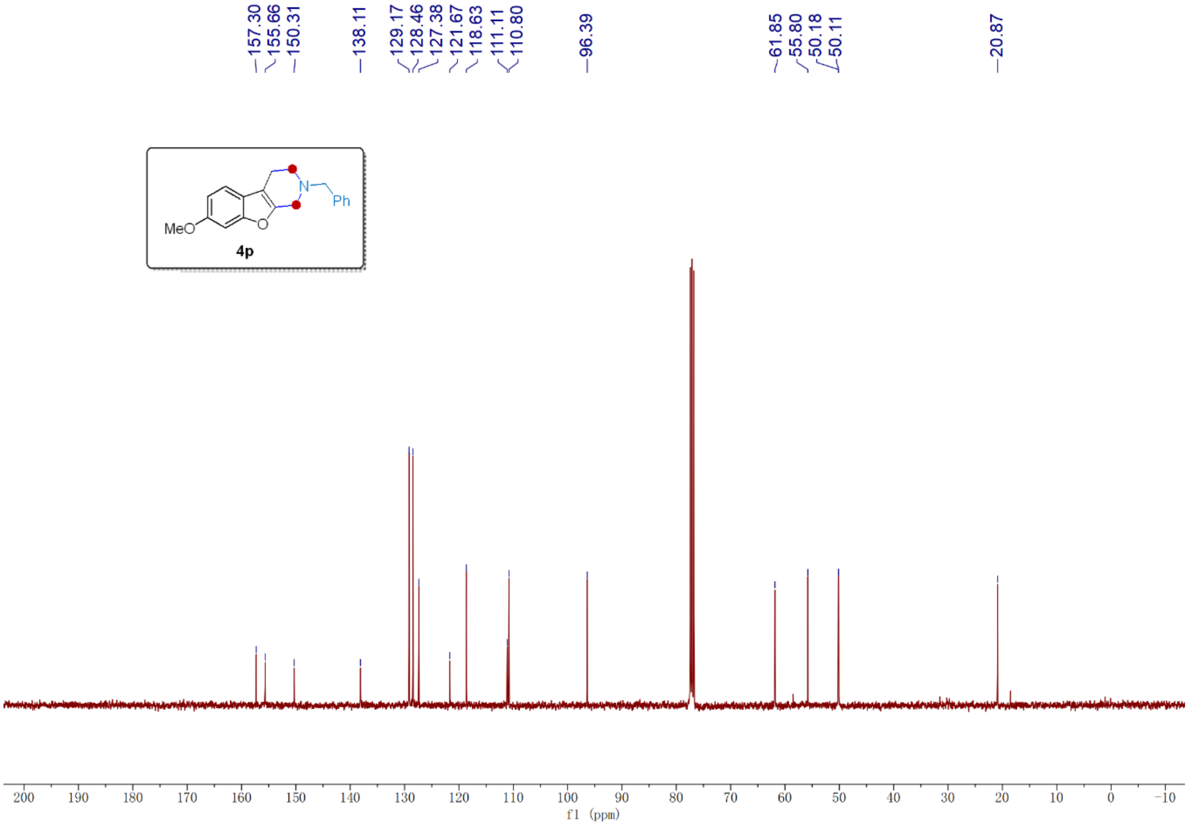

Supplementary Figure 64. ^{1}H-NMR spectrum of compound **4q** (400 MHz, $CDCl_3$)

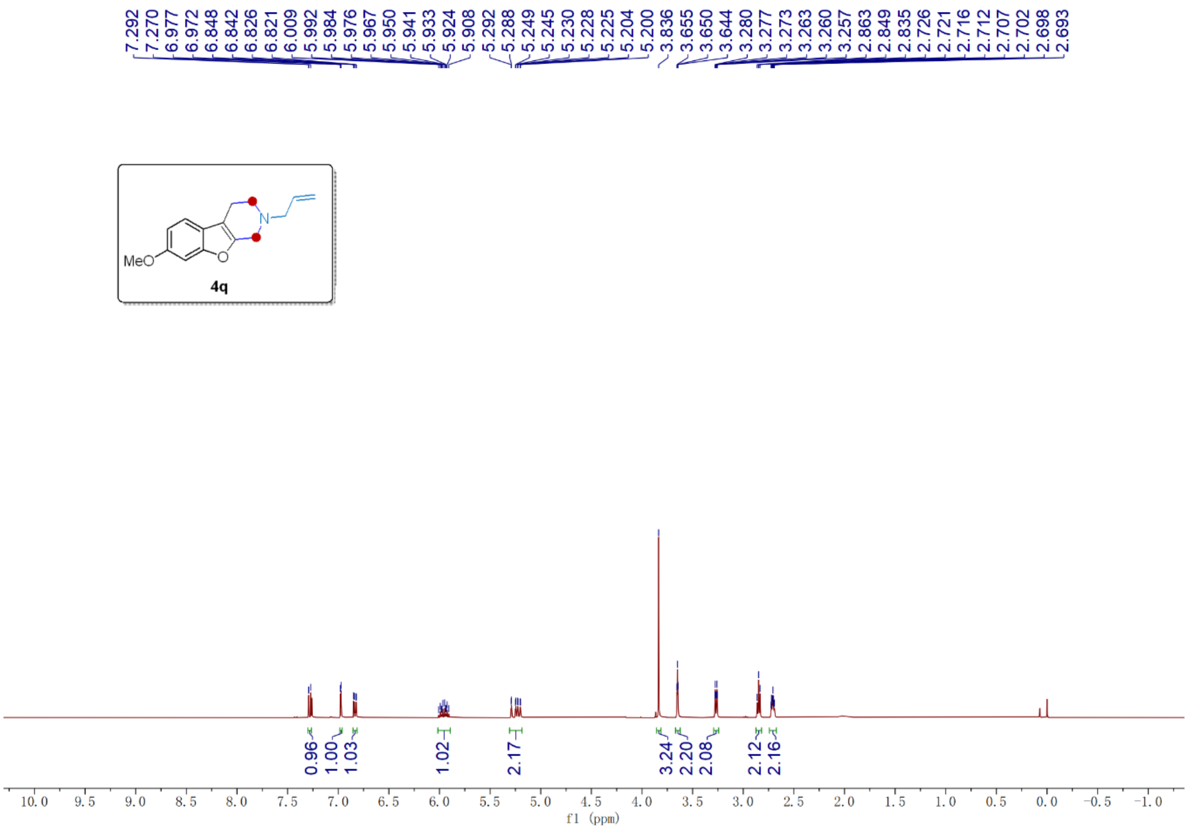

Supplementary Figure 65. ^{13}C-NMR spectrum of compound **4q** (100 MHz, $CDCl_3$)

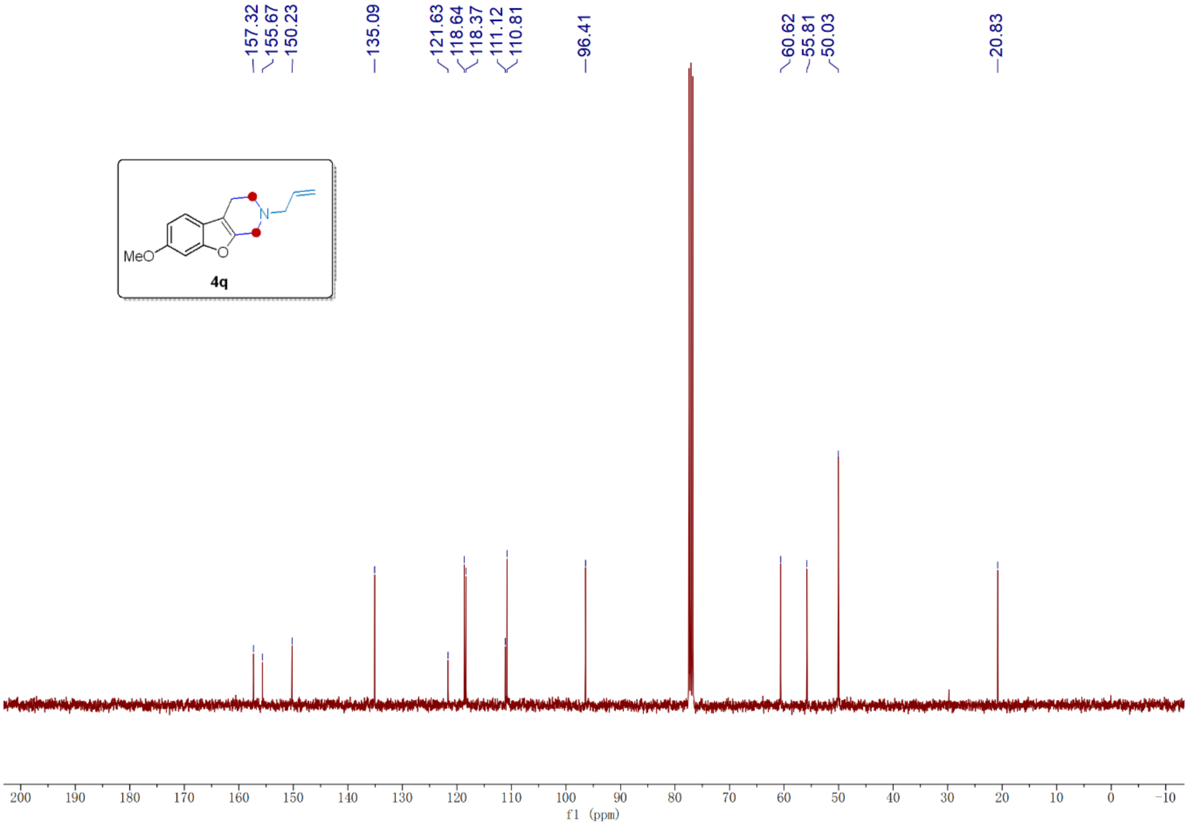

Supplementary Figure 66. ^{1}H-NMR spectrum of compound **4r** (400 MHz, $CDCl_3$)

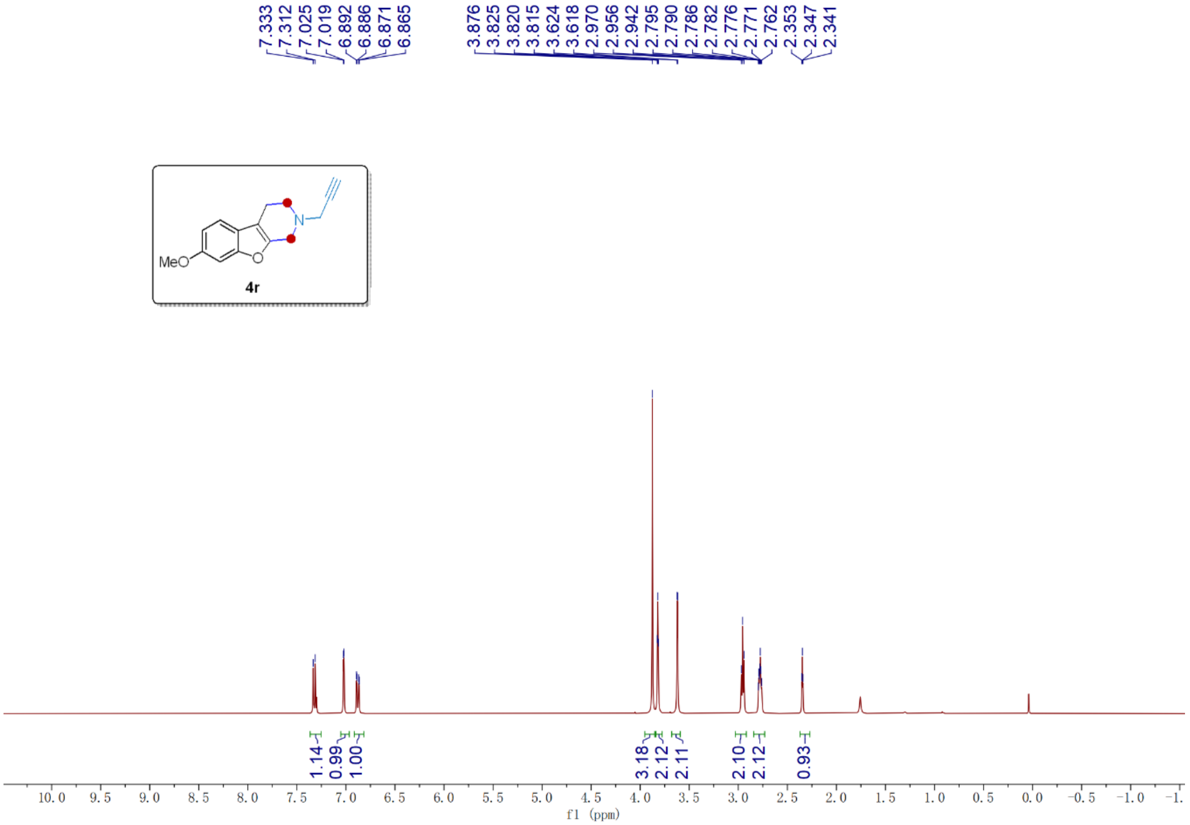

Supplementary Figure 67. ^{13}C-NMR spectrum of compound **4r** (100 MHz, $CDCl_3$)

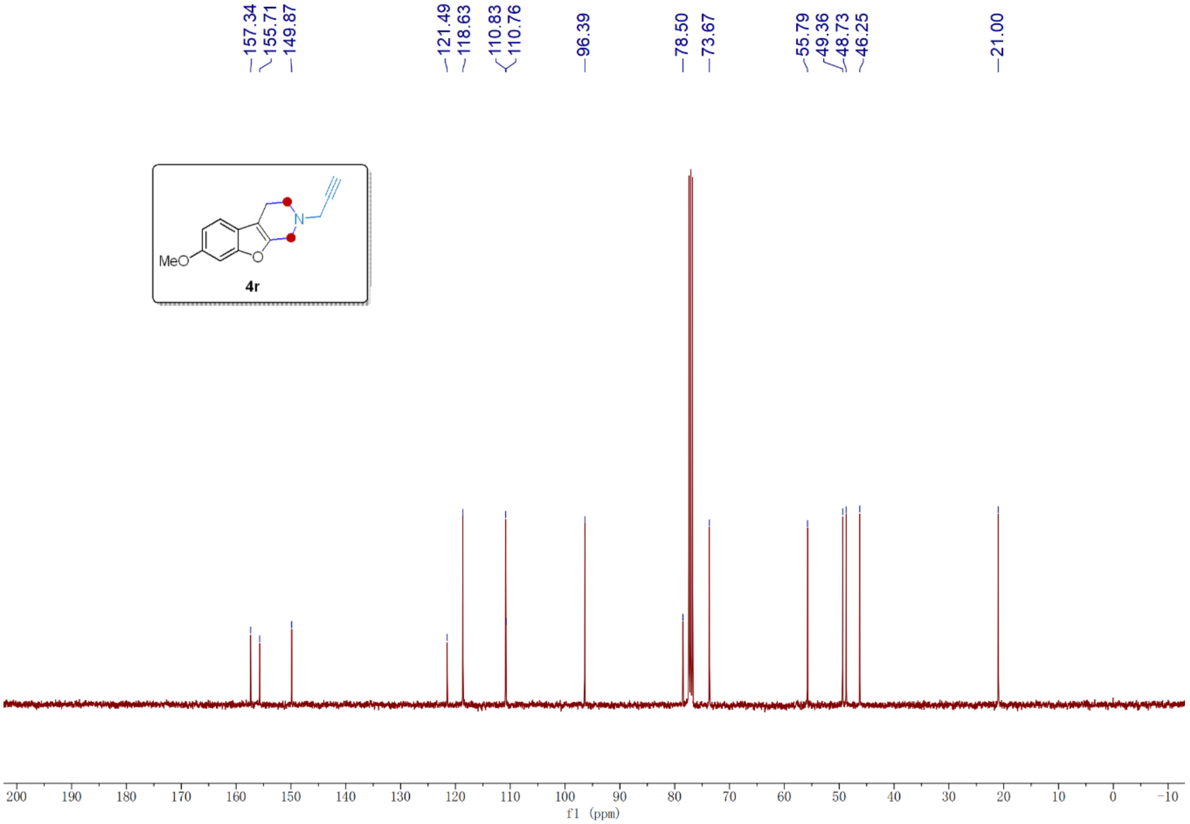

Supplementary Figure 68. ^{1}H-NMR spectrum of compound **4s** (600 MHz, $CDCl_3$)

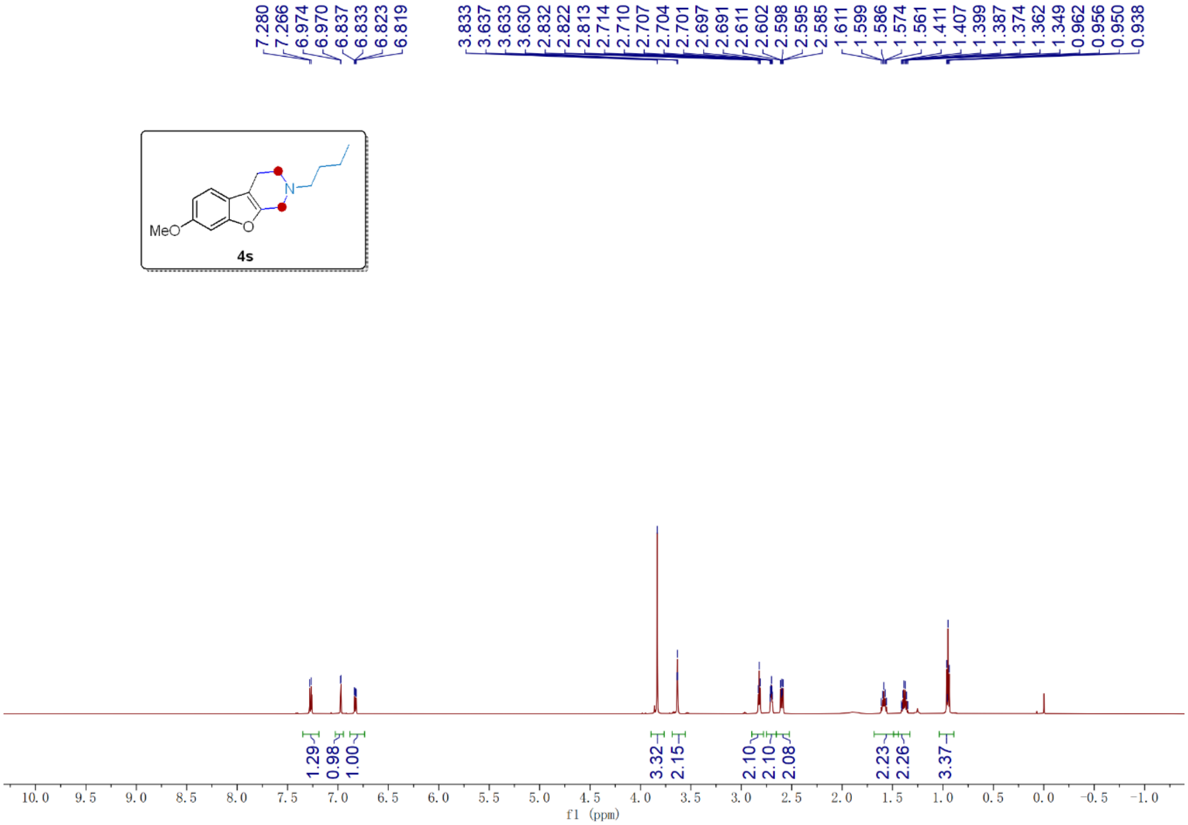

Supplementary Figure 69. ^{13}C-NMR spectrum of compound **4s** (125 MHz, $CDCl_3$)

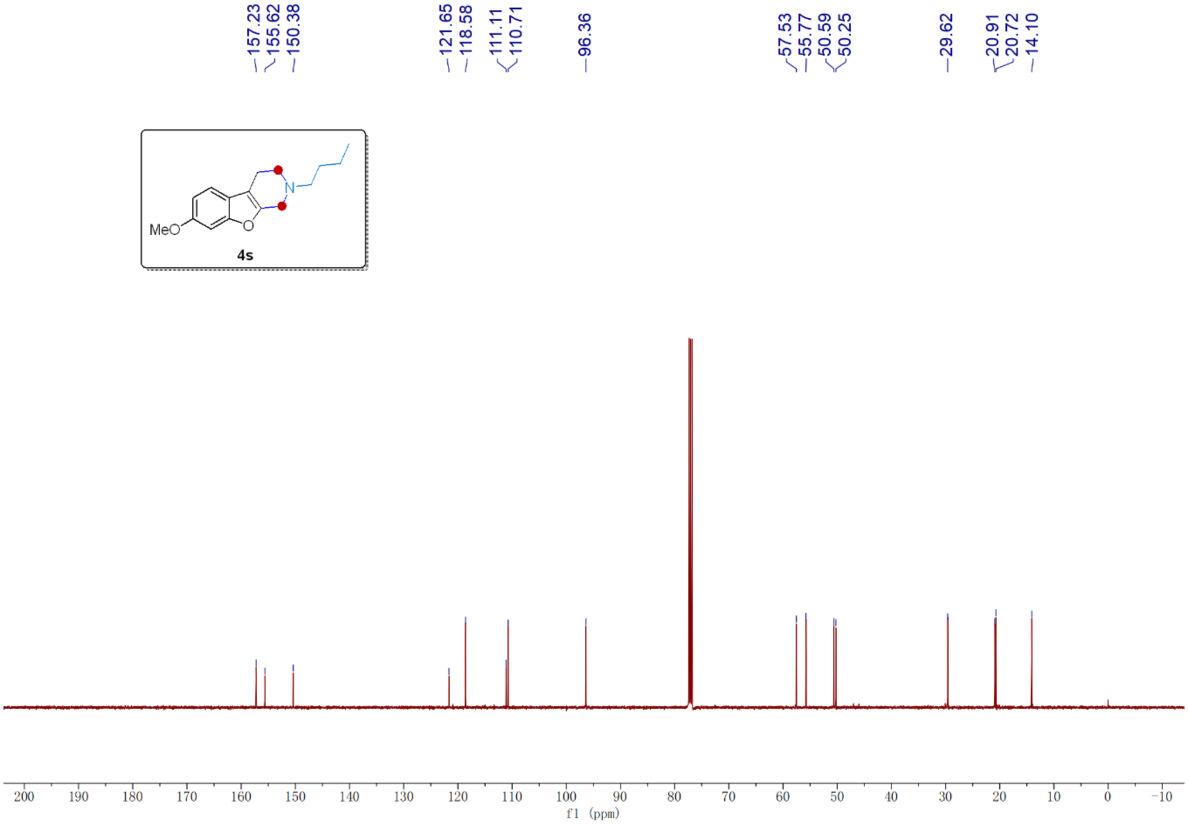

Supplementary Figure 70. ^{1}H-NMR spectrum of compound **4t** (500 MHz, $CDCl_3$)

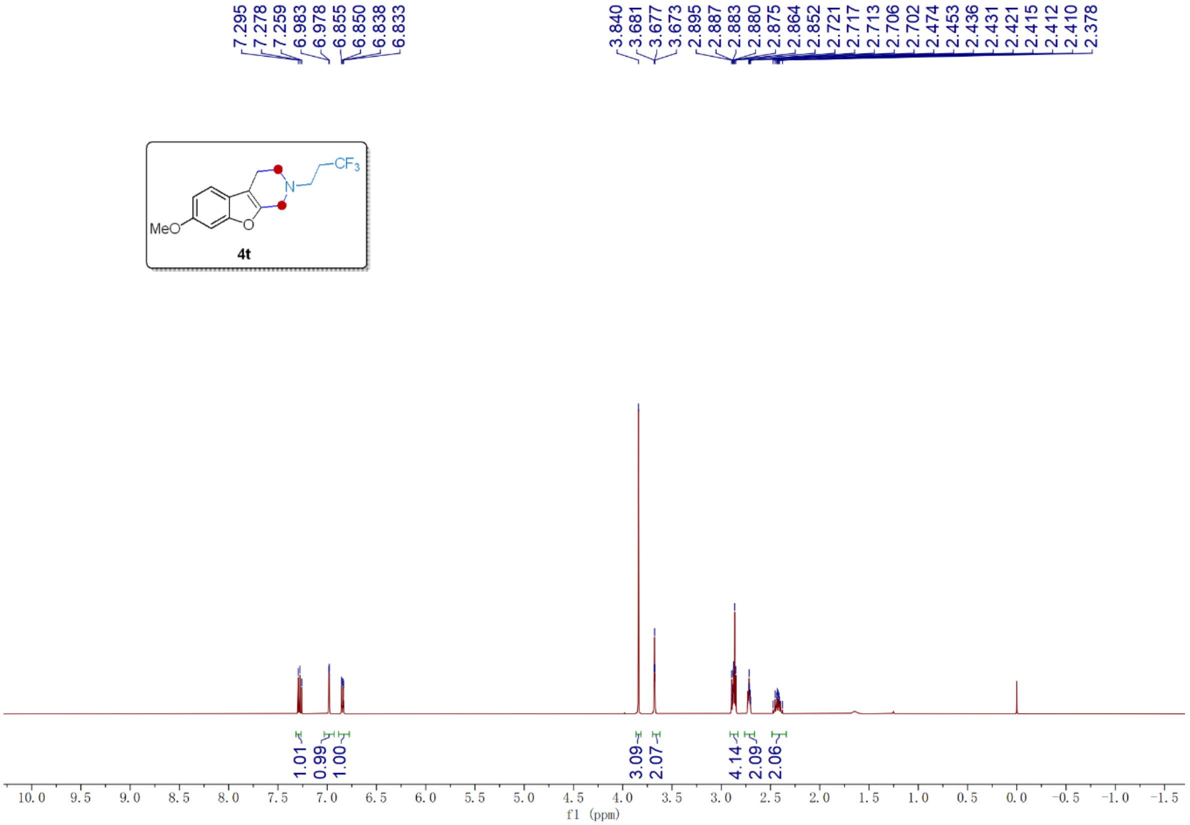

Supplementary Figure 71. ^{13}C-NMR spectrum of compound **4t** (125 MHz, $CDCl_3$)

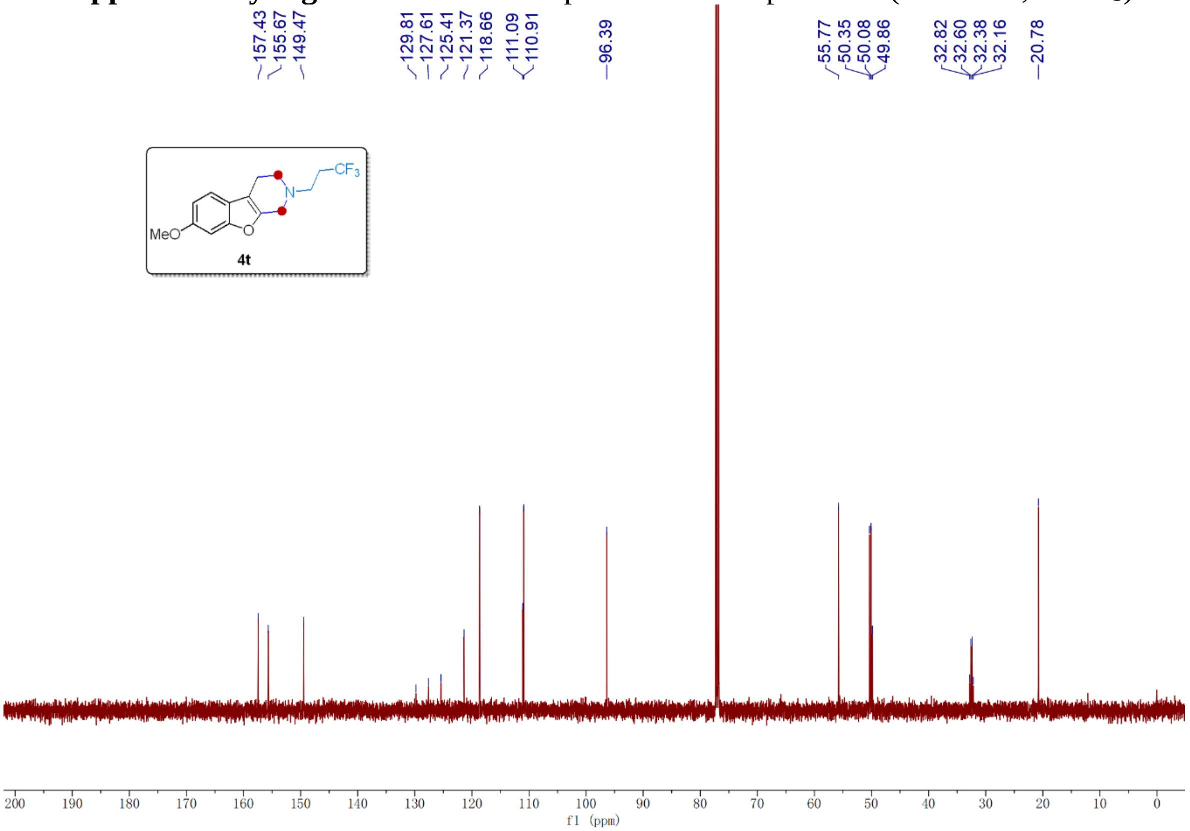

Supplementary Figure 72. ^{1}H-NMR spectrum of compound **4u** (400 MHz, $CDCl_3$)

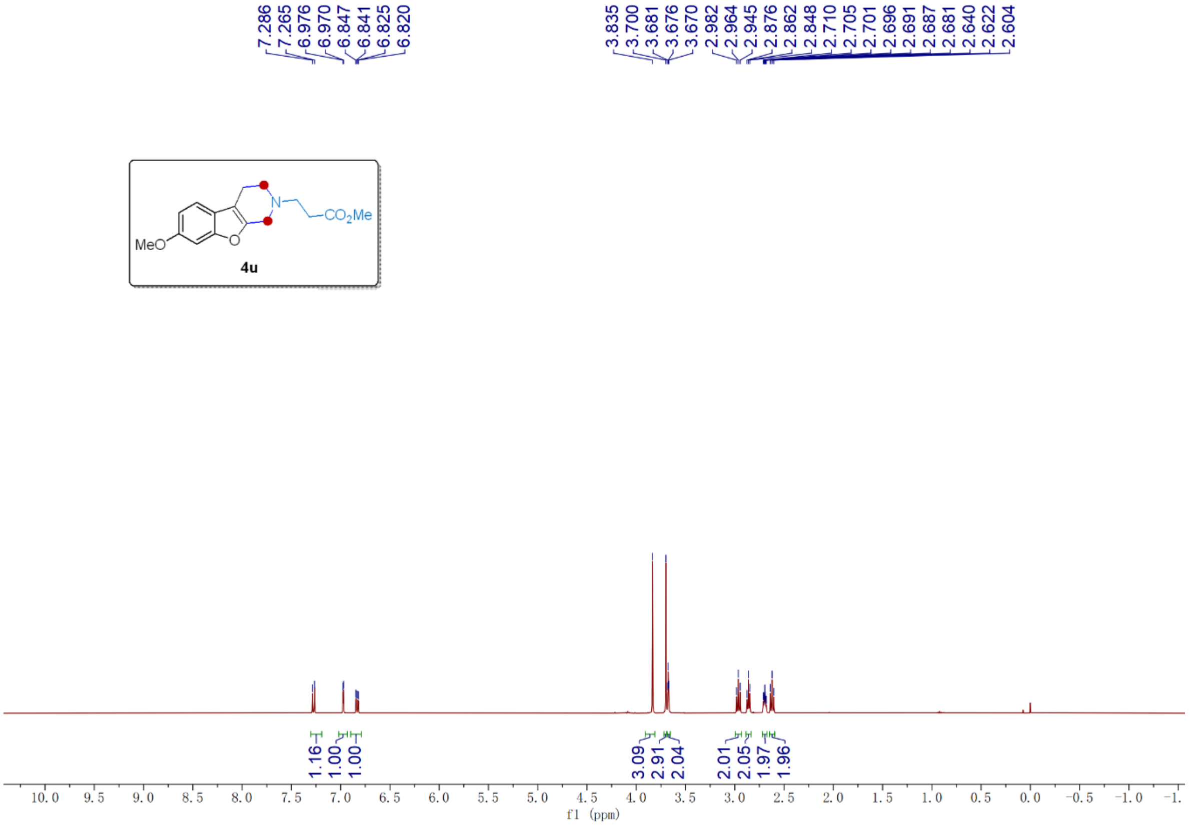

Supplementary Figure 73. ^{13}C-NMR spectrum of compound **4u** (100 MHz, $CDCl_3$)

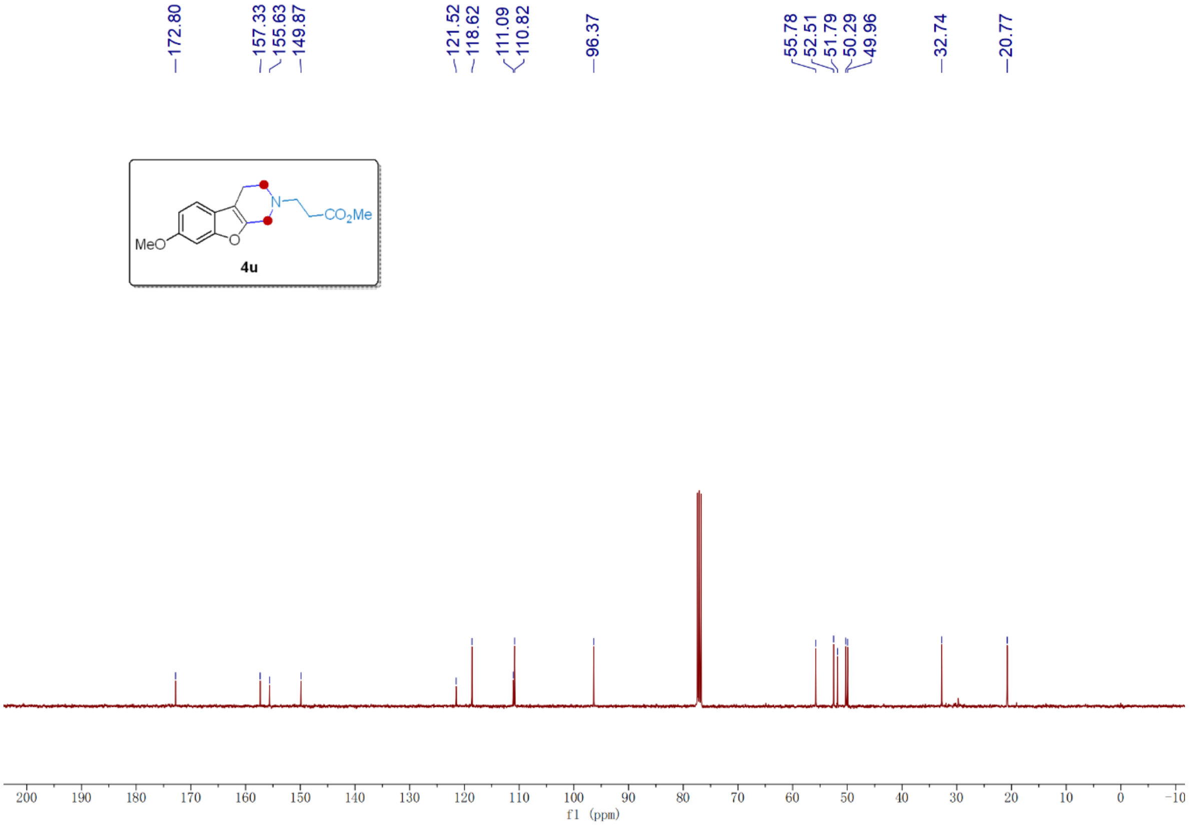

Supplementary Figure 74. ^{1}H-NMR spectrum of compound **4v** (500 MHz, $CDCl_3$)

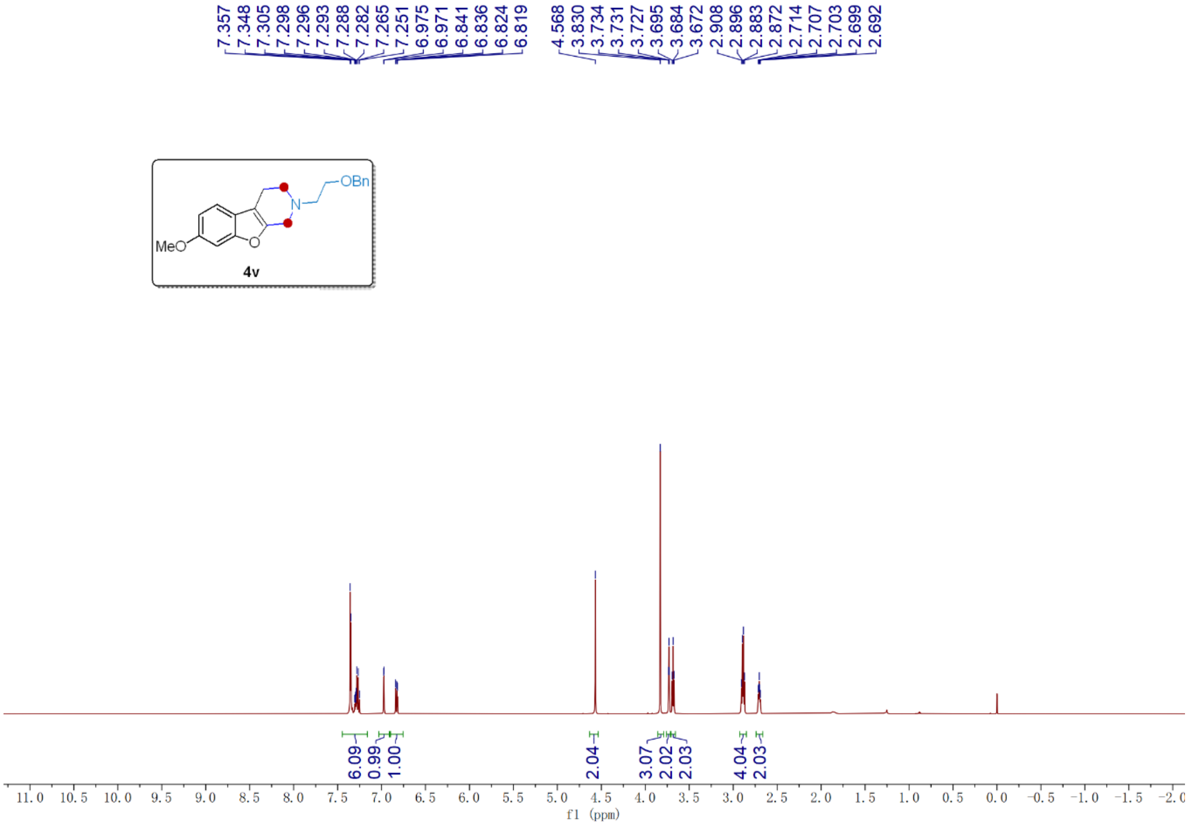

Supplementary Figure 75. ^{13}C-NMR spectrum of compound **4v** (125 MHz, $CDCl_3$)

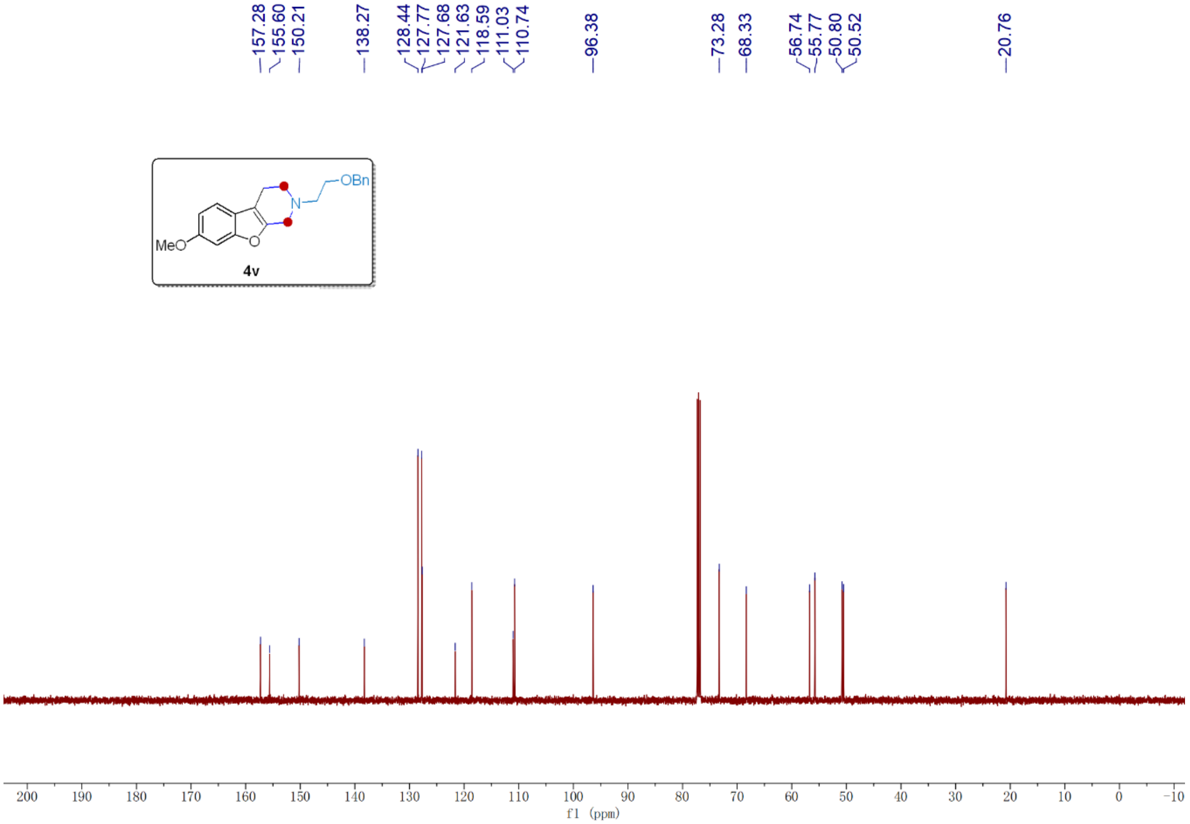

Supplementary Figure 76. ^{1}H-NMR spectrum of compound **4w** (500 MHz, $CDCl_3$)

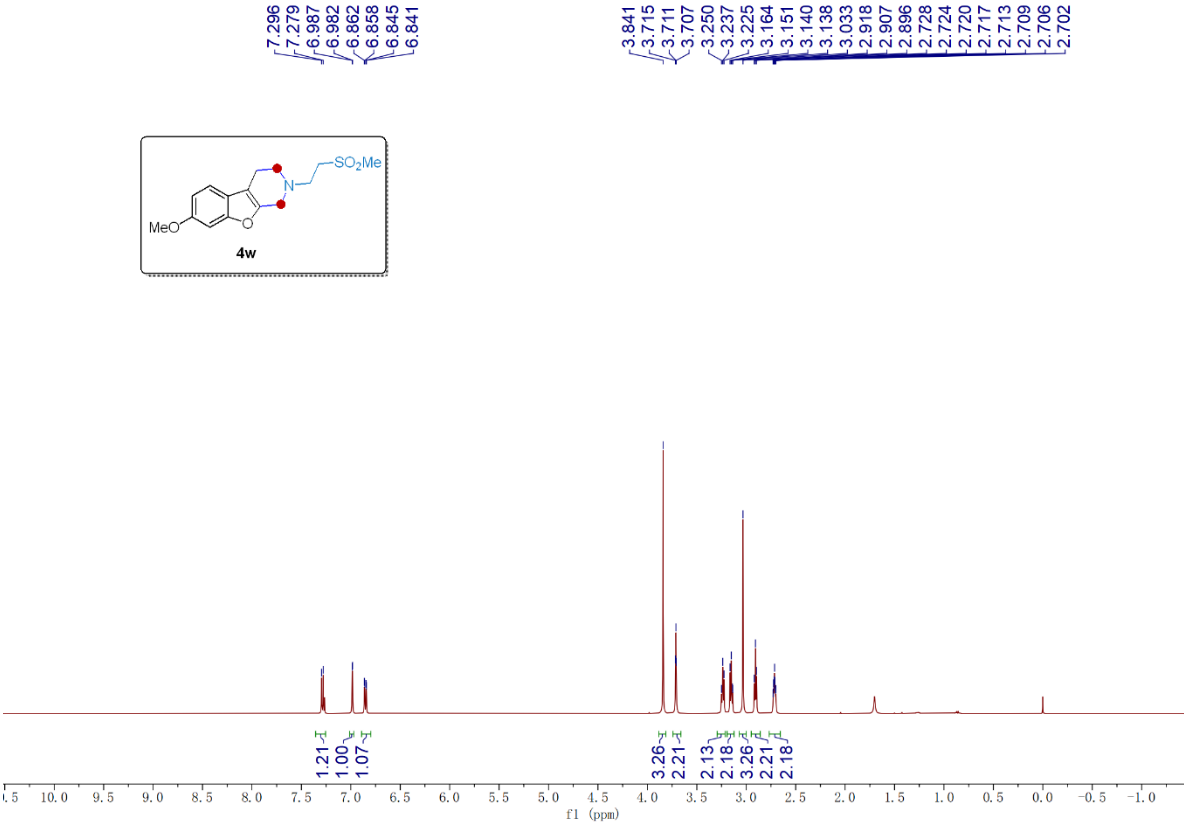

Supplementary Figure 77. ^{13}C-NMR spectrum of compound **4w** (100 MHz, $CDCl_3$)

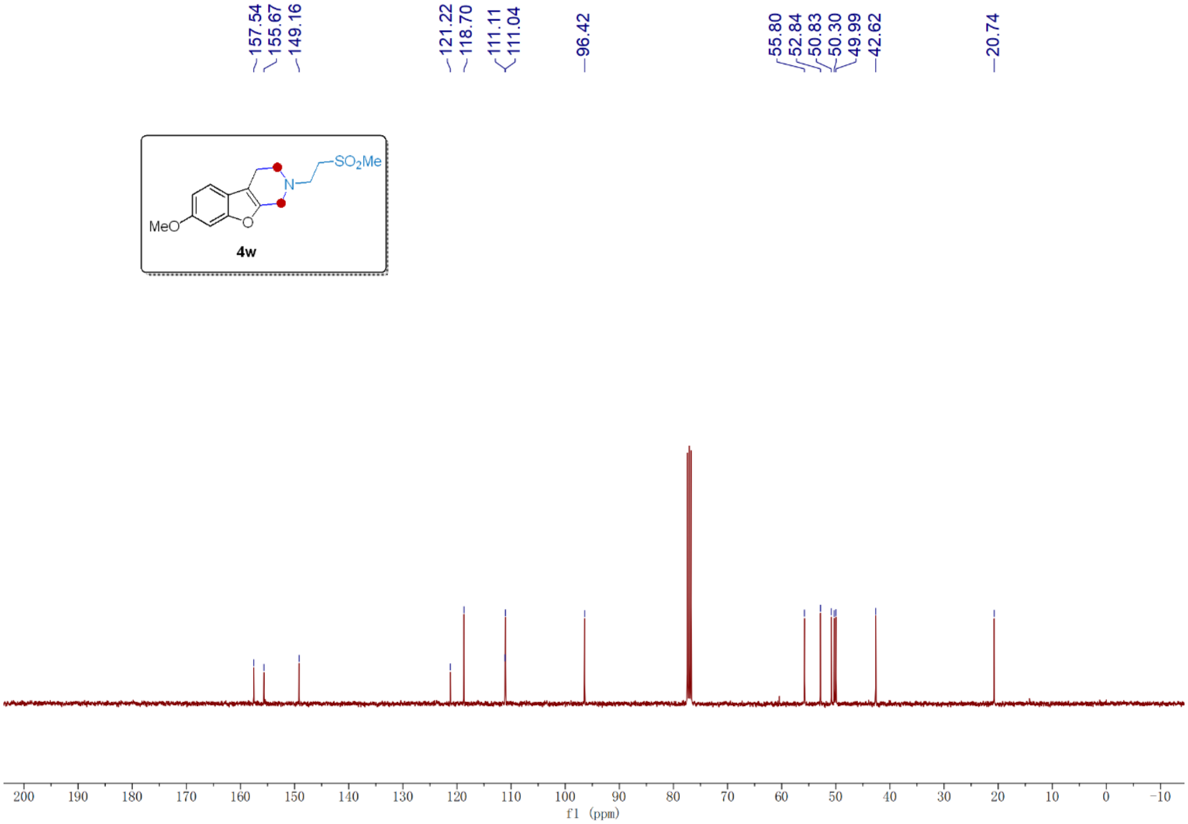

Supplementary Figure 78. ^{1}H-NMR spectrum of compound **4x** (500 MHz, $CDCl_3$)

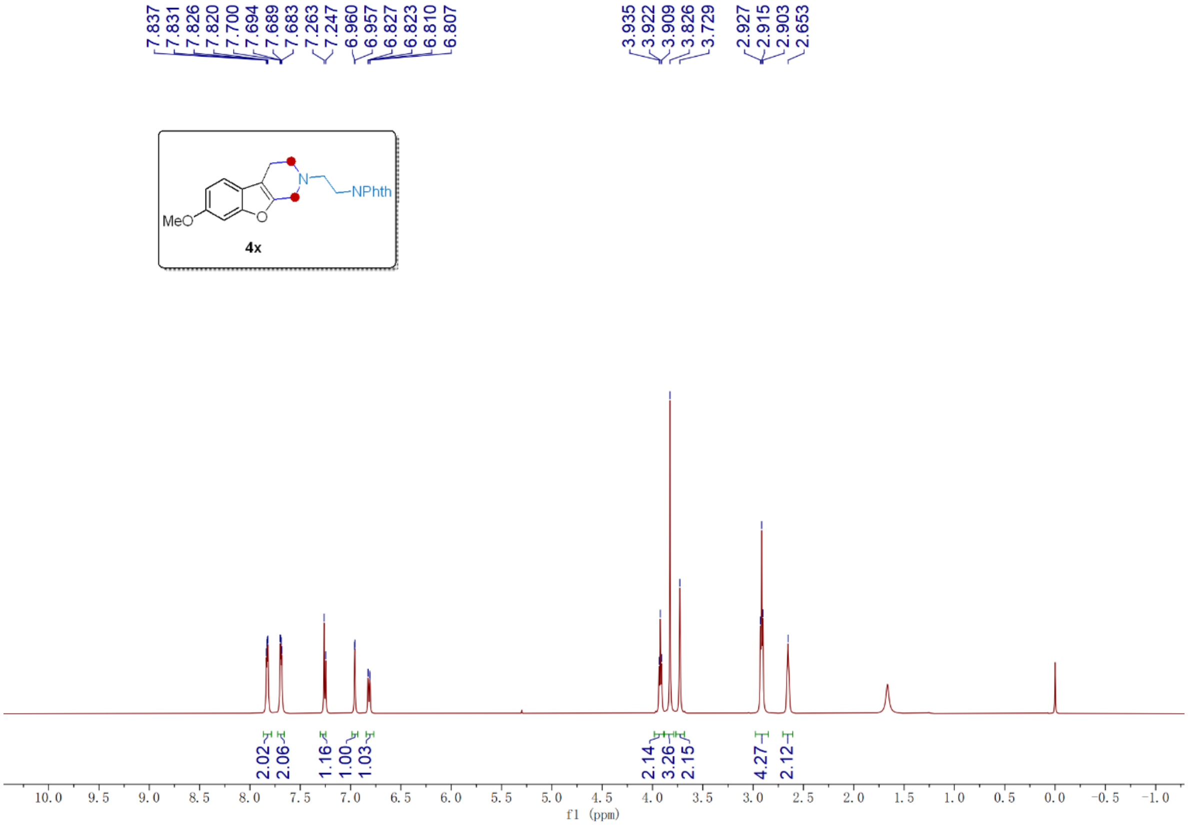

Supplementary Figure 79. ^{13}C-NMR spectrum of compound **4x** (125 MHz, $CDCl_3$)

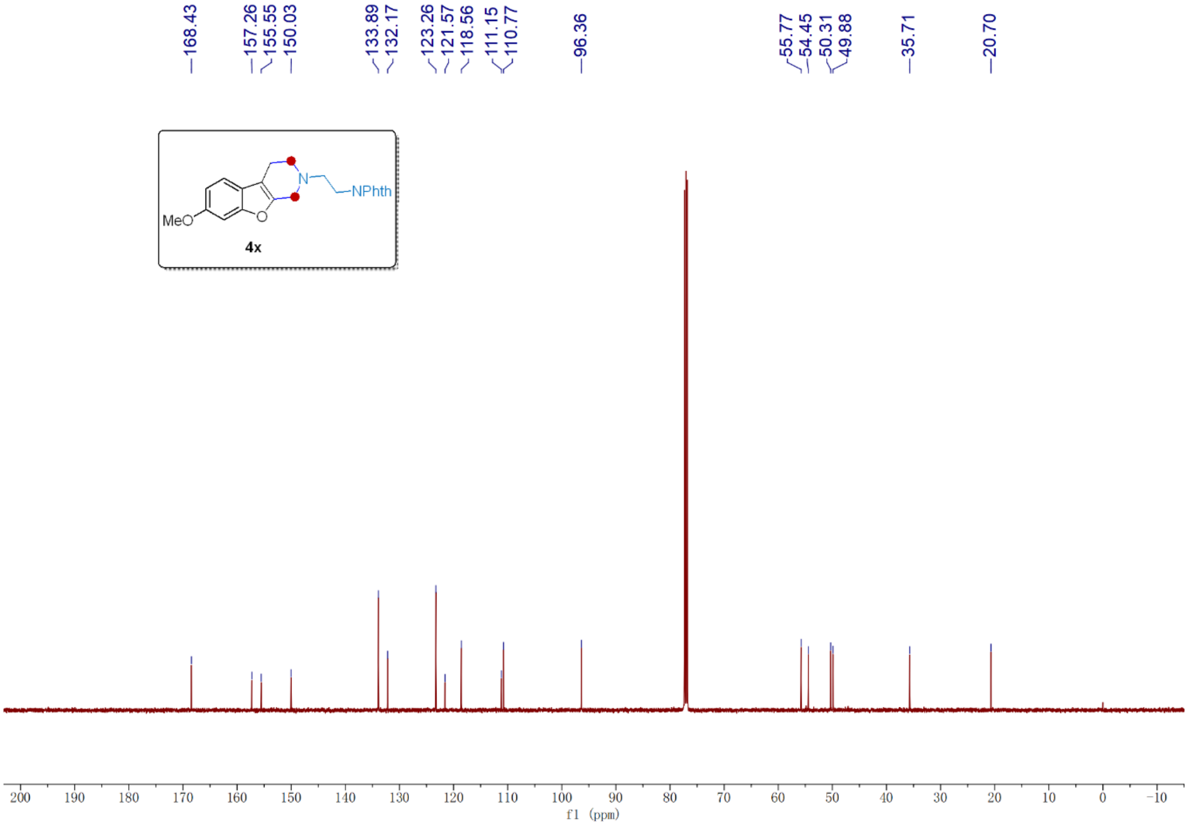

Supplementary Figure 80. ^{1}H-NMR spectrum of compound **4y** (500 MHz, $CDCl_3$)

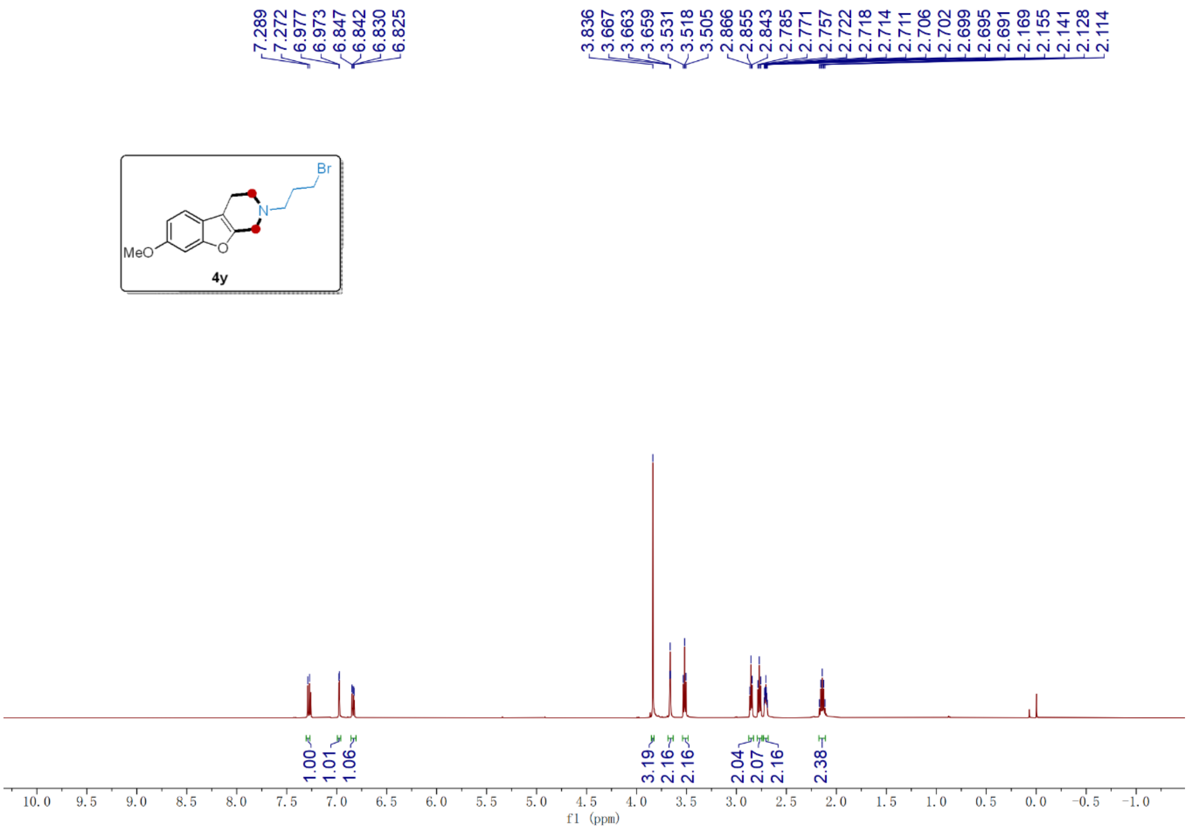

Supplementary Figure 81. ^{13}C-NMR spectrum of compound **4y** (125 MHz, $CDCl_3$)

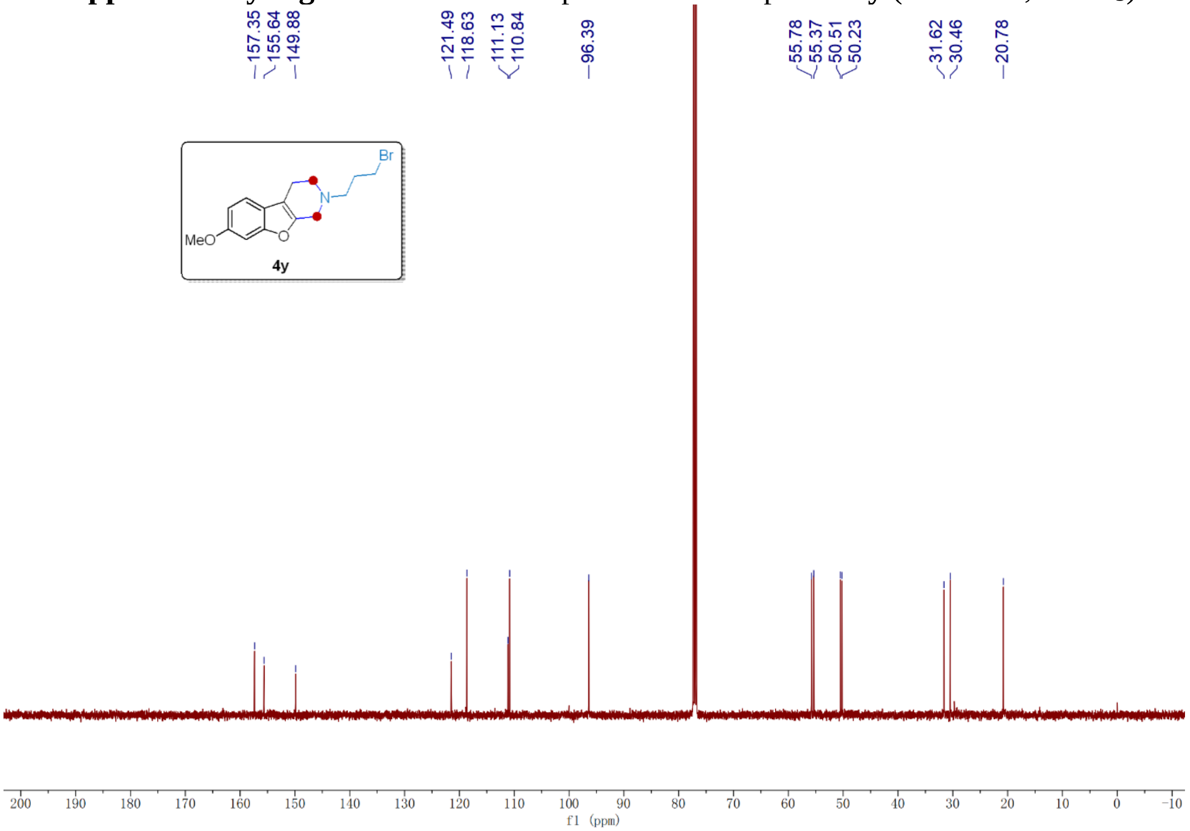

Supplementary Figure 82. ^{1}H-NMR spectrum of compound **4z** (600 MHz, $CDCl_3$)

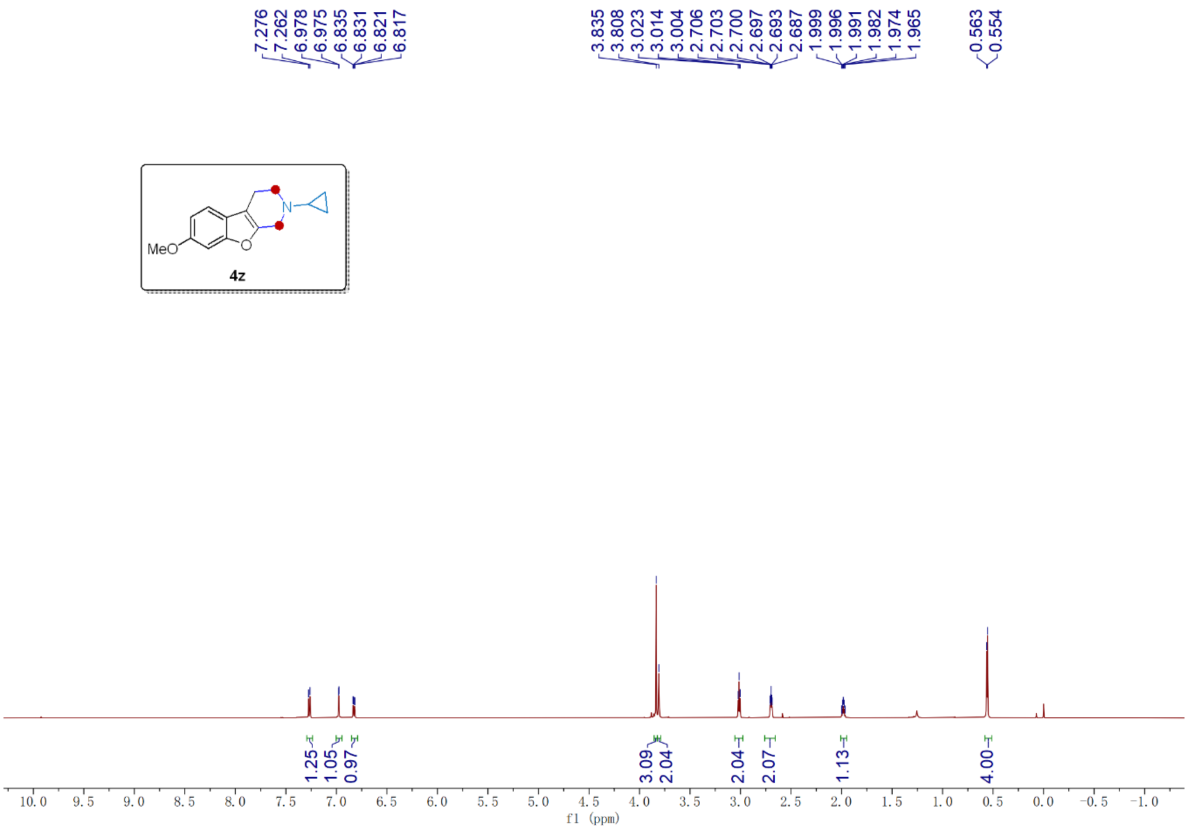

Supplementary Figure 83. ^{13}C-NMR spectrum of compound **4z** (150 MHz, $CDCl_3$)

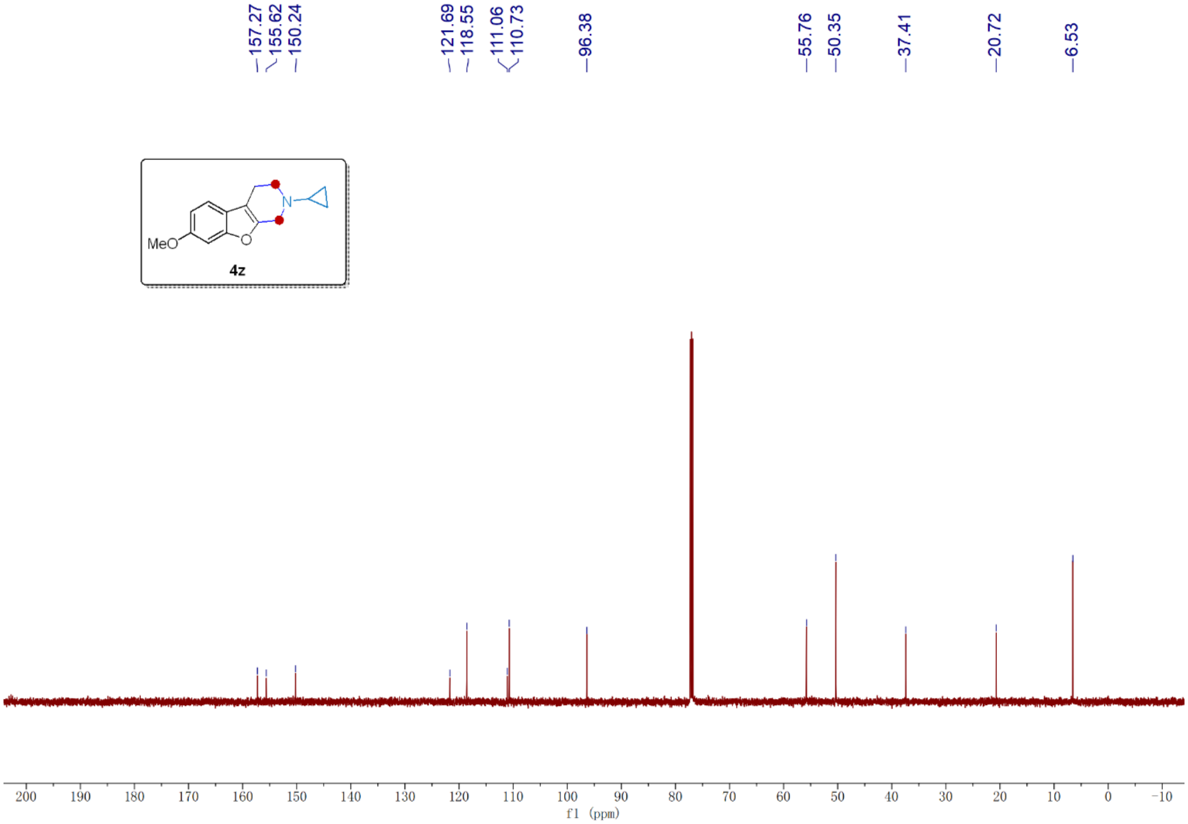

Supplementary Figure 84. ^{1}H-NMR spectrum of compound **4aa** (400 MHz, $CDCl_3$)

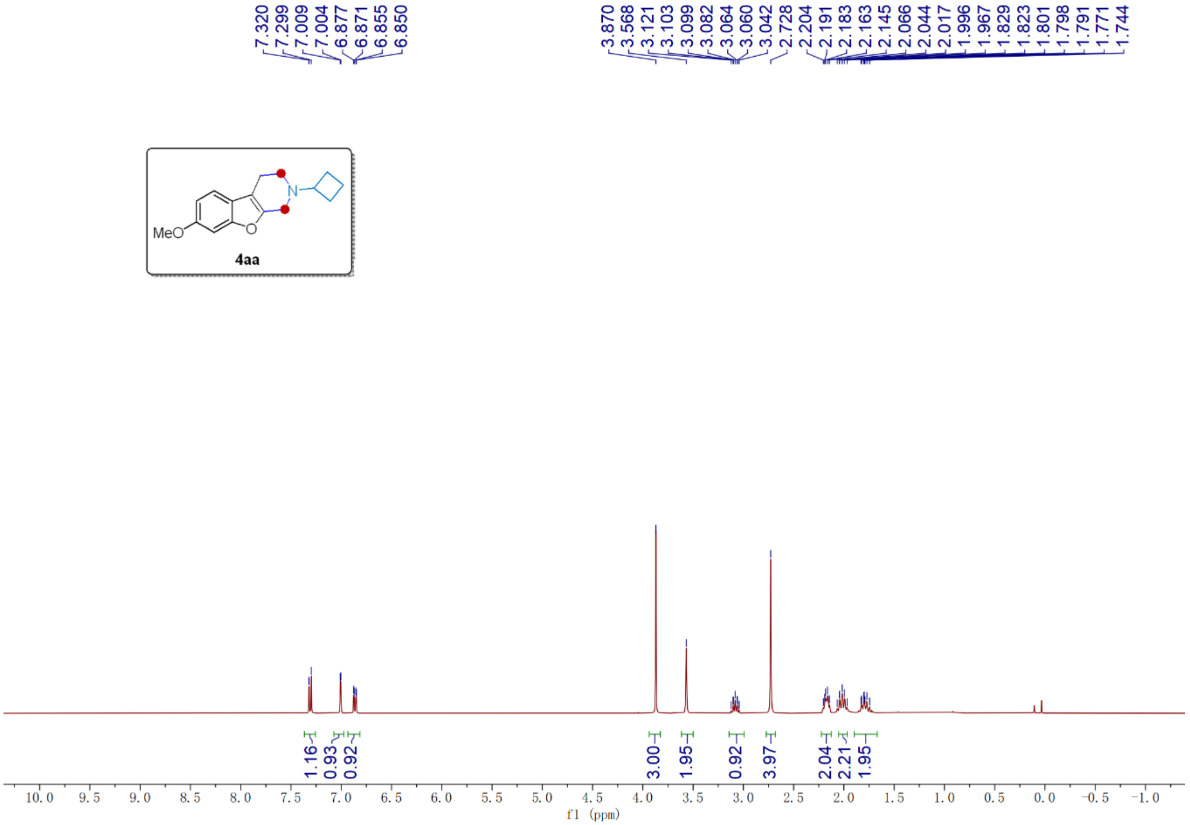

Supplementary Figure 85. ^{13}C-NMR spectrum of compound **4aa** (100 MHz, $CDCl_3$)

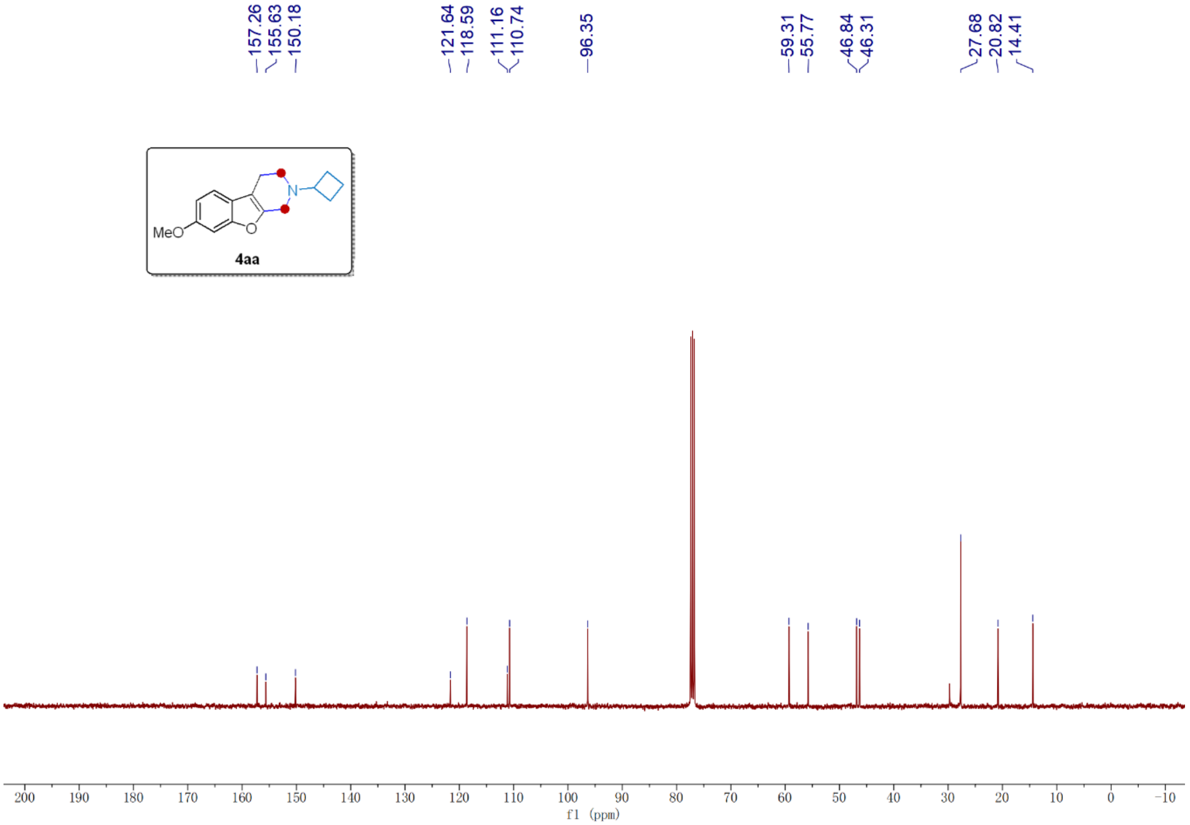

Supplementary Figure 86. ^{1}H-NMR spectrum of compound **4ab** (600 MHz, $CDCl_3$)

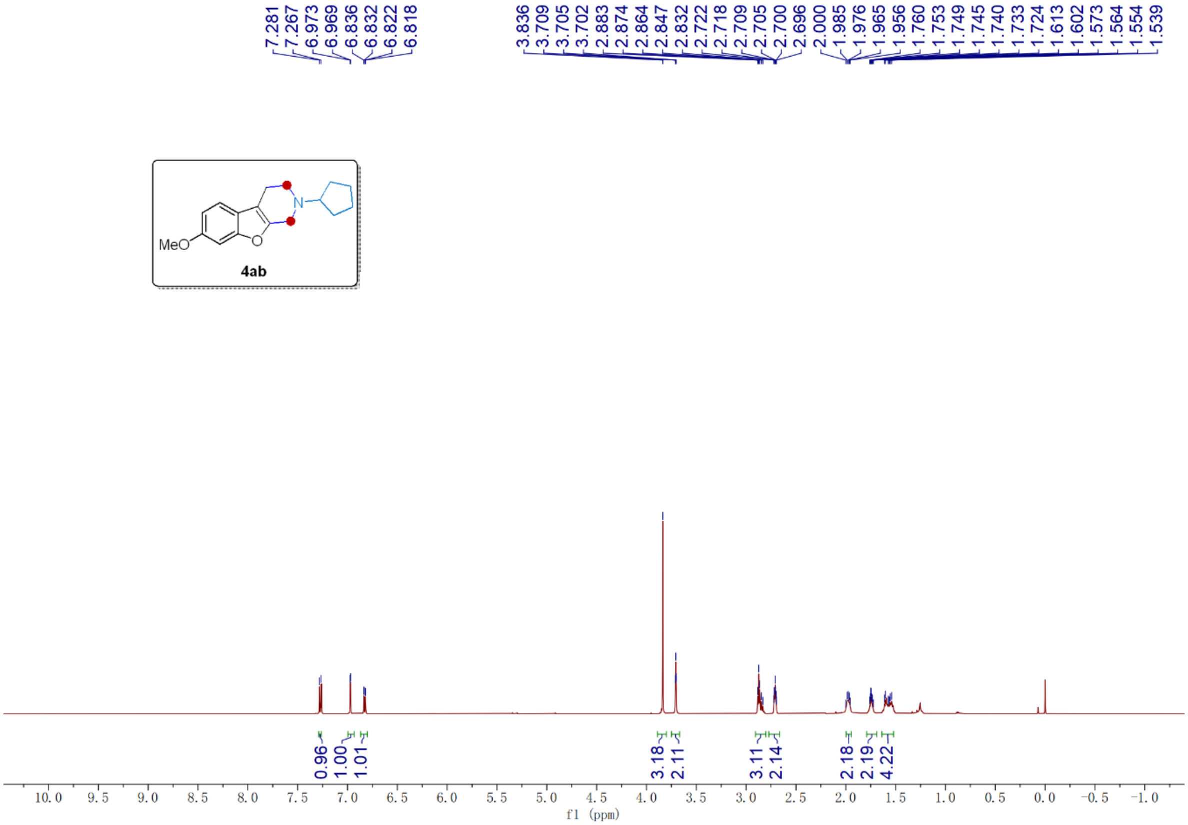

Supplementary Figure 87. ^{13}C-NMR spectrum of compound **4ab** (150 MHz, $CDCl_3$)

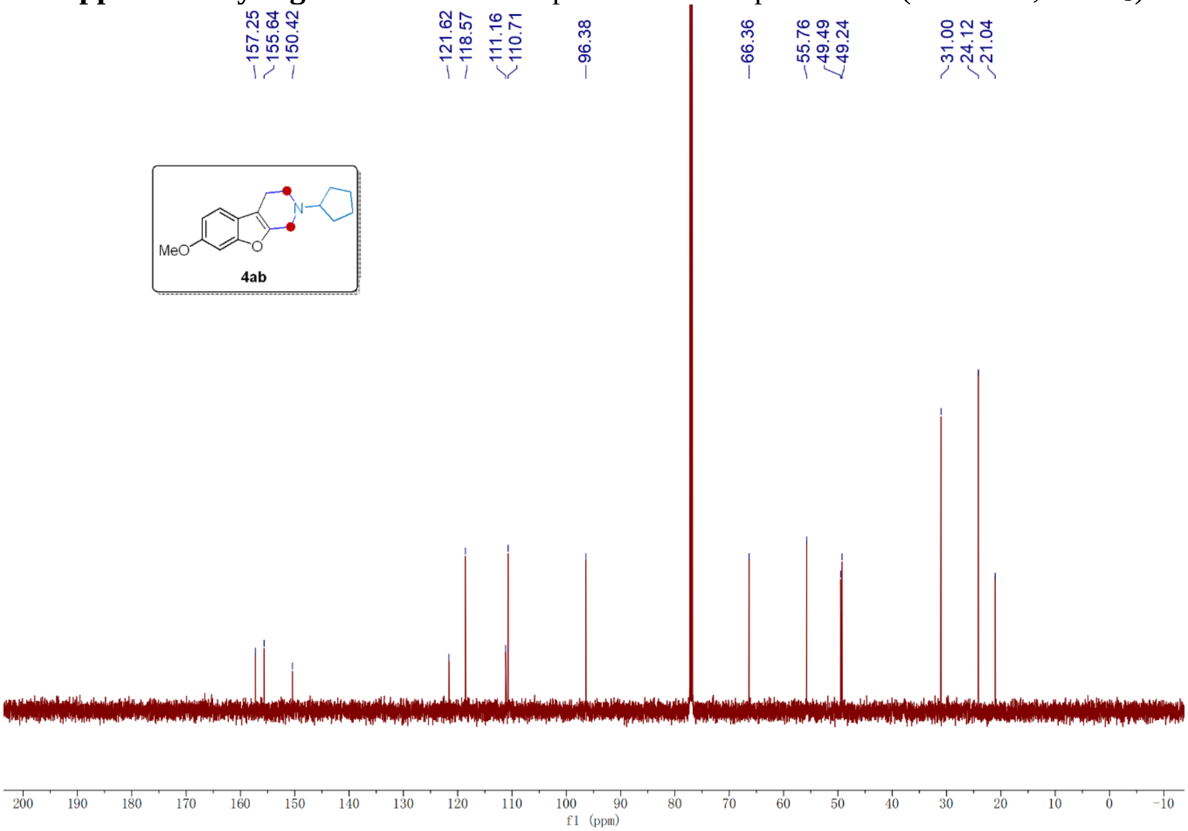

Supplementary Figure 88. ^{1}H-NMR spectrum of compound **4ac** (500 MHz, $CDCl_3$)

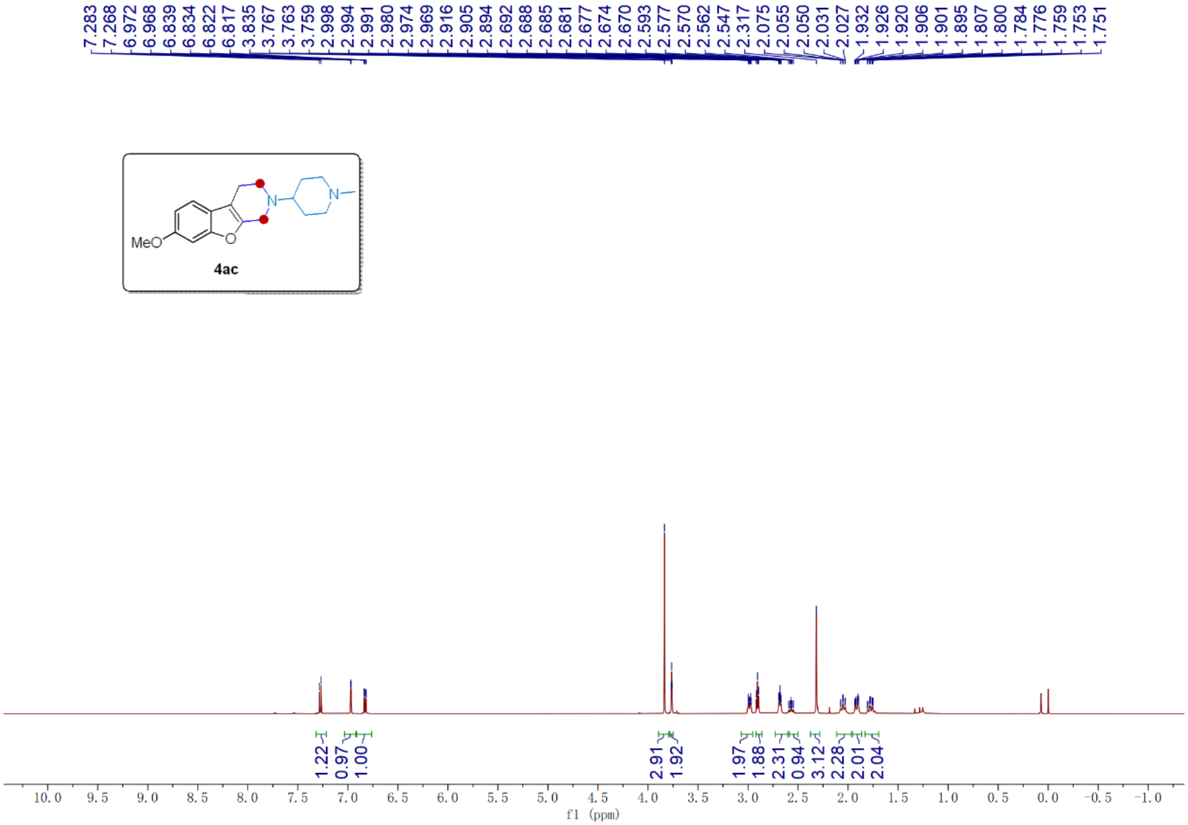

Supplementary Figure 89. ^{13}C-NMR spectrum of compound **4ac** (125 MHz, $CDCl_3$)

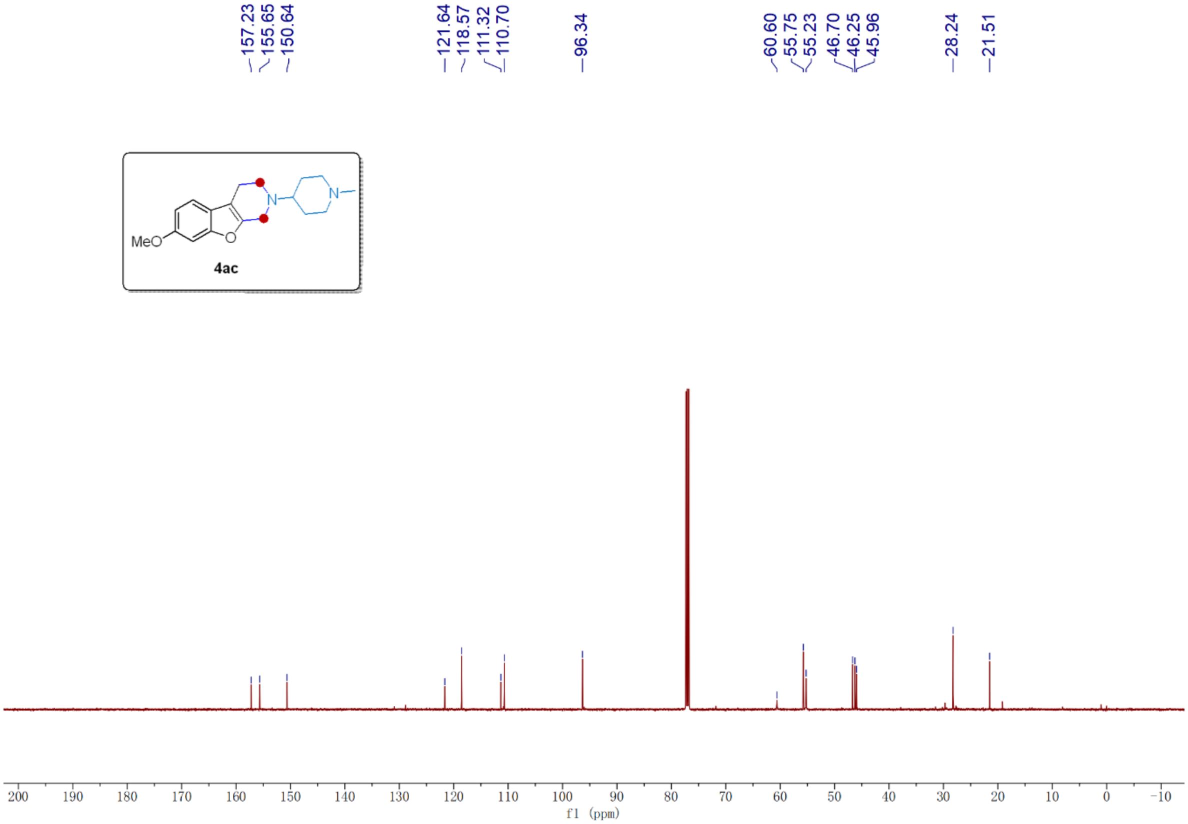

Supplementary Figure 90. ^{1}H-NMR spectrum of compound **4ad** (400 MHz, $CDCl_3$)

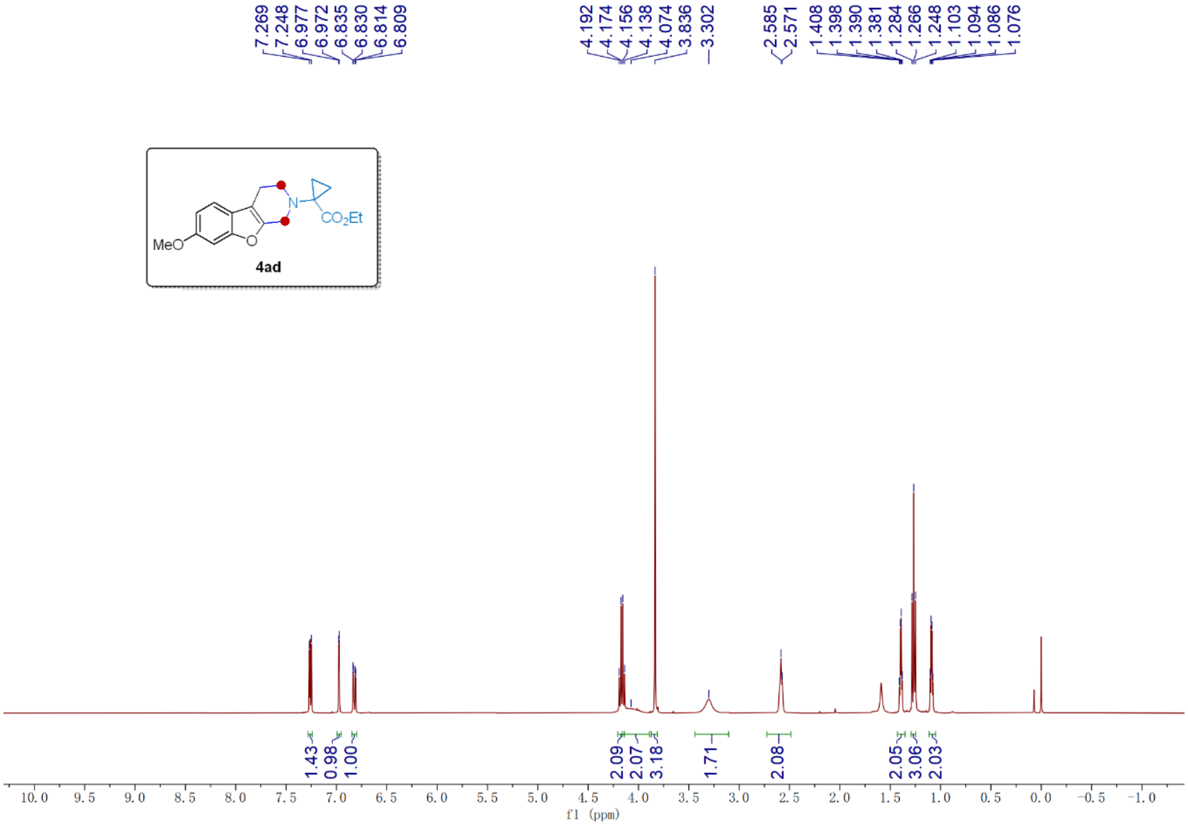

Supplementary Figure 91. ^{13}C-NMR spectrum of compound **4ad** (100 MHz, $CDCl_3$)

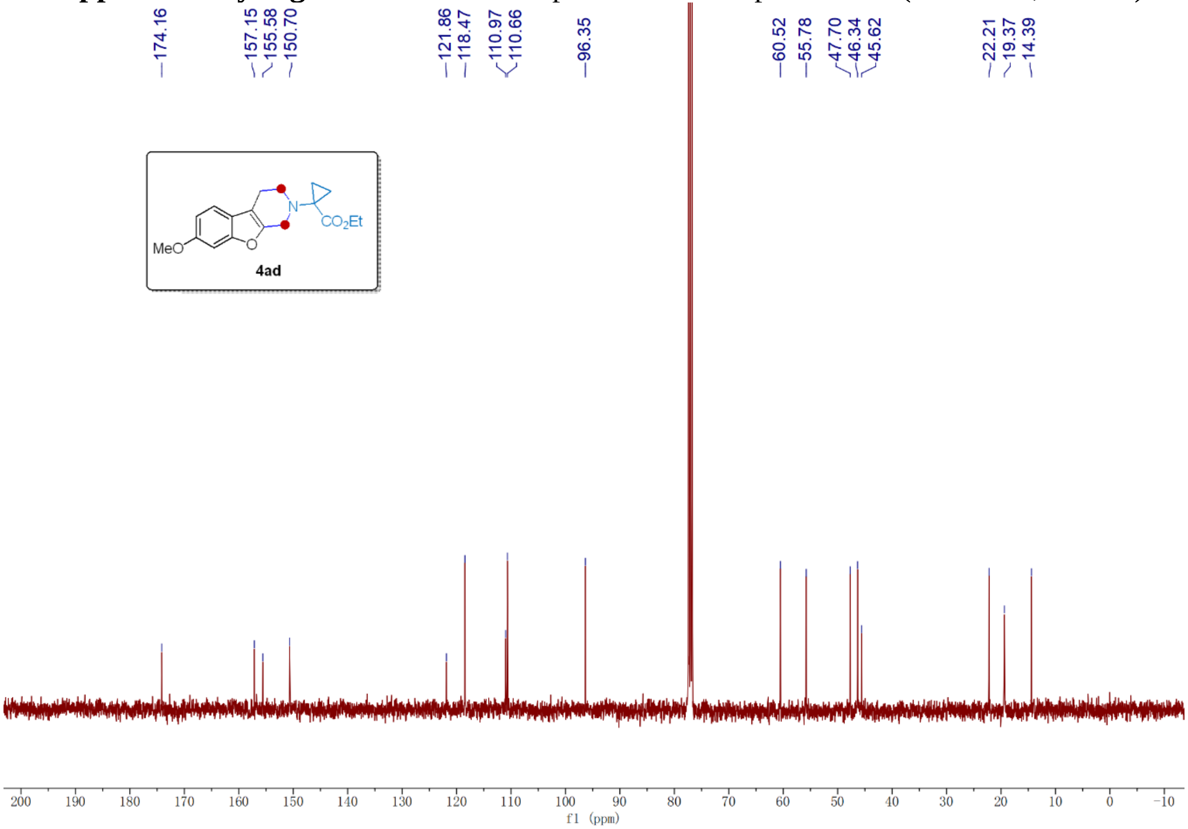

Supplementary Figure 92. ^{1}H-NMR spectrum of compound **4ae** (500 MHz, $CDCl_3$)

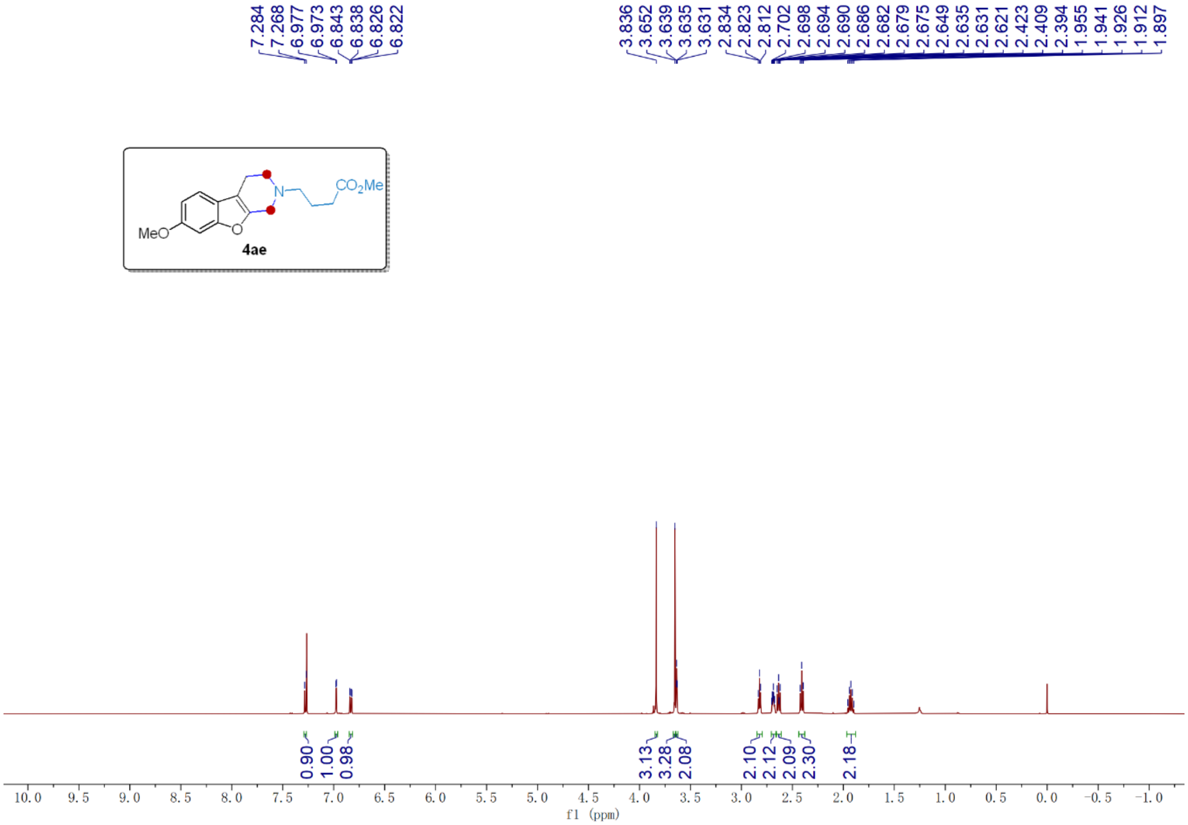

Supplementary Figure 93. ^{13}C-NMR spectrum of compound **4ae** (125 MHz, $CDCl_3$)

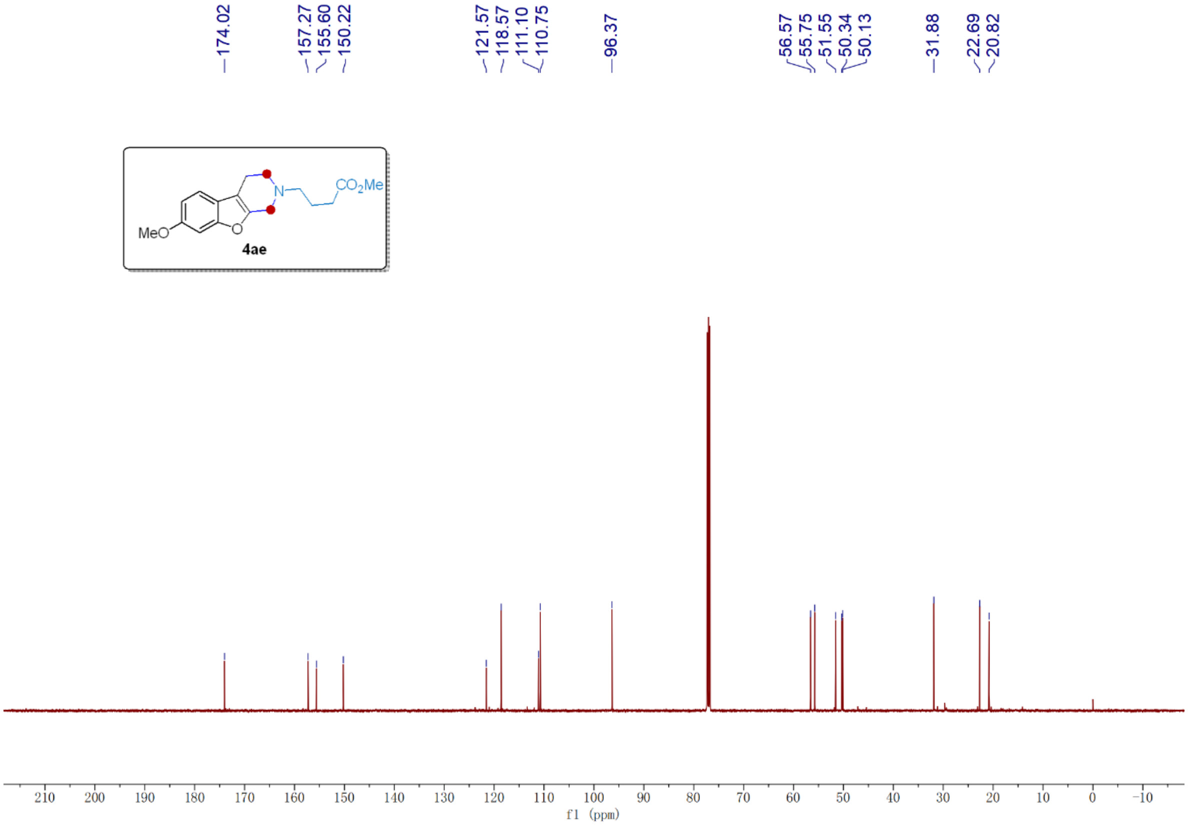

Supplementary Figure 94. ^{1}H-NMR spectrum of compound **4af** (400 MHz, $CDCl_3$)

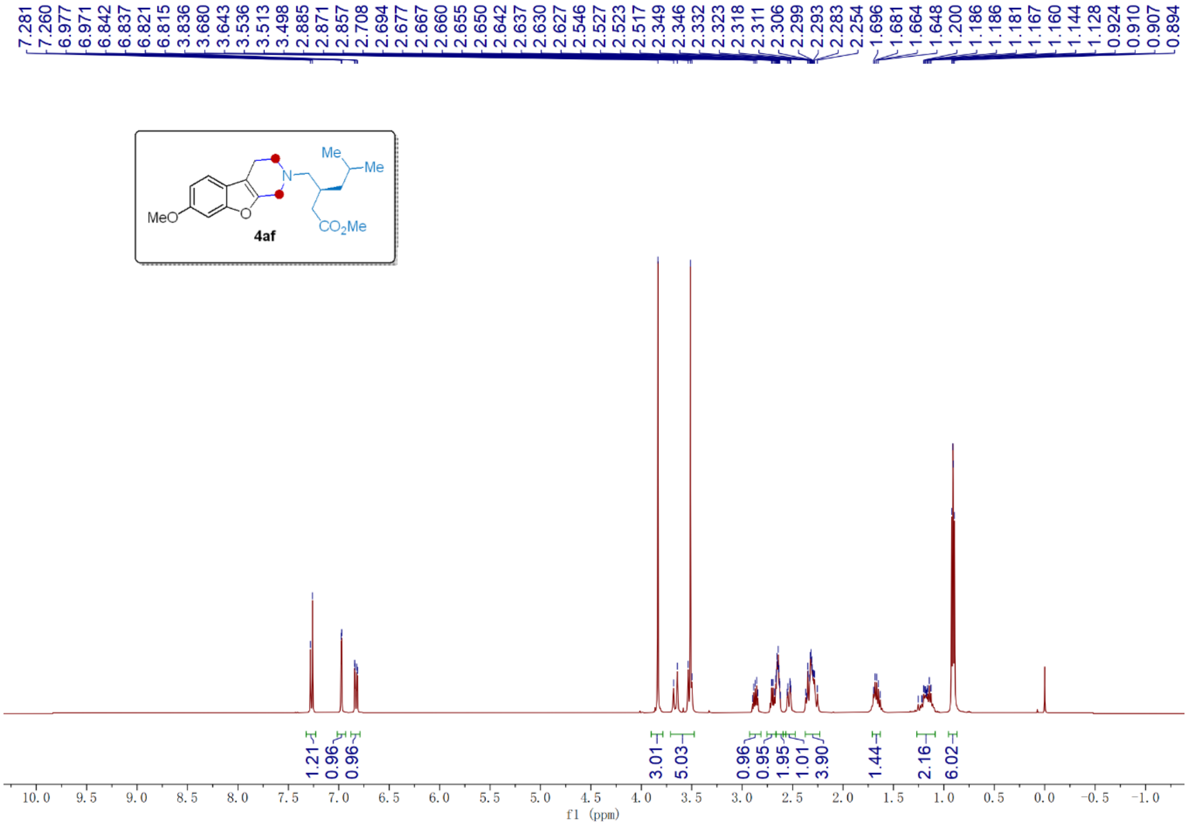

Supplementary Figure 95. ^{13}C-NMR spectrum of compound **4af** (100 MHz, $CDCl_3$)

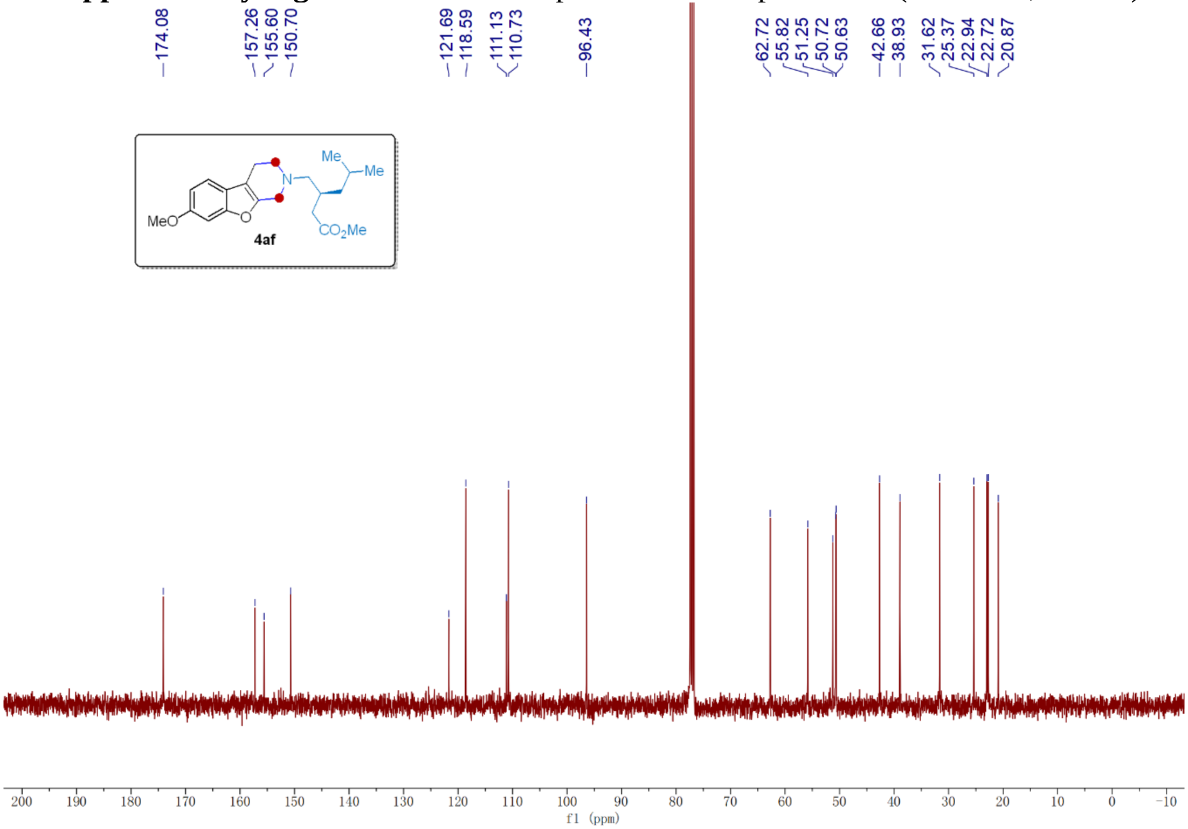

Supplementary Figure 96. ^{1}H-NMR spectrum of compound **4ag** (400 MHz, $CDCl_3$)

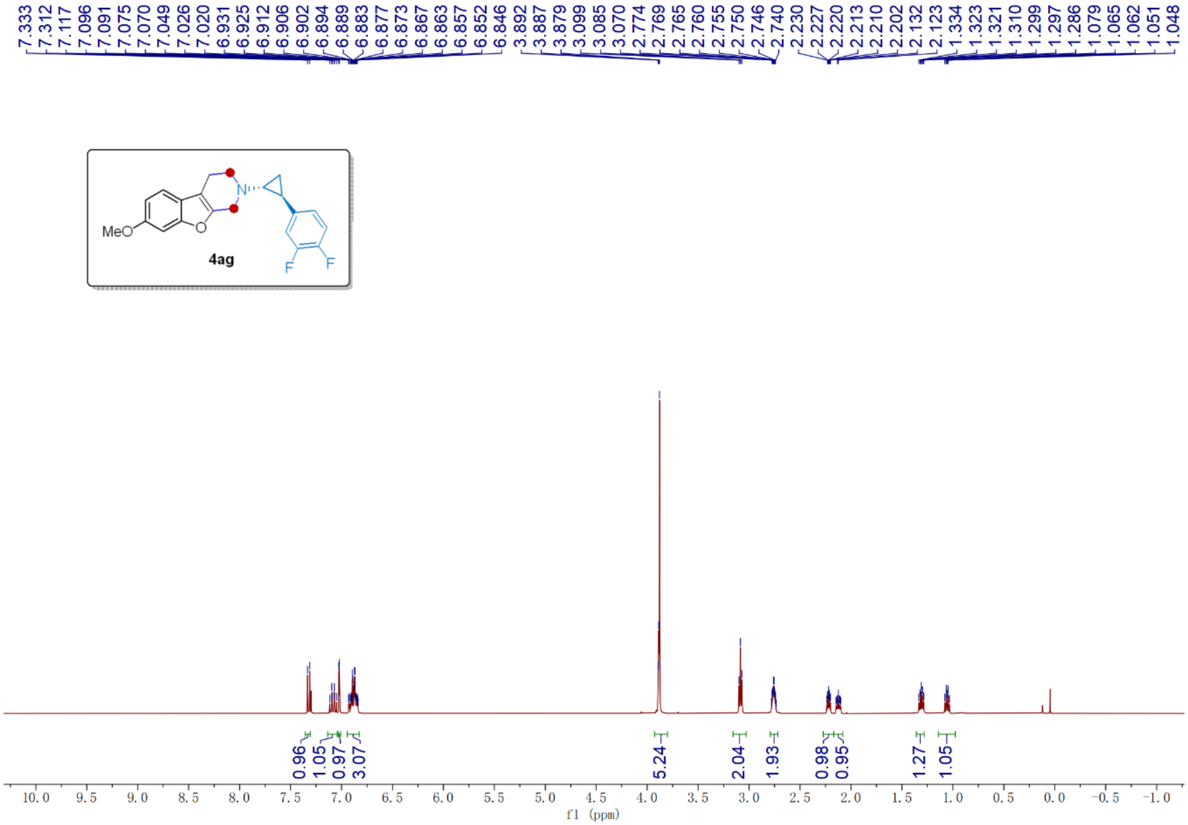

Supplementary Figure 97. ^{13}C-NMR spectrum of compound **4ag** (100 MHz, $CDCl_3$)

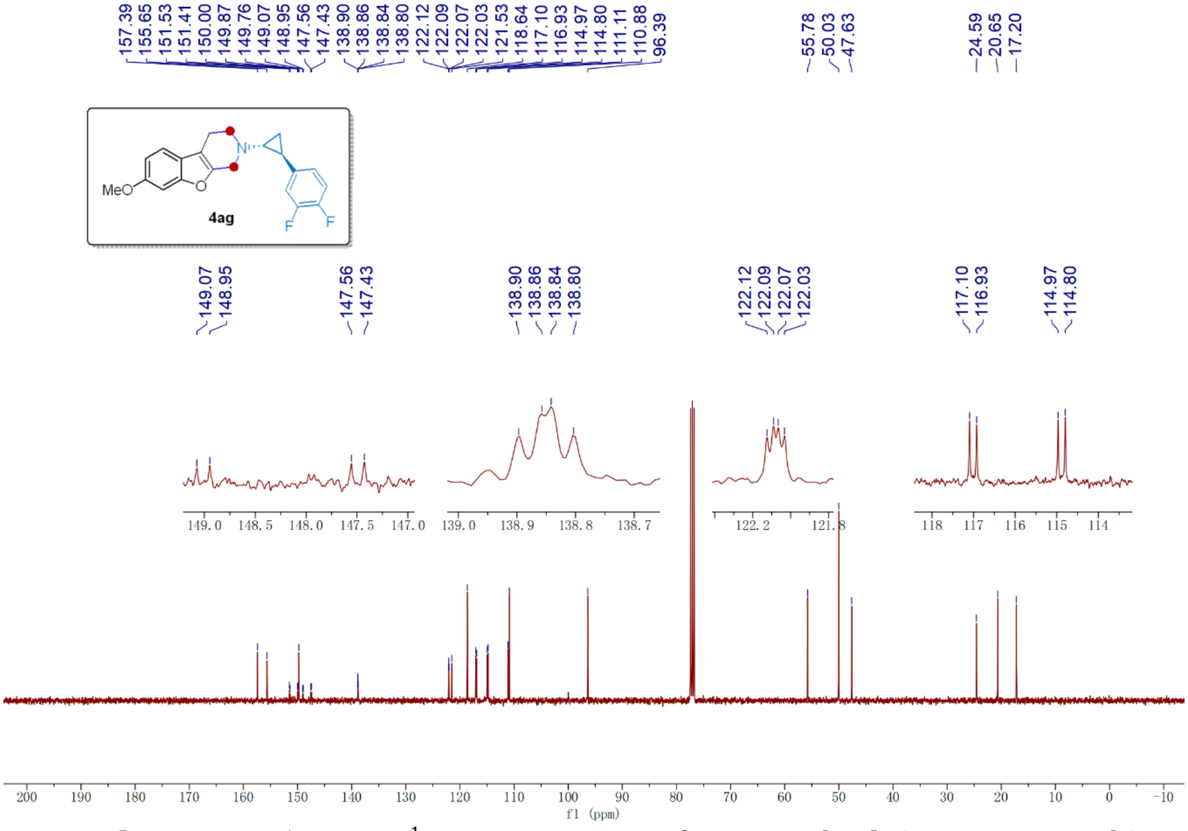

Supplementary Figure 98. ^{1}H-NMR spectrum of compound **4ah** (500 MHz, $CDCl_3$)

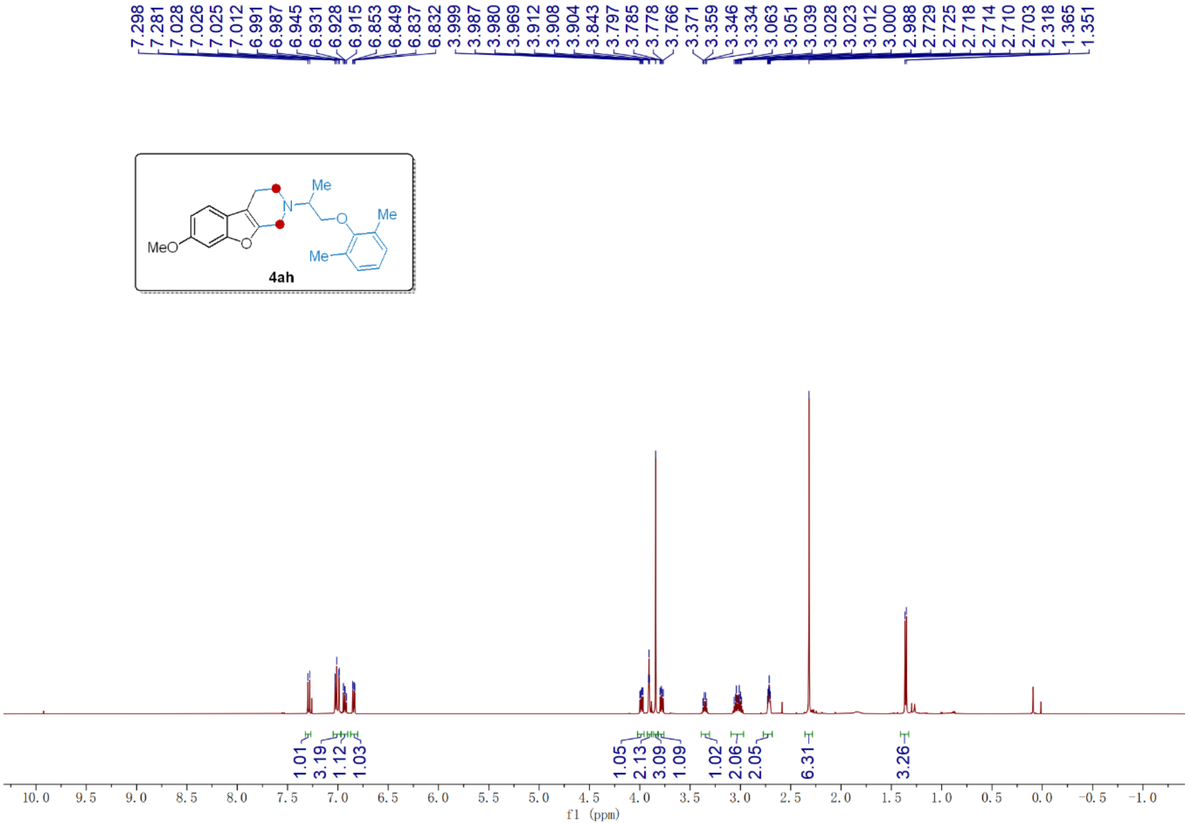

Supplementary Figure 99. ^{13}C-NMR spectrum of compound **4ah** (150 MHz, $CDCl_3$)

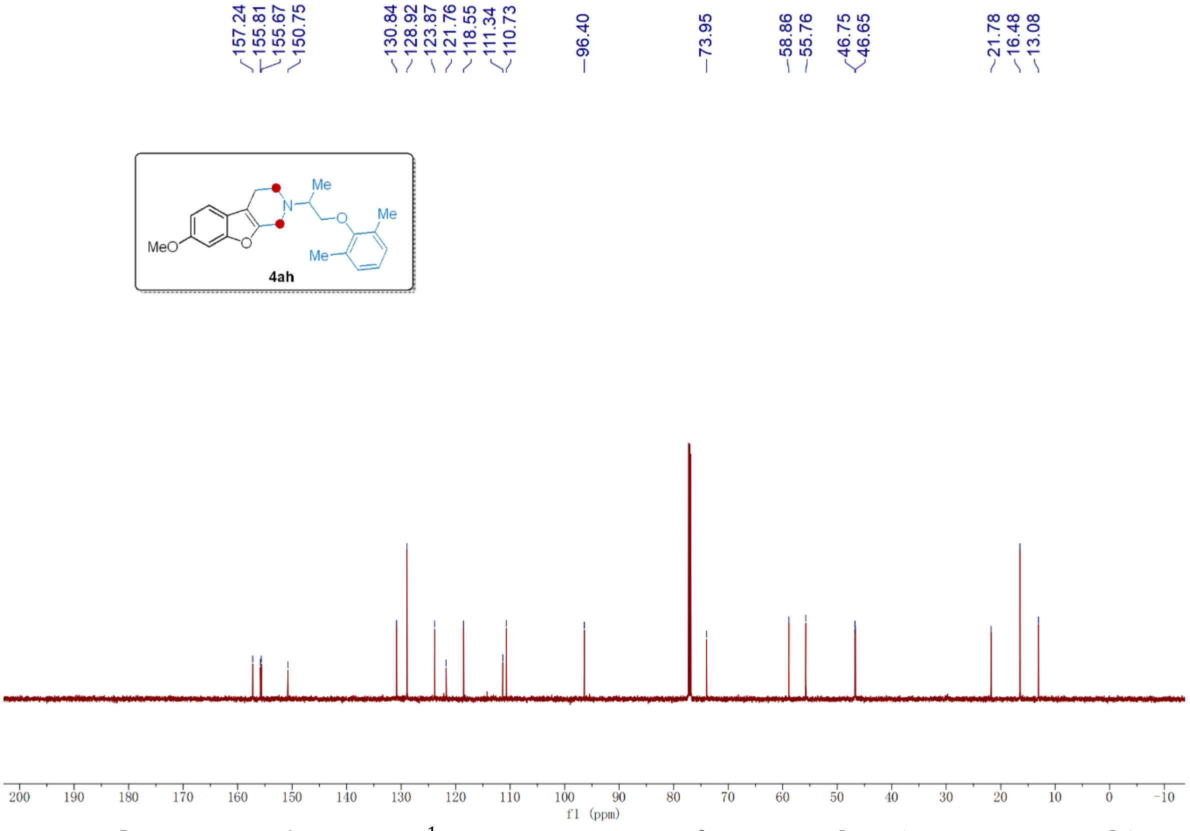

Supplementary Figure 100. ^{1}H-NMR spectrum of compound **5a** (600 MHz, $CDCl_3$)

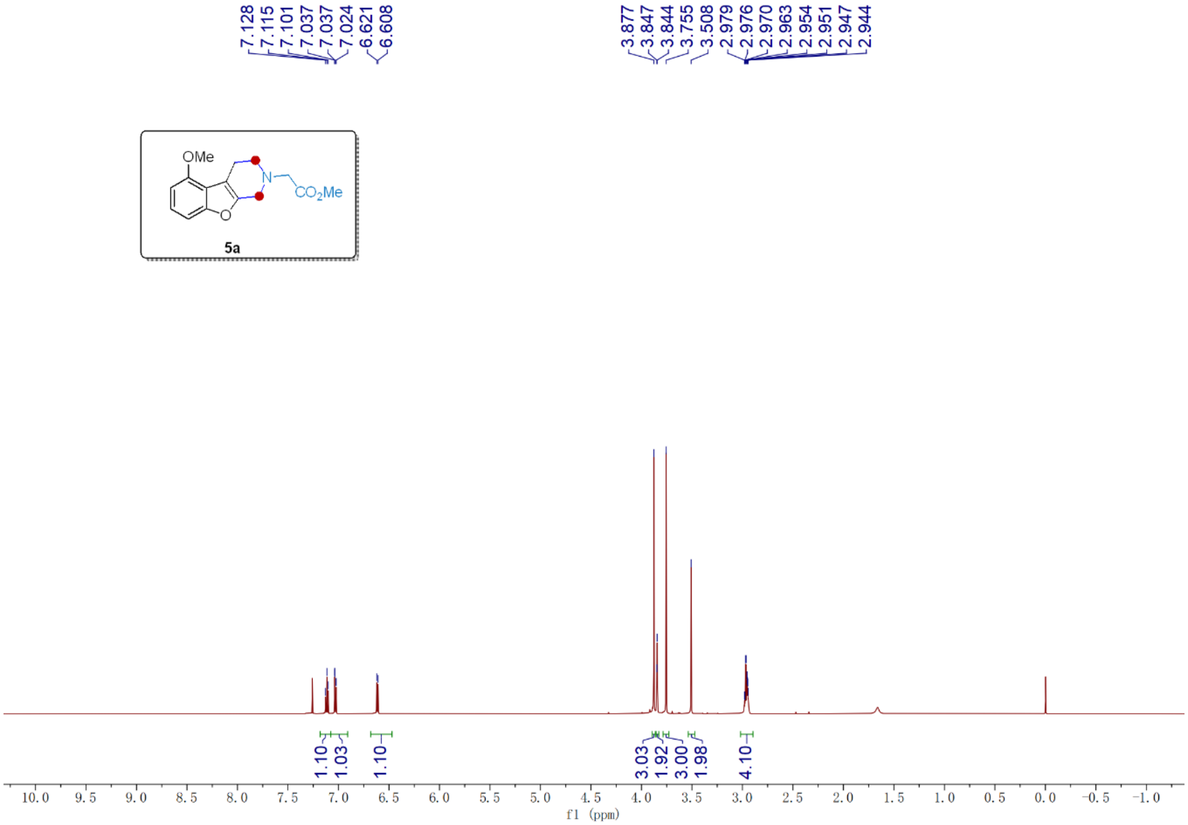

Supplementary Figure 101. ^{13}C-NMR spectrum of compound **5a** (125 MHz, $CDCl_3$)

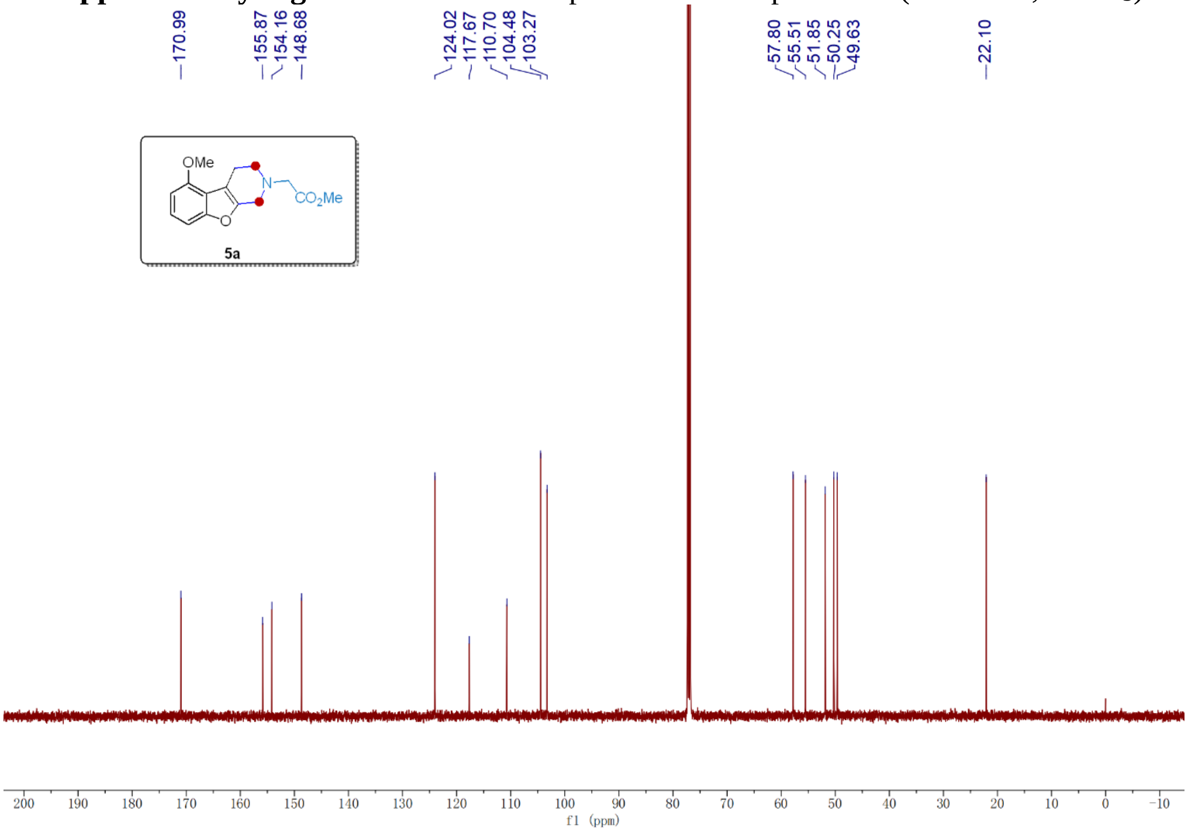

Supplementary Figure 102. ^{1}H-NMR spectrum of compound **5b** (600 MHz, $CDCl_3$)

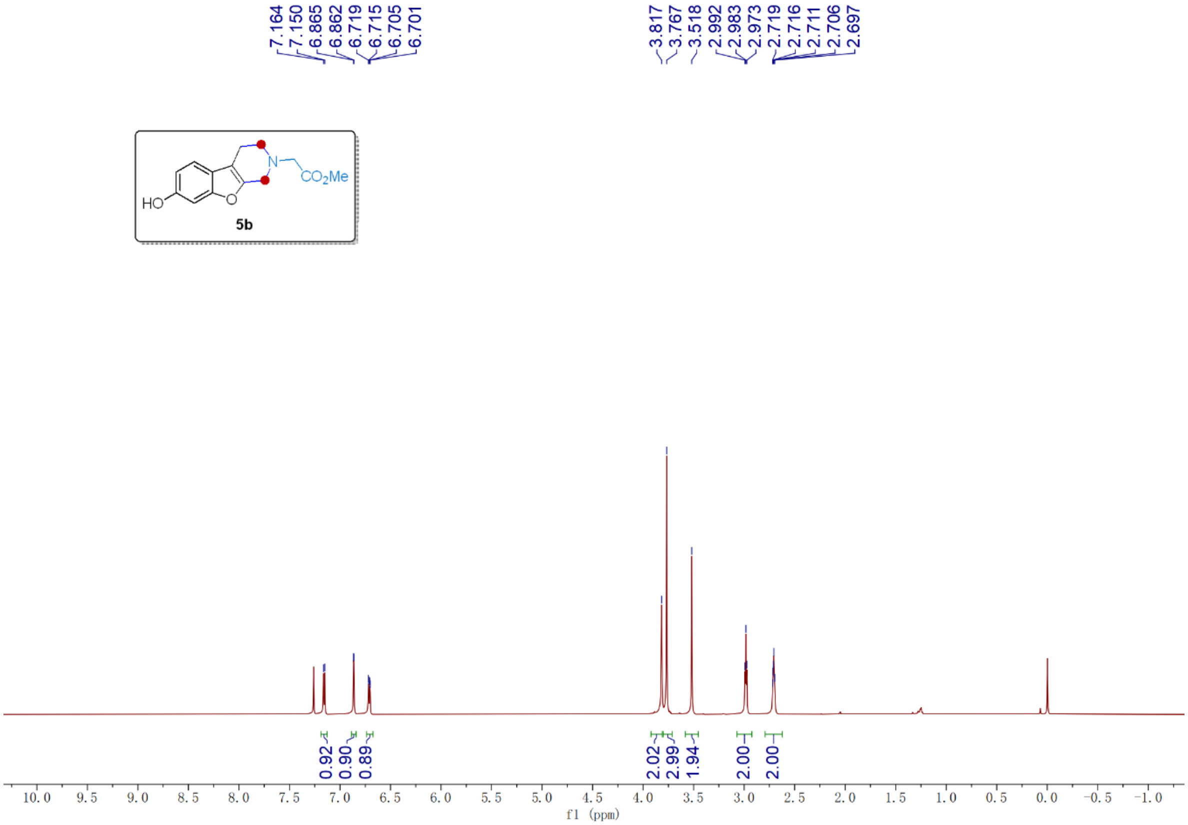

Supplementary Figure 103. ^{13}C-NMR spectrum of compound **5b** (150 MHz, $CDCl_3$)

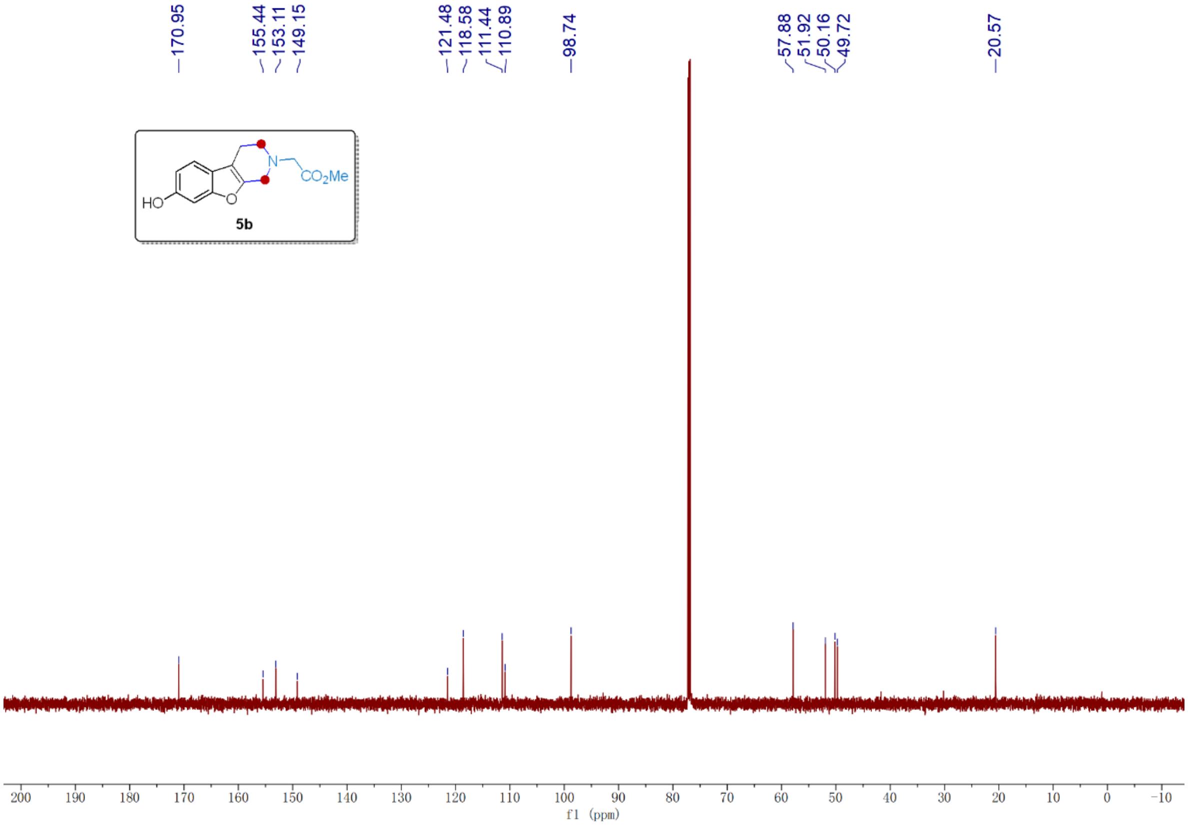

Supplementary Figure 104. ^{1}H-NMR spectrum of compound **5c** (600 MHz, $CDCl_3$)

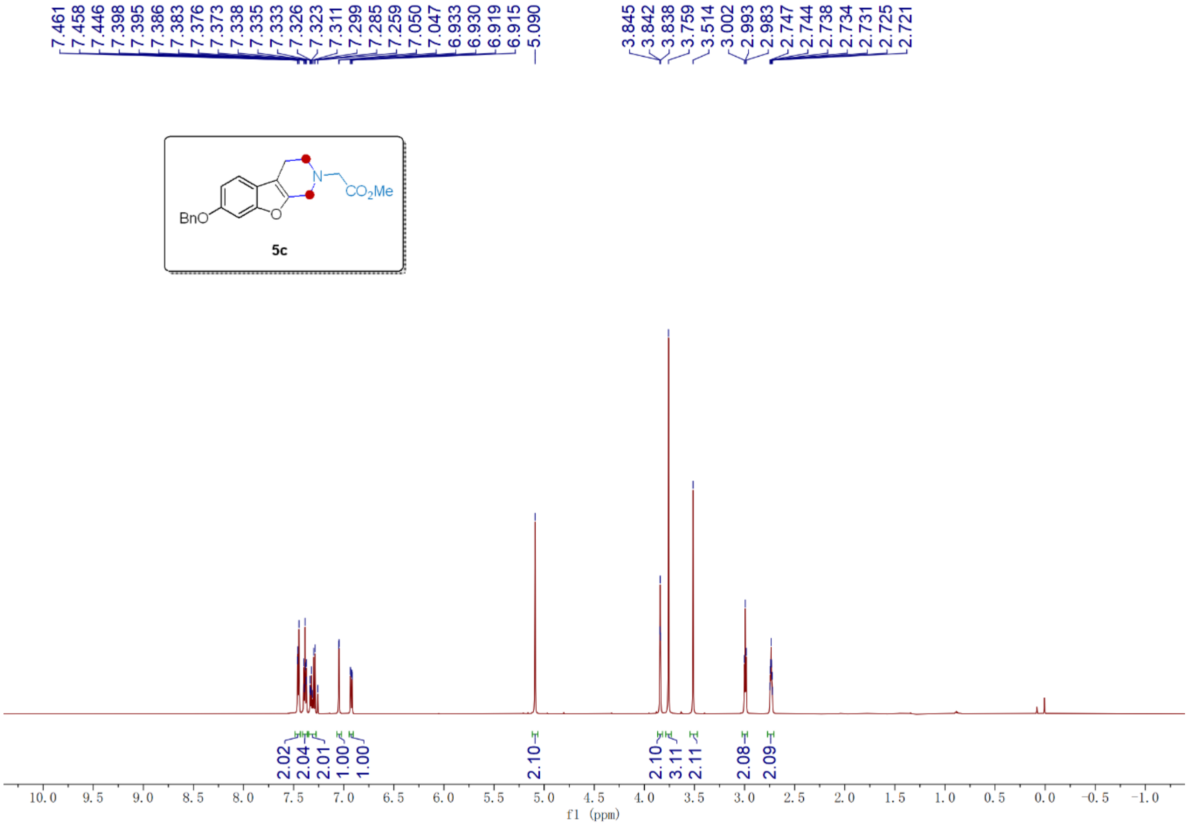

Supplementary Figure 105. ^{13}C-NMR spectrum of compound **5c** (150 MHz, $CDCl_3$)

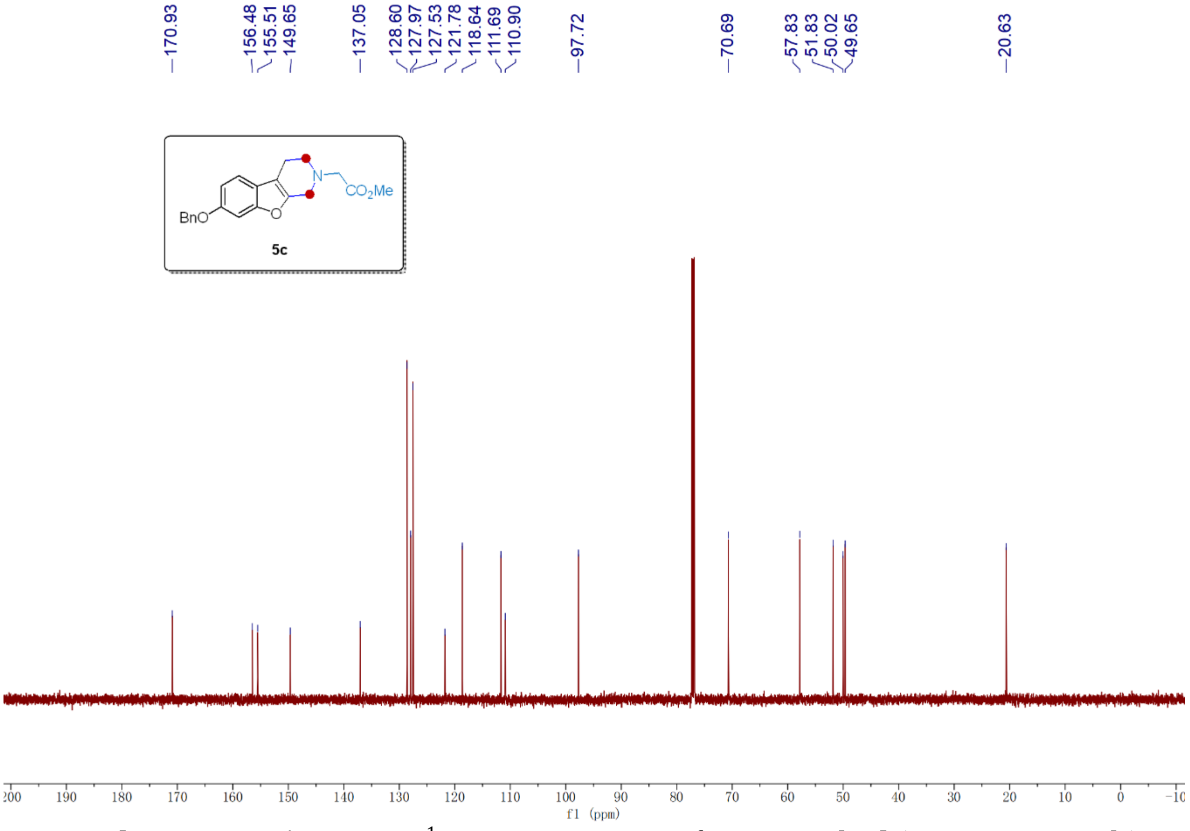

Supplementary Figure 106. ^{1}H-NMR spectrum of compound **5d** (500 MHz, $CDCl_3$)

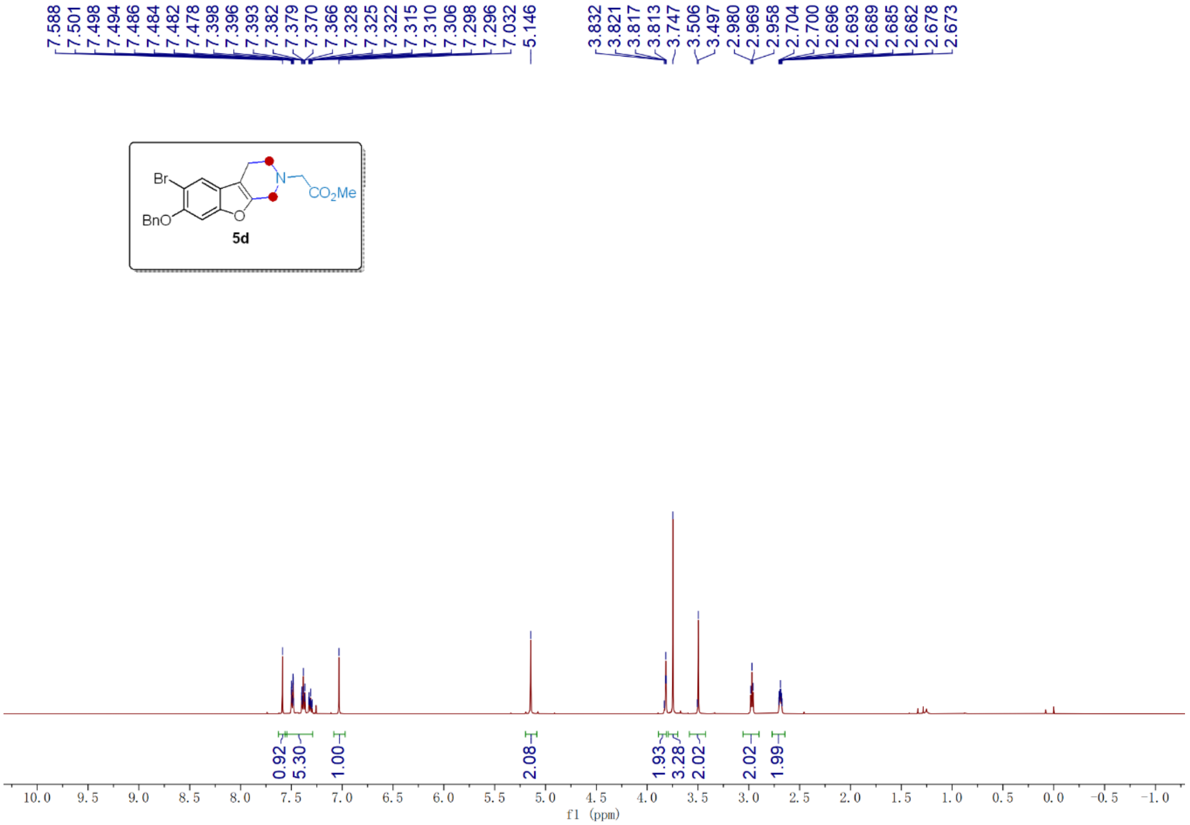

Supplementary Figure 107. ^{13}C-NMR spectrum of compound **5d** (125 MHz, $CDCl_3$)

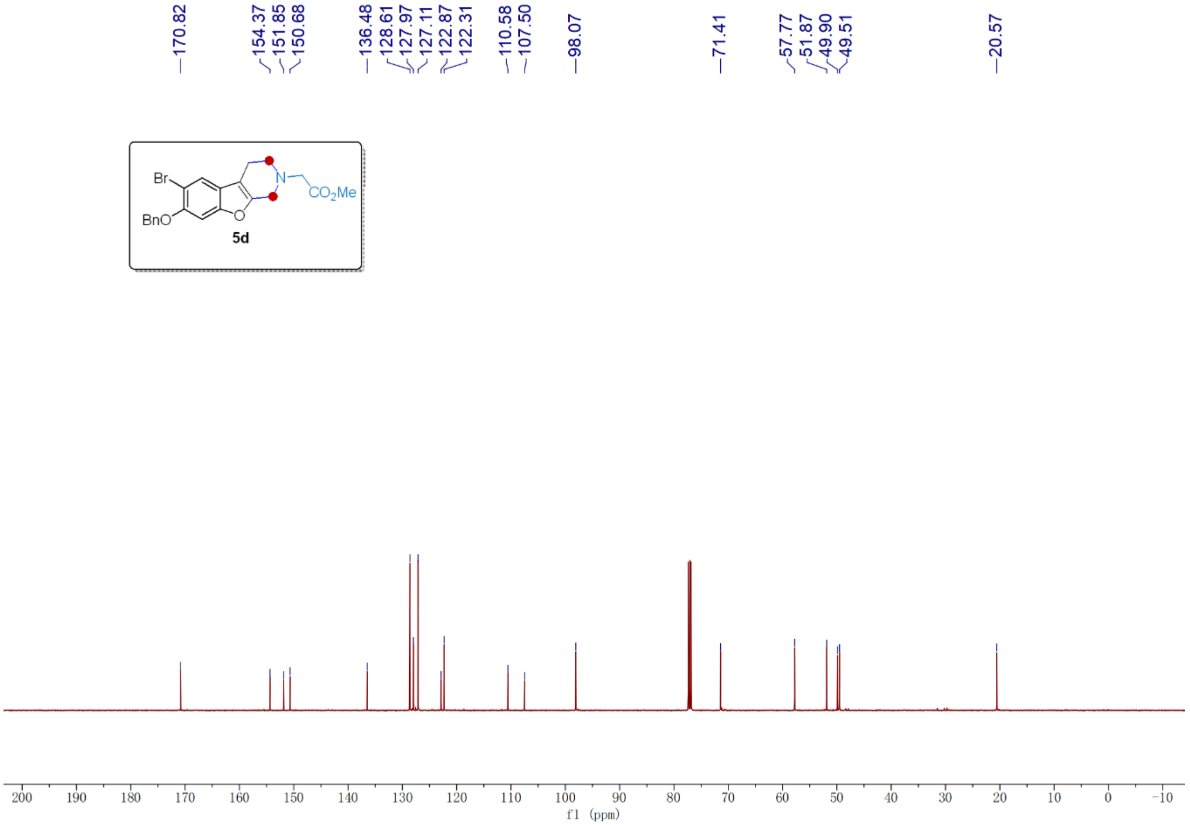

Supplementary Figure 108. ^{1}H-NMR spectrum of compound **5e** (600 MHz, $CDCl_3$)

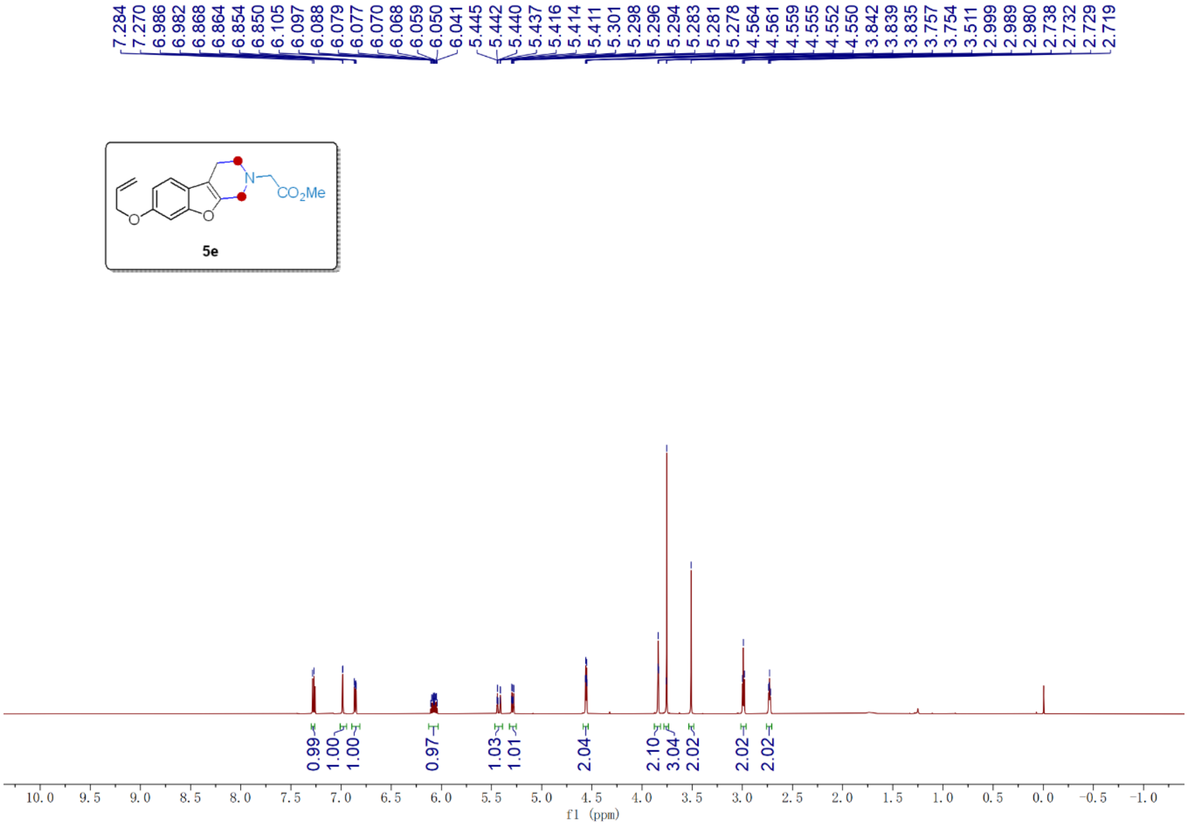

Supplementary Figure 109. ^{13}C-NMR spectrum of compound **5e** (125 MHz, $CDCl_3$)

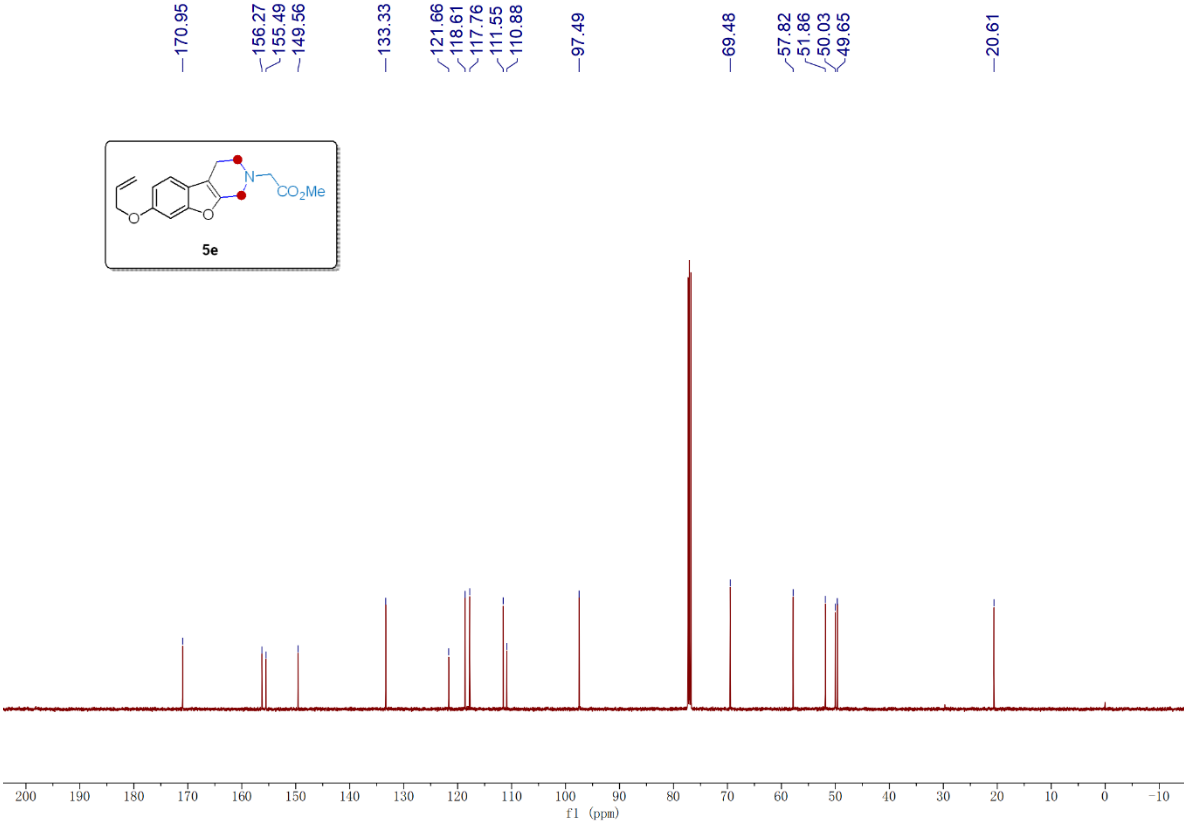

Supplementary Figure 110. ^{1}H-NMR spectrum of compound **5f** (500 MHz, $CDCl_3$)

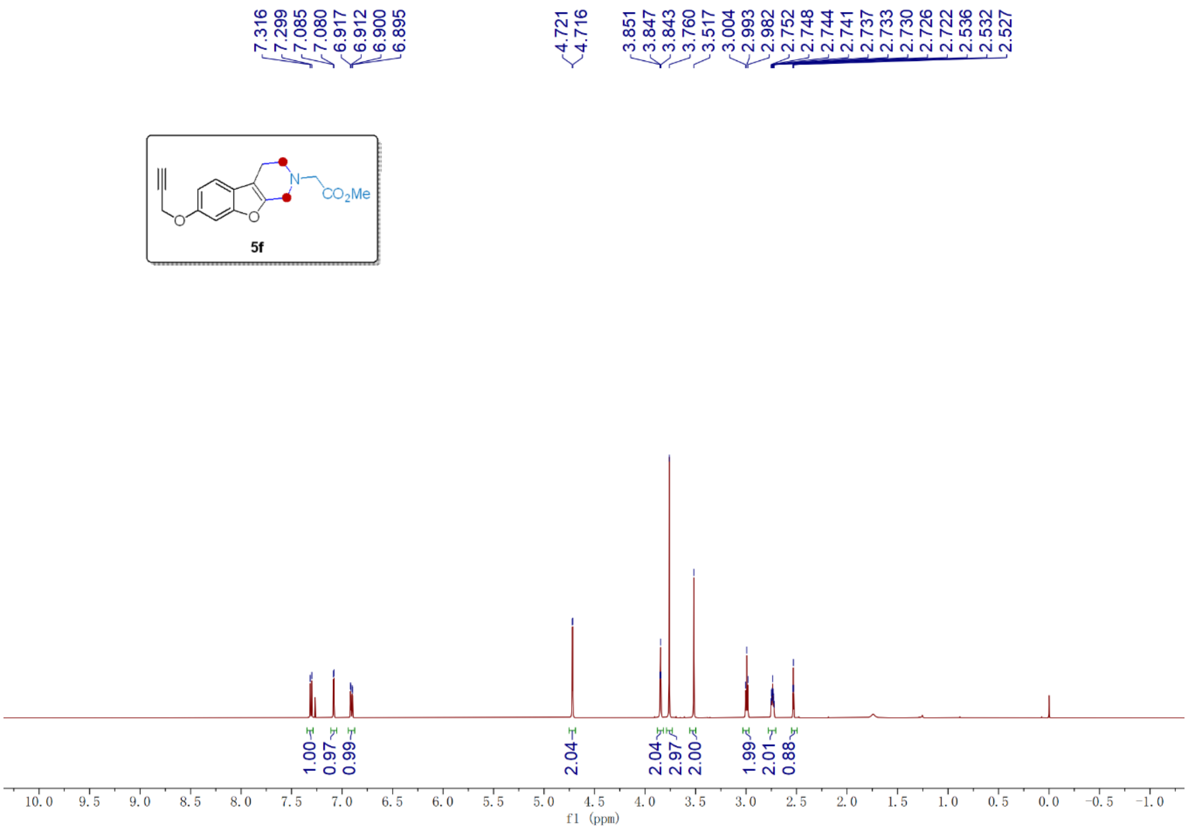

Supplementary Figure 111. ^{13}C-NMR spectrum of compound **5f** (125 MHz, $CDCl_3$)

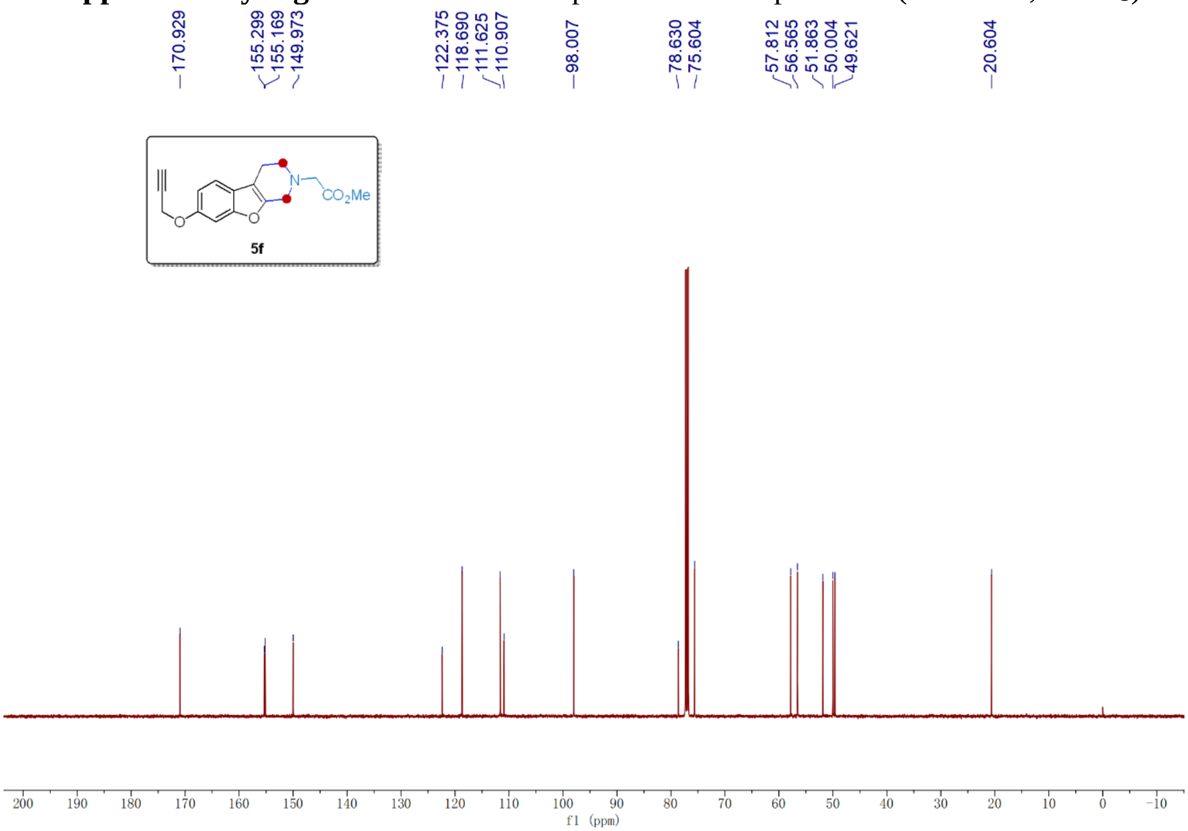

Supplementary Figure 112. ^{1}H-NMR spectrum of compound **5g** (400 MHz, $CDCl_3$)

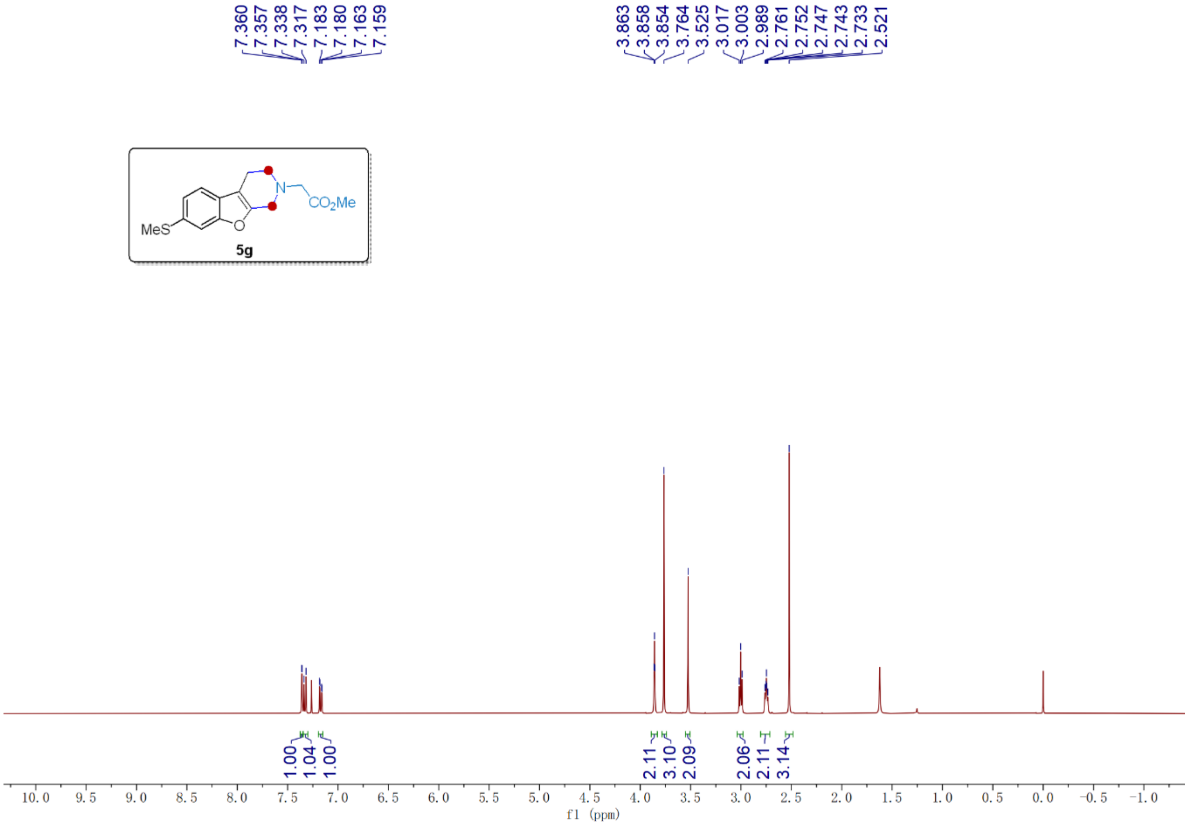

Supplementary Figure 113. ^{13}C-NMR spectrum of compound **5g** (100 MHz, $CDCl_3$)

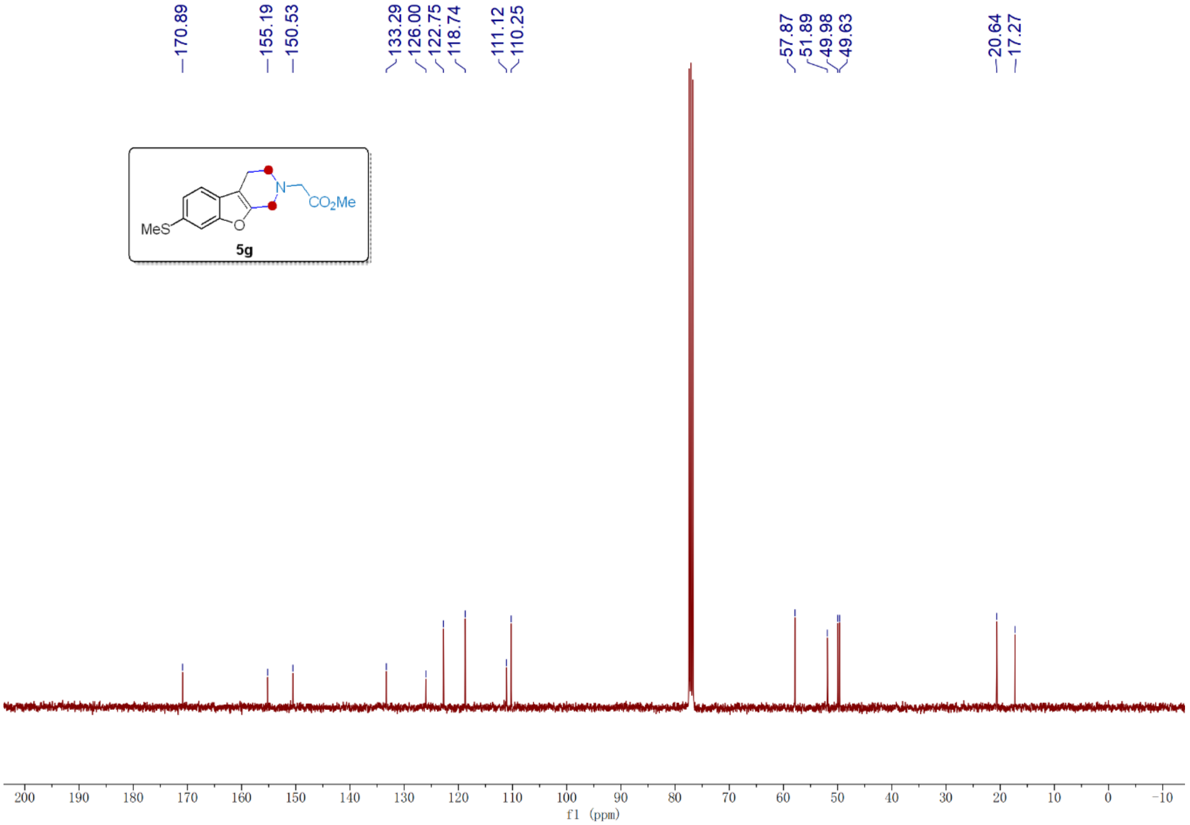

Supplementary Figure 114. ^{1}H-NMR spectrum of compound **5h** (600 MHz, $CDCl_3$)

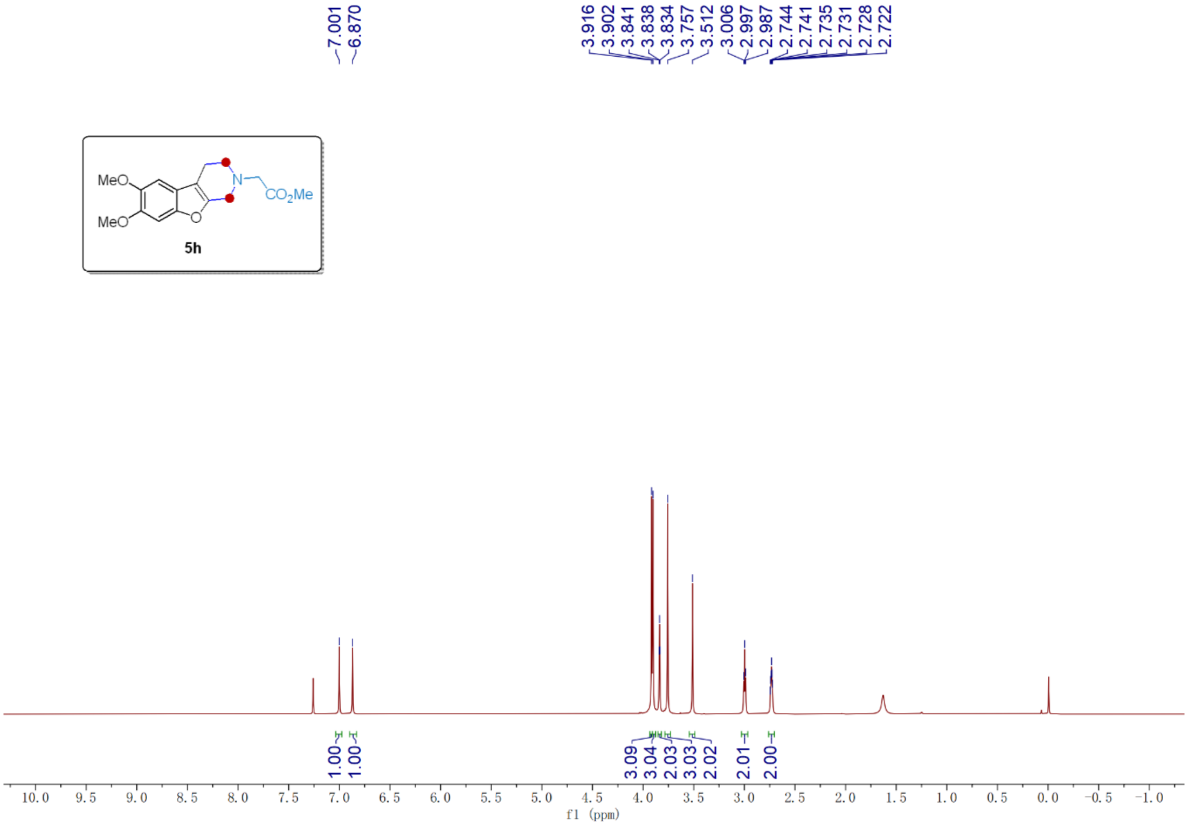

Supplementary Figure 115. ^{13}C-NMR spectrum of compound **5h** (100 MHz, $CDCl_3$)

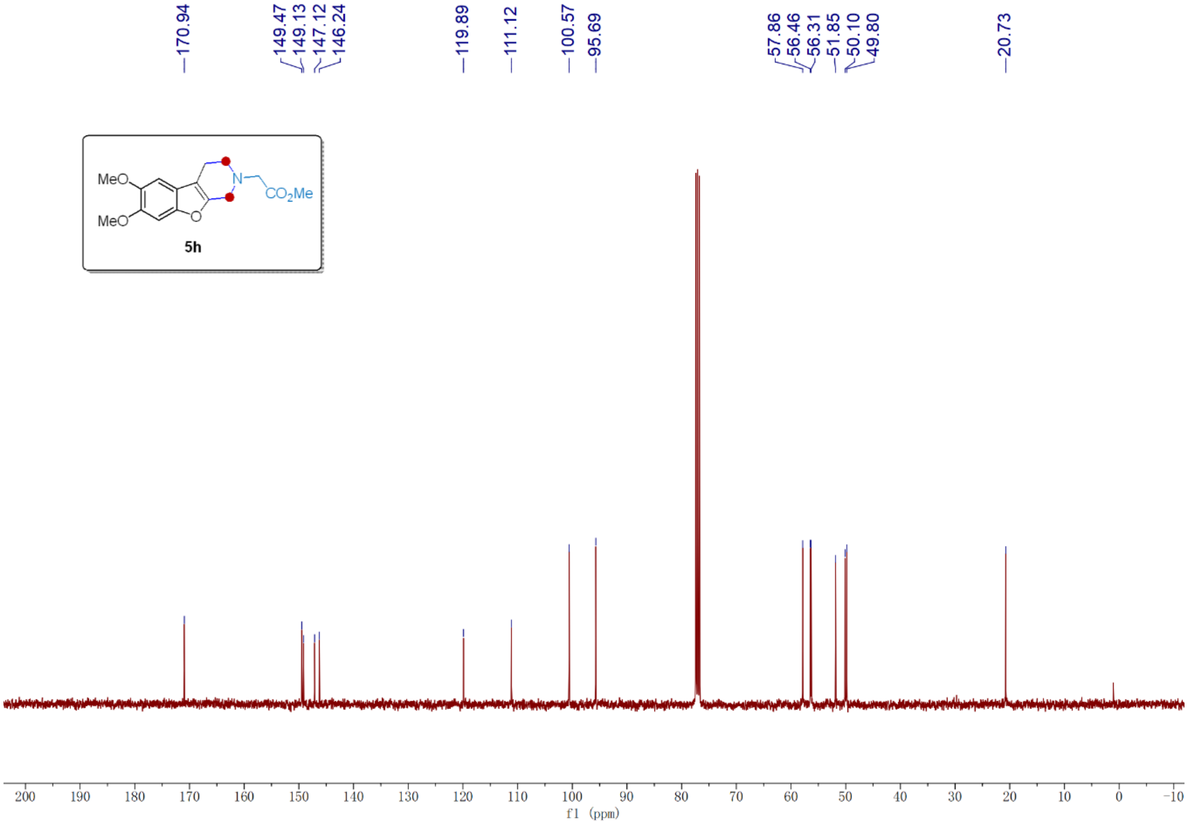

Supplementary Figure 116. ^{1}H-NMR spectrum of compound **5i** (400 MHz, $CDCl_3$)

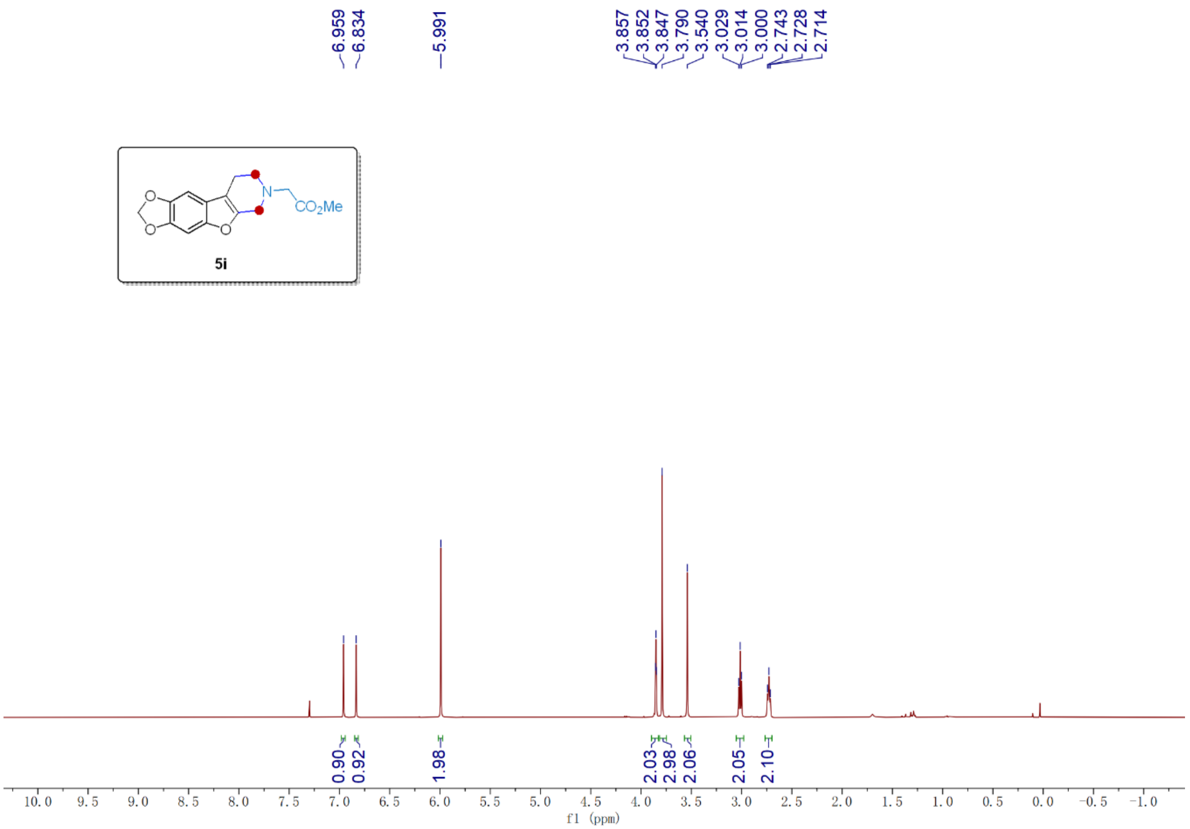

Supplementary Figure 117. ^{13}C-NMR spectrum of compound **5i** (100 MHz, $CDCl_3$)

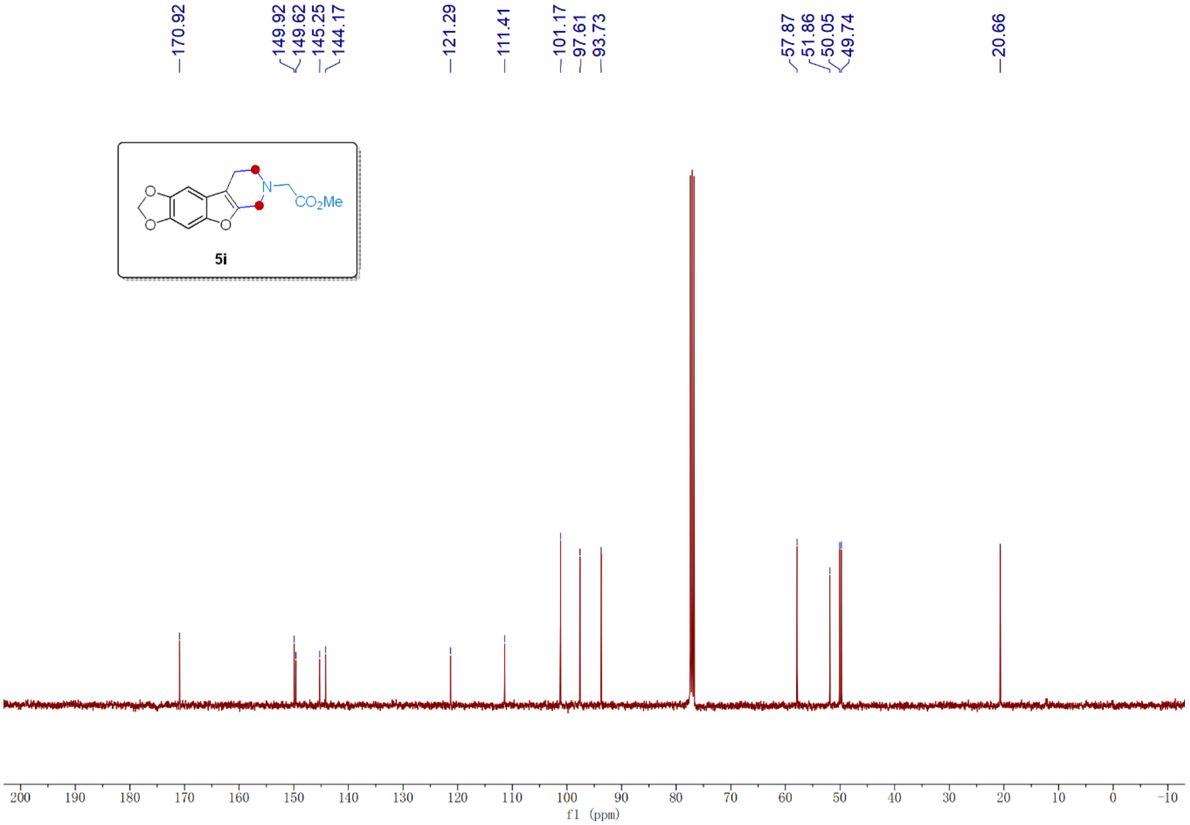

Supplementary Figure 118. ^{1}H-NMR spectrum of compound **5j** (400 MHz, $CDCl_3$)

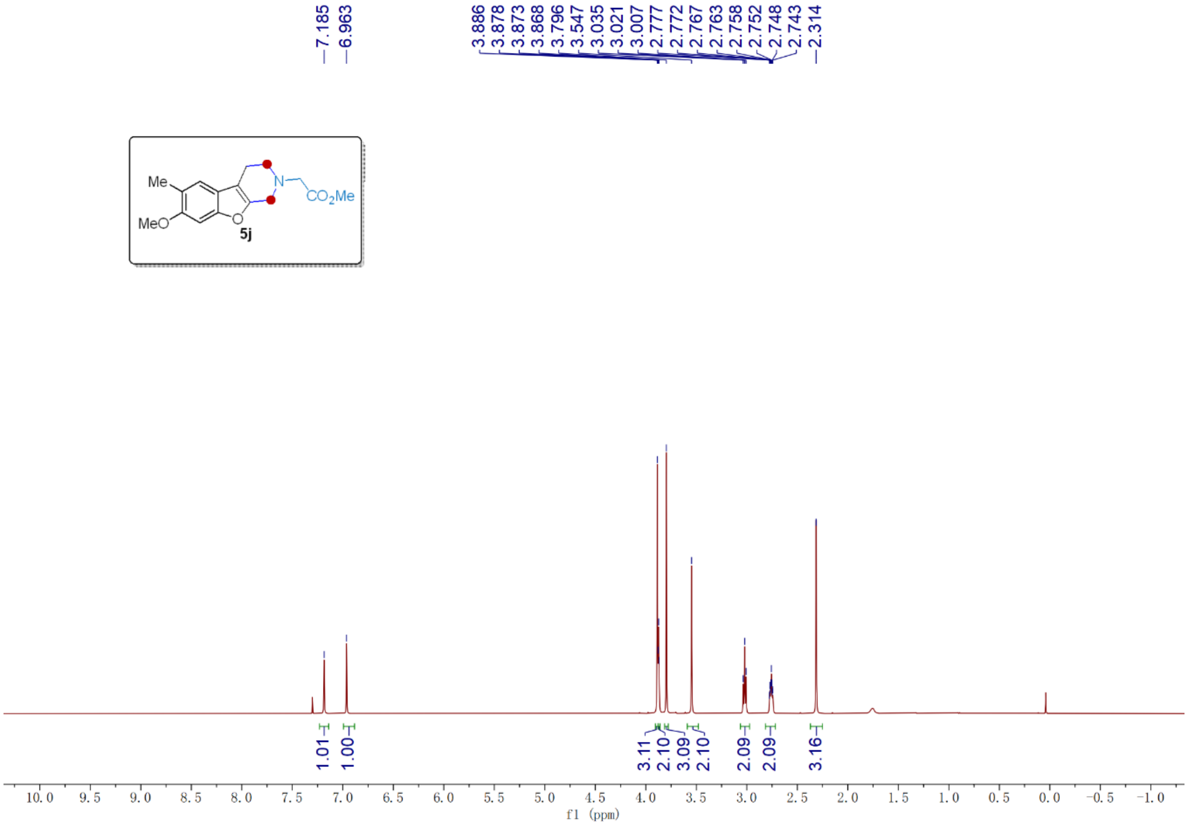

Supplementary Figure 119. ^{13}C-NMR spectrum of compound **5j** (100 MHz, $CDCl_3$)

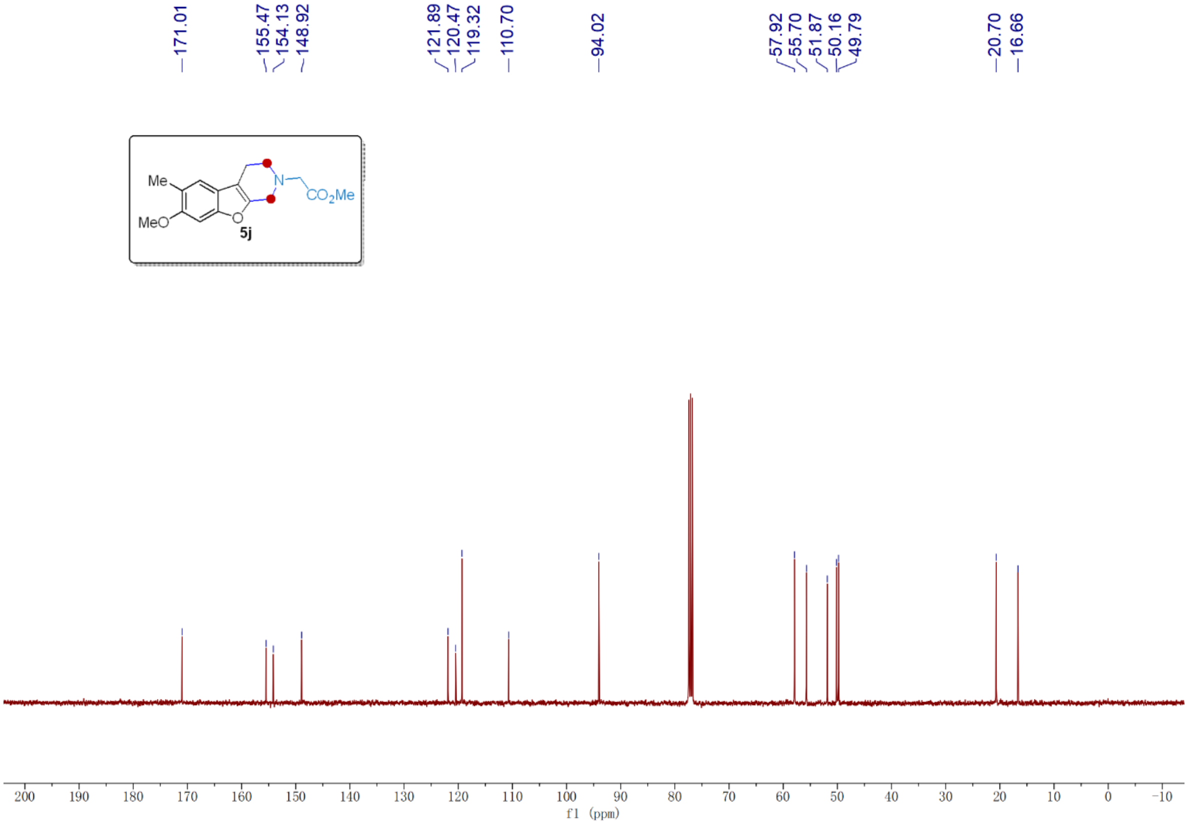

Supplementary Figure 120. ^{1}H-NMR spectrum of compound **5k** (500 MHz, $CDCl_3$)

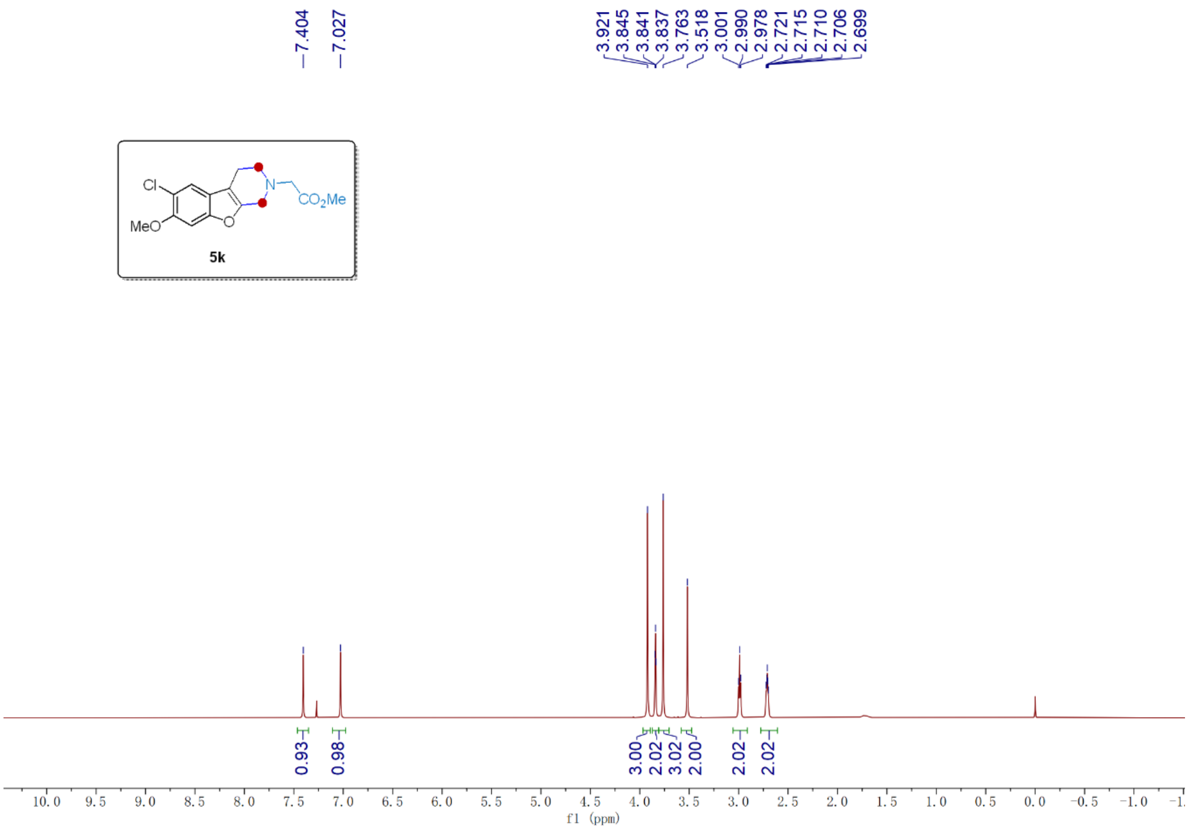

Supplementary Figure 121. ^{13}C-NMR spectrum of compound **5k** (125 MHz, $CDCl_3$)

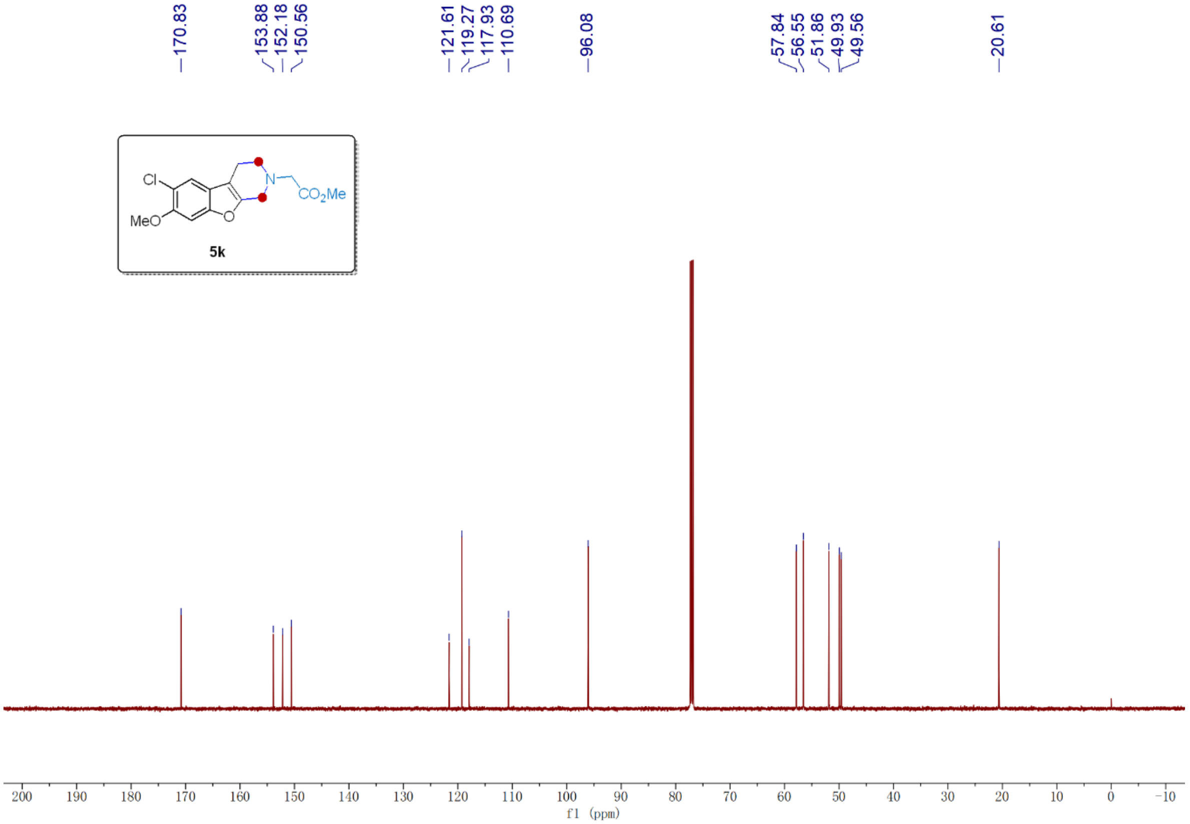

Supplementary Figure 122. ^{1}H-NMR spectrum of compound **5l** (600 MHz, $CDCl_3$)

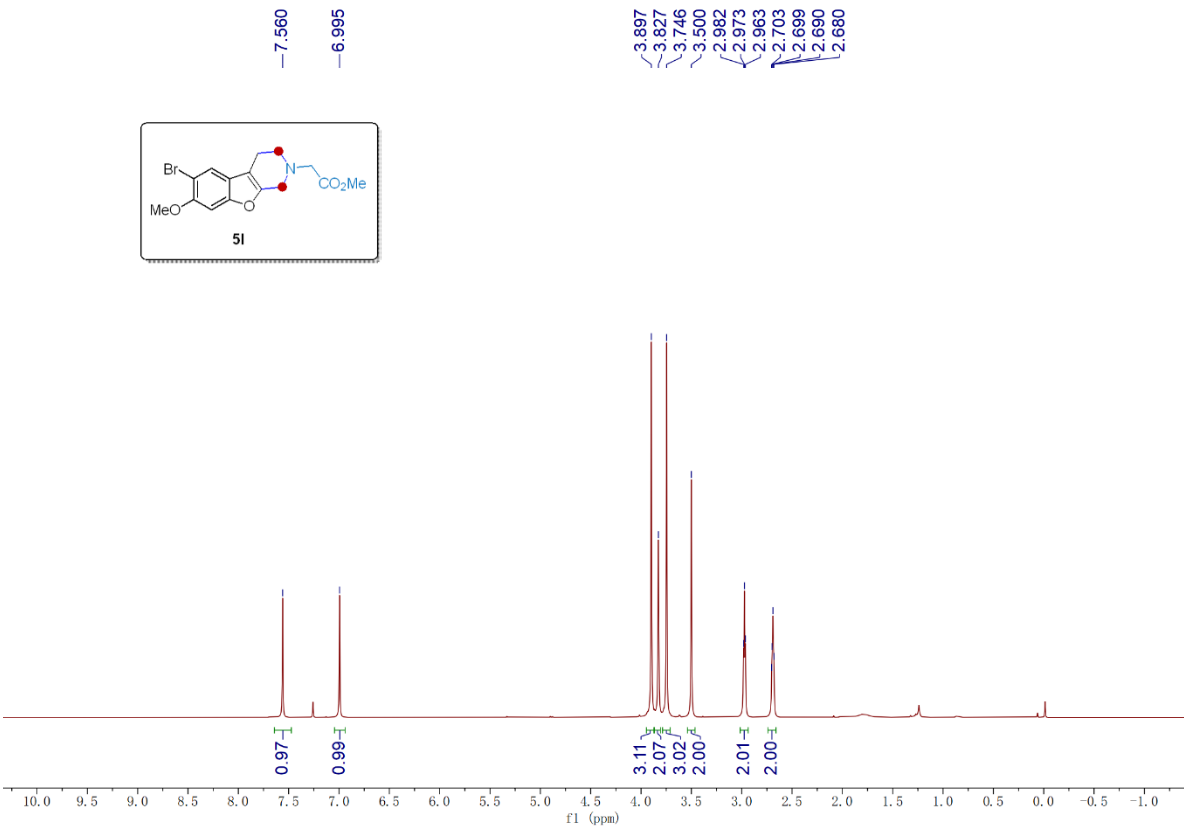

Supplementary Figure 123. ^{13}C-NMR spectrum of compound **5l** (100 MHz, $CDCl_3$)

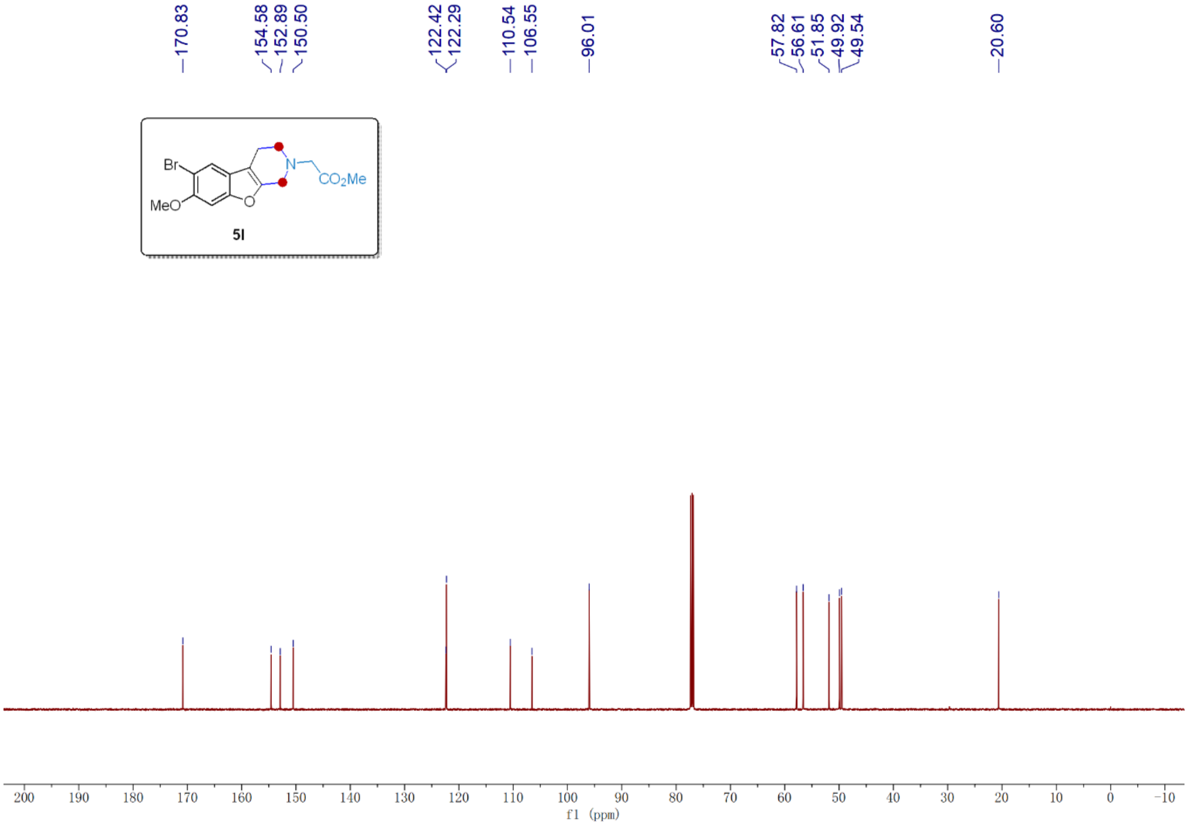

Supplementary Figure 124. ^{1}H-NMR spectrum of compound **5m** (600 MHz, DMSO)

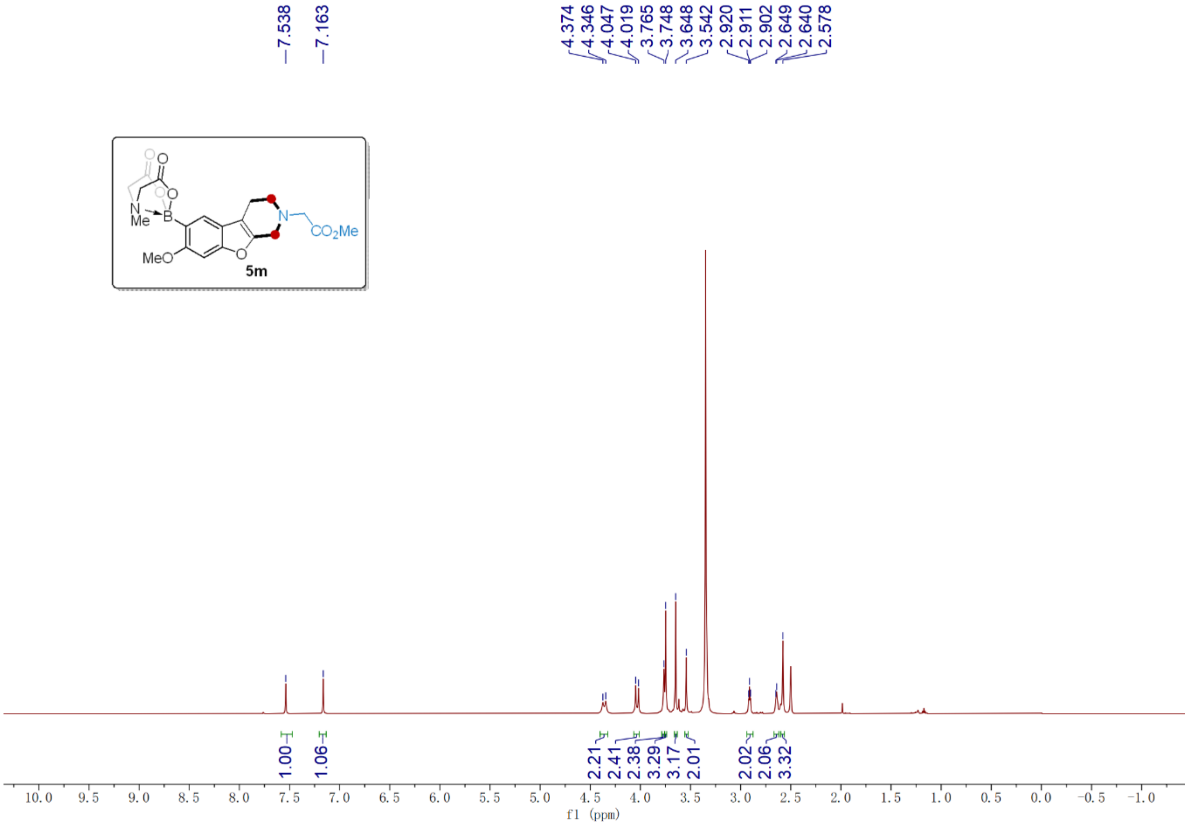

Supplementary Figure 125. ^{13}C-NMR spectrum of compound **5m** (125 MHz, DMSO)

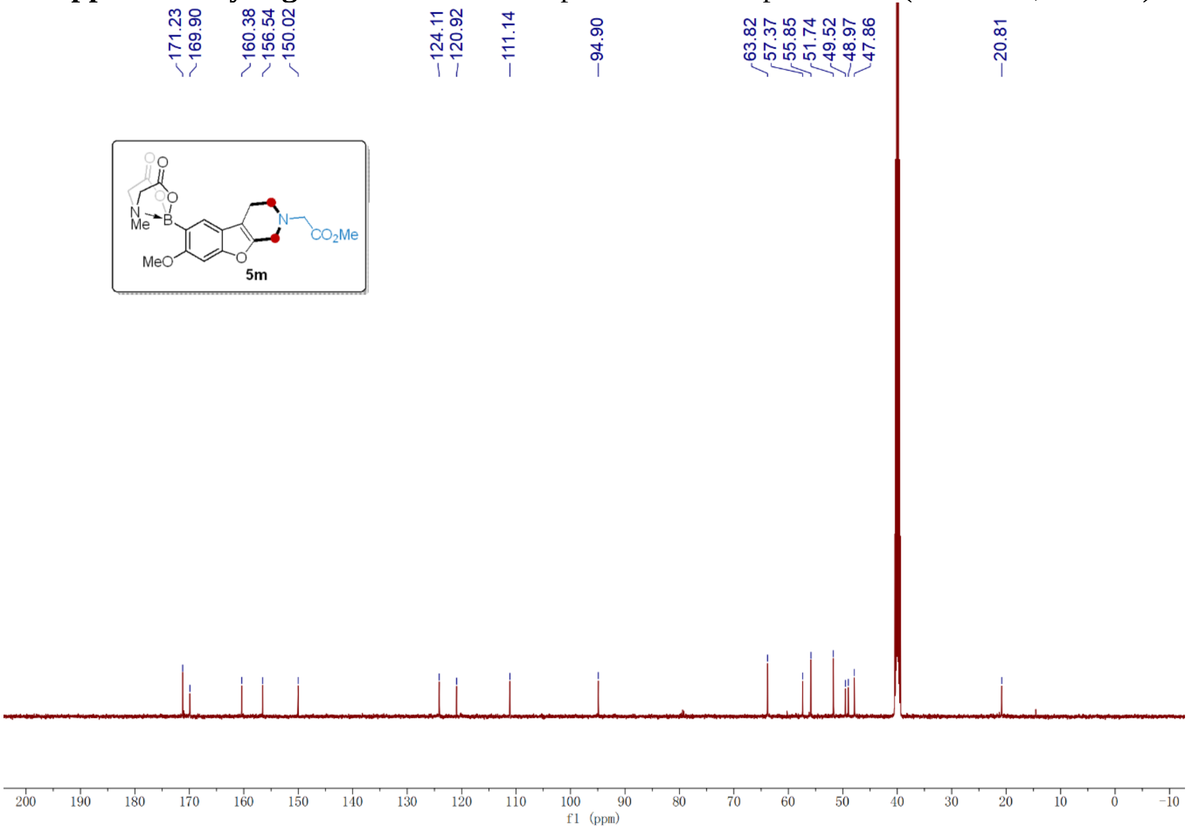

Supplementary Figure 126. ^{1}H-NMR spectrum of compound **5n** (600 MHz, $CDCl_3$)

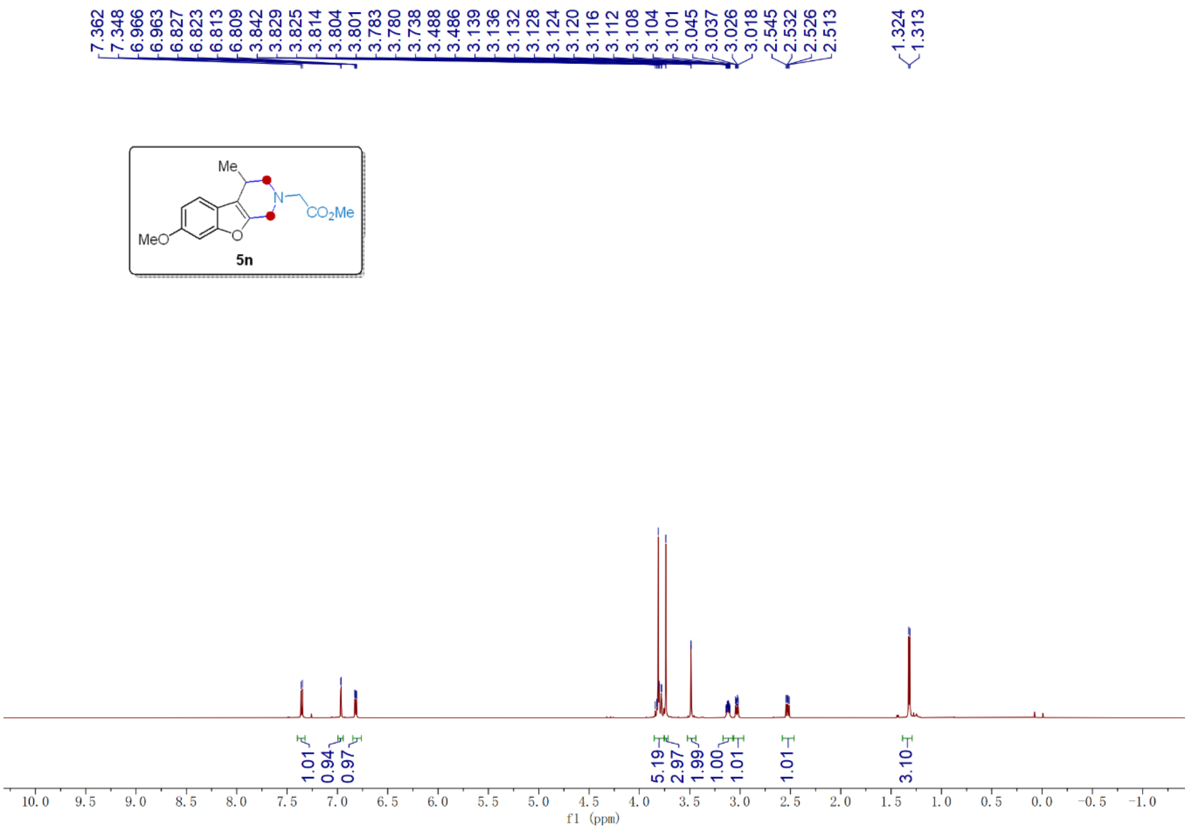

Supplementary Figure 127. ^{13}C-NMR spectrum of compound **5n** (150 MHz, $CDCl_3$)

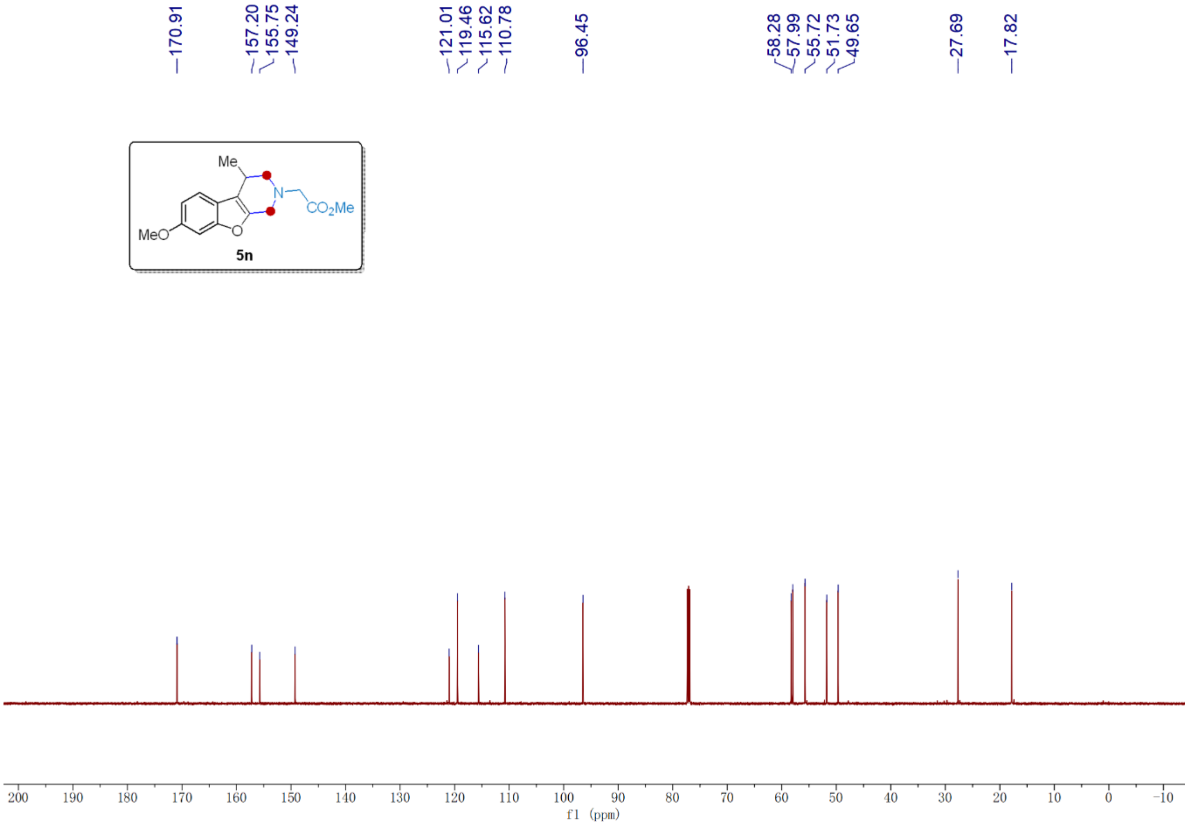

Supplementary Figure 128. ^{1}H-NMR spectrum of compound **5o** (500 MHz, $CDCl_3$)

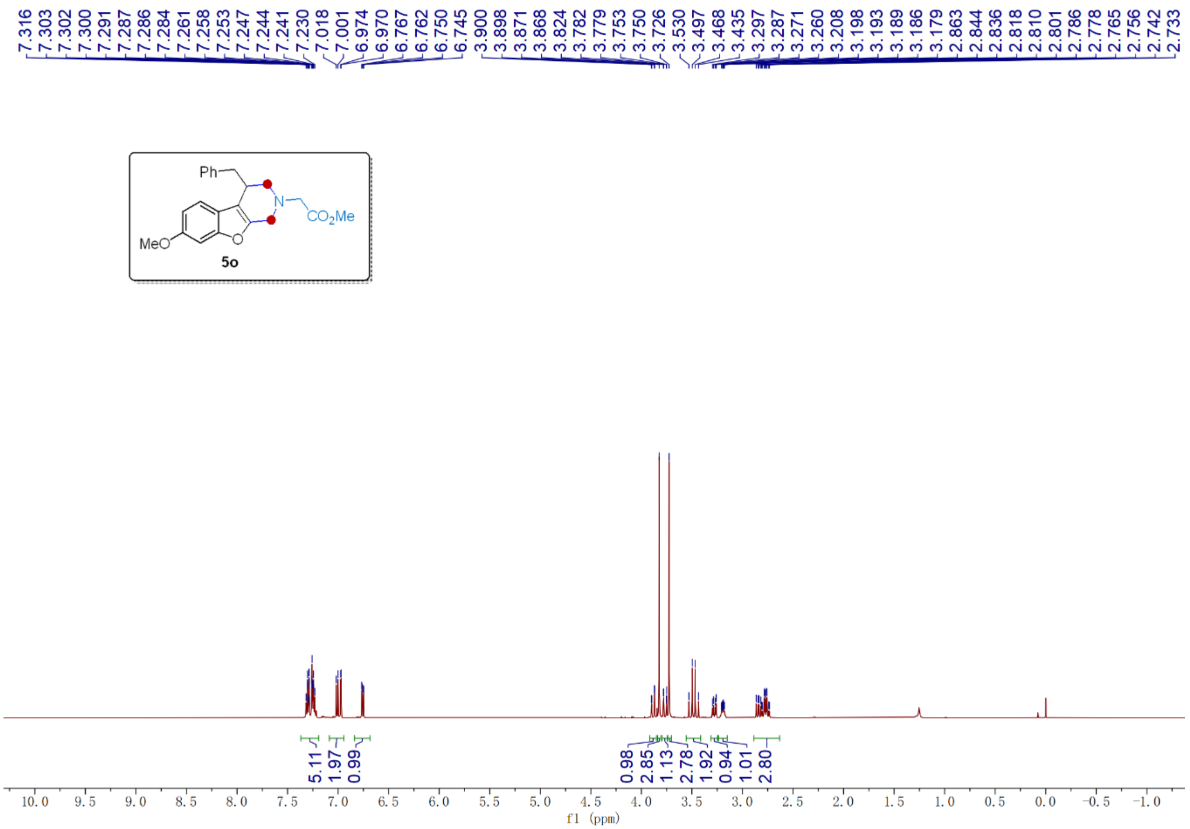

Supplementary Figure 129. ^{13}C-NMR spectrum of compound **5o** (125 MHz, $CDCl_3$)

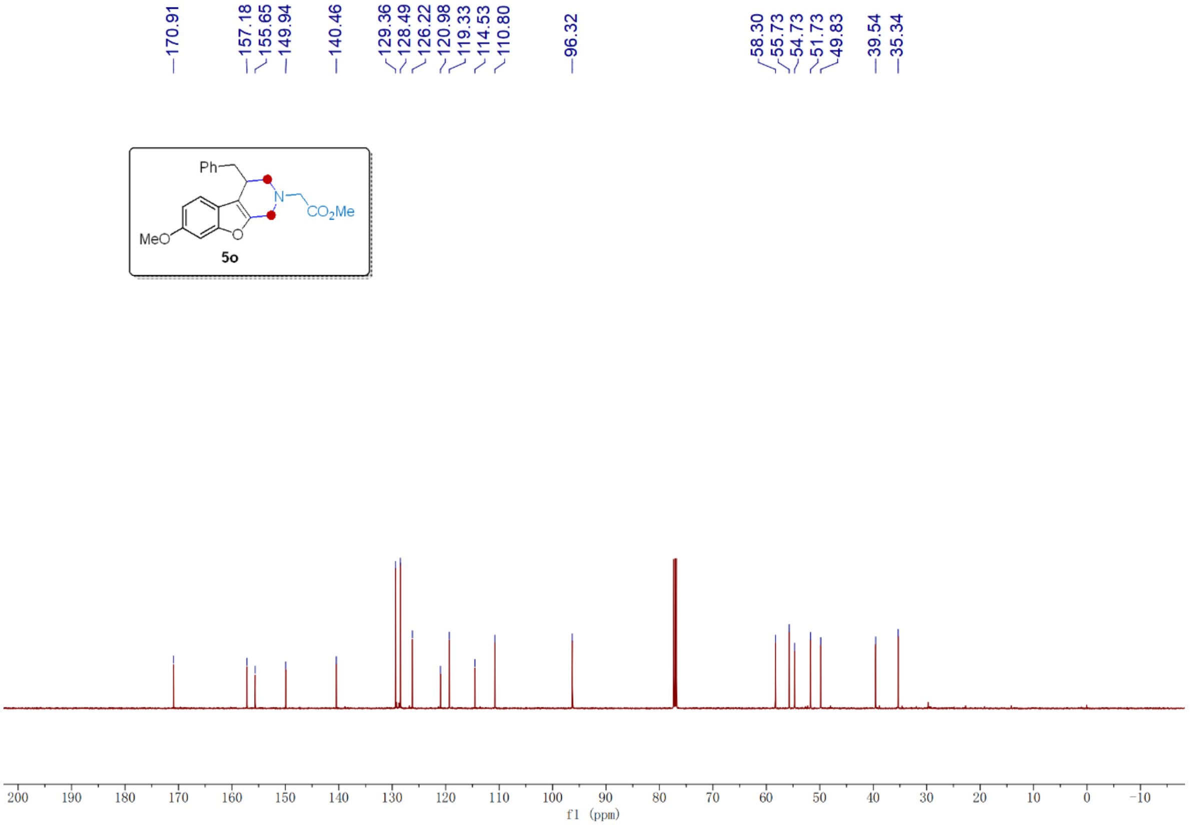

Supplementary Figure 130. ^{1}H-NMR spectrum of compound **5p** (500 MHz, $CDCl_3$)

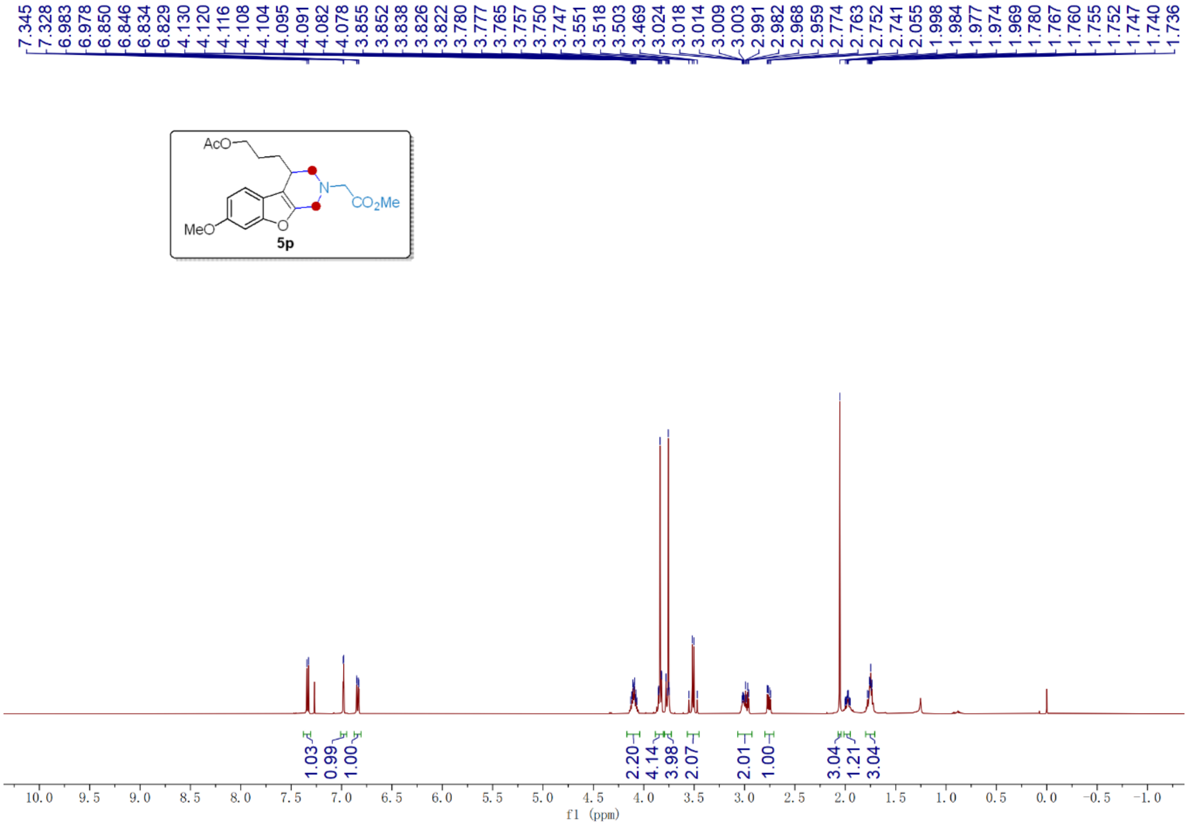

Supplementary Figure 131. ^{13}C-NMR spectrum of compound **5p** (125 MHz, $CDCl_3$)

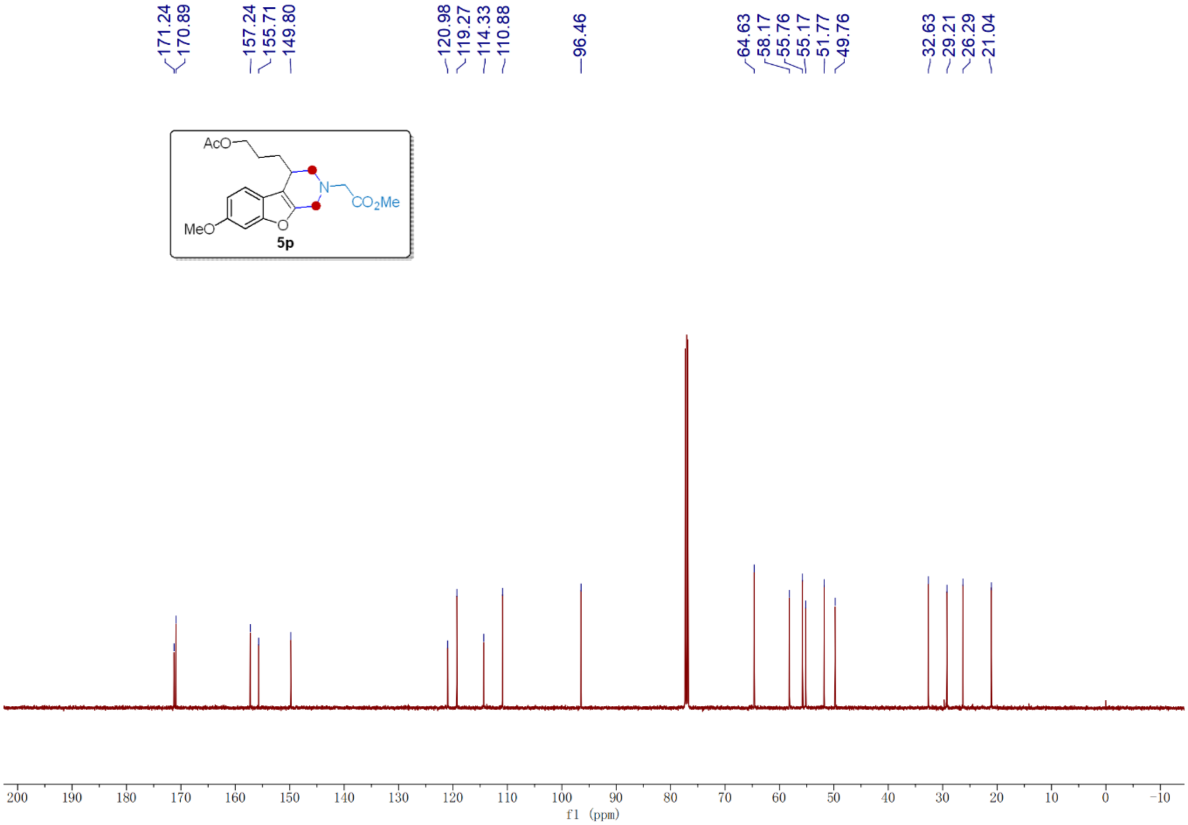

Supplementary Figure 132. ^{1}H-NMR spectrum of compound **5q** (600 MHz, $CDCl_3$)

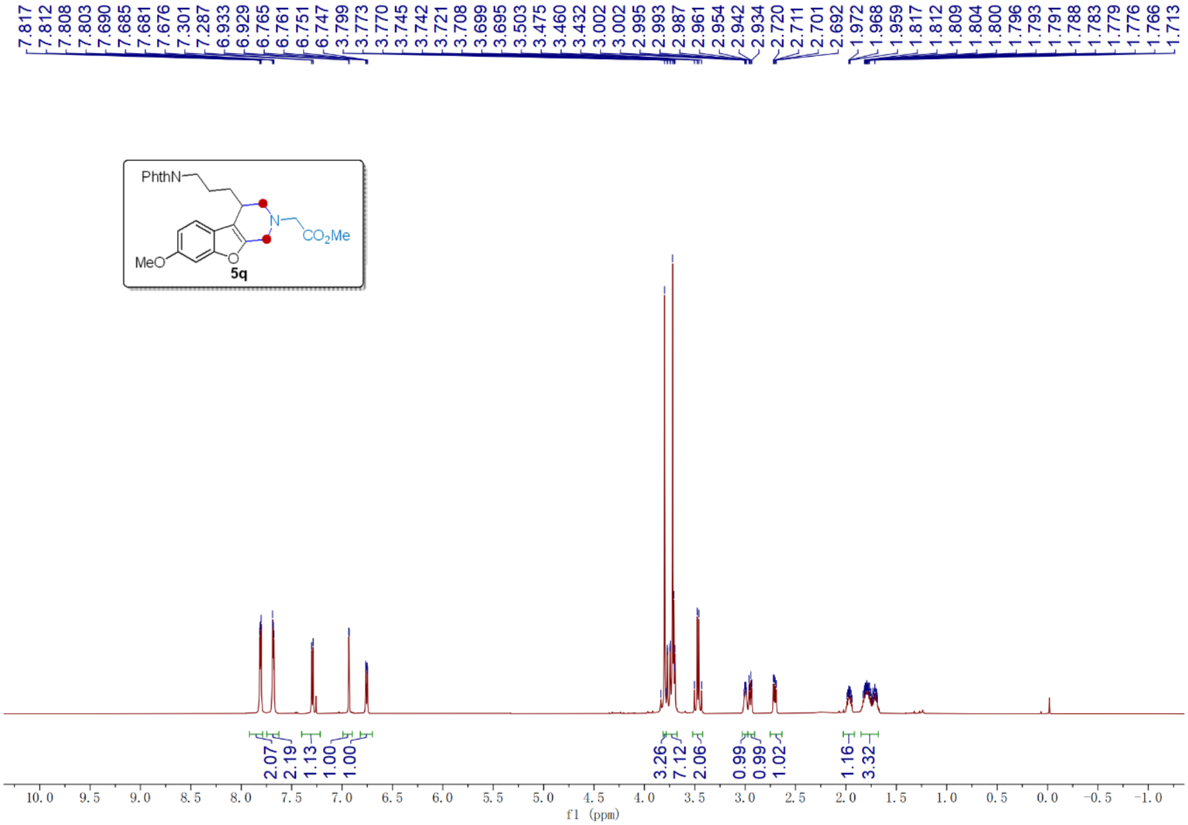

Supplementary Figure 133. ^{13}C-NMR spectrum of compound **5q** (150 MHz, $CDCl_3$)

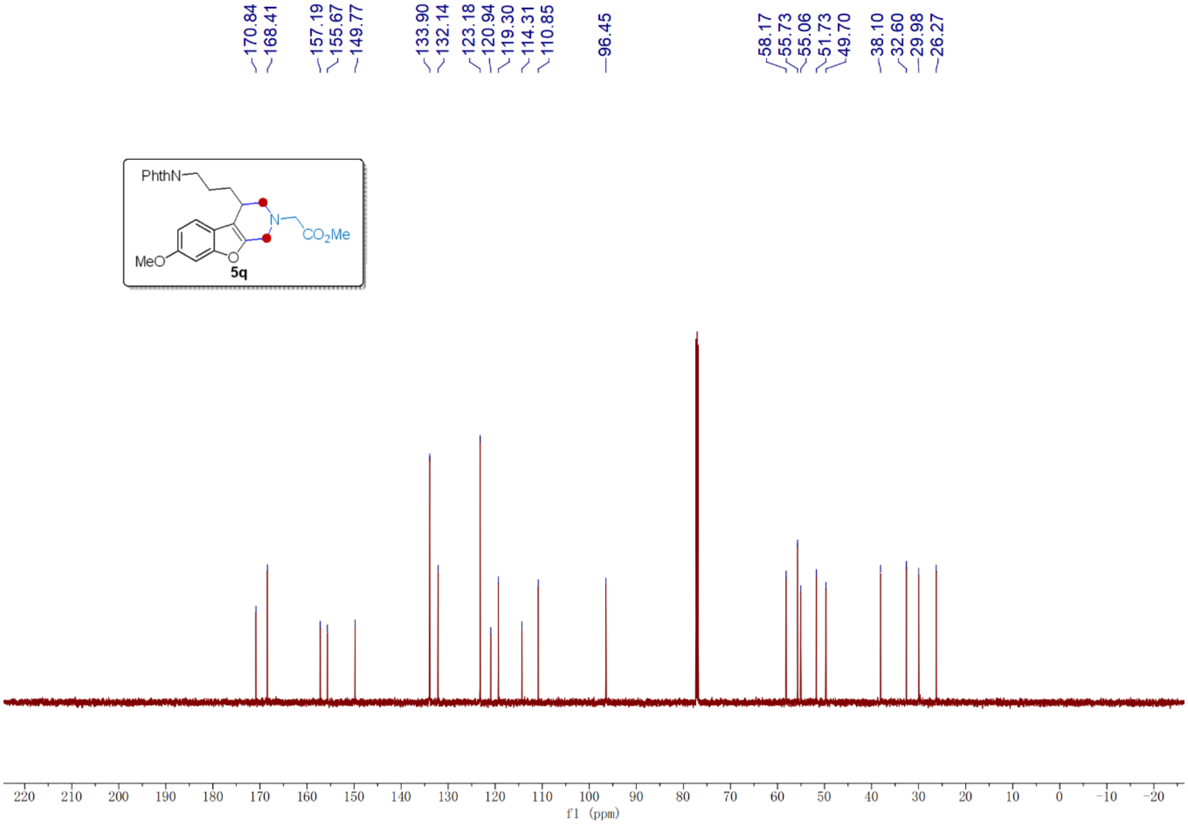

Supplementary Figure 134. ^{1}H-NMR spectrum of compound **5r** (500 MHz, $CDCl_3$)

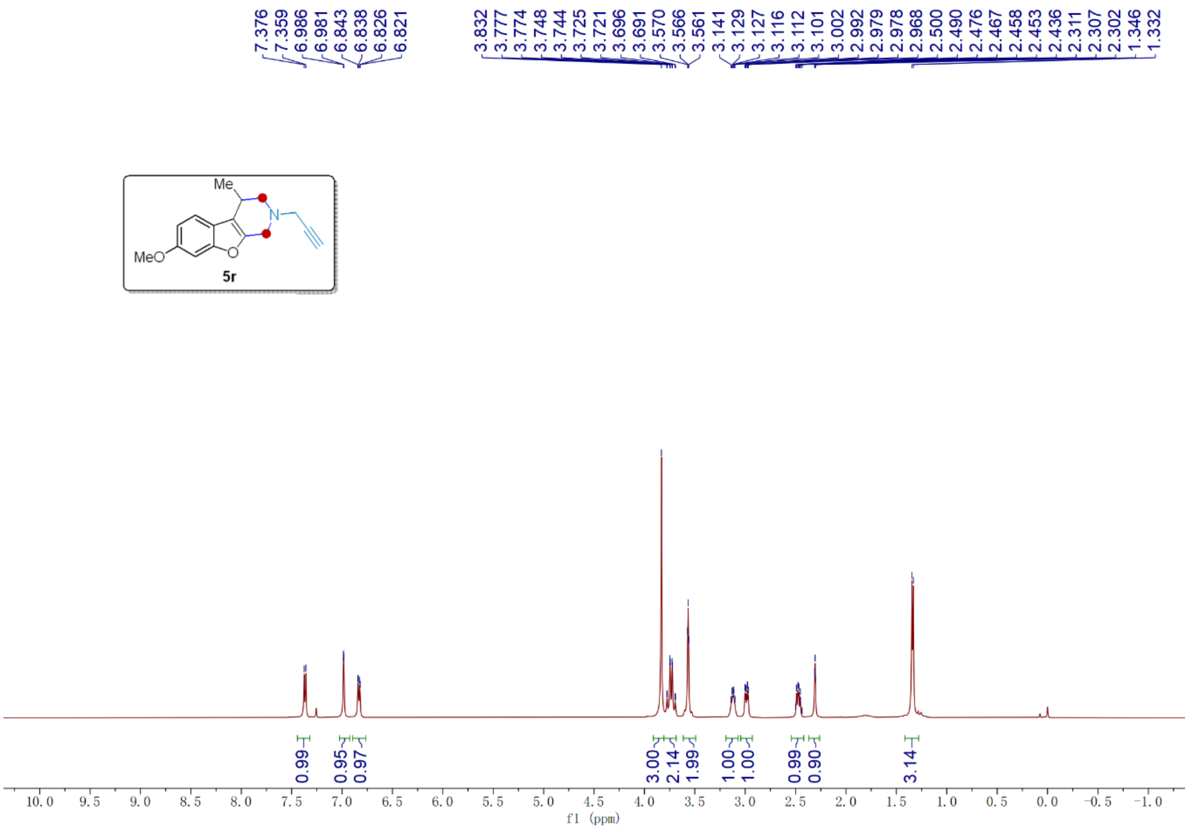

Supplementary Figure 135. ^{13}C-NMR spectrum of compound **5r** (125 MHz, $CDCl_3$)

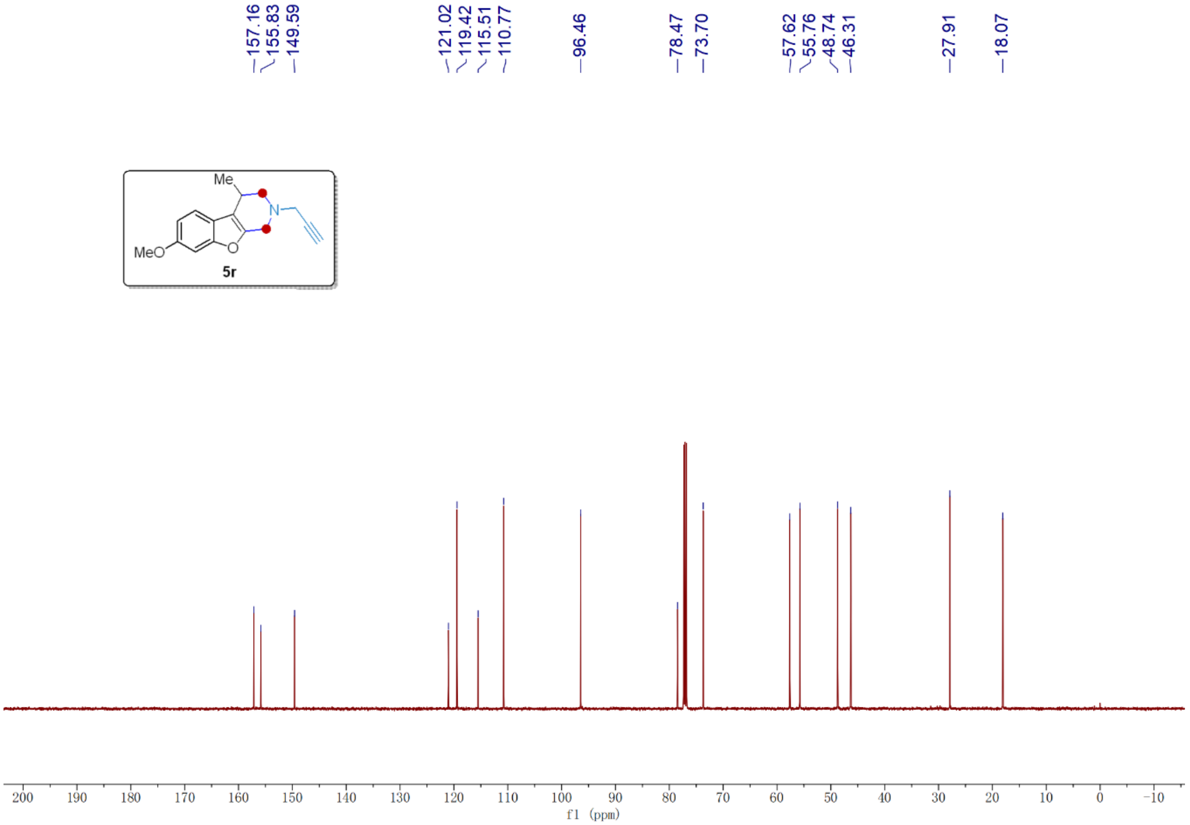

Supplementary Figure 136. ^{1}H-NMR spectrum of compound **5s** (500 MHz, $CDCl_3$)

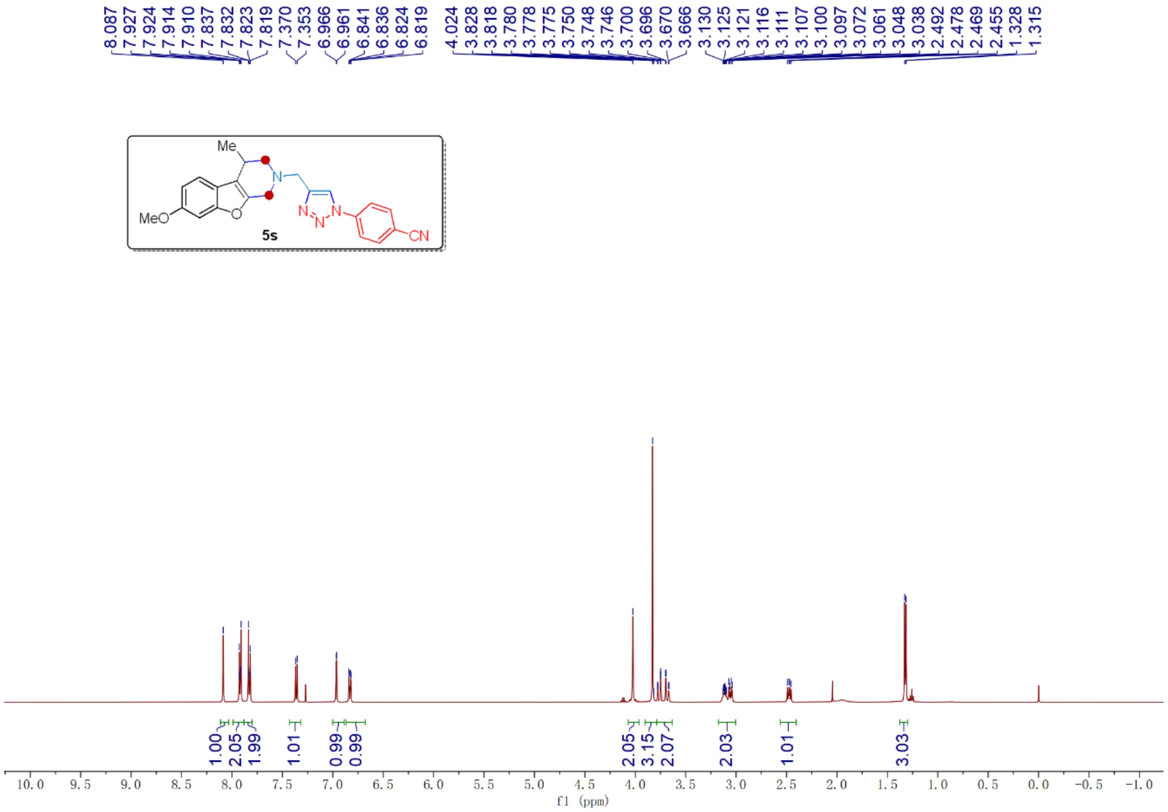

Supplementary Figure 137. ^{13}C-NMR spectrum of compound **5s** (125 MHz, $CDCl_3$)

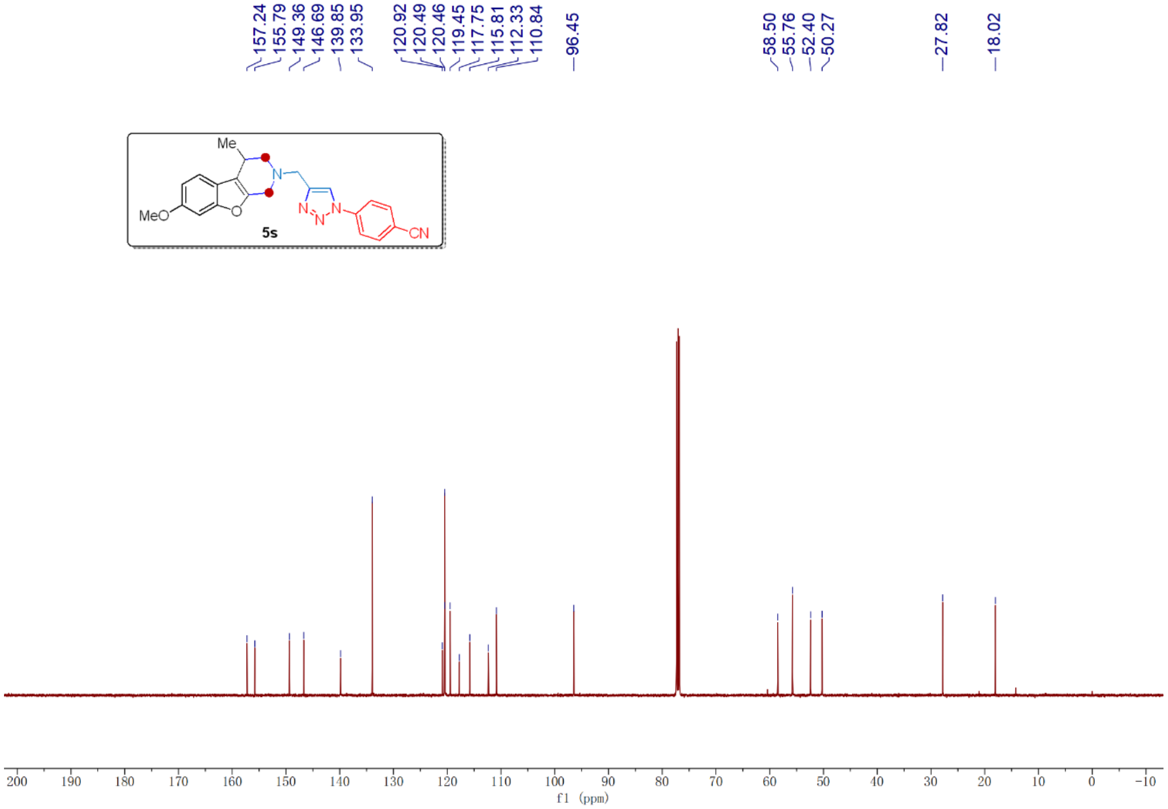

Supplementary Figure 138. ^{1}H-NMR spectrum of compound **5t** (600 MHz, $CDCl_3$)

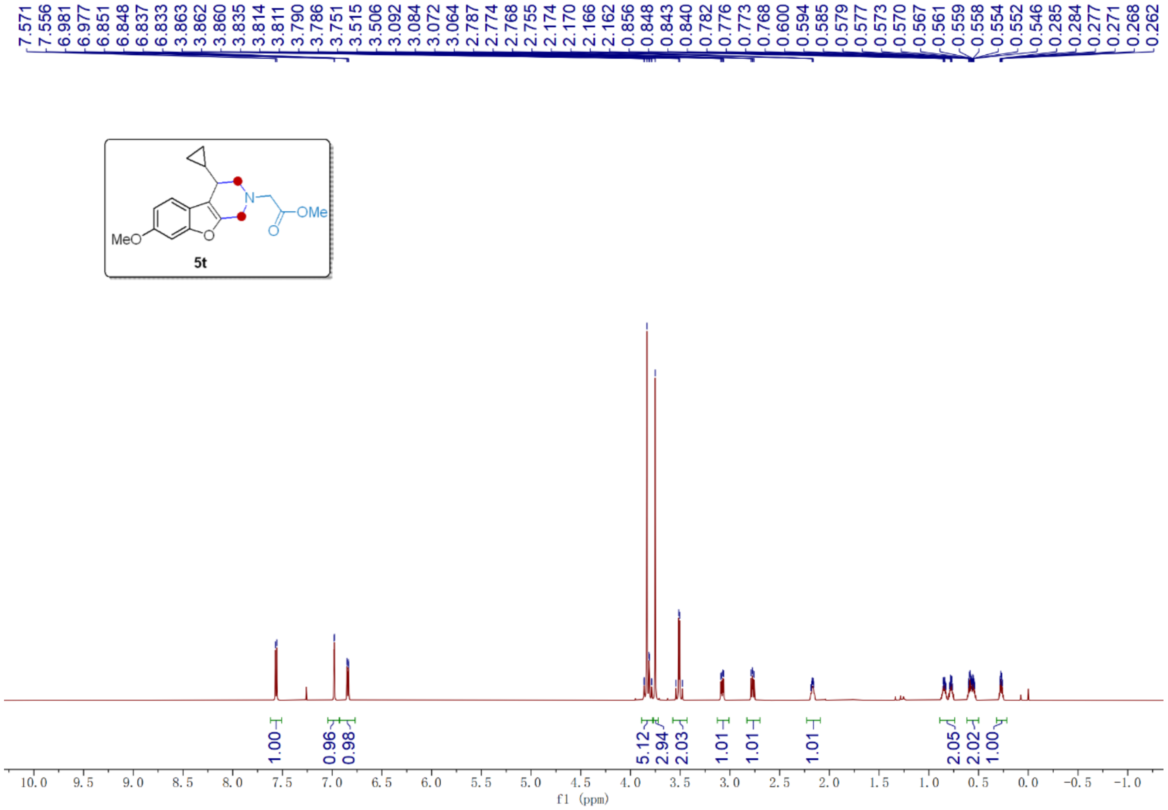

Supplementary Figure 139. ^{13}C-NMR spectrum of compound **5t** (125 MHz, $CDCl_3$)

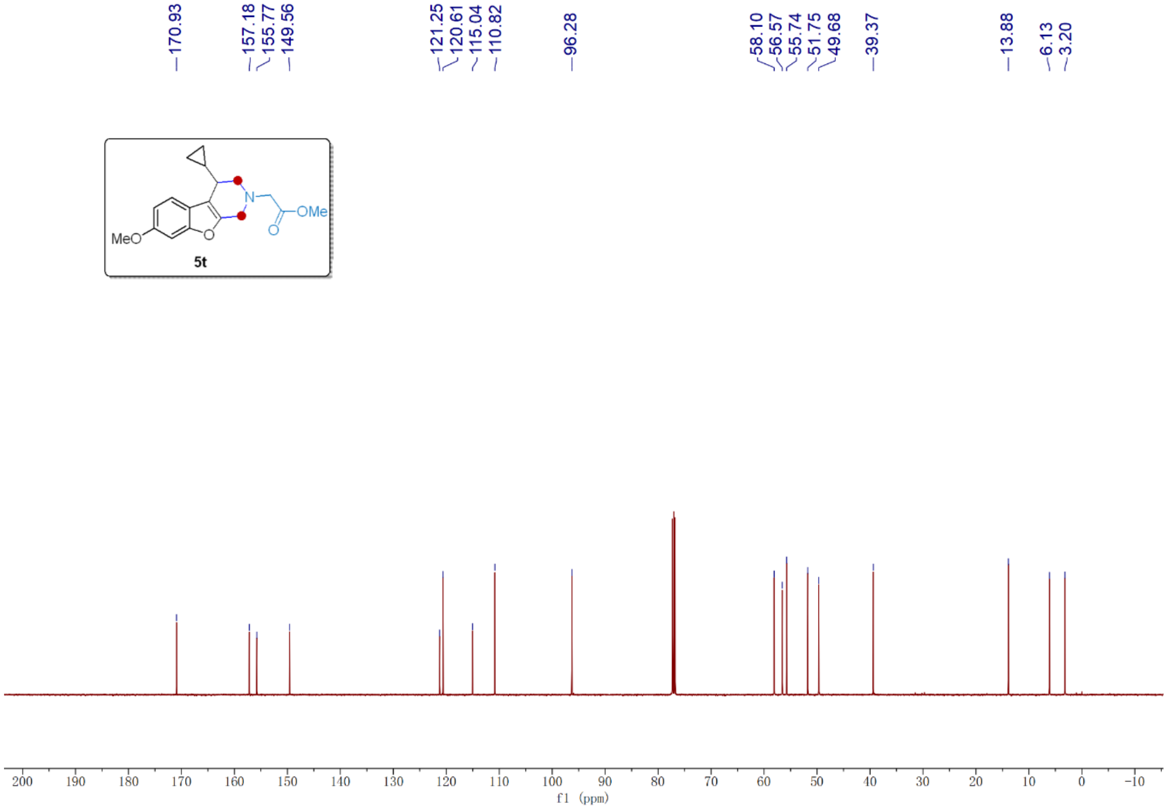

Supplementary Figure 140. ^{1}H-NMR spectrum of compound **6** (500 MHz, $CDCl_3$)

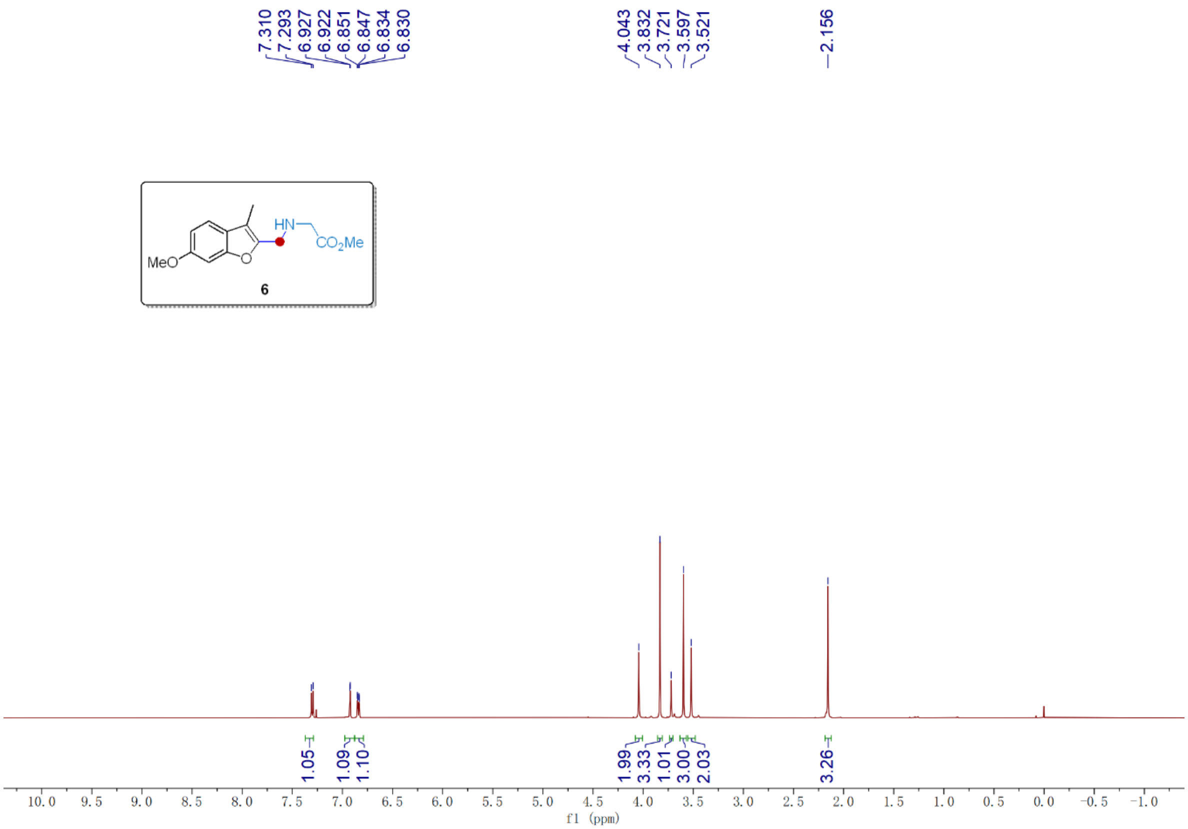

Supplementary Figure 141. ^{13}C-NMR spectrum of compound **6** (100 MHz, $CDCl_3$)

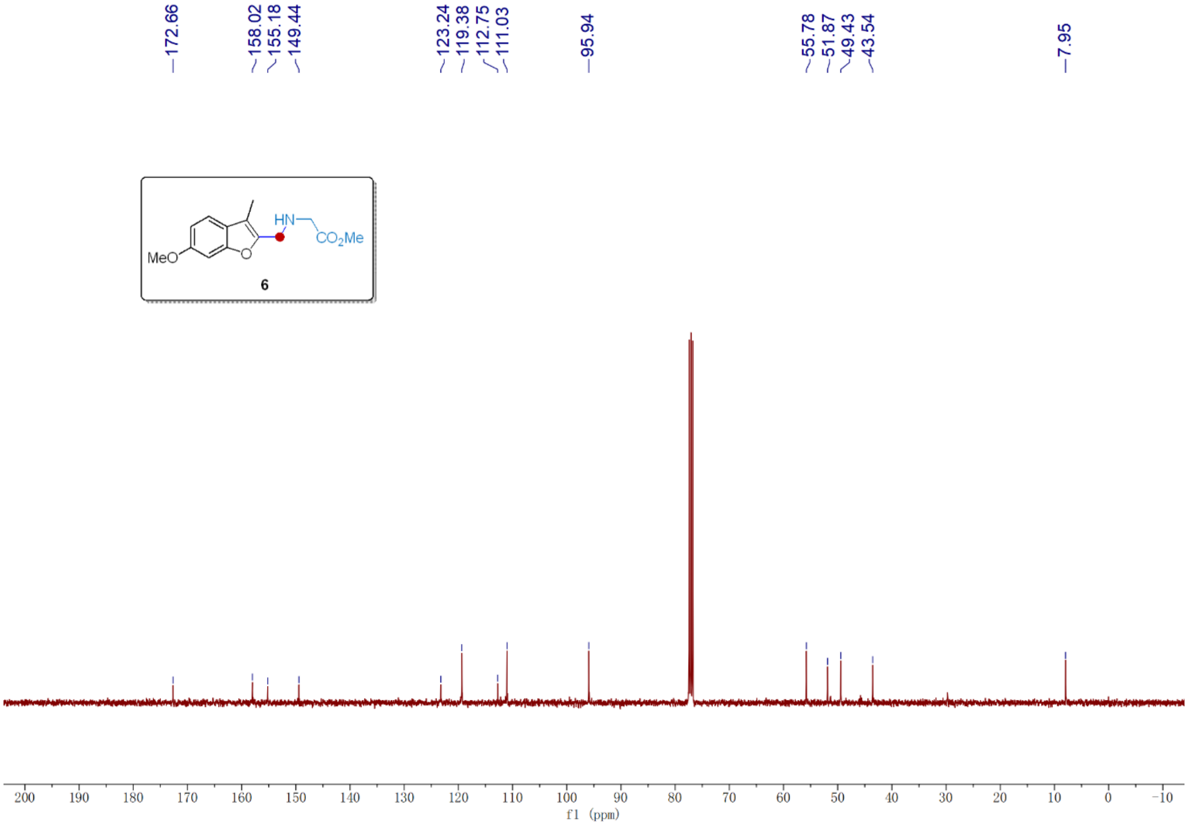

Supplementary Figure 142. ^{1}H-NMR spectrum of compound **7** (400 MHz, $CDCl_3$)

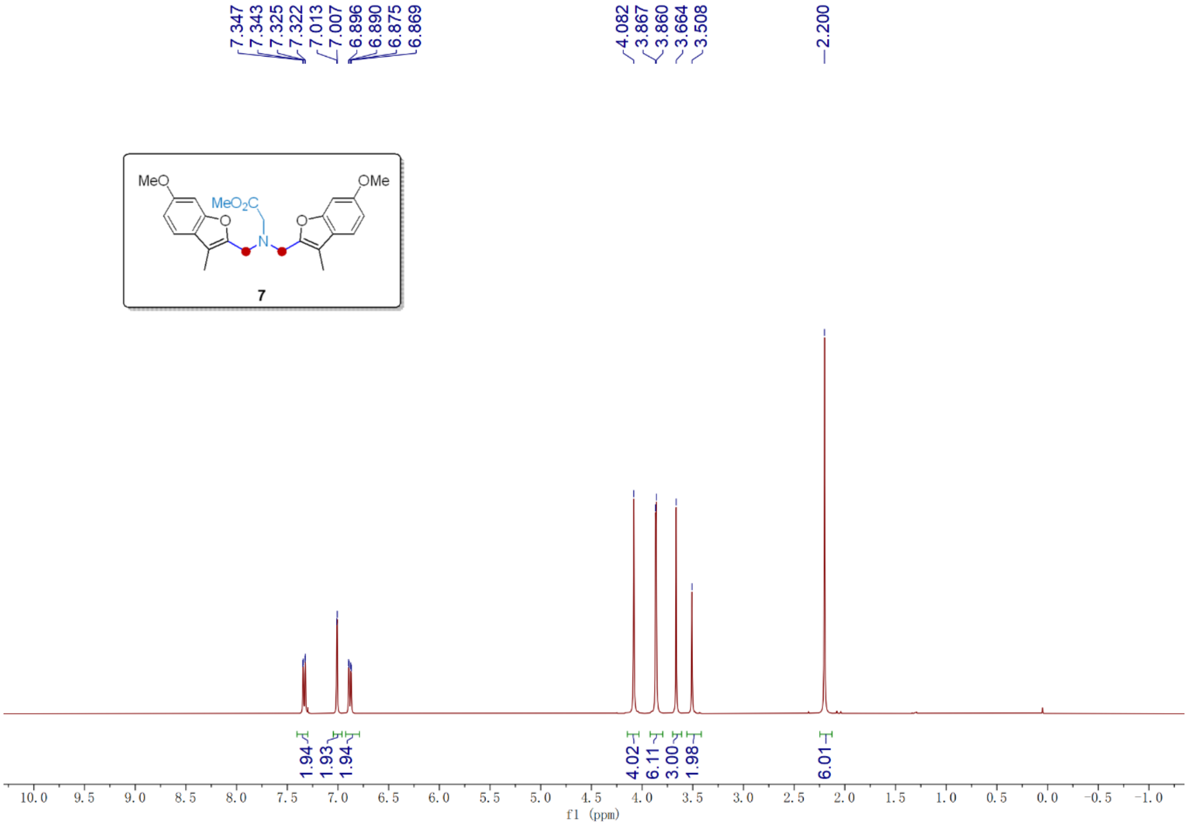

Supplementary Figure 143. ^{13}C-NMR spectrum of compound **7** (100 MHz, $CDCl_3$)

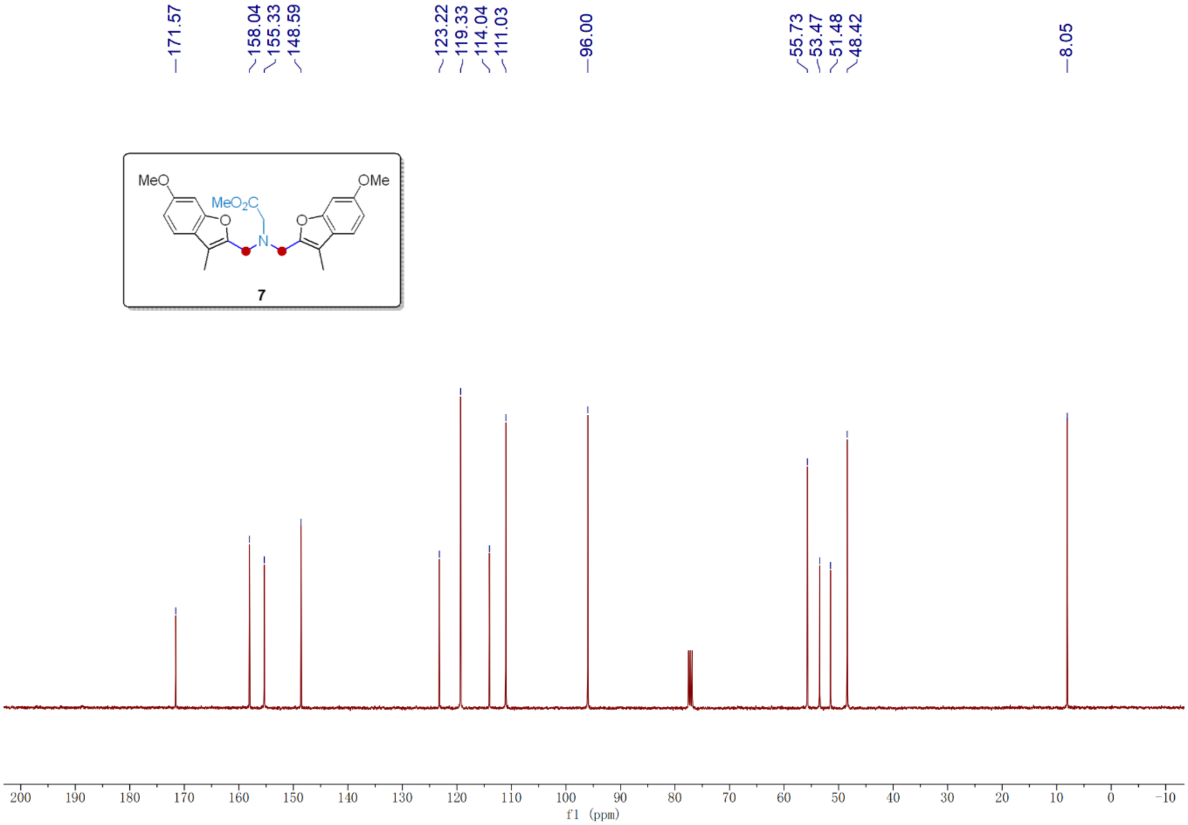

Supplementary Figure 144. ^{1}H-NMR spectrum of compound **[D$_2$]-4a** (500 MHz, $CDCl_3$)

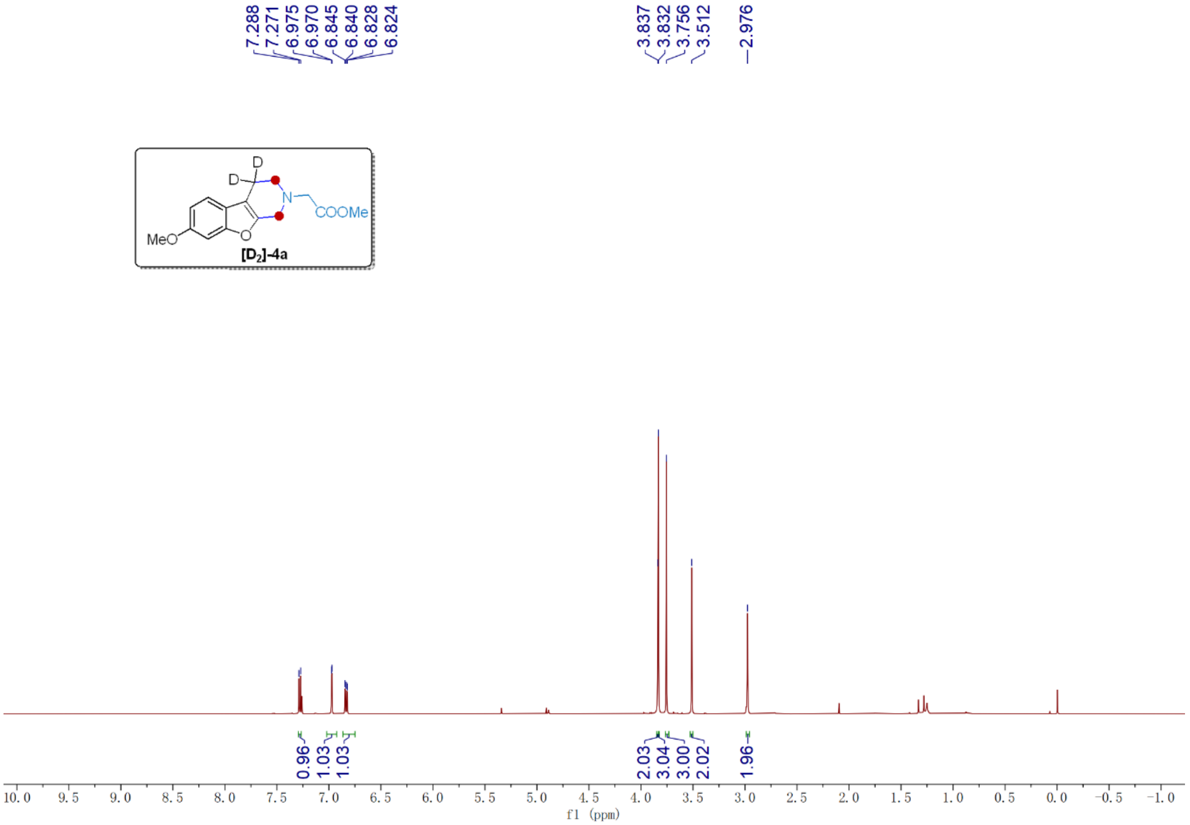

Supplementary Figure 145. ^{13}C-NMR spectrum of compound **[D$_2$]-4a** (125 MHz, $CDCl_3$)

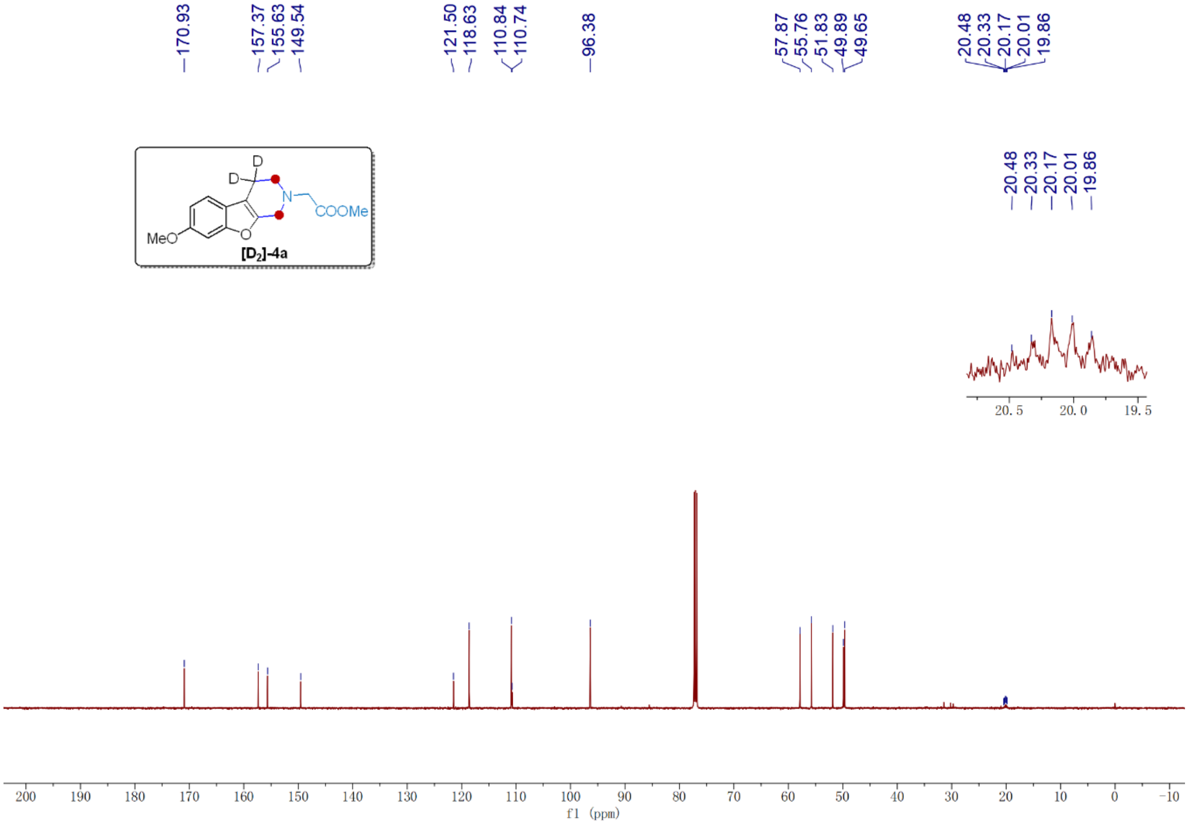

Supplementary Figure 146. ^{1}H-NMR for KIE (mixture of **[D$_2$]-4a** and **4a**, 500 MHz, $CDCl_3$)

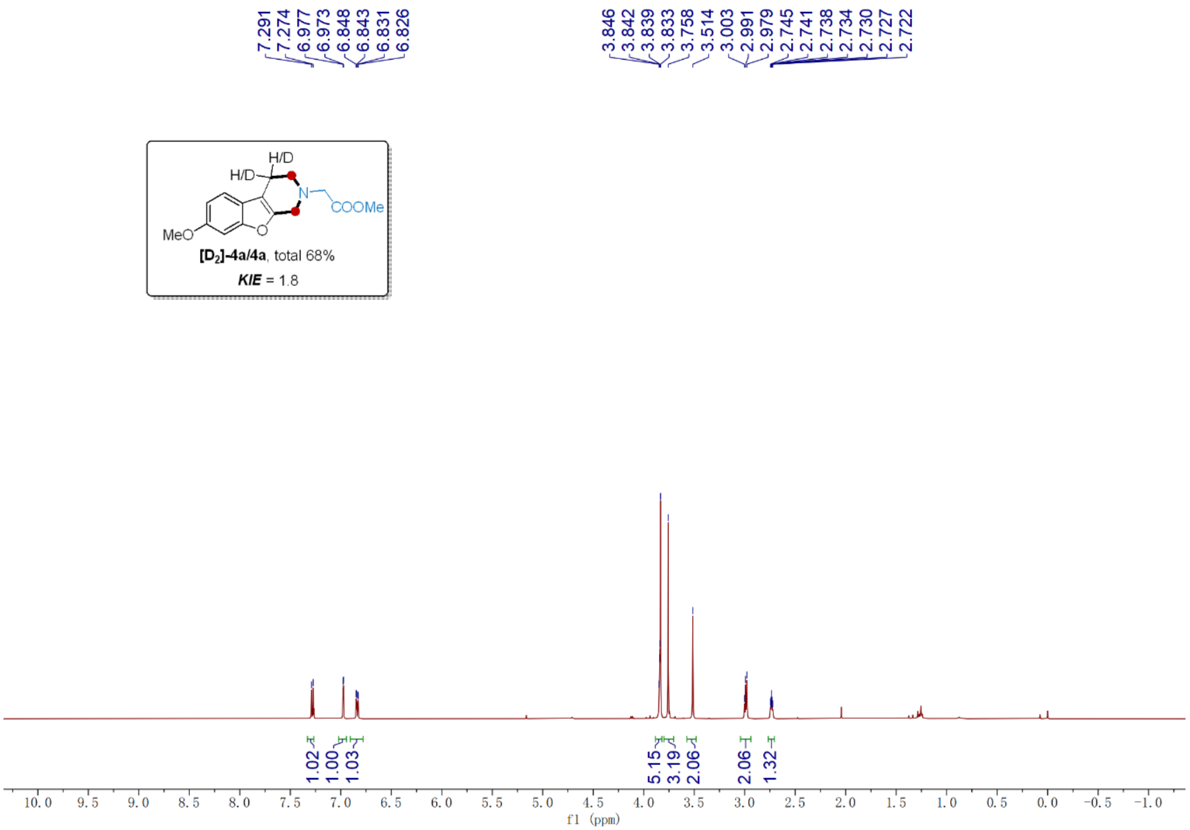

6. HPLC Charts

Supplementary Figure 147. 4d: IC-3 column (hexane/isopropanol = 95/5, flow = 1.0 mL/min) 254 nm

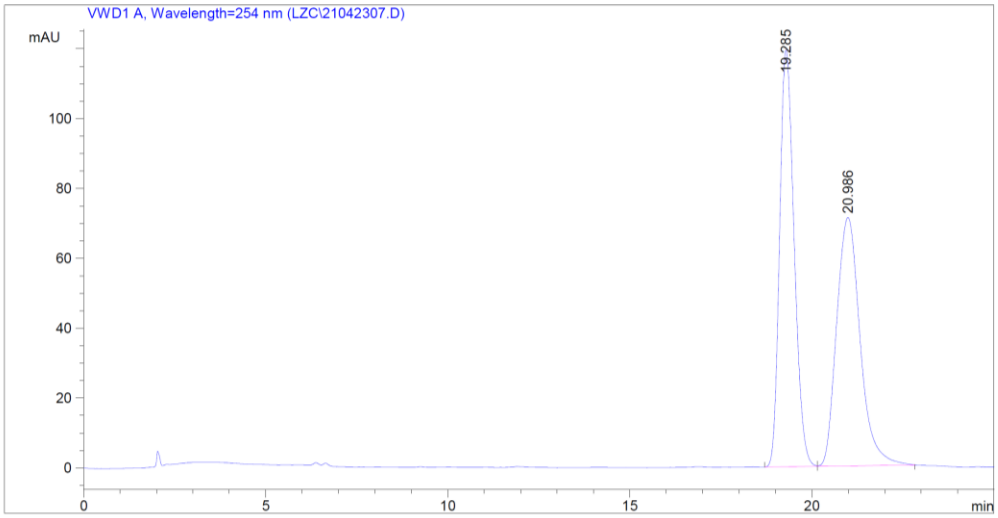

<Peak Table>

Detector A 254 nm

Peak#	Ret. Time	Area	Height	Conc.
**1**	19.285	3306	119	50.8821
**2**	20.986	3191	71	49.1179
**Total**		6497	190	

Supplementary Figure 148. <Chromatogram>

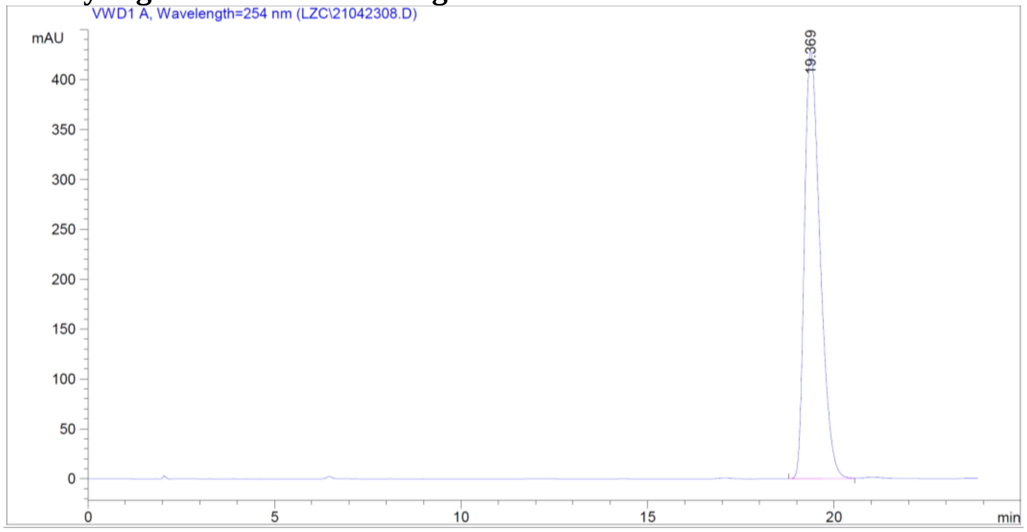

<Peak Table>

Detector A 254 nm

Peak#	Ret. Time	Area	Height	Conc.
1	19.369	12535	428	100
Total		12535	428	

Supplementary Figure 149. 4e: IC-3 column (hexane/isopropanol = 95/5, flow = 1.0 mL/min) 254 nm

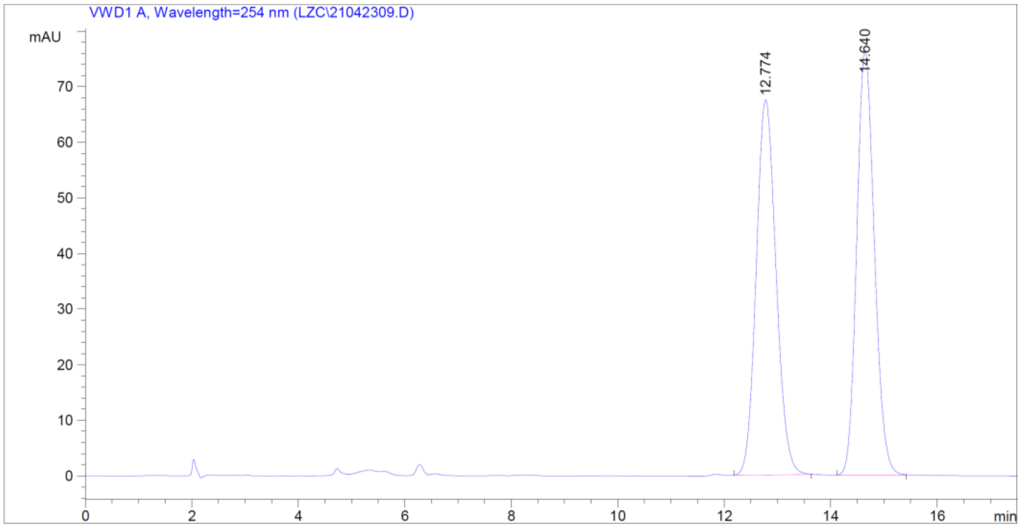

<Peak Table>

Detector A 254 nm

Peak#	Ret. Time	Area	Height	Conc.
1	12.774	1750	68	49.8550
2	14.640	1760	76	50.1450
Total		3510	144	

Supplementary Figure 150. **<Chromatogram>**

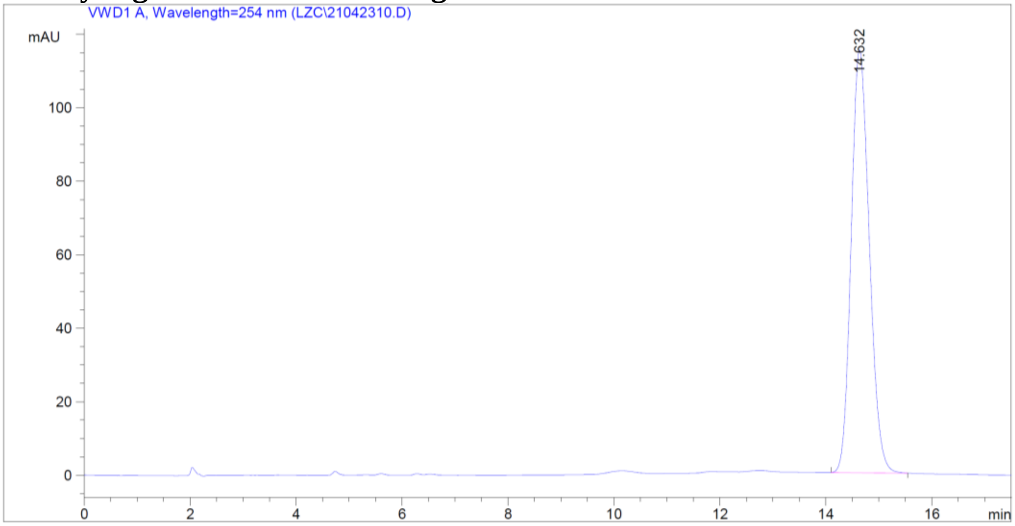

<Peak Table>

Detector A 254 nm

Peak#	Ret. Time	Area	Height	Conc.
**1**	14.632	2739	115	100
**Total**		2739	115	

Supplementary Figure 151. 4f: IC-3 column (hexane/isopropanol = 95/5, flow = 1.0 mL/min) 254 nm

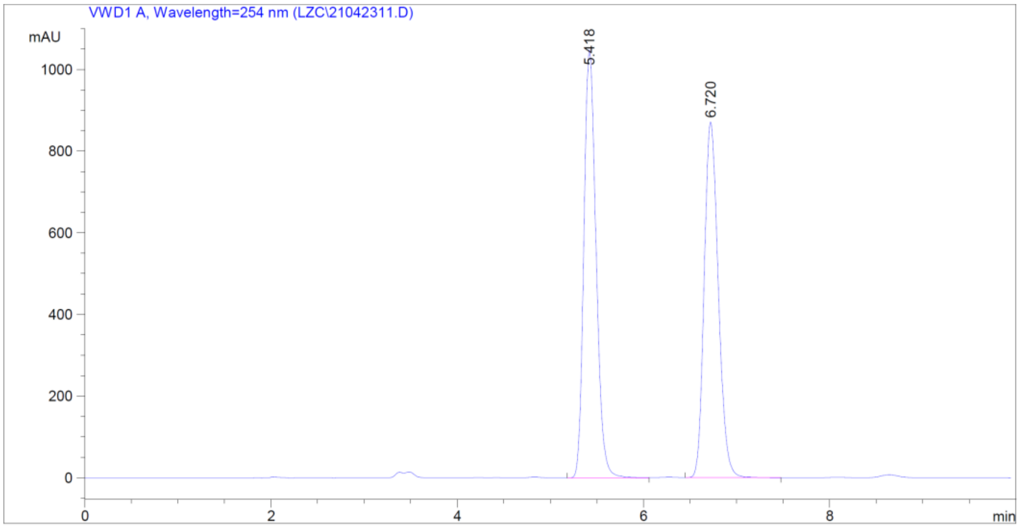

<Peak Table>

Detector A 254 nm

Peak#	Ret. Time	Area	Height	Conc.
**1**	5.418	9521	1048	50.9202
**2**	6.720	9177	871	49.0798
**Total**		18698	1919	

Supplementary Figure 152. <Chromatogram>

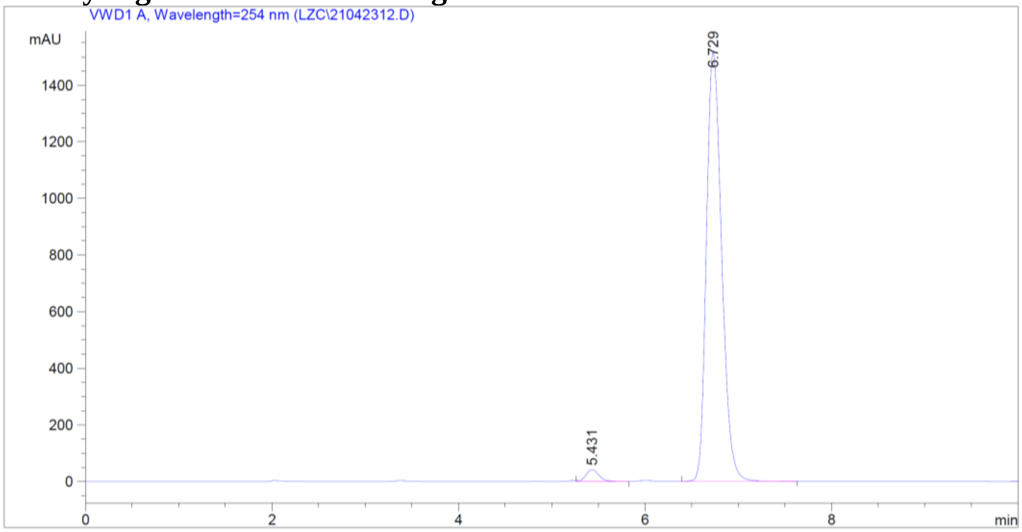

<Peak Table>

Detector A 254 nm

Peak#	Ret. Time	Area	Height	Conc.
**1**	5.431	415	42	2.3492
**2**	6.729	17247	1516	97.6508
**Total**		17662	1558	

Supplementary Figure 153. 4g: IC-3 column (hexane/isopropanol = 95/5, flow = 1.0 mL/min) 254 nm

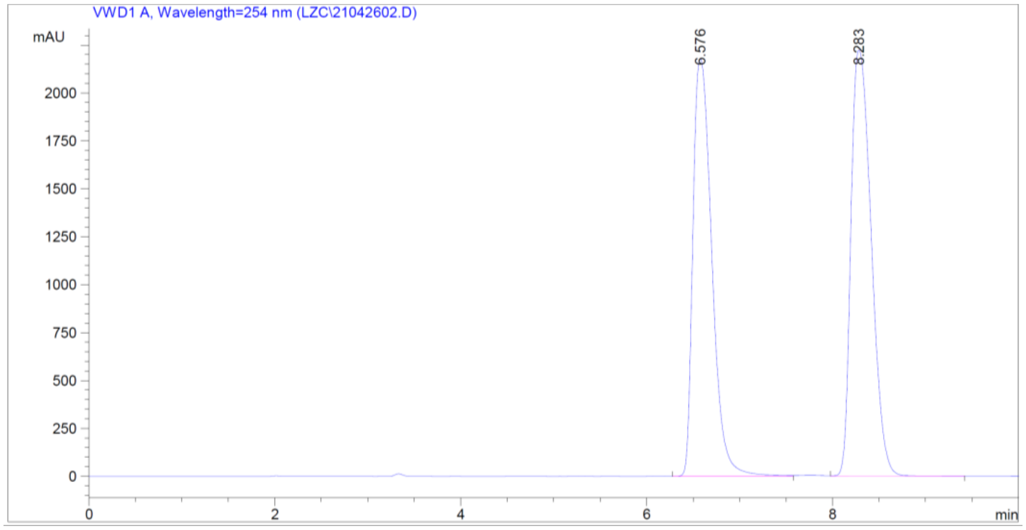

<Peak Table>

Detector A 254 nm

Peak#	Ret. Time	Area	Height	Conc.
**1**	6.576	30818	2181	47.7530
**2**	8.283	33718	2224	52.2470
**Total**		64536	4405	

Supplementary Figure 154. <Chromatogram>

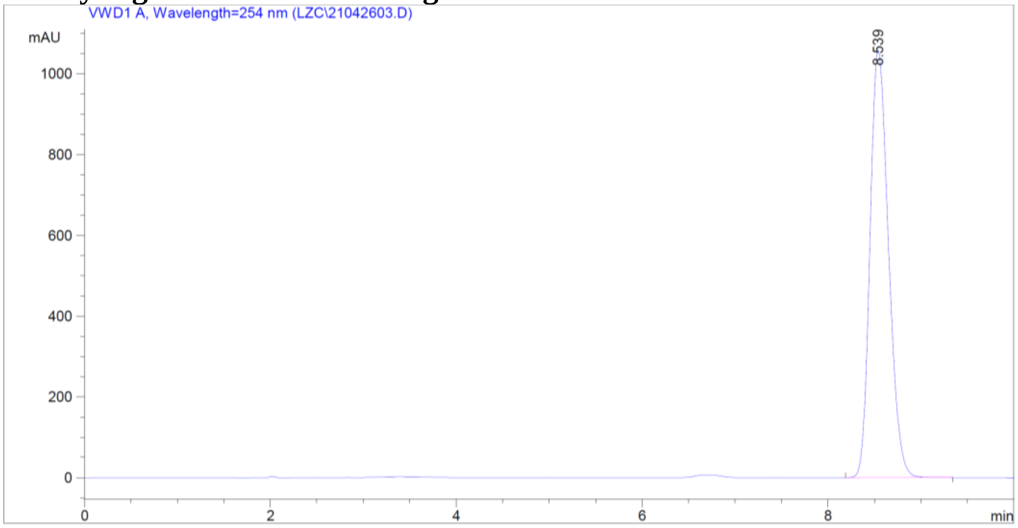

<Peak Table>

Detector A 254 nm

Peak#	Ret. Time	Area	Height	Conc.
1	8.539	14603	1057	100
Total		14603	1057	

Supplementary Figure 155. 4h: IE-3 column (hexane/isopropanol = 75/25, flow = 0.6 mL/min) 254 nm

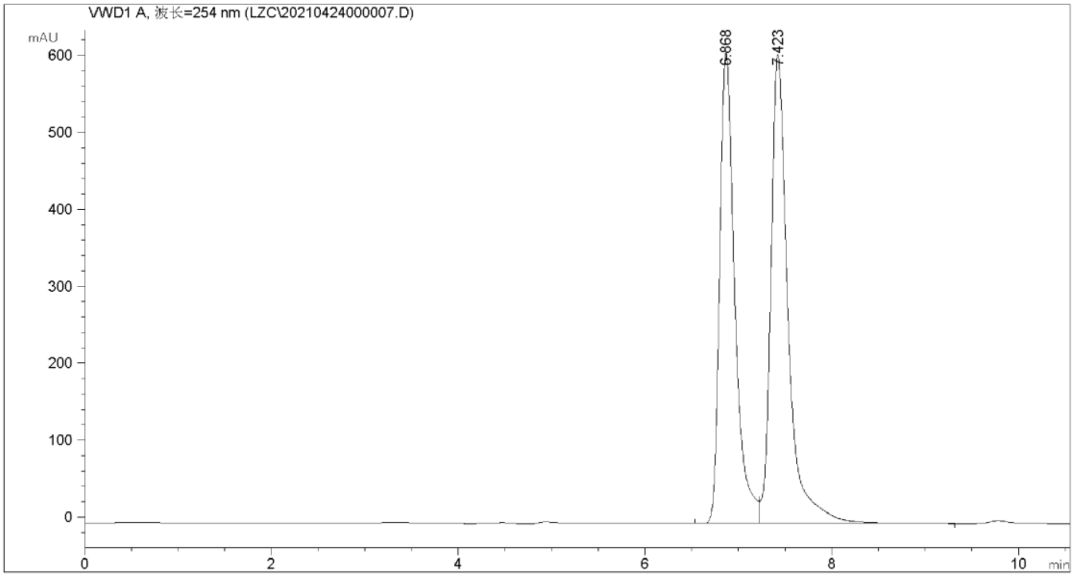

<Peak Table>

Detector A 254 nm

Peak#	Ret. Time	Area	Height	Conc.
1	6.868	6911	611	46.9782
2	7.423	7800	610	53.0218
Total		14711	1221	

Supplementary Figure 156. <Chromatogram>

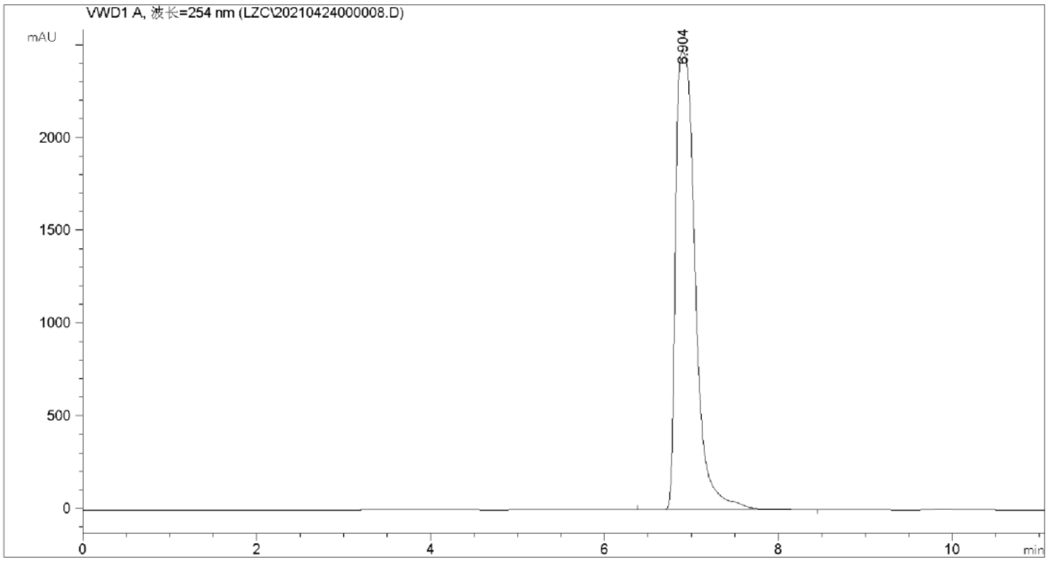

<Peak Table>
Detector A 254 nm

Peak#	Ret. Time	Area	Height	Conc.
**1**	6.904	38627	2469	100
**Total**		38627	2469	

Supplementary Figure 157. 4j: IC-3 column (hexane/isopropanol = 95/5, flow = 1.0 mL/min) 254 nm

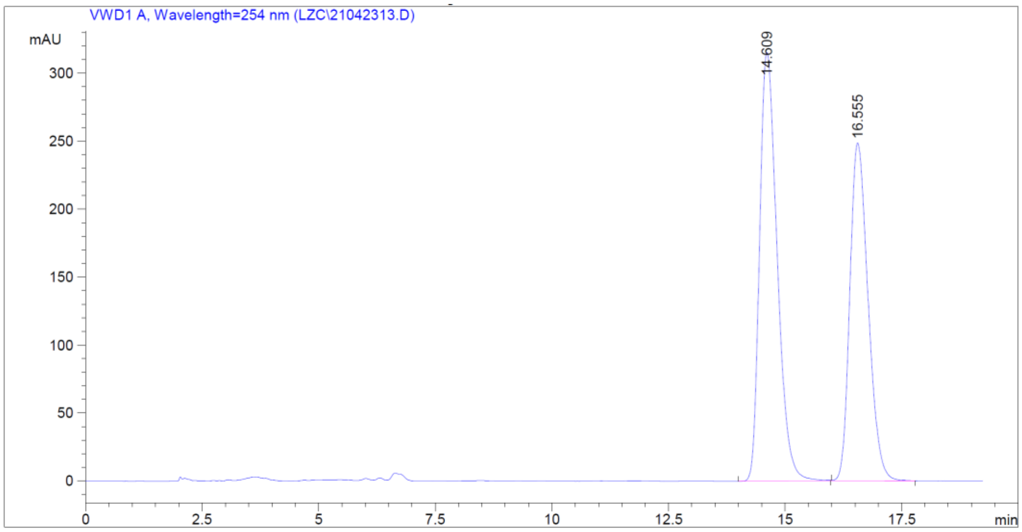

<Peak Table>
Detector A 254 nm

Peak#	Ret. Time	Area	Height	Conc.
**1**	14.609	8258	315	54.9654
**2**	16.555	6766	248	45.0346
**Total**		15024	563	

Supplementary Figure 158. <Chromatogram>

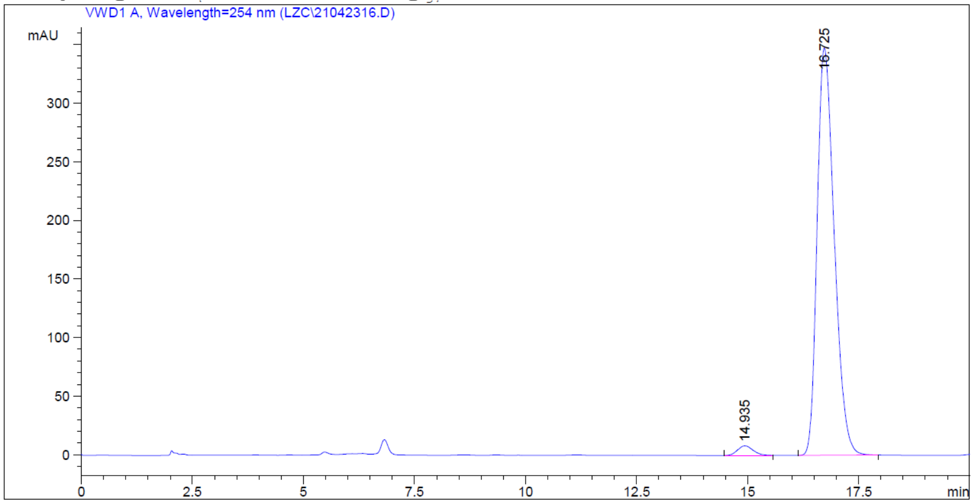

<Peak Table>

Detector A 254 nm

Peak#	Ret. Time	Area	Height	Conc.
1	14.935	200	8	2.1076
2	16.725	9317	347	97.8924
Total		9517	355	

Supplementary Figure 159. 4k: IE-3 column (hexane/isopropanol = 70/30, flow = 0.6 mL/min) 254 nm

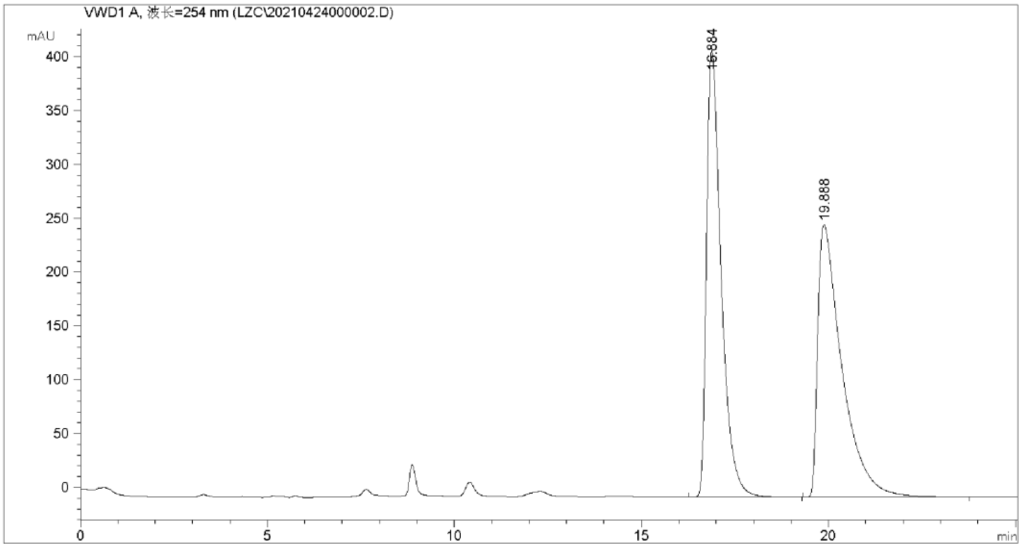

<Peak Table>

Detector A 254 nm

Peak#	Ret. Time	Area	Height	Conc.
1	16.884	11304	414	49.9937
2	19.888	11307	253	50.0063
Total		22611	667	

Supplementary Figure 160. <Chromatogram>

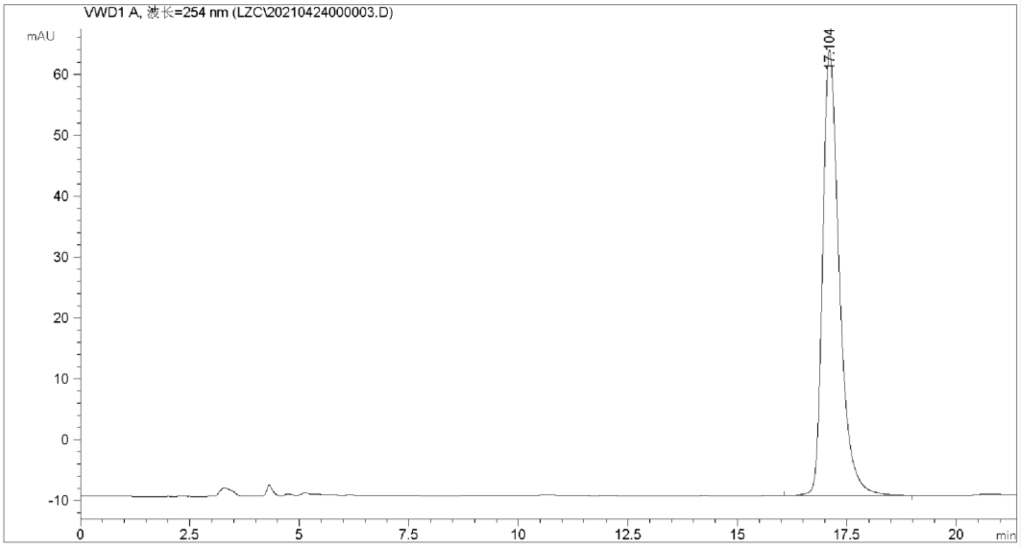

<Peak Table>

Detector A 254 nm

Peak#	Ret. Time	Area	Height	Conc.
**1**	17.104	1930	73	100
**Total**		1930	73	

Supplementary Figure 161. 4l: IE-3 column (hexane/isopropanol = 70/30, flow = 0.6 mL/min) 254 nm

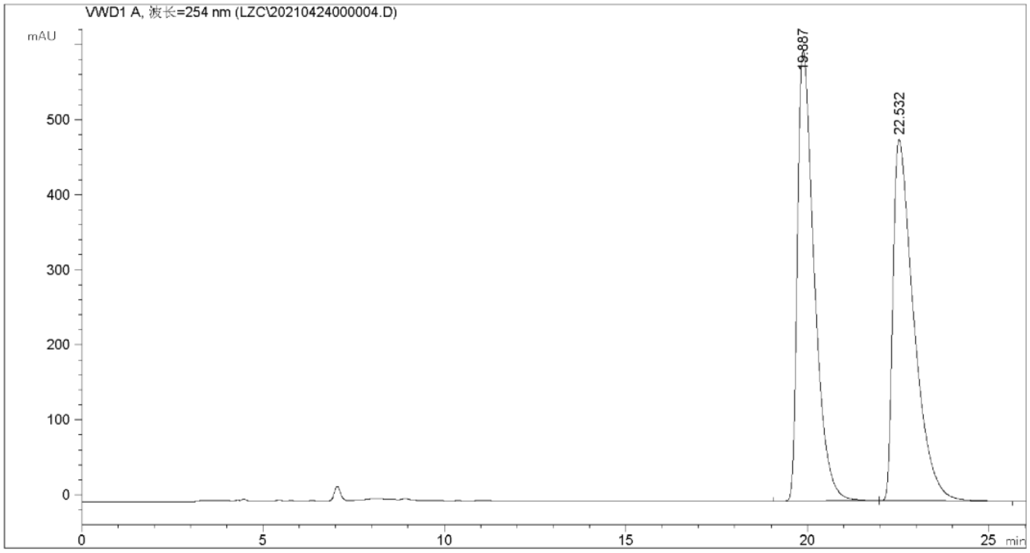

<Peak Table>

Detector A 254 nm

Peak#	Ret. Time	Area	Height	Conc.
**1**	19.887	19280	600	50.0428
**2**	22.532	19247	481	49.9572
**Total**		38527	1081	

Supplementary Figure 162. <Chromatogram>

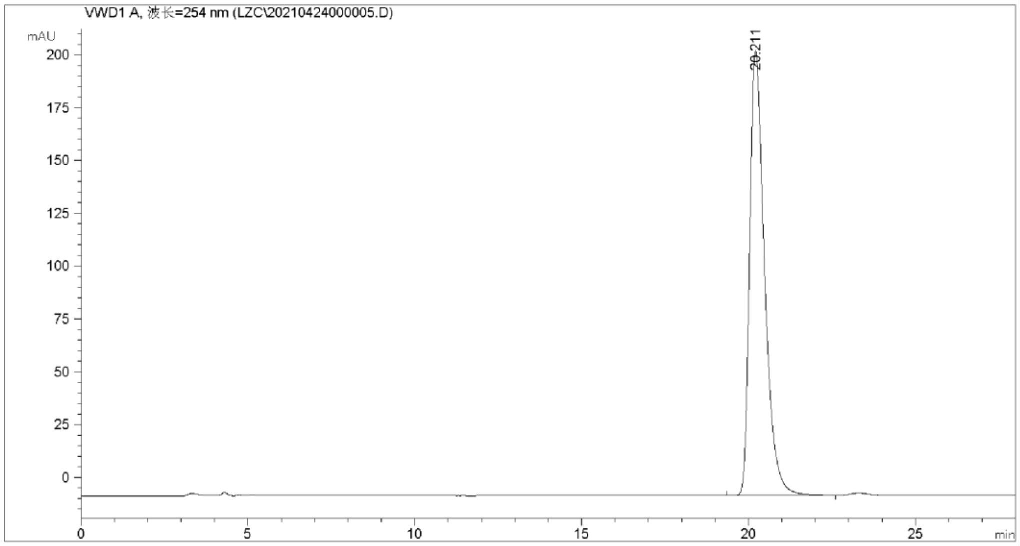

<Peak Table>

Detector A 254 nm

Peak#	Ret. Time	Area	Height	Conc.
**1**	20.211	6529	210	100
**Total**		6529	210	

Supplementary Figure 163. 4m: IC-3 column (hexane/isopropanol = 95/5, flow = 1.0 mL/min) 254 nm

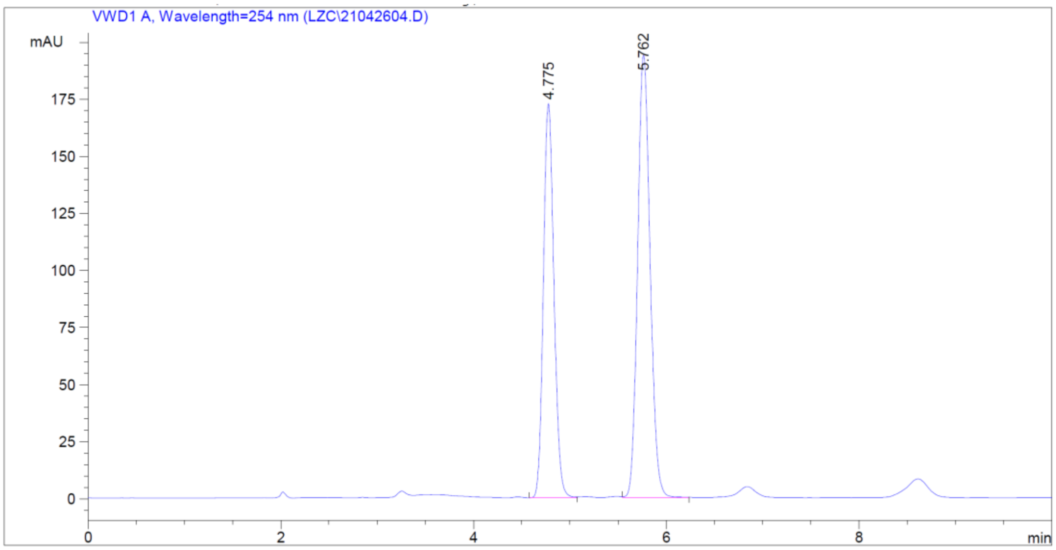

<Peak Table>

Detector A 254 nm

Peak#	Ret. Time	Area	Height	Conc.
**1**	4.775	1311	172	42.9198
**2**	5.762	1744	193	57.0802
**Total**		3055	365	

Supplementary Figure 164. <Chromatogram>

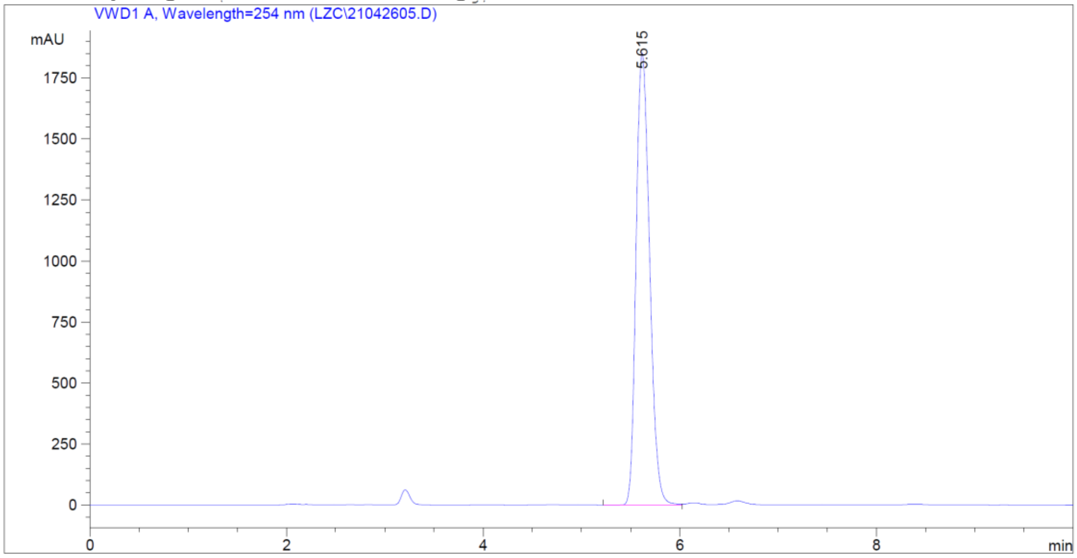

<Peak Table>

Detector A 254 nm

Peak#	Ret. Time	Area	Height	Conc.
1	5.615	17699	1851	100
Total		17699	1851	

Supplementary Figure 165. 4n: AD-H column (hexane/isopropanol = 80/20, flow = 1.0 mL/min) 254 nm

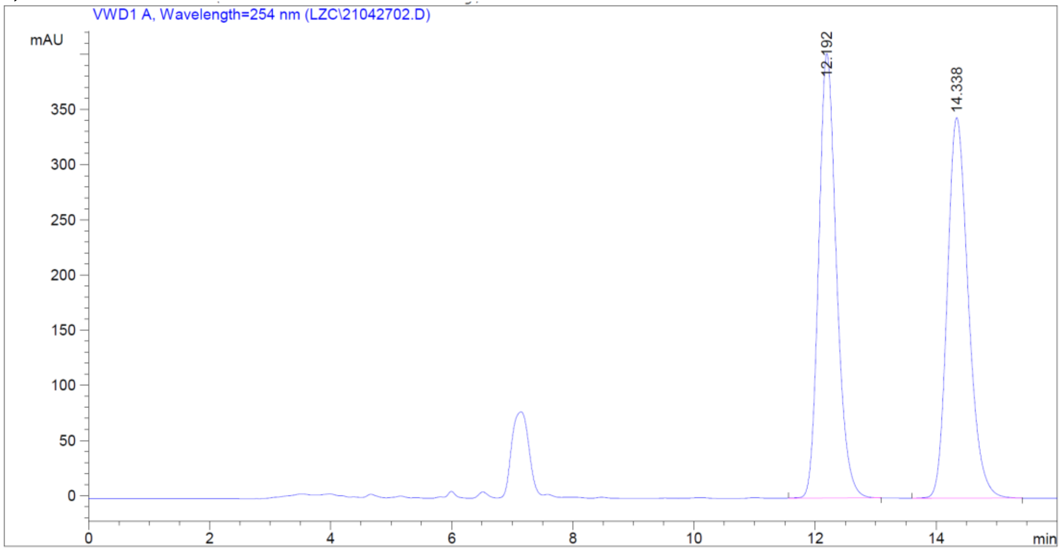

<Peak Table>

Detector A 254 nm

Peak#	Ret. Time	Area	Height	Conc.
1	12.195	8244	402	49.7506
2	15.338	8326	344	50.2494
Total		16570	746	

Supplementary Figure 166. <Chromatogram>

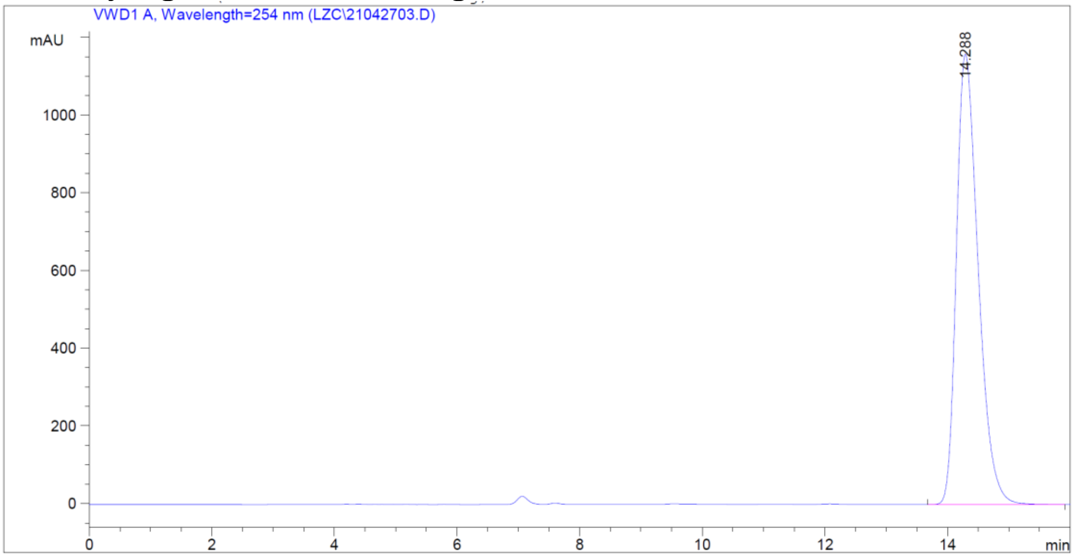

<Peak Table>

Detector A 254 nm

Peak#	Ret. Time	Area	Height	Conc.
**1**	14.288	28652	1159	100
**Total**		28652	1159	

3. Supplementary References

1. Kim, I.; Lee, S.H.; Lee S. *Tetrahedron Lett*. **49**, 6579–6584 (2008).
2. Collins, Elizabeth, Aaron *et al.*, **2006**, WO 2006/107784.
3. Chen, X.; Liu, X.; Martinez, J. S.; Mohr, J. T. *Tetrahedron* **72**, 3653–3665 (2016).
4. Foster, R. T.; Robertson, A.; Bushra, A. 457. *Journal of the Chemical Society (Resumed)*, **1948**, 2254–2260 (1948).
5. Jones, G. H.; Mackenzie, J. B. D.; Robertson, A.; Whalley, W. B. 121. *Journal of the Chemical Society (Resumed)*, **1949**, 562–569 (1948).
6. Kenji, F.; Mitsuru, N.; Sachihiko, T. *Bull. Chem. Soc. Japan* **42**, 1971–1975 (1969).
7. Cherkaoui, O.; Nebois, P.; Fillion, H. *Chem. Pharma. Bull*. **45**, 457–458 (1997).

8. Santini C.; Berger, G. D.; Han, W.; Mosley, R.; MacNaul, K.; Berger, J.; Doebber, T.; Wu, M.; Moller, D. E.; Tolman, R. L.; Sahoo, S. P. *Bioorg. Med. Chem. Lett.* **13**, 1277–1280 (2003).

9. Sahoo, S. P.; Santini, C.; Boueres, J. K.; James, H. V., E. Metzger, **2000**, WO 00/78312 A1

10 Gaussian 09, Revision A.02, M. J. Frisch, G. W. Trucks, H. B. Schlegel, G. E. Scuseria, M. A. Robb, J. R. Cheeseman, G. Scalmani, V. Barone, G. A. Petersson, H. Nakatsuji, X. Li, M. Caricato, A. Marenich, J. Bloino, B. G. Janesko, R. Gomperts, B. Mennucci, H. P. Hratchian, J. V. Ortiz, A. F. Izmaylov, J. L. Sonnenberg, D. Williams-Young, F. Ding, F. Lipparini, F. Egidi, J. Goings, B. Peng, A. Petrone, T. Henderson, D. Ranasinghe, V. G. Zakrzewski, J. Gao, N. Rega, G. Zheng, W. Liang, M. Hada, M. Ehara, K. Toyota, R. Fukuda, J. Hasegawa, M. Ishida, T. Nakajima, Y. Honda, O. Kitao, H. Nakai, T. Vreven, K. Throssell, J. A. Montgomery, Jr., J. E. Peralta, F. Ogliaro, M. Bearpark, J. J. Heyd, E. Brothers, K. N. Kudin, V. N. Staroverov, T. Keith, R. Kobayashi, J. Normand, K. Raghavachari, A. Rendell, J. C. Burant, S. S. Iyengar, J. Tomasi, M. Cossi, J. M. Millam, M. Klene, C. Adamo, R. Cammi, J. W. Ochterski, R. L. Martin, K. Morokuma, O. Farkas, J. B. Foresman, and D. J. Fox, Gaussian, Inc., Wallingford CT, **2016**.

11 (a) C. Lee, W. Yang, R. G. Parr, *Phys. Rev. B* **1988**, *37*, 785; (b) A. D. Becke, *J. Chem. Phys.* **1993**, *98*, 5648.

12 S. Grimme, J. Antony, S. Ehrlich, H. Krieg, *J. Chem. Phys.* **2010**, *132*, 154104.

13 (a) F. Weigend, R. Ahlrichs, *Phys. Chem. Chem. Phys.* **2005**, *7*, 3297; (b) F. Weigend, *Phys. Chem. Chem. Phys.* **2006**, *8*, 1057.

14 A. V. Marenich, C. J. Cramer, D. G. Truhlar, *J. Phys. Chem. B* **2009**, *113*, 6378.

15 CYLview20; C. Y. Legault, Université de Sherbrooke, **2020** (http://www.cylview.org).
